# Gold-Catalyzed Cyclizations of 4‑Alkyl-2-yn-1-yl (Oxy)cyclohexa-2,5-dien-1-ones (**1**) to Develop Two Cyclizations via π‑Alkyne versus π‑Allene Activations

**DOI:** 10.1021/acs.orglett.5c05132

**Published:** 2026-01-13

**Authors:** Akshay Suresh Kshirsagar, Pei-Qin Liao, Tzuhsiung Yang, Rai-Shung Liu

**Affiliations:** † Department of Chemistry, 34881 National Tsing Hua University, Hsinchu 300, Taiwan (ROC); ‡ Shanghai Academy of Artificial Intelligence for Science, Shanghai 200433, China

## Abstract

This work reports two new gold-catalyzed cyclizations of 4-alkyl-2-yn-1-yl (oxy)­cyclohexa-2,5-dien-1-ones (**1**) via gold−π-allene versus gold−π-alkyne routes, The π-allene route involves a [3.3] Claisen rearrangement of 1-alloxyallene intermediates to enable a 1,3-group migration, ultimately affording 2-substituted 3-formylbenzofurans (**2**). Density functional theory (DFT) calculations support this proposed mechanism in which water plays a key role. An alternative π-alkyne route contains cocatalyst PhNH_2_ (10 mol %), leading to the formation of C_3,4_-fused tetrahydrobenzofuran-5­(4*H*)-ones (**3**) stereoselectively.

## Graphical abstract

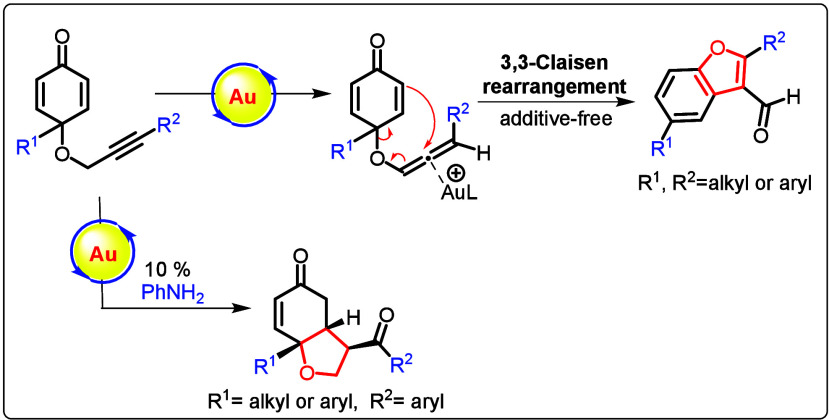

Benzofurans along with dihydrobenzofurans make up an important class of heterocyclic scaffolds.^1^ A representative family is 2-aryl-3-formylbenzofurans (**iv**) that are well fitted to bioactive molecules (**i–iii**), as shown in Figure 1. Typically, benzofurans undergo electrophilic additions at C(2), whereas bioactive benzofurans (**i–iii**) have an aldehyde at C(3).^1^ Chemical synthesis of 2-aryl-3-formylbenzofurans (**iv**) relies on special reagents such as 2-methoxy-3-formylbenzofurans or 2-(2-methoxyphenyl)-3-oxo-3-phenylpropanal,^2^ which are not readily available from common procedures. Metal-catalyzed cyclizations of 4-alkyl-2-yn-1-yl (oxy)­cyclohexa-2,5-dien-1-ones (**1**) have been considerably studied^3–5^ because their resulting bicyclic products contain a versatile cyclohexenone ring. Apart from Rh­(I), Ir­(I), and Co­(I) catalysts,^3^ much attention has been focused on Lewis acid or Brønsted acid catalysts,^4^ as depicted in eqs 1–3. A 1,2-group migration occurs to form *meta*-substituted phenols when Ag­(I), Cu­(I), and HOTf catalysts activate the ketone groups to generate vinyl cations (**Int-1**) (eq 1)^4^ that undergo subsequent skeletal rearrangement, ultimately forming a main product (**I**). Unfortunately, a valuable bicyclic species (**3**) is produced in only 15% yield using HOTf. Alternatively, an acetate addition at the Pd­(II)−π-alkynes (**Int-2**)^5a^ generates alkenylmetal species (**Int-3**), enabling a ring closure at C(3) to yield *trans*-acetate products (**II**). A broad scope of applicable nucleophiles (X = Cl, Br, I, and OAc) has been well explored to involve a Ru–X insertion into π-alkyne before a similar enone insertion,^5b^ yielding the *cis*-X configured products (**II′**). We apply divergent gold catalysis^5d–5f^ to this system, successfully developing two new cyclizations via π-allene versus π-alkyne activations (eqs 4 and 5). Herein, an initial π-alkyne → π-allene transformation is surprisingly implemented by a gold catalyst and water in hot 1,2-dichloroethane without a strong base.^6^ A gold catalyst is known to catalyze an isomerization of propargyl acetates to allenylacetates,^6^ but not for propargyl ethers. In this new gold catalysis, the formation of biologically significant 2-aryl-3-formylbenzofurans (**2**) involves a remarkable 1,3-group migration through a [3,3] Claisen rearrangement of gold−π-allene species **Int-5**. In the π-alkyne route, our resulting products, C_3,4_-fused tetrahydrobenzofuran-5­(4*H*)-ones (**3**), are not directly available from other metal catalysts in eqs 1–3, manifesting additional value. To verify the π-allene mechanism, DFT calculations are performed to conduct a novel [3,3] Claisen rearrangement of gold−π-allenes as the key step. In a previous paper by Toste,^7^ a [3,3] Claisen rearrangement is described for vinyloxy propargyl species (eq 6), distinct from allyloxyallene intermediates in this study.

**Figure 1:**
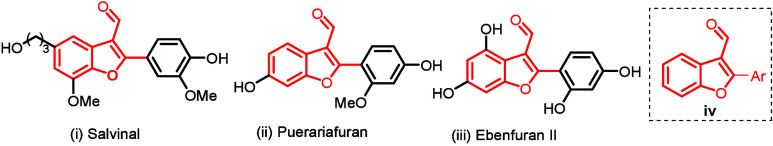
Selected bioactive molecules.

The importance of our gold catalysis is the one-pot synthesis of 2-aryl-3-formylbenzofurans (**2**) using widely used substrates (**1**). Bioactive active molecules comprising this specific core are shown in Figure 1. Salvinal (**i**) is a folk medicine against angina pectoris and acute myocardial infarction.^8a^ Puerariafuran (**ii**) exhibits active secondary metabolites and anti-inflammatory activities,^8b^ whereas Ebenfurans II (**iii**) show antiplasmodial, antioxidant, estrogenic, and anti-HIV activities^8c^ (Figure 1).

Table 1 shows our reaction optimization using Au­(I) and Ag­(I) catalysts. Wet DCE (1,2-dichloroethane) is a mixture of freshly distilled DCE and H_2_O (4 equiv). A wet DCE solution of 4-methyl-4-((3-phenylprop-2-yn-1-yl)­oxy)­cyclohexa-2,5-dien-1-one **1a** (0.14 M) and gold catalysts (2 mol %) was placed in a preheated oil bath at 85 °C for 35–45 h before purification. As shown in Table 1, with an initial trial with P­(*t*-Bu)_2_(*o*-biphenyl)/AgNTf_2_ in wet DCE (85 °C, 40 h), compound **2a** was isolated in 78% yield (entry 1); its molecular structure was revealed by an X-ray diffraction study.^9^ Compound **2a** has a benzofuran framework, but its formation from substrate **1a** involves a novel 1,3-group migration. Changing the ligand as in L′AuCl/AgNTf_2_ (L′ = PPh_3_ or IPr) led to catalytic inactivity. We believe that IPrAu^+^ is not acidic enough whereas PPh_3_Au^+^ is deactivated in such a protracted heating_._ (entries 2 and 3). However, P­(OPh)_3_AuCl/AgNTf_2_ could afford **2a** in 48% yield (entry 4). Changing the silver salts for P­(*t*-Bu)_2_(*o*-biphenyl)­AuCl/AgX (X = OTf or SbF_6_) afforded our target **2a** in 54% and 25% yields, respectively (entries 5 and 6, respectively). Silver-free P­(*t*-Bu)_2_(*o*-biphenyl)­AuCl/NaBARF (BARF = [B­{3,5-(CF_3_)_2_C_6_H_3_}_4_]) is catalytically inactive (entry 7). AgNTf_2_ and AgSbF_6_ alone, each at 10 mol %, afforded *meta*-substituted phenol **2a′** in 15% and 38% yields, respectively (entries 8 and 9, respectively). With LAuCl/AgNTf_2_, the yields of compound **2a** in various wet solvents are as follows: 35% with toluene, 0% with THF, and 0% with MeCN­(entries 10–12, respectively).

**Table 1:**
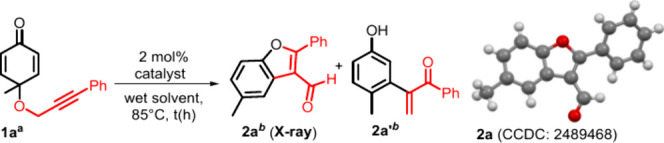
Catalyst Screening and Reaction Conditions^a^

				yield^b^(%)		
entry	catalyst	solvent	time (h)	**1a** ^b^	**2a** ^b^	**2a′** ^b^
1	LAuCl/AgNTf_2_	DCE	40	–	78	–
2	Ph_3_PAuCl/AgNTf_2_	DCE	38	70	trace	–
3	IPrAuCl/AgNTf_2_	DCE	42	65	trace	–
4	(OPh)_3_PAuCl//AgNTf_2_	DCE	40	25	48	–
5	LAuCl/AgOTf	DCE	45	20	54	trace
6	LAuCl/AgSbF_6_	DCE	42	35	25	trace
7	LAuCl/NaBARF	DCE	35	85	–	–
8^c^	AgNTf_2_	DCE	20	55	–	15
9^c^	AgSbF_6_	DCE	20	20	–	38
10	LAuCl/AgNTf_2_	toluene	40	45	35	–
11	LAuCl/AgNTf_2_	THF	40	75	–	–
12	LAuCl/AgNTf_2_	MeCN	40	80	–	–

a Reaction conditions: **1a** (1.0 equiv) in 1,2-CH_2_Cl_2_ (0.14 M) at 85 °C in a preheated oil bath. IPr = 1,3-bis­(diisopropylphenyl) imidazol-2-ylidene. L = P­(*t*-Bu)_2_(*o*-biphenyl). DCE = 1,2-dichloroethane. THF = tetrahydrofuran.

b Product yields are obtained after purification from a silica column.

c With 10 mol % catalyst for two Ag­(I) catalysts.

In Scheme 2, we assess the substrate scope for various 4-alkyl-2-yn-1-yl (oxy)­cyclohexa-2,5-dien-1-ones **1** using LAuCl/AgNTf_2_ (2 mol %; L = P­(*t*-Bu)_2_(*o*-biphenyl)) in 1,2-dichloroethane at 85 °C. We prepared substrates **1b**–**1d** bearing *p*-phenyl groups, (*p-*C_6_H_4_X, where X = OMe, Me, or Cl), affording the desired products **2b**–**2d**, respectively, in 71–82% yields. We also prepared new substrates **1e** and **1f** containing *m*-phenyl substituents (*m-*C_6_H_4_X, where X = Me or Cl); their corresponding products **2e** and **2f** were isolated in 74% and 69% yields, respectively. Furthermore, substrates bearing an *o*-phenyl **1g** (*o-*C_6_H_4_OMe) delivered product **2g** in 69% yield. 1-Naphthalene- and 2-thiophene-containing substrates **1h** and **1i** proved to be fully compatible to deliver benzofuran derivatives **2h** and **2i** in 72% and 75% yields, respectively. For three alkylalkyne-containing substrates **1j**–**1l** (alkyl = *n*-Bu, *c*-Pr, and cyclohexyl, respectively), the transformation proceeded smoothly to yield products **2j–2l**, respectively, in 76–80% yields. The molecular structure of product **2k** was confirmed by X-ray diffraction analysis.^9^ A C(4)-phenyl substituent on the cyclohexadienone ring as in substrate **1m** afforded product **2m** in 80% yield. Additional alkyl chains (**1n**–**1p**, where R^1^ = *n*-Bu, Et, or *i*-Pr) at C(4) of dienone furnished expected products **2n**–**2p** in satisfactory yields (72–76%). Finally, we examined the tolerance of functional groups, including ketone-, aldehyde-, and cyano-containing substrates **1q**–**1s**, respectively, and their products **2q**–**2s** were obtained in 60%, 38%, and 48% yields, respectively. Low yields of products **2r** and **2s** likely arise from the coordination of aldehyde and cyano to a gold catalyst to reduce the acidity. A substrate bearing a methyl group **1v** at C(3) was found to be catalytically inactive, failing to deliver compound **2t**.
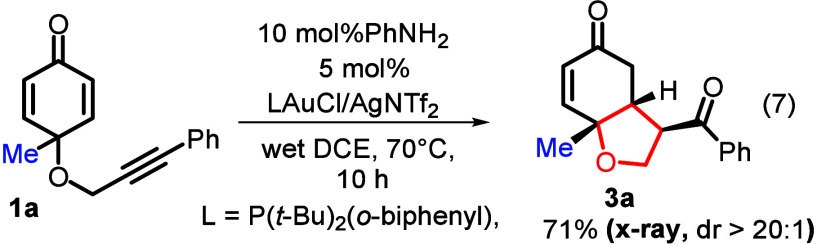


With a cyclohexadienone substrate **1a**, a gold catalyst in wet DCE, the presence of PhNH_2_ in a catalytic amount (10 mol %) surprisingly altered the reaction chemoselectivity; herein, water is a reaction partner. Tetrahydrobenzofuran-5­(4*H*)-one **3a** is created stereoselectively with dr > 20:1 and 71% yield (eq 7); its molecular structure was confirmed by an X-ray diffraction study.^9^

In the Pd­(II) catalysis (Scheme 1, eq 2), compound **3a** could also be obtained from K_2_CO_3_ hydrolysis of its vinylacetate analogue (**II**), and this route was completed in two steps with an overall 72% yield, which is close to our value (71%). However, our system employs only 10% PhNH_2_, instead of HOAc, benzoquinone, or K_2_CO_3_ in excessive proportion (1–3 equiv) in two separate reactions. Accordingly, we expand this new PhNH_2_/Au­(I) catalysis using selected substrates **1** in Scheme 2. We employed the standard condition in Table S1.

**Scheme 1:**
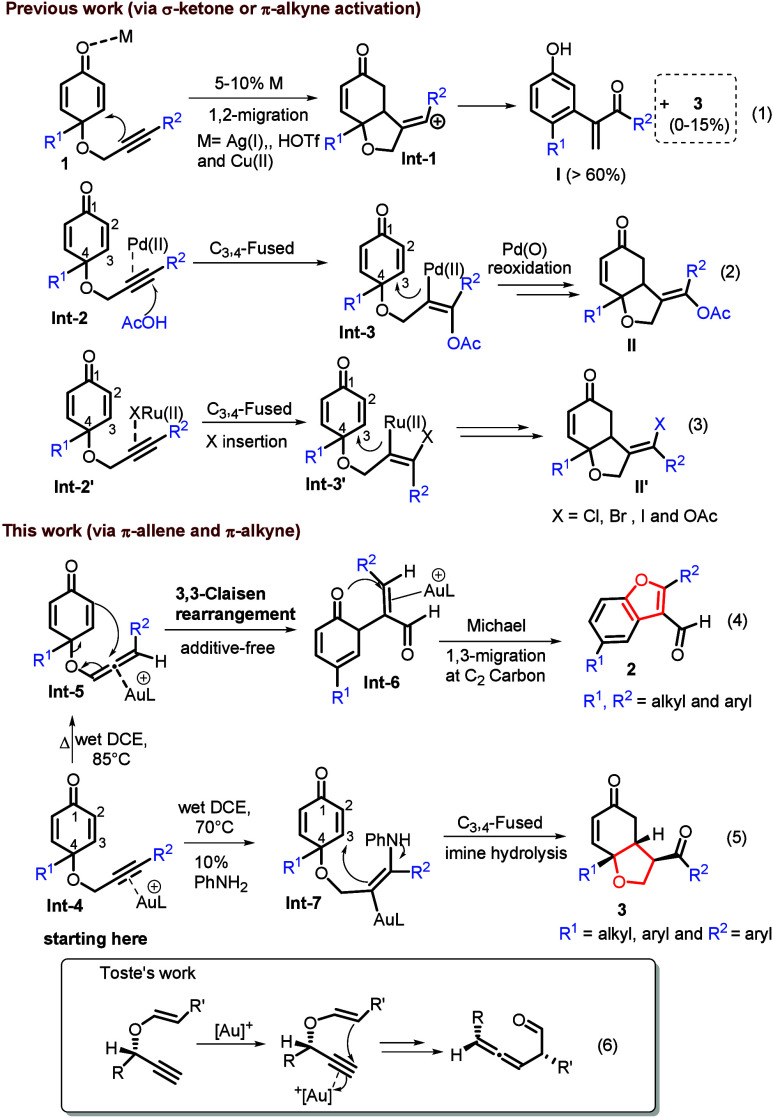
Types of Catalytic Reactions on Substrates (**1**)

**Scheme 2:**
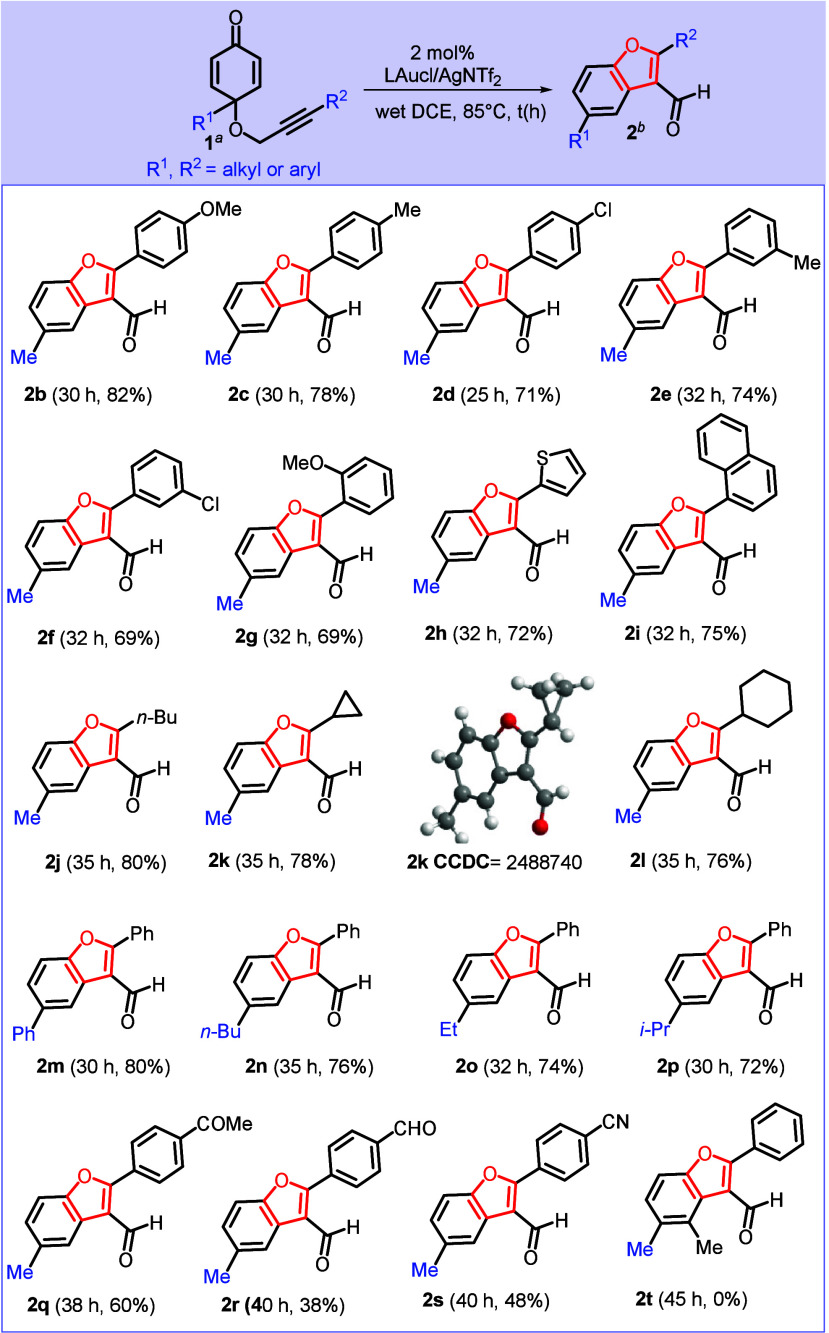
Substrate Scope for Benzofuran Synthesis^a^

a [**1**] = 0.14 M. L = P­(*t*-Bu)_2_(*o*-biphenyl). DCE = 1,2-dichloroethane.
b Product yields are reported after silica column.

In Scheme 3, substrates bearing a *p*-phenyl group, **1c** and **1d** (*p-*C_6_H_4_X, where X = Me and Cl), delivered their tetrahydrobenzofuran-5­(4*H*)-one derivatives **3b** and **3c** in 78% and 69% yields, respectively. For substrates **1e** and **1f** containing *m*-phenyl substituents (*m-*C_6_H_4_X, where X = Me or Cl), their desired products **3d** and **3e** were obtained in 70% and 65% yields, respectively. For *o*-phenyl substrate **1g** (*o-*C_6_H_4_OMe), we managed to isolate the desired product **3f** in 70% yield. For substrates bearing C(4)-alkyl groups (R^1^ = *i*-Pr or *n*-Bu), their corresponding products **3g** and **3h** were obtained in 62% and 70% yields, respectively. Additional substrates **1t** and **1u** bearing one or two methyl groups at C(2) or C(6) also afforded products **3i** and **3j** in 72% and 75% yields, respectively. In addition, the substrate containing 2-thiophene **1h** delivered compound **3k** in 60% yield; changing the substitution at C(4) of dienone with a phenyl variant is also catalytically effective to produce tetrahydrobenzofuran-5­(4*H*)-one **3l** in 78% yield. We tested reactions for substrates **1j** and **1k** containing aliphatic substitution at R^2^ (*n*-Bu or *c*-Pr), albeit leading to catalytic inactivity.

**Scheme 3:**
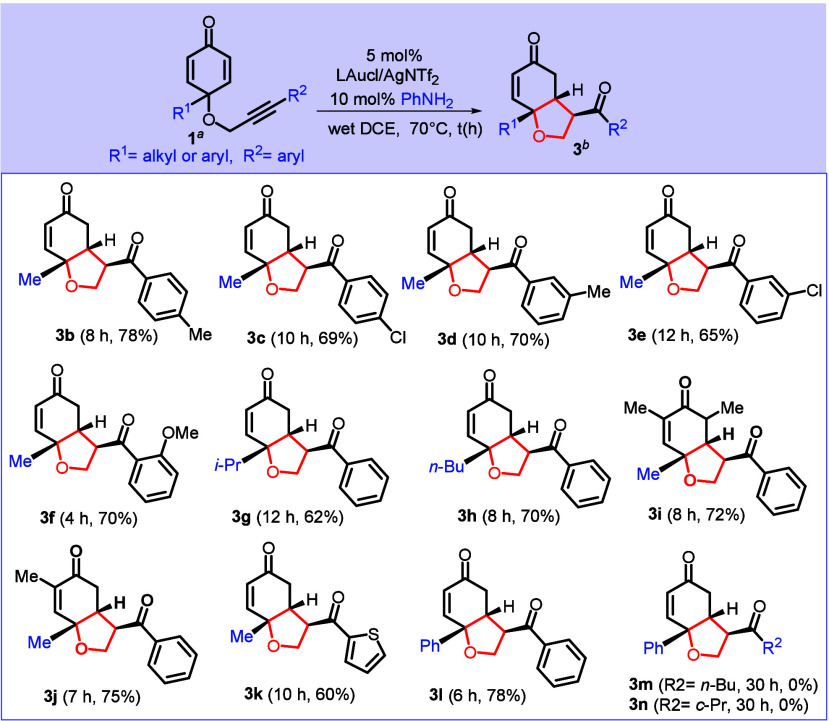
Substrate Scope for Tetrahydrobenzofuran-5­(4*H*)-one Synthesis^a^

a [**1**] = 0.14 M. L = P­(*t*-Bu)_2_(*o*-biphenyl). DCE = 1,2-dichloroethane.
b Product yields are reported after the silica column.

In Scheme 4, this gold catalysis was performed on a large scale using **1a** (2.1 mmol); expected product **2a** was obtained in 68% yield. Treatment of **2a** with PhMgBr and NaBH_4_ efficiently afforded alcohol derivatives **4a** and **4b**, respectively. We also performed chemical functionalization for tetrahydrobenzofuran-5­(4*H*)-one **3a**. Its reaction with *p*-methylthiophenol afforded conjugate addition product **5a** in 80% yield with dr = 1.1:1. Hydrogenation of **3a** with the Pd/C catalyst and H_2_ (1 atm) produced **5b** as a single diastereomer (dr > 20:1); the yield was 76%. Epoxidation of species **3a** with H_2_O_2_/KOH afforded epoxide **5c** as a single diastereomer with dr > 20:1 and 78% yield; the stereochemistry is clarified by ^1^H NOE spectra.

**Scheme 4:**
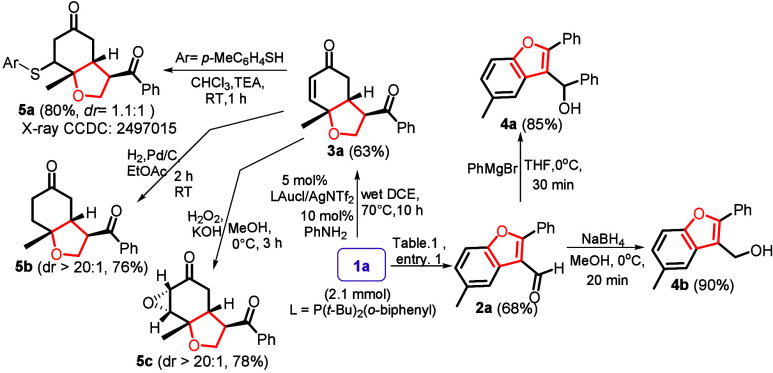
Chemical Functionalizations

We performed one control experiment involving substrate **1g** and PhNH_2_ (1.2 equiv) using P­(*t*-Bu)_2_(*o*-biphenyl)­AuCl/AgNTf_2_ (10 mol) in dry DCE, and a brief heating at 60 °C (4 h) gave 3,4-fused imine product **3f′** in 63% yield (Scheme 5). Without aniline, starting compound **1g** was recovered in 70% yield. With the same gold catalyst, heating imine product **3f′** in wet DCE at 50 °C for 2 h led to imine hydrolysis to afford target **3f** in 96% yield. This reaction sequence supports a mechanism involving an initial attack on Au­(I)−π-alkyne species **Int-4** to deliver intermediate **Int-7** that is expected to give our observed product **3** after a facile imine hydrolysis (eq 8). We performed a crossover experiment to identify an intra- or intermolecular process for the formation of benzofuran derivatives **2**. In a reaction involving starting substrates **1b** and **1n** with an equal proportion, the resulting products contained only benzofurans **2b** and **2n**, respectively (eq 9), indicating an intramolecular process. We also studied the effect of water on the efficiency of this benzofuran synthesis. In dry DCE (4 Å MS), heating substrate **1a** with a Au­(I) catalyst at 85 °C for 30 h led to the formation of compound **3a** in only 14% yield, whereas the presence of water (3 or 5 equiv) greatly increased the yields to 73% or 78%, respectively. Water is sure to play a key role in this benzofuran synthesis (eq 10). In eq 11, P­(*o*-biphenyl)­(*t*-Bu)_2_AuCl/AgNTf_2_ effectively isomerized ethyl 4-phenylbut-3-ynoate **6a** to afford allene derivative **7a** in 55% yield. Heating this catalyst (2 mol %) with methyl propargyl ether (**8a**) in hot DCE (60 °C, 12 h) yielded cinnamaldehyde **10a** in 18% yield (eq 12). We confirmed that the desired allenyl ether (**9a**) quickly formed the same aldehyde (**10a**) with the gold catalyst even at 25 °C in DCE. Accordingly, this gold catalyst is catalytically active toward the alkyne–allene isomerization.

**Scheme 5:**
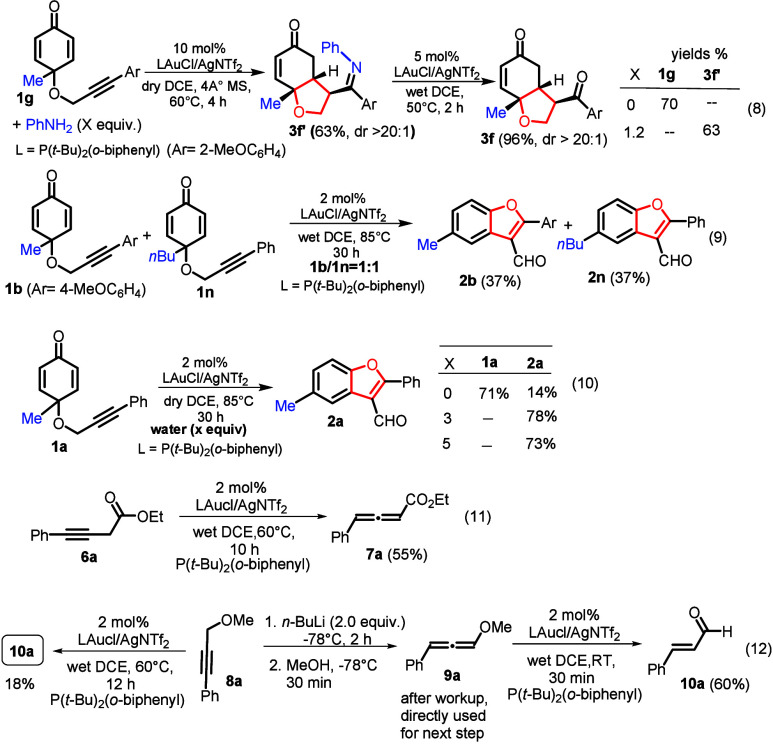
Mechanistic Elucidation

The mechanism of a 1,3-group migration to yield benzofuran **2a** is further resolved by DFT calculations.^10–12^ A transformation of nonacidic propargyl ethers to oxyallenes is feasible by the experiments depicted in eqs 11 and 12. We have performed calculations on one-, two-, and three-water systems; a three-water cluster provides the most satisfactory results. The one-water system has a barrier (**TS1**) that is too high to exist, whereas the two-water system has a barrier that is larger than that of the three-water system by 5.1 kcal/mol. The outcome of the one- and two-water system is provided in Figures S2 and S3. As shown in Scheme 6, the π-alkyne coordination between substrate **1a** and P­(*t*-Bu)_2_(*o*-biphenyl)­Au^+^ to form complex **A** is favored by 13.4 kcal/mol. We postulate an initial deprotonation of complex **A** with three water molecules that fit the simulation of a hydrogen-bonded water cluster in a nonpolar organic solvent. One water acts as a Brønsted base to deprotonate the substrate, while the other two water molecules stabilize the base through hydrogen bonding. This model has been employed in Au-catalyzed alkyne–allene isomerization using highly acidic propargyl species.^10^ This deprotonation step generates allene intermediate **C**, in which the gold catalyst coordinates to the allenyl carbon, via transition state **TS1** with a Gibbs free energy of activation (Δ*G* ^⧧^) of 26.9 kcal/mol. **TS1** is the rate-determining step (RDS) of the reaction. Intermediate **C** subsequently undergoes protodeauration to afford allene intermediate **D**, followed by dissociation of the water cluster to generate **E**. Resulting gold−π-allene intermediate **E** undergoes a 3,3-sigmatropic Claisen-type rearrangement, further yielding intermediate **F** via **TS2** with a Δ*G* ^⧧^ of 21.4 kcal/mol, together with a release of Gibbs free energy of ∼32.8 kcal/mol. A subsequent facile ring within intermediate **F** is expected to form 3,4-dihydrofuran intermediate **G** via **TS3** with a Δ*G* ^⧧^ of 10.5 kcal/mol. **TS3** is facilitated by the saturation of position 6 of the cyclohexadienone, which positions the π* orbital of the cinnamaldehyde in the plane of the lone-pair electrons of the carbonyl oxygen.

**Scheme 6:**
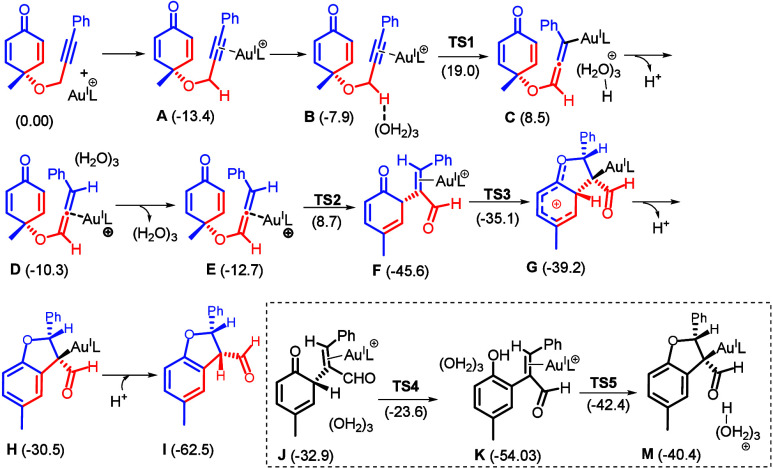
Calculated Potential Energy Surface Using the ωB97X-D4/def2-TZVPP/SMD­(DCE)//TPSS-D4/def2-TZVPP/def2-SVP Method

In the cyclization step from **F** to **G**, the short O···C distance between the ketone oxygen and the benzylic carbon, together with a favorable chairlike conformation of the olefin in **TS3**, may serve as the driving force for the 5-exo-trig cyclization, yielding a furan ring with an envelope-like conformation. Carbocation **G** is then deprotonated by water, aromatizing the six-membered ring to give intermediate **H**. A subsequent protodeauration releases the gold catalyst to afford the desired precursor **I**. Finally, the aerobic oxidation of intermediate **I** delivers final product **2a**. We also calculated an alternative pathway involving the **F–J–K–M–I** sequence. However, this pathway is energetically unfavorable because of the high-energy states of intermediate **J** (−32.9 kcal/mol) and transition state **TS4** (−23.6 kcal/mol). The increased energy of this pathway can likely be attributed to steric interactions of the water cluster in the environment. A complete energy profile diagram for Scheme 6 is provided in Figure S1.

In summary, gold-catalyzed intramolecular cyclizations of 4-methyl-2-yn-1-yl (oxy)­cyclohexa-2,5-dien-1-ones (**1**) proceed through gold−π-allene versus −π-alkyne intermediates. The former process leads to a 1,3-group migration via a [3,3] Claisen rearrangement of gold−π-allene species, affording biologically significant 2-aryl-3-formylbenzofurans (**2**). We have employed DFT calculations to clarify that water plays a crucial role in the transformation of gold−π-alkyne into gold−π-allene. Our second cyclization provides tetrahydrobenzofuran-5­(4*H*)-ones (**3**), which is saves material to meet atom economy, using only 10 mol % aniline.

## Supplementary Material



## Data Availability

The data underlying this study are available in the published article and its Supporting Information.

## Notes

Experimental procedures, characterization data, computational details, and ^1^H and ^13^C NMR spectra of representative compounds (PDF)
