# Supplementary material for: Gold-Catalyzed Cyclizations of 4‑Alkyl-2-yn-1-yl (Oxy)cyclohexa-2,5-dien-1-ones (1) to Develop Two Cyclizations via π‑Alkyne versus π‑Allene Activations
Source: Org Lett. 2026 Jan 13;28(3):1048–53. doi: 10.1021/acs.orglett.5c05132 (PMC12836350; doi:10.1021/acs.orglett.5c05132)
Supplement: Supplementary file 1 [file ol5c05132_si_001.pdf]

Supporting Information

Gold-Catalyzed Cyclizations of 4-Alkyl-2-yn-1-yl(oxy)cyclohexa-2,5-dien-1-ones (1) to Develop Two Cyclizations via π-Alkyne versus π-Allene Activations.

Akshay Suresh Kshirsagar,[a] Pei-Qin Liao[a], Tzuhsiung Yang[b]* and Rai-Shung Liu[a]*

[a]College of Semiconductor Research Institute and Department of Chemistry, National Tsing-Hua University, Hsinchu, Taiwan, ROC---------e-mail: rsliu@mx.nthu.edu.tw

[b]Shanghai Academy of Artificial Intelligence for Science, 18, Longyao Road, Shanghai, China---e-mail:yangzixiong@sais.org.cn

Content:

(1) Representative synthetic procedures: ---S2

(2) Standard procedures for catalytic operation and scale up synthesis: ---------------------------S4

(3) Table S1: -- S6

(4) Typical procedure for Chemical Functionalizations and Mechanistic elucidation: -----------S6

(5) NOE of compound **5c and 3f'** :---S12

(6) References: --S13

(7) Spectral data for key compounds: --S14

(8) X-ray crystallographic structure and data for compound (**2a**, **2k**, **3a**, and **5a**): ---------------S32

(9) Computational Details for the DFT calculations and Gibbs free energy profile diagrams: -S66

9.1. Figure S1 (Energy profile in three-water system) -------------------------------------- S67

9.2. Figure S2 (Energy profile in one-water system) -- S68

9.3. Figure S3(Energy profile in two-water system) --------------------------------------- S69

(10) ^{1}H ,^{13}C spectral data of compounds: ---161

1. Representative synthetic procedures:

(a) General procedure:

Unless otherwise noted, all the reactions for the preparation of the substrates were performed in oven-dried glassware under a nitrogen atmosphere with freshly distilled solvents. THF, Toluene, 1, 2-dichloroethane (DCE) and DCM were distilled from Na metal under nitrogen. Other solvents like triethylamine, 1, 2-dichloroethane, Chloroform, EtOH, ethyl acetate, acetonitrile, and hexane were used from commercial sources without further distillation. All other commercial reagents were used without further purification, unless otherwise indicated. ^{1}H NMR and ^{13}C NMR spectra were recorded on a Bruker 400, 500, 600 MHz and Varian 700 MHz spectrometers using chloroform-*d* ($CDCl_3$) and DMSO-d_6 as the internal standards. High resolution mass spectral analysis (HRMS) data were recorded were conducted using a JEOL JMS-T100LP AccuTOF mass spectrometer equipped with an IonSense DART® ionization source and Magnetic Sector Mass Analyzer (MS station) equipped with the EI source. Single-crystal X-ray diffraction intensity data were collected on a Bruker X8 APEX diffractometer equipped with a CCD area detector and Mo Kα radiation (λ = 0.71073 Å) at 100 K; all data calculations were performed by using the PC version of the APEX2 program package. Final R indices were obtained using those reflections $I > 2\sigma(I)$.

(b) General procedure for the synthesis of substrates (1a – 1v):

All Alkyne-Tethered Cyclohexadienones are reported substrates **(1a – 1v)** were prepared according to well known reported methods. [s1]

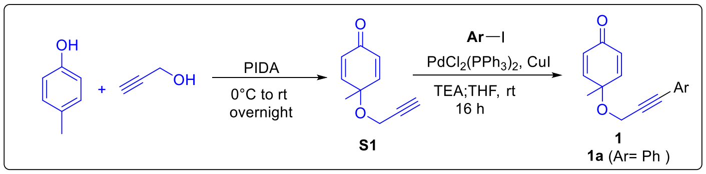

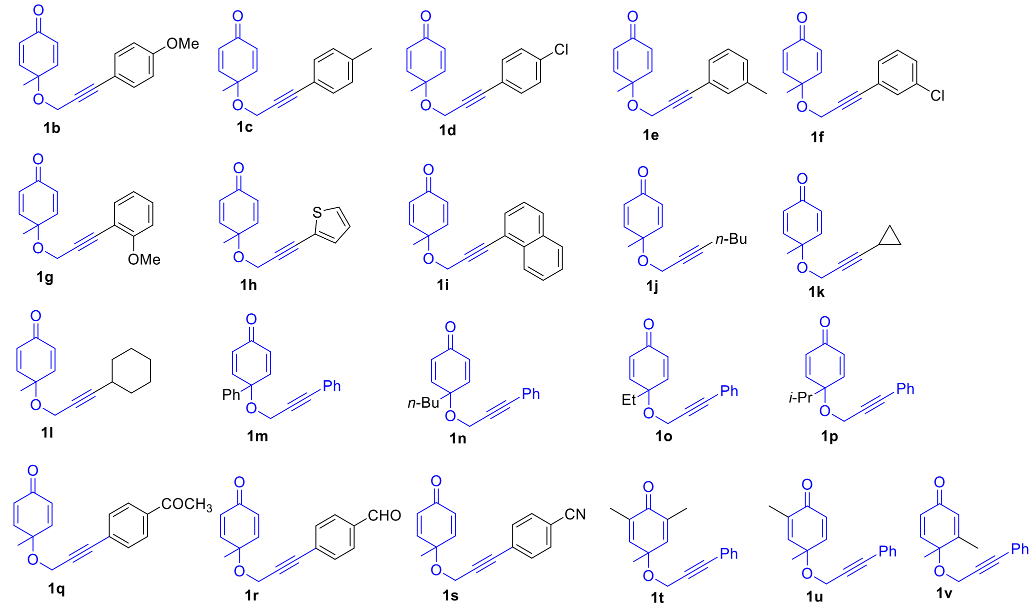

A well-stirred suspension of *p*-substituted phenol (1.0 mmol) in 1 mL of propargyl alcohol was added phenyliodine(III) diacetate (PIDA, 1.2 mmol) in several portions at 0 ^{o}C. The resulting reaction mixture was stirred at room temperature for overnight. Then the reaction mixture was diluted with water (5 mL) and extracted with ethyl acetate (5 mL × 3). The combined organic solvent was washed with brine (5 mL), dried (Na_2SO_4), filtered, and concentrated in vacuo. The crude reaction mixture was purified by column chromatography (EtOAc/hexane) to give the desired products **S1**.

To a suspension of *O*-tethered alkyne **S1** (3.0 mmol) in degassed Et_3N (3 mL) and THF (3 mL) was added $Pd(PPh_3)_2Cl_2$ (3 mol %), CuI (1.5 mol %) and aryl iodide (3.6 mmol). The mixture was stirred at room temperature under a nitrogen atmosphere. The progress of the reaction was monitored by TLC. After the reaction was complete, quenched with water (10 mL), and the mixture was extracted with EtOAc (3 × 20 mL). The combined organic solvent was washed with 10% aqueous HCl to pH=7, dried (Na_2SO_4), filtered, and concentrated in vacuo. The mixture was purified by column chromatography (EtOAc/hexane) to give aryl substituted alkynes **1**.

Aniline, Phenol, Thiophenol, Triethylamine and Phenyl propargyl alcohol derivatives are commercially available from Sigma-Aldrich, Alfa Aesar, Combi-Blocks.

2. Standard procedures for catalytic operation and scale up (2.10 mmol) synthesis:

2.1 (a). Typical procedure for synthesis of 5-methyl-2-phenylbenzofuran-3-carbaldehyde (2a):

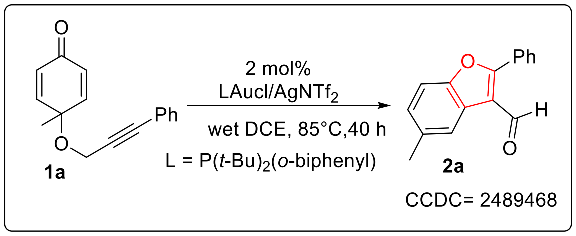

A 10 mL sealed tube was charged with L= P(*t*-Bu)$_2$(*o*-biphenyl)P-AuCl (2.2 mg, 0.0042 mmol, 0.02 equiv.), $AgNTf_2$ (1.6 mg, 0.0042 mmol, 0.02 equiv.), and to this mixture was added wet 1, 2-dichloroethane (0.5 mL). The resulting mixture was stirred at room temperature for 5 min. To this mixture was slowly added a wet 1, 2-dichloroethane (1.0 mL) solution of 4-methyl-4-((3-phenylprop-2-yn-1-yl)oxy)cyclohexa-2,5-dien-1-one **1a** (50 mg, 0.209 mmol); the reaction mixture was further stirred at 85 °C in a pre-heated oil bath for 40 h. The resulting mixture was filtered over a short celite bed, concentrated, and purified through a silica column using ethyl acetate/hexane (5: 95) as the eluent to give 5-methyl-2-phenylbenzofuran-3-carbaldehyde **2a** (38.7 mg, 0.163 mmol, 78%) as a white solid.

(b). Typical procedure for synthesis of (3R,3aR,7aR)-3-benzoyl-7a-methyl-2,3,3a,7a-tetrahydrobenzofuran-5(4*H*)-one (3a):

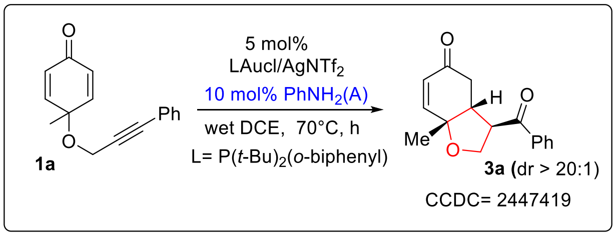

A 10 mL sealed tube was charged with L= P(*t*-Bu)$_2$(*o*-biphenyl)P-AuCl (5.5 mg, 0.0105 mmol, 0.05 equiv.), $AgNTf_2$ (4.07 mg, 0.0105 mmol, 0.05 equiv.) and to this mixture was added wet 1, 2-dichloroethane (0.5 mL). The resulting mixture was stirred at room temperature for 5 min. To

this mixture was added a wet 1, 2-dichloroethane (1.0 mL) solution 4-methyl-4-((3-phenylprop-2-yn-1-yl)oxy)cyclohexa-2,5-dien-1-one **1a** (50 mg, 0.209 mmol) and promoter aniline **A** (1.95 mg, 0.0209 mmol, 0.1 equiv.); the reaction mixture was further stirred at 70 °C in a pre-heated oil bath for 10 h. The resulting mixture was filtered over a short celite bed, concentrated, and purified through a silica column using ethyl acetate/hexane (15: 85) as the eluent to give compound (3R,3aR,7aR)-3-benzoyl-7a-methyl-2,3,3a,7a- tetrahydrobenzofuran-5(4*H*)-one **3a** (38.2 mg, 0.149 mmol, 71%) as a white solid.

2.2 Scale up synthesis:

(a). A scale up synthesis (2.10 mmol) of 5-methyl-2-phenylbenzofuran-3-carbaldehyde (2a):

A 80 mL sealed tube was charged with L= P(*t*-Bu)$_2$(*o*-biphenyl)P-AuCl (22 mg, 0.0420 mmol, 0.02 equiv.), $AgNTf_2$ (16 mg, 0.0420 mmol, 0.02 equiv.), and to this mixture was added wet 1, 2-dichloroethane (5 mL). The resulting mixture was stirred at room temperature for 10 min. To this mixture was slowly added a wet 1, 2-dichloroethane (10 mL) solution of 4-methyl-4-((3-phenylprop-2-yn-1-yl)oxy)cyclohexa-2,5-dien-1-one **1a** (500 mg, 2.10 mmol); the reaction mixture was further stirred at 85 °C in a pre-heated oil bath for 40 h. The resulting mixture was filtered over a short celite bed, concentrated, and purified through a silica column using ethyl acetate/hexane (5: 95) as the eluent to give 5-methyl-2-phenylbenzofuran-3-carbaldehyde **2a** (338 mg, 1.43 mmol, 68%) as a white solid.

(b). A scale up synthesis (2.10 mmol) of (3R,3aR,7aR)-3-benzoyl-7a-methyl-2,3,3a,7a-tetrahydrobenzofuran-5(4*H*)-one (3a):

A 80 mL sealed tube was charged with L= P(*t*-Bu)$_2$(*o*-biphenyl)P-AuCl (55.1 mg, 0.105 mmol, 0.05 equiv.), $AgNTf_2$ (40.7 mg, 0.105 mmol, 0.05 equiv.) and to this mixture was added wet 1, 2-dichloroethane (5 mL). The resulting mixture was stirred at room temperature for 10 min. To this mixture was added a wet 1, 2-dichloroethane (10 mL) solution 4-methyl-4-((3-phenylprop-2-yn-1-yl)oxy)cyclohexa-2,5-dien-1-one **1a** (500 mg, 2.10 mmol) and promoter aniline **A** (19.5 mg, 0.210 mmol, 0.1 equiv.); the reaction mixture was further stirred at 70 °C in a pre-heated oil bath for 10 h. The resulting mixture was filtered over a short celite bed, concentrated, and purified through a silica column using ethyl acetate/hexane (15: 85) as the eluent to give compound (3R,3aR,7aR)-3-benzoyl-7a-methyl-2,3,3a,7a- tetrahydrobenzofuran-5(4*H*)-one **3a** (340 mg, 1.33 mmol, 63%) as a white solid.

3. Table S1. The change of reaction chemoselectivity with $ArNH_2$.

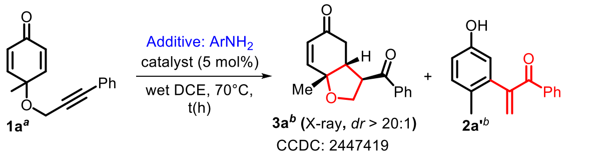

Entry	Catalyst	Additive[c] (mol%)	time (h)	yield[b](%) 3a[b]	2a'[b]
1	LAuCl/$AgNTf_2$	A (20)	24	52	--
2	**LAuCl/$AgNTf_2$**	**A (10)**	**10**	**71**	--
3	$Ph_3PAuCl/AgNTf_2$	A (10)	10	40	--
4	LAuCl/$AgNTf_2$	B (10)	08	58	--
5	LAuCl/$AgNTf_2$	C (10)	10	54	--
6	$AgSbF_6$	A (10)	20	05	15
7	LAuCl/$AgNTf_2$	D (10)	10	00	--
8	LAuCl/$AgNTf_2$	E (10)	10	00	--
9	LAuCl/$AgNTf_2$	F (10)	10	00	--

[a]Reaction conditions: **1a** (1 equiv) in 1,2 CH_2Cl_2 (0.14M) at 70 °C in pre-heated oil bath. [b]Product yields are obtained after purification from a silica column. [c]$ArNH_2$ = 4-$XC_6H_4NH_2$, X = H (A), X = OMe (B), X = F (C), NEt_3 (D), PhOH (E), PhSH (F), L = P(*t*-Bu)$_2$(*o*-biphenyl), DCE = 1,2-dichloroethane.

4. Typical procedure for Chemical Functionalizations and Mechanistic elucidation:

4.1. Typical procedure for Chemical Functionalizations

(a). Synthetic procedures for synthesis of (5-methyl-2-phenylbenzofuran-3-yl)(phenyl)methanol (4a)

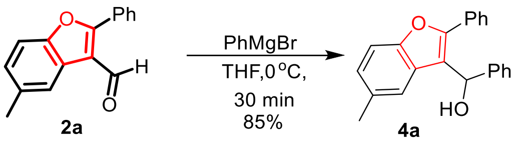

A stirred solution of 5-methyl-2-phenylbenzofuran-3-carbaldehyde **2a** (40 mg, 0.169 mmol) in dry THF (2.0 mL) at 0 °C was added PhMgBr (16 % solution in THF; 31 mg, 0.169 mmol, 1.0 equiv.), and the mixture was stirred at room temperature for 30 min. The resulting mixture was quenched with a saturated solution of NH_4Cl, followed by separation of the organic layer before it was concentrated and purified through a silica column using ethyl acetate/hexane (15: 85) as

the eluent to give compound (5-methyl-2-phenylbenzofuran-3-yl)(phenyl)methanol **4a** (45.5 mg, 0.144 mmol, 85%) as a colorless oil.

(b). Synthetic procedures for synthesis of (5-methyl-2-phenylbenzofuran-3-yl)methanol (4b)

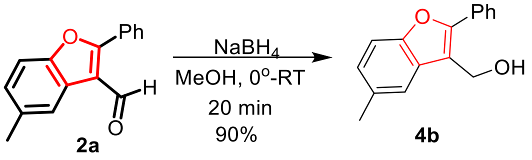

A stirred solution of 5-methyl-2-phenylbenzofuran-3-carbaldehyde **2a** (40 mg, 0.169 mmol) in dry methanol (2.0 mL) at 0 ℃ was added sodium borohydride $NaBH_4$ (3.2 mg, 0.084 mmol, 0.5 equiv.), and the mixture was stirred at 0 ℃ for additional 20 min. The reaction mixture was quenched with a saturated solution of NH_4Cl, followed by separation of organic layer before it was concentrated and purified through a silica column using ethyl acetate/hexane (12: 88) as the eluent to give compound (5-methyl-2-phenylbenzofuran-3-yl)methanol **4b** (36.4 mg, 0.152 mmol, 90%) as a colorless oil.

(c). Synthetic procedures for synthesis of (3R,3aR,7R,7aS)-3-benzoyl-7a-methyl-7-(p-tolylthio)hexahydrobenzofuran-5(4*H*)-one (5a)

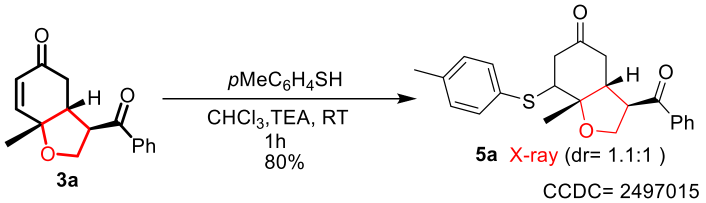

A stirred solution of (3R,3aR,7aR)-3-benzoyl-7a-methyl-2,3,3a,7a- tetrahydrobenzofuran-5(4*H*)-one **3a** (40 mg, 0.156 mmol) in $CHCl_3$ (3 mL) was added *p*-MeC_6H_4SH (29 mg, 0.234 mmol, 1.5 equiv.) and triethylamine (0.032 ml, 0.234 mmol, 1.5 equiv.) subsequently. The reaction mixture was stirred for 1 h. Upon completion, the reaction mixture was then evaporated under vacuum and purified through a silica column using ethyl acetate/hexane (10: 90) as the eluent to give compound (3R,3aR,7R,7aS)-3-benzoyl-7a-methyl-7-(*p*-tolylthio)hexahydrobenzofuran-5(4*H*)-one **5a** (47.6 mg, 0.125 mmol, 80%) as a white solid.

(d). Synthetic procedures for synthesis of (3R,3aR,7aR)-3-benzoyl-7a-methylhexahydrobenzofuran-5(4*H*)-one (5b)

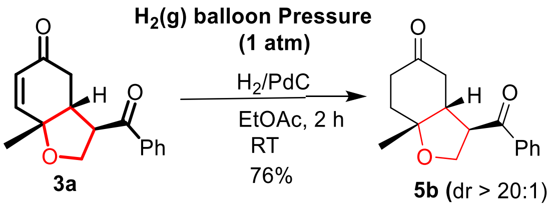

A stirred solution of (3R,3aR,7aR)-3-benzoyl-7a-methyl-2,3,3a,7a- tetrahydrobenzofuran-5(4*H*)-one **3a** (40 mg, 0.156 mmol) in an ethyl acetate (2 mL) was degassed with N_2 for 10min. To this solution Palladium on carbon (Pd/C, 20.0 mg, 10%Wt), was added then reaction vessel was evacuated and then fitted with H_2(g) balloon (1 atm) and reaction was stirred at room temperature for 2 h. Reaction progress was monitored by TLC, after completion reaction mixture was filtered over a short celite bed, concentrated, and purified through a silica column using ethyl acetate/hexane (10: 90) as the eluent to afford (3R,3aR,7aR)-3-benzoyl-7a-methylhexahydrobenzofuran-5(4*H*)-one **5b** (30.7 mg, 0.118 mmol, 76%) as a colorless oil.

(e). Synthetic procedures for synthesis of (3aR,4R,6aS)-4-benzoyl-6a-methylhexahydrooxireno[2,3-g]benzofuran-2(1a*H*)-one (5c)

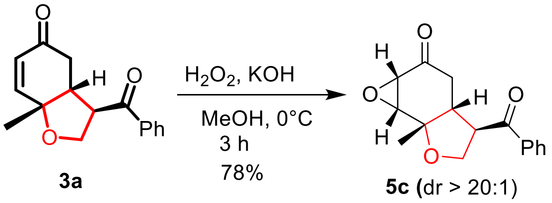

A stirred solution of (3R,3aR,7aR)-3-benzoyl-7a-methyl-2,3,3a,7a- tetrahydrobenzofuran-5(4*H*)-one **3a** (40 mg, 0.156 mmol) and KOH (4.3 mg, 0.078 mmol) were dissolved in 1 mL MeOH in a pressure tube, the reaction was cooled to 0 ℃. Then 30% H_2O_2 solution (13.2 mg, 0.012ml, 0.390 mmol) was then added slowly under stirring and reaction was stirred at 0℃ for 3 h. Reaction progress was monitored by TLC, after completion reaction mixture was filtered over a short celite bed, concentrated, and purified through a silica column using ethyl acetate/hexane (15: 85) as the eluent to afford 3aR,4R,6aS)-4-benzoyl-6a-methylhexahydrooxireno[2,3-*g*]benzofuran-2(1a*H*)-one **5c** (33.3 mg, 0.122 mmol, 78%) as a colorless oil.

4.2. Typical procedure for Mechanistic elucidation

(a). Typical procedure for synthesis of (3S,3aR,7aR)-3-((E)-(2-methoxyphenyl)(phenylimino)methyl)-7a-methyl-2,3,3a,7a-tetrahydrobenzofuran-5(4*H*)-

one (3f') and (3R,3aR,7aR)-3-(2-methoxybenzoyl)-7a-methyl-2,3,3a,7a-tetrahydrobenzofuran-5(4*H*)-one (3f)

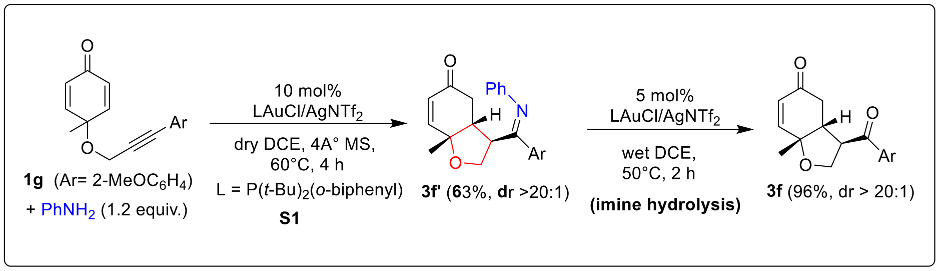

To a suspension of L= P(*t*-Bu)$_2$(*o*-biphenyl)P-AuCl (9.8 mg, 0.0186 mmol, 0.1 equiv.), $AgNTf_2$ (7.2 mg, 0.0186 mmol, 0.1 equiv.) in dry DCE (0.5 mL) was fitted with an N_2 balloon; this mixture was stirred for 5 min at room temperature. To this mixture was added a dry DCE (1.5 mL) solution of 4-((3-(2-methoxyphenyl)prop-2-yn-1-yl)oxy)-4-methylcyclohexa-2,5-dien-1-one **1g** (50 mg, 0.186 mmol) and aniline (20.8 mg, 0.223 mmol, 1.2 equiv.); the resulting mixture was further stirred at 60°C for 4 h. The resulting mixture was filtered over a short celite bed, concentrated, and purified through a silica column using ethyl acetate/hexane (15: 85) as the eluent to afford compound (3S,3aR,7aR)-3-((E)-(2-methoxyphenyl)(phenylimino)methyl)-7a-methyl-2,3,3a,7a-tetrahydrobenzofuran-5(4*H*)-one **3f'** (42.5 mg, 0.117 mmol, 63%) as a brown oil.

(**Imine hydrolysis**): To a suspension of L= P(*t*-Bu)$_2$(*o*-biphenyl)P-AuCl (3.1 mg, 0.0058 mmol, 0.05 equiv.), $AgNTf_2$ (2.2 mg, 0.0058 mmol, 0.05 equiv.) in wet DCE (1 mL); this mixture was stirred for 5 min at room temperature. To this mixture was added a wet DCE (1.5 mL) solution of (3S,3aR,7aR)-3-((E)-(2-methoxyphenyl)(phenylimino)methyl)-7a-methyl-2,3,3a,7a-tetrahydrobenzofuran-5(4*H*)-one **3f'** (42.5 mg, 0.117 mmol); the resulting mixture was further stirred at 50°C for 2 h. The resulting mixture was filtered over a short celite bed, concentrated, and purified through a silica column using ethyl acetate/hexane (10: 90) as the eluent to afford compound (3R,3aR,7aR)-3-(2-methoxybenzoyl)-7a-methyl-2,3,3a,7a-tetrahydrobenzofuran-5(4*H*)-one **3f** (32.5 mg, 0.113 mmol, 96%) as a yellow oil.

(b). Typical procedure for crossover experiment

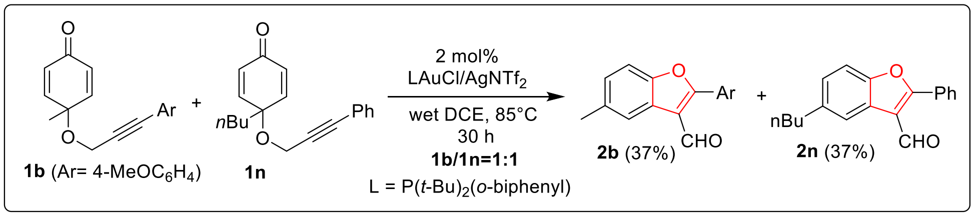

A 10 mL sealed tube was charged with L= P(*t*-Bu)$_2$(*o*-biphenyl)P-AuCl (1.9 mg, 0.0037 mmol, 0.02 equiv.), AgNTf$_2$ (1.4 mg, 0.0037 mmol, 0.02 equiv.), and to this mixture was added wet 1, 2-dichloroethane (0.5 mL). The resulting mixture was stirred at room temperature for 5 min. To this mixture was slowly added a wet 1, 2-dichloroethane (1.5 mL) solution of (**1b/1n=1:1**) 4-((3-(4-methoxyphenyl)prop-2-yn-1-yl)oxy)-4-methylcyclohexa-2,5-dien-1-one **1b** (50 mg, 0.186 mmol) and 4-butyl-4-((3-phenylprop-2-yn-1-yl)oxy)cyclohexa-2,5-dien-1-one **1n** (52 mg, 0.186 mmol); the reaction mixture was further stirred at 85ºC in a pre-heated oil bath for 30 h. The resulting mixture was filtered over a short celite bed, concentrated, and purified through a silica column using ethyl acetate/hexane (10: 90) as the eluent to give 2-(4-methoxyphenyl)-5-methylbenzofuran-3-carbaldehyde **2b** (18.5 mg, 0.069 mmol, 37%) as a yellow oil and 5-butyl-2-phenylbenzofuran-3-carbaldehyde **2n** (19.5 mg, 0.070 mmol, 37%) as a colorless oil.

(c). Synthesis of ethyl 4-phenylbuta-2,3-dienoate (7a)

The ethyl 4-phenylbut-3-ynoate (**6a**) is reported substrates were prepared according to well-known reported methods. [s2]

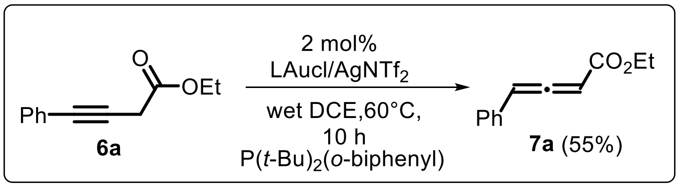

A 10 mL sealed tube was charged with L= P(*t*-Bu)$_2$(*o*-biphenyl)P-AuCl (2.8 mg, 0.0053 mmol, 0.02 equiv.), AgNTf$_2$ (2.0 mg, 0.0053 mmol, 0.02 equiv.), and to this mixture was added wet 1, 2-dichloroethane (0.5 mL). The resulting mixture was stirred at room temperature for 5 min. To this mixture was slowly added a wet 1, 2-dichloroethane (1.0 mL) solution of ethyl 4-phenylbut-3-ynoate **6a** (50 mg, 0.265 mmol); the reaction mixture was further stirred at 60 ˚C in a pre-heated oil bath for 10 h. The resulting mixture was filtered over a short celite bed, concentrated, and purified through a silica column using ethyl acetate/hexane (2: 98) as the eluent to give ethyl 4-phenylbuta-2,3-dienoate **7a** (27.6 mg, 0.146 mmol, 55%) as a colorless oil.

Spectral data for Ethyl 4-phenylbut-3-ynoate (**6a**):

^{1}H NMR (400 MHz, CDCl$_3$): δ 7.43 – 7.41 (m, 2H), 7.29 – 7.26 (m, 3H), 4.22 (q, *J* = 7.1 Hz, 2H), 3.48 (s, 2H), 1.29 (t, *J* = 7.2 Hz, 3H); ^{13}C NMR (100 MHz, CDCl$_3$): δ 168.2, 131.7, 128.2,

128.1, 123.0, 83.4, 81.2, 61.6, 26.7, 14.1, two carbons merged; HRMS-ESI+ calcd for $C_{12}H_{12}O_2$ $[M+H]^+$: 189.0915, found: 189.0912.

Spectral data for Ethyl 4-phenylbuta-2,3-dienoate (**7a**):

^{1}H NMR (400 MHz, $CDCl_3$): δ 7.32 – 7.28 (m, 5H), 6.61 (d, *J* = 6.4 Hz, 1H), 6.00 (d, *J* = 6.4 Hz, 1H), 4.21 (q, *J* = 7.1 Hz, 2H), 1.27 (t, *J* = 7.1 Hz, 3H); ^{13}C NMR (100 MHz, $CDCl_3$): δ 214.6, 165.1, 131.7, 131.1, 128.8, 128.1, 127.5, 98.6, 91.9, 61.1, 14.2, one carbon merged; HRMS-ESI+ calcd for $C_{12}H_{12}O_2$ $[M+H]^+$: 189.0915, found: 189.0915.

(d). Synthesis of cinnamaldehyde (10a)

The (3-methoxyprop-1-yn-1-yl)benzene (**8a**) and (3-methoxypropa-1,2-dien-1-yl)benzene (**9a**) are reported substrates were prepared according to well-known reported methods. [s3]

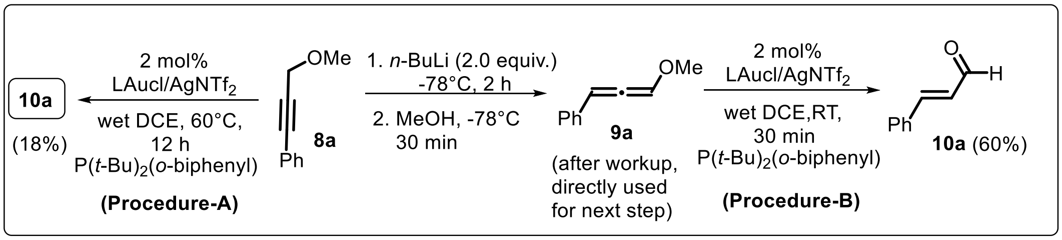

(Procedure-A)

A 10 mL sealed tube was charged with L= P(*t*-Bu)$_2$(*o*-biphenyl)P-AuCl (3.6 mg, 0.0068 mmol, 0.02 equiv.), $AgNTf_2$ (2.6 mg, 0.0068 mmol, 0.02 equiv.), and to this mixture was added wet 1, 2-dichloroethane (0.5 mL). The resulting mixture was stirred at room temperature for 5 min. To this mixture was slowly added a wet 1, 2-dichloroethane (1.0 mL) solution of (3-methoxyprop-1-yn-1-yl)benzene **8a** (50 mg, 0.342 mmol); the reaction mixture was further stirred at 60 °C in a pre-heated oil bath for 12 h. The resulting mixture was filtered over a short celite bed, concentrated, and purified through a silica column using ethyl acetate/hexane (5: 95) as the eluent to give cinnamaldehyde **10a** (8.2 mg, 0.062 mmol, 18%) as a yellow oil.

(Procedure-B)

A 10 mL sealed tube was charged with L= P(*t*-Bu)$_2$(*o*-biphenyl)P-AuCl (3.6 mg, 0.0068 mmol, 0.02 equiv.), $AgNTf_2$ (2.6 mg, 0.0068 mmol, 0.02 equiv.), and to this mixture was added wet 1, 2-dichloroethane (0.5 mL). The resulting mixture was stirred at room temperature for 5 min. To

this mixture was slowly added a wet 1, 2-dichloroethane (1.0 mL) solution of (3-methoxypropa-1,2-dien-1-yl)benzene **9a** (50 mg, 0.342 mmol); the reaction mixture was further stirred at room temperature for 30 min. The resulting mixture was filtered over a short celite bed, concentrated, and purified through a silica column using ethyl acetate/hexane (5: 95) as the eluent to give cinnamaldehyde **10a** (27.2 mg, 0.205 mmol, 60%) as a yellow oil.

Spectral data for (3-methoxyprop-1-yn-1-yl)benzene (8a)

^{1}H NMR (400 MHz, $CDCl_3$): δ 7.45 – 7.42 (m, 2H), 7.31 – 7.29 (m, 3H), 4.30 (s, 2H), 3.44 (s, 3H); ^{13}C NMR (100 MHz, $CDCl_3$): δ 131.7, 128.4, 128.3, 122.6, 86.3, 84.9, 60.4, 57.6, two carbons merged; HRMS-ESI+ calcd for $C_{10}H_{10}O$ $[M+H]^+$: 147.0809, found: 147.0811.

Spectral data for (3-methoxypropa-1,2-dien-1-yl)benzene (9a)

(Note: Spectral data of **9a** were recorded immediately after workup and further directly used for next step (found unstable during column purification))

^{1}H NMR (400 MHz, $CDCl_3$): δ 7.41 – 7.38 (m, 2H), 7.35 – 7.30 (m, 2H), 7.26 – 7.24 (m, 1H), 7.08 (d, *J* = 5.6 Hz, 1H), 6.81 (d, *J* = 5.5 Hz, 1H), 3.45 (s, 3H); ^{13}C NMR (100 MHz, $CDCl_3$): δ 194.9, 135.3, 131.7, 128.6, 128.3, 127.9, 127.2, 125.5, 109.0, 56.0; HRMS-ESI+ calcd for $C_{10}H_{10}O$ $[M+H]^+$: 147.0809, found: 147.0811.

Spectral data for cinnamaldehyde (10a)

^{1}H NMR (400 MHz, $CDCl_3$): δ 9.68 (d, *J* = 7.7 Hz, 1H), 7.55 – 7.52 (m, 2H), 7.47 – 7.39 (m, 4H), 6.70 (dd, *J* = 15.9, 7.7 Hz, 1H); ^{13}C NMR (100 MHz, $CDCl_3$): δ 193.6, 152.7, 134.0, 131.2, 129.1, 128.5, three carbons merged; HRMS-ESI+ calcd for C_9H_8O $[M+H]^+$: 133.0653, found: 133.0655.

5. NOE data:

(a) NOE of compound 5c:

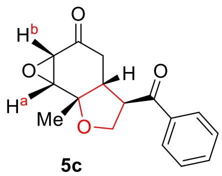

5c

Sr. No.	Irradiation	Intensity increase % (Key peaks)

1.	H[a] (δ 3.45)	H[b] (δ 3.36, 6.63%), Me (δ 1.65, 5.02%)
2.	H[b] (δ 3.36)	H[a] (δ 3.45, 13.32%)

(b) NOE of compound 3f':

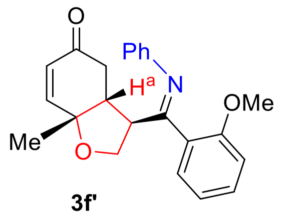

Sr. No.	Irradiation	Intensity increase % (Key peaks)
1.	H[a] (δ 3.13)	Me (δ 1.55, 77.29%), N-Phenyl protons (δ 6.64 – 6.61, 59.65%)
2.	Me (δ 1.55)	H[a] (δ 3.13, 26.78%), N-Phenyl protons (δ 6.64 – 6.61, 25.04%)

6. References:

[S1] (1) Mallick, R. K.; Vangara, S.; Kommu, N.; Guntreddi, T.; Sahoo, A. K. Lewis Acid-Driven Meyer–Schuster-Type Rearrangement of Yne-Dienone. *J. Org. Chem.* **2021**, *86*, 7059−7068. (2) Rai, A.; Das, U. Accessing *meta*-Enone-Substituted Anisoles Using ArN_2BF_4 as Precatalyst via Rearrangement of Alkyne-Tethered Cyclohexadienones. *Synthesis* **2024**, *56* (16), 2499–2506. (3) Munakala, A.; Chegondi, R. Silver(I)-Catalyzed Enyne Cyclization/Aromatization of Alkyne-Tethered Cyclohexadienones to Access *meta*-Substituted Phenols. *Org. Lett.* **2021**, *23*, 317–323. (4) Gollapelli, K. K.; Donikela, S.; Manjula, N.; Chegondi, R. *ACS Catal.* **2018**, *8*, 1440−1447. (5) Munakala, A.; Gollapelli, K. K.; Nanubolu, J. B.; Chegondi, R. Silver(I)-Catalyzed Oxidative Intramolecular Cyclopropanation: Access to Complex Tricyclo[3.3.1.0]nonanediones via Semipinacol-Type Rearrangement. *Org. Lett.* **2020**, *22*, 7019–7024. (6) Gollapelli, K. K.; Donikela, S.; Manjula, N.; Chegondi, R. Rhodium-Catalyzed Highly Regio- and Enantioselective Reductive Cyclization of Alkyne-Tethered Cyclohexadienones. *ACS Catal.* **2018**, *8*, 1440−1447. (7) Kumar, R.; Hoshimoto, Y.; Tamai, E.; Ohashi, M.; Ogoshi, S. Two-Step Synthesis of Chiral Fused Tricyclic Scaffolds from Phenols via Desymmetrization on Nickel. *Nat. Commun.* **2017**, *8*, 32. (8) Clarke, C.; Incerti-Pradillos, C. A.; Lam, H. W. Enantioselective Nickel-Catalyzed anti-Carbometallative Cyclizations of Alkynyl Electrophiles Enabled by Reversible Alkenylnickel E/Z Isomerization. *J. Am. Chem. Soc.* **2016**, *138*, 8068−8071. (9) Singh, A.; Shukla, R. K.; Volla, C. M. R. Palladium-Catalyzed Highly Diastereoselective Cascade Dihalogenation of Alkyne-Tethered Cyclohexadienones via

Umpolung of Palladium Enolate. *Chem. Commun.* **2019**, *55*, 13442–13445. (10) Prasad, V. S.; Ravi, D.; Rao, V. R.; Krishna, S. M.; Kumar, C. A.; Adiyala, P. R. Light-Induced Diastereoselective Ketoesterification to Access 6,5-Fused Tetrahydrobenzofuranones in Batch and Continuous Flow Conditions. *J. Org. Chem.* **2024**, *89*, 12628–12638.

[S2] a) Trinh, T. A.; Fu, Y.; Hu, D. B.; Zappia, S. A.; Guzei, I. A.; Liu, P.; Schomaker, J. M. Chemo- and Enantioselective Intramolecular Silver-Catalyzed Aziridinations of Carbamimidates. *Chem. Commun.* **2024**, *60*, 224−227. b) Suárez, A.; Fu, G. C. A Straightforward and Mild Synthesis of Functionalized 3-Alkynoates. *Angew. Chem. Int. Ed.* **2004**, *43*, 3580−3582.

[S3] Chambers, K. J.; Sanghong, P.; Martos, D. C.; Casoni, G.; Mykura, R. C.; Hari, D. P.; Noble, A.; Aggarwal, V. K. Stereospecific Conversion of Boronic Esters into Enones using Methoxyallene: Application in the Total Synthesis of 10-Deoxymethynolide. *Angew. Chem. Int. Ed.* **2023**, *62*, e202312054.

7. Spectral data of key compound:

a) Spectral data for products:

Spectral data for 5-methyl-2-phenylbenzofuran-3-carbaldehyde (2a)

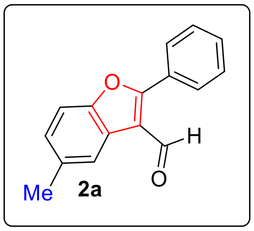

Compound **2a** was purified on a silica gel column using ethyl acetate/hexane: (5: 95) as the eluent; white solid (38.7 mg, 0.163 mmol, 78%); ^{1}H NMR (700 MHz, $CDCl_3$): δ 10.33 (s, 1H), 8.08 (s, 1H), 7.85 – 7.84 (m, 2H), 7.57 – 7.56 (m, 3H), 7.44 (d, *J* = 8.4 Hz, 1H), 7.22 (d, *J* = 8.7 Hz, 1H), 2.49 (s, 3H); ^{13}C NMR (175 MHz, $CDCl_3$): δ 186.8, 165.5, 152.4, 134.6, 131.0, 129.1, 128.7, 127.2, 125.4, 122.4, 117.3, 110.6, 21.3, three carbons merged with aromatic region; HRMS-ESI+ calcd for $C_{16}H_{12}O_2$ $[M+H]^+$: 237.0915, found: 237.0908.

Spectral data for 2-(5-hydroxy-2-methylphenyl)-1-phenylprop-2-en-1-one (2a')

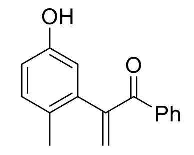

2a'

Compound **2a'** was purified on a silica gel column using ethyl acetate/hexane: (10: 90) as the eluent; yellow solid (19 mg, 0.079 mmol, 38%);^{1}H NMR (400 MHz, $CDCl_3$): δ 7.89 (d, *J* = 8.2 Hz, 2H), 7.57 – 7.53 (m, 1H), 7.46 – 7.42 (m, 2H), 7.02 (d, *J* = 8.2 Hz, 1H), 6.77 – 6.71 (m, 2H), 6.00 (d, *J* = 11.3 Hz, 2H), 5.07 (s, 1H), 2.11 (s, 3H); ^{13}C NMR (100 MHz, $CDCl_3$): δ 196.5, 153.7, 148.9, 139.2, 137.1, 132.7, 131.3, 129.8, 128.3, 128.0, 127.6, 116.7, 115.3, 19.5, two carbons merged; HRMS-ESI+ calcd for $C_{16}H_{14}O_2$ $[M+H]^+$: 239.1072, found: 239.1077.

Spectral data for 2-(4-methoxyphenyl)-5-methylbenzofuran-3-carbaldehyde (2b)

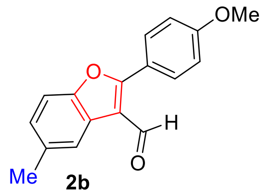

2b

Compound **2b** was purified on silica gel column using ethyl acetate/hexane: (8: 92) as the eluent; yellow oil (41 mg, 0.153 mmol, 82%); ^{1}H NMR (700 MHz, $CDCl_3$): δ 10.30 (s, 1H), 8.05 (s, 1H), 7.82 (d, *J* = 8.8 Hz, 2H), 7.41 (d, *J* = 8.3 Hz, 1H), 7.18 (d, *J* = 6.5 Hz, 1H), 7.07 (d, *J* = 8.8 Hz, 2H), 3.91 (s, 3H), 2.48 (s, 3H); ^{13}C NMR (175 MHz, $CDCl_3$): δ 186.7, 165.7, 161.9, 152.2, 134.4, 130.6, 126.8, 125.6, 122.2, 121.2, 116.4, 114.6, 110.4, 55.5, 21.3, two carbons merged with aromatic region; HRMS-ESI+ calcd for $C_{17}H_{14}O_3$ $[M+H]^+$: 267.1021, found 267.1016.

Spectral data for 5-methyl-2-(*p*-tolyl)benzofuran-3-carbaldehyde (2c)

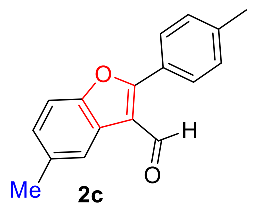

2c

Compound **2c** was purified on silica gel column using ethyl acetate/heane: (5: 95) as the eluent; colorless oil (38.7 mg, 0.154 mmol, 78%); ^{1}H NMR (500 MHz, $CDCl_3$): δ 10.32 (s, 1H), 8.07 (s, 1H), 7.75 (d, *J* = 8.2 Hz, 2H), 7.43 (d, *J* = 8.4 Hz, 1H), 7.37 (d, *J* = 7.9 Hz, 2H), 7.20 (d, *J* = 10.0 Hz, 1H), 2.48 (s, 3H), 2.46 (s, 3H); ^{13}C NMR (125 MHz, $CDCl_3$): δ 186.8, 165.9, 152.3, 141.6, 134.5, 129.8, 129.0, 127.0, 125.9, 125.5, 122.3, 117.0, 110.5, 21.5, 21.3, two carbons merged with aromatic region; HRMS-ESI+ calcd for $C_{17}H_{14}O_2$ $[M+H]^+$: 251.1072, found: 251.1064.

Spectral data for 2-(4-chlorophenyl)-5-methylbenzofuran-3-carbaldehyde (2d)

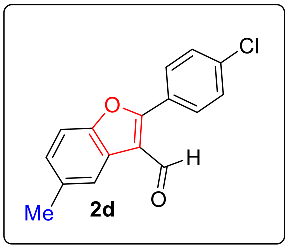

Compound **2d** was purified on silica gel column using ethyl acetate/hexane: (5: 95) as the eluent; brown oil (35.3 mg, 0.130 mmol, 71%); ^{1}H NMR (700 MHz, $CDCl_3$): δ 10.32 (s, 1H), 8.06 (s, 1H), 7.81 (d, *J* = 8.5 Hz, 2H), 7.55 (d, *J* = 8.3 Hz, 2H), 7.44 (d, *J* = 8.4 Hz, 1H), 7.23 (d, *J* = 8.4 Hz, 1H), 2.49 (s, 3H); ^{13}C NMR (175 MHz, $CDCl_3$): δ 186.2, 163.8, 152.4, 137.4, 134.8, 130.1, 129.4, 127.4, 127.1, 125.3, 122.4, 117.5, 110.6, 21.4, two carbons merged with aromatic region; HRMS-ESI+ calcd for $C_{16}H_{11}O_2Cl$ $[M+H]^+$: 271.0525, found: 271.0520.

Spectral data for 5-methyl-2-(*m*-tolyl)benzofuran-3-carbaldehyde (2e)

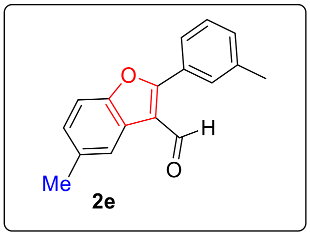

Compound **2e** was purified on silica gel column using ethyl acetate/hexane: (10: 90) as the eluent; colorless oil (36.8 mg, 0.147 mmol, 74%); ^{1}H NMR (500 MHz, $CDCl_3$): δ 10.32 (s, 1H), 8.08 (s, 1H), 7.66 – 7.63 (m, 2H), 7.46 – 7.43 (m, 2H), 7.38 (d, *J* = 7.6 Hz, 1H), 7.21 (d, *J* = 9.0 Hz, 1H), 2.49 (s, 3H), 2.47 (s, 3H); ^{13}C NMR (125 MHz, $CDCl_3$): δ 186.9, 165.9, 152.4, 139.0,

134.6, 131.8, 129.6, 128.9, 128.6, 127.1, 126.3, 125.4, 122.4, 117.3, 110.5, 21.4, 21.3; HRMS-ESI+ calcd for $C_{17}H_{14}O_2$ $[M+H]^+$: 251.1072, found: 251.1076.

Spectral data for 2-(3-chlorophenyl)-5-methylbenzofuran-3-carbaldehyde (2f)

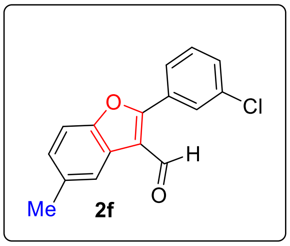

Compound **2f** was purified on silica gel column using ethyl acetate/hexane: (8: 92) as the eluent; pale brown oil (34.3 mg, 0.126 mmol, 69%); ^{1}H NMR (700 MHz, $CDCl_3$): δ 10.34 (s, 1H), 8.07 (s, 1H), 7.86 (s, 1H), 7.74 (d, J = 7.5 Hz, 1H), 7.54 – 7.49 (m, 2H), 7.45 (d, J = 8.4 Hz, 1H), 7.24 (d, J = 8.2 Hz, 1H), 2.49 (s, 3H); ^{13}C NMR (175 MHz, $CDCl_3$): δ 186.2, 163.3, 152.5, 135.2, 134.8, 130.9, 130.3, 128.8, 127.6, 127.1, 125.2, 122.4, 117.8, 110.7, 21.4, one carbon merged with aromatic region; HRMS-ESI+ calcd for $C_{16}H_{11}O_2Cl$ $[M+H]^+$: 271.0525, found: 271.0514.

Spectral data for 2-(2-methoxyphenyl)-5-methylbenzofuran-3-carbaldehyde (2g)

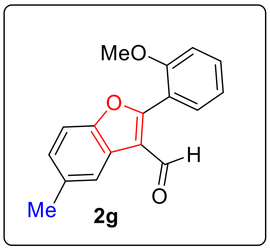

Compound **2g** was purified on silica gel column using ethyl acetate/hexane: (10: 90) as the eluent; yellow oil (34.2 mg, 0.128 mmol, 69%); ^{1}H NMR (700 MHz, $CDCl_3$): δ 10.08 (s, 1H), 8.06 (s, 1H), 7.62 (d, J = 7.5 Hz, 1H), 7.52 (t, J = 7.8 Hz, 1H), 7.42 (d, J = 7.8 Hz, 1H), 7.19 (d, J = 8.3 Hz, 1H), 7.12 (t, J = 7.5 Hz, 1H), 7.08 (d, J = 8.4 Hz, 1H), 3.86 (s, 3H), 2.48 (s, 3H); ^{13}C NMR (175 MHz, $CDCl_3$): δ 187.8, 162.8, 157.3, 153.0, 134.3, 132.4, 131.7, 126.7, 125.1, 122.2, 120.8, 118.0, 117.8, 111.6, 110.5, 55.7, 21.3; HRMS-ESI+ calcd for $C_{17}H_{14}O_3$ $[M+H]^+$: 267.1021, found: 267.1008.

Spectral data for 5-methyl-2-(thiophen-2-yl)benzofuran-3-carbaldehyde (2h)

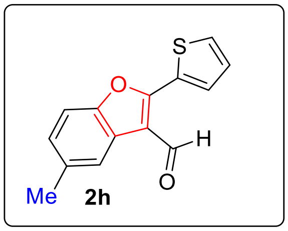

Compound **2h** was purified on silica gel column using ethyl acetate/hexane: (5: 95) as the eluent; brown oil (36 mg, 0.148 mmol, 72%); ^{1}H NMR (700 MHz, $CDCl_3$): δ 10.53 (s, 1H), 8.01 (s, 1H), 7.82 (d, *J* = 3.7 Hz, 1H), 7.62 (d, *J* = 4.9 Hz, 1H), 7.40 (d, *J* = 8.4 Hz, 1H), 7.23 (t, *J* = 4.4 Hz, 1H), 7.20 (d, *J* = 8.3 Hz, 1H), 2.48 (s, 3H); ^{13}C NMR (175 MHz, $CDCl_3$): δ 185.4, 158.9, 152.1, 134.7, 130.4, 130.3, 130.0, 128.3, 127.3, 125.5, 122.0, 116.1, 110.4, 21.3; HRMS-ESI+ calcd for $C_{14}H_{10}O_2S$ $[M+H]^+$: 243.0479, found: 243.0480.

Spectral data for 5-methyl-2-(naphthalen-1-yl)benzofuran-3-carbaldehyde (2i)

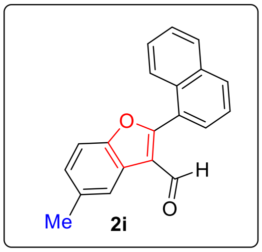

Compound **2i** was purified on silica gel column using ethyl acetate/hexane: (10: 90) as the eluent; colorless oil (37.5 mg, 0.130 mmol, 75%); ^{1}H NMR (700 MHz, $CDCl_3$): δ 9.99 (s, 1H), 8.14 (s, 1H), 8.08 (dd, *J* = 19.2, 7.8 Hz, 2H), 7.97 (d, *J* = 7.8 Hz, 1H), 7.75 (d, *J* = 7.0 Hz, 1H), 7.63 – 7.56 (m, 3H), 7.50 (d, *J* = 8.3 Hz, 1H), 7.27 (s, 1H), 2.53 (s, 3H); ^{13}C NMR (175 MHz, $CDCl_3$): δ 187.1, 166.5, 153.0, 134.7, 133.7, 131.8, 131.6, 130.6, 128.6, 127.6, 127.2, 126.8, 125.5, 125.3, 124.9, 124.7, 122.3, 119.5, 110.8, 21.4; HRMS-ESI+ calcd for $C_{20}H_{14}O_2$ $[M+H]^+$: 287.1072, found: 287.1062.

Spectral data for 2-butyl-5-methylbenzofuran-3-carbaldehyde (2j)

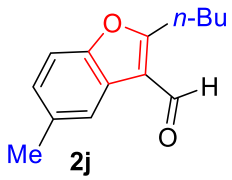

2j

Compound **2j** was purified on silica gel column using ethyl acetate/hexane: (5: 95) as the eluent; colorless oil (39.8 mg, 0.184 mmol, 80%); ^{1}H NMR (700 MHz, $CDCl_3$): δ 10.20 (s, 1H), 7.93 (s, 1H), 7.33 (d, *J* = 7.7 Hz, 1H), 7.13 (d, *J* = 8.4 Hz, 1H), 3.08 (t, *J* = 7.7 Hz, 2H), 2.45 (s, 3H) 1.84 – 1.79 (m, 2H), 1.45 – 1.41 (m, 2H), 0.96 (t, *J* = 7.0 Hz, 3H); ^{13}C NMR (175 MHz, $CDCl_3$): δ 185.0, 170.9, 152.3, 134.2, 126.2, 124.6, 121.6, 117.4, 110.3, 30.3, 26.8, 22.2, 21.3, 13.6; HRMS-ESI+ calcd for $C_{14}H_{16}O_2$ $[M+H]^+$: 217.1228, found: 217.1233.

Spectral data for 2-cyclopropyl-5-methylbenzofuran-3-carbaldehyde (2k)

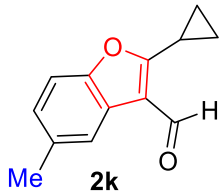

2k

Compound **2k** was purified on silica gel column using ethyl acetate/hexane: (5: 95) as the eluent; white solid (38.7 mg, 0.193 mmol, 78%); ^{1}H NMR (700 MHz, $CDCl_3$): δ 10.31 (s, 1H), 7.88 (s, 1H), 7.23 (d, *J* = 8.4 Hz, 1H), 7.08 (d, *J* = 8.0 Hz, 1H), 2.57 – 2.54 (m, 1H), 2.43 (s, 3H), 1.36 – 1.35 (m, 2H), 1.25 – 1.23 (m, 2H); ^{13}C NMR (175 MHz, $CDCl_3$): δ 184.6, 171.2, 151.4, 134.1, 125.8, 125.1, 121.1, 117.4, 110.0, 21.2, 9.7, 8.7, one carbon merged; HRMS-ESI+ calcd for $C_{13}H_{12}O_2$ $[M+H]^+$: 201.0915, found: 201.0919.

Spectral data for 2-cyclohexyl-5-methylbenzofuran-3-carbaldehyde (2l)

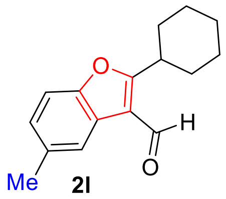

2l

Compound **2l** was purified on silica gel column using ethyl acetate/hexane: (8: 92) as the eluent; colorless oil (37.8 mg, 0.155 mmol, 76%); ^{1}H NMR (700 MHz, $CDCl_3$): δ 10.25 (s, 1H), 7.94 (s, 1H), 7.33 (d, *J* = 8.4 Hz, 1H), 7.13 (d, *J* = 7.3 Hz, 1H), 3.31 – 3.27 (m, 1H), 2.45 (s, 3H), 1.96 – 1.90 (m, 4H), 1.83 – 1.78 (m, 3H), 1.47 – 1.41 (m, 2H), 1.38 – 1.34 (m, 1H); ^{13}C NMR (175 MHz, $CDCl_3$): δ 184.8, 174.5, 152.2, 134.1, 126.1, 124.7, 121.7, 115.8, 110.3, 37.0, 31.4, 26.0, 25.5, 21.3, two carbons merged; HRMS-ESI+ calcd for $C_{16}H_{18}O_2$ $[M+H]^+$: 243.1385, found: 243.1383.

Spectral data for 2,5-diphenylbenzofuran-3-carbaldehyde (2m)

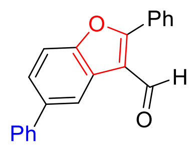

2m

Compound **2m** was purified on silica gel column using ethyl acetate/hexane: (8: 92) as the eluent; white solid (39.8 mg, 0.133 mmol, 80%); ^{1}H NMR (700 MHz, $CDCl_3$): δ 10.38 (s, 1H), 8.50 (s, 1H), 7.89 – 7.88 (m, 2H), 7.68 – 7.61 (m, 4H), 7.60 – 7.59 (m, 3H), 7.47 (t, *J* = 7.5 Hz, 2H), 7.37 (t, *J* = 7.3 Hz, 1H); ^{13}C NMR (175 MHz, $CDCl_3$): δ 186.6, 165.9, 153.6, 141.0, 138.5, 131.2, 129.18, 129.15, 128.7, 128.5, 127.5, 127.2, 126.0, 125.6, 121.1, 117.6, 111.2, four carbons merged with aromatic region; HRMS-ESI+ calcd for $C_{21}H_{14}O_2$ $[M+H]^+$: 299.1072, found: 299.1068.

Spectral data for 5-butyl-2-phenylbenzofuran-3-carbaldehyde (2n)

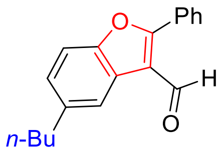

2n

Compound **2n** was purified on silica gel column using ethyl acetate/hexane: (8: 92) as the eluent; colorless oil (37.7 mg, 0.135 mmol, 76%); ^{1}H NMR (700 MHz, $CDCl_3$): δ 10.33 (s, 1H), 8.08 (s, 1H), 7.85 (dd, *J* = 4.0, 3.3 Hz, 2H), 7.57 – 7.56 (m, 3H), 7.46 (d, *J* = 8.4 Hz, 1H), 7.23 (d, *J* = 8.4 Hz, 1H), 2.75 (t, *J* = 7.8 Hz, 2H), 1.67 (p, *J* = 7.7 Hz, 2H), 1.41 – 1.37 (m, 2H), 0.94 (t, *J* = 7.3

Hz, 3H); ^{13}C NMR (175 MHz, $CDCl_3$): δ 186.8, 165.6, 152.6, 139.9, 130.9, 129.1, 128.7, 126.7, 125.3, 121.8, 117.4, 110.6, 35.6, 34.2, 22.3, 13.9, three carbons merged; HRMS-ESI+ calcd for $C_{19}H_{18}O_2$ $[M+H]^+$: 279.1385, found: 279.1397.

Spectral data for 5-ethyl-2-phenylbenzofuran-3-carbaldehyde (2o)

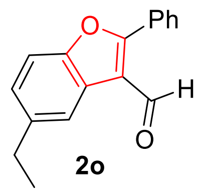

2o

Compound **2o** was purified on silica gel column using ethyl acetate/hexane: (8: 92) as the eluent; brown oil (36.8 mg, 0.147 mmol, 74%); ^{1}H NMR (700 MHz, $CDCl_3$): δ 10.34 (s, 1H), 8.11 (s, 1H), 7.85 (ddd, J = 4.9, 2.4, 1.5 Hz, 2H), 7.57 – 7.56 (m, 3H), 7.47 (d, J = 8.4 Hz, 1H), 7.25 (d, J = 9.7 Hz, 1H), 2.79 (q, J = 7.6 Hz, 2H), 1.30 (t, J = 7.7 Hz, 3H); ^{13}C NMR (175 MHz, $CDCl_3$): δ 186.8, 165.6, 152.6, 141.2, 131.0, 129.1, 128.7, 126.2, 125.4, 121.3, 117.4, 110.7, 28.9, 16.3, three carbons merged with aromatic region; HRMS-ESI+ calcd for $C_{17}H_{14}O_2$ $[M+H]^+$: 251.1072, found: 251.1065.

Spectral data for 5-isopropyl-2-phenylbenzofuran-3-carbaldehyde (2p)

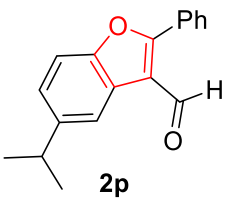

2p

Compound **2p** was purified on silica gel column using ethyl acetate/hexane: (8: 92) as the eluent; colorless oil (35.8 mg, 0.135 mmol, 72%); ^{1}H NMR (700 MHz, $CDCl_3$): δ 10.34 (s, 1H), 8.14 (d, J = 1.9 Hz, 1H), 7.85 (dd, J = 6.6, 3.1 Hz, 2H), 7.57 – 7.56 (m, 3H), 7.48 (d, J = 8.5 Hz, 1H), 7.29 (dd, J = 8.5, 1.8 Hz, 1H), 3.07 (p, J = 6.9 Hz, 1H), 1.33 (d, J = 6.9 Hz, 6H); ^{13}C NMR (175 MHz, $CDCl_3$): δ 186.8, 165.6, 152.6, 146.0, 131.0, 129.1, 128.7, 125.3, 124.8, 119.9, 117.5, 110.7, 34.3, 24.4, four carbons merged; HRMS-ESI+ calcd for $C_{18}H_{16}O_2$ $[M+H]^+$: 265.1228, found: 265.1230.

Spectral data for 2-(4-acetylphenyl)-5-methylbenzofuran-3-carbaldehyde (2q)

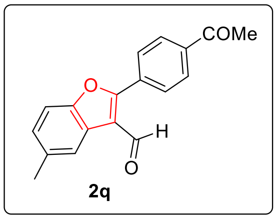

Compound **2q** was purified on silica gel column using ethyl acetate/hexane: (10: 90) as the eluent; brown oil (30.0 mg, 0.107 mmol, 60%); ^{1}H NMR (400 MHz, $CDCl_3$): δ 10.38 (s, 1H), 8.14 – 8.12 (m, 2H), 8.09 (s, 1H), 7.99 – 7.96 (m, 2H), 7.47 (d, *J* = 8.4 Hz, 1H), 7.24 – 7.23 (m, 1H), 2.68 (s, 3H), 2.50 (s, 3H); ^{13}C NMR (100 MHz, $CDCl_3$) δ 197.1, 186.2, 163.2, 152.7, 138.4, 135.0, 132.8, 129.1, 128.9, 127.8, 125.3, 122.5, 118.4, 110.7, 26.7, 21.4, two carbons merged; HRMS-ESI+ calcd for $C_{18}H_{14}O_3$ $[M+H]^+$: 279.1021, found: 279.1020.

Spectral data for 2-(4-formylphenyl)-5-methylbenzofuran-3-carbaldehyde (2r)

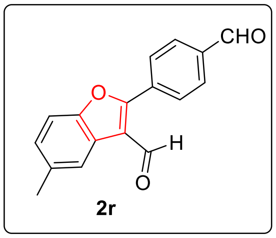

Compound **2r** was purified on silica gel column using ethyl acetate/hexane: (10: 90) as the eluent; yellow oil (19.0 mg, 0.071 mmol, 38%); ^{1}H NMR (400 MHz, $CDCl_3$): δ 10.40 (s, 1H), 10.13 (s, 1H), 8.09 – 8.04 (m, 5H), 7.48 (d, *J* = 8.5 Hz, 1H), 7.28 – 7.27 (m, 1H), 2.50 (s, 3H); ^{13}C NMR (175 MHz, $CDCl_3$) δ 191.2, 186.0, 162.7, 152.8, 137.4, 135.0, 134.0, 130.1, 129.4, 128.0, 125.3, 122.5, 118.6, 110.8, 21.4, two carbons merged with aromatic region; HRMS-ESI+ calcd for $C_{17}H_{12}O_3$ $[M+H]^+$: 265.0864, found: 265.0867.

Spectral data for 4-(3-formyl-5-methylbenzofuran-2-yl)benzonitrile

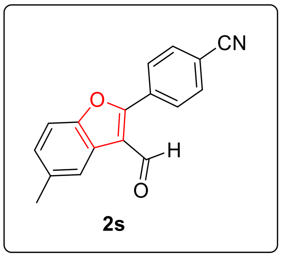

Compound **2s** was purified on silica gel column using ethyl acetate/hexane: (10: 90) as the eluent; colorless oil (24.0 mg, 0.091 mmol, 48%); ^{1}H NMR (700 MHz, $CDCl_3$): δ 10.38 (s, 1H), 8.08 (s, 1H), 8.02 (d, *J* = 8.3 Hz, 2H), 7.86 (d, *J* = 7.9 Hz, 2H), 7.47 (d, *J* = 8.4 Hz, 1H), 7.27 (s, 1H), 2.50 (s, 3H); ^{13}C NMR (175 MHz, $CDCl_3$): δ 185.6, 161.5, 152.7, 135.2, 132.7, 129.3, 128.2, 125.2, 122.5, 118.6, 117.9, 114.3, 110.8, 21.4, three carbons merged with aromatic region; HRMS-ESI+ calcd for $C_{17}H_{11}NO_2$ $[M+H]^+$:262.0868, found: 262.0869.

Spectral data for (3R,3aR,7aR)-3-benzoyl-7a-methyl-2,3,3a,7a-tetrahydrobenzofuran-5(4*H*)-one (**3a**)

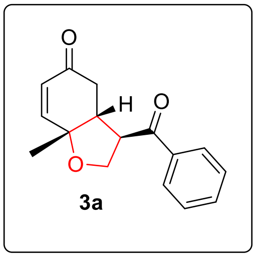

Compound **3a** was purified on silica gel column using ethyl acetate/hexane: (15: 85) as the eluent; white solid (38.2 mg, 0.149 mmol, 71%); ^{1}H NMR (700 MHz, $CDCl_3$): δ 7.82 (d, *J* = 7.2 Hz, 2H), 7.57 (t, *J* = 7.4 Hz, 1H), 7.46 – 7.44 (m, 2H), 6.67 (dd, *J* = 10.3, 2.0 Hz, 1H), 6.02 (dd, *J* = 10.3, 1.2 Hz, 1H), 4.14 (t, *J* = 9.2 Hz, 1H), 3.89 – 3.84 (m, 1H), 3.83 – 3.80 (m, 1H), 3.11 – 3.08 (m, 1H), 2.66 (dd, *J* = 17.4, 5.5 Hz, 1H), 2.57 – 2.54 (m, 1H), 1.53 (s, 3H); ^{13}C NMR (175 MHz, $CDCl_3$): δ 197.8, 197.1, 152.8, 136.1, 133.7, 128.9, 128.8, 128.4, 80.5, 68.9, 51.7, 45.7, 37.5, 23.3, two carbons merged with aromatic region; HRMS-ESI+ calcd for $C_{16}H_{16}O_3$ $[M+H]^+$: 257.1177, found: 257.1176.

Spectral data for (3R,3aR,7aR)-7a-methyl-3-(4-methylbenzoyl)-2,3,3a,7a-tetrahydrobenzofuran-5(4*H*)-one (3b)

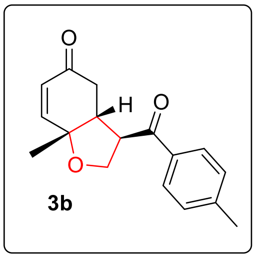

Compound **3b** was purified on silica gel column using ethyl acetate/hexane: (8: 92) as the eluent; colorless oil (41.9 mg, 0.155 mmol, 78%); ^{1}H NMR (700 MHz, $CDCl_3$): δ 7.73 (d, *J* = 7.2 Hz, 2H), 7.26 (d, *J* = 9.9 Hz, 2H), 6.67 (d, *J* = 10.3 Hz, 1H), 6.03 (d, *J* = 10.3 Hz, 1H), 4.14 (t, *J* = 9.2 Hz, 1H), 3.89 – 3.87 (m, 1H), 3.81 (q, *J* = 8.9 Hz, 1H), 3.10 – 3.08 (m, 1H), 2.67 – 2.64 (m, 1H), 2.57 (d, *J* = 16.5 Hz, 1H), 2.40 (s, 3H), 1.54 (s, 3H); ^{13}C NMR (175 MHz, $CDCl_3$): δ 197.4, 197.2, 152.9, 144.7, 133.7, 129.6, 128.9, 128.5, 80.5, 69.0, 51.5, 45.7, 37.5, 23.4, 21.6, two carbons merged; HRMS-ESI+ calcd for $C_{17}H_{18}O_3$ $[M+H]^+$: 271.1334, found: 271.1337.

Spectral data for (3R,3aR,7aR)-3-(4-chlorobenzoyl)-7a-methyl-2,3,3a,7a-tetrahydrobenzofuran-5(4*H*)-one (3c)

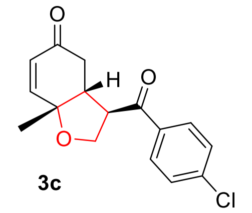

3c

Compound **3c** was purified on silica gel column using ethyl acetate/hexane: (8: 92) as the eluent; brown oil (36.8 mg, 0.126 mmol, 69%); ^{1}H NMR (700 MHz, $CDCl_3$); δ 7.78 (d, *J* = 7.8 Hz, 2H), 7.46 (d, *J* = 8.2 Hz, 2H), 6.68 (d, *J* = 10.3 Hz, 1H), 6.04 (d, *J* = 10.3 Hz, 1H), 4.15 – 4.12 (m, 1H), 3.88 (t, *J* = 7.8 Hz, 1H), 3.78 (q, *J* = 9.1 Hz, 1H), 3.11 – 3.09 (m, 1H), 2.69 (dd, *J* = 17.1, 4.6 Hz, 1H), 2.57 (d, *J* = 17.1 Hz, 1H), 1.55 (s, 3H); ^{13}C NMR (175 MHz, $CDCl_3$): δ 197.0, 196.6, 152.7, 140.3, 134.4, 129.8, 129.2, 129.0, 80.6, 68.8, 51.7, 45.7, 37.5, 23.3, two carbons merged; HRMS-ESI+ calcd for $C_{16}H_{15}O_3Cl$ $[M+H]^+$: 291.0788, found: 291.0790.

Spectral data for (3R,3aR,7aR)-7a-methyl-3-(3-methylbenzoyl)-2,3,3a,7a-tetrahydrobenzofuran-5(4*H*)-one (3d)

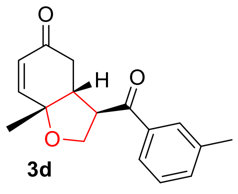

3d

Compound **3d** was purified on silica gel column using ethyl acetate/hexane: (8: 92) as the eluent; brown oil (37.8 mg, 0.139 mmol, 70%); ^{1}H NMR (700 MHz, $CDCl_3$): δ 7.64 (s, 1H), 7.61 (d, *J* =

7.6 Hz, 1H), 7.40 (d, J = 7.8 Hz, 1H), 7.35 (t, J = 7.6 Hz, 1H), 6.68 (dd, J = 10.8, 1.4 Hz, 1H), 6.04 (d, J = 10.3 Hz, 1H), 4.15 (t, J = 9.8 Hz, 1H), 3.90 – 3.81 (m, 2H), 3.12 – 3.10 (m, 1H), 2.68 (dd, J = 17.4, 5.5 Hz, 1H), 2.58 (d, J = 17.5 Hz, 1H), 2.41 (s, 3H), 1.55 (s, 3H); ^{13}C NMR (175 MHz, $CDCl_3$): δ 198.0, 197.2, 152.9, 138.8, 136.2, 134.5, 128.95, 128.94, 128.7, 125.6, 80.5, 69.0, 51.7, 45.7, 37.5, 23.4, 21.3; HRMS-ESI+ calcd for $C_{17}H_{18}O_3$ $[M+H]^+$: 271.1334, found: 271.1338.

Spectral data for (3R,3aR,7aR)-3-(3-chlorobenzoyl)-7a-methyl-2,3,3a,7a-tetrahydrobenzofuran-5(4*H*)-one (3e)

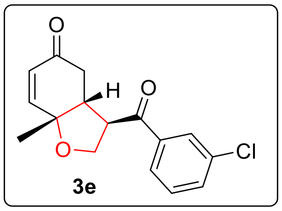

Compound **3e** was purified on silica gel column using ethyl acetate/hexane: (8: 92) as the eluent; brown oil (35.0 mg, 0.120 mmol, 65%); ^{1}H NMR (700 MHz, $CDCl_3$): δ 7.82 (s, 1H), 7.68 (d, J = 7.7 Hz, 1H), 7.57 (d, J = 7.7 Hz, 1H), 7.42 (t, J = 7.9 Hz, 1H), 6.68 (dd, J = 2.1, 1.4 Hz, 1H), 6.05 (d, J = 10.5 Hz, 1H), 4.15 (t, J = 9.1 Hz, 1H), 3.88 – 3.85 (m, 1H), 3.78 – 3.74 (m, 1H), 3.13 – 3.11 (m, 1H), 2.69 (dd, J = 11.9, 5.5 Hz, 1H), 2.57 (d, J = 18.2 Hz, 1H), 1.56 (s, 3H); ^{13}C NMR (175 MHz, $CDCl_3$): δ 196.9, 196.6, 152.7, 137.7, 135.4, 133.7, 130.2, 129.1, 128.5, 126.4, 80.6, 68.7, 52.0, 45.6, 37.5, 23.3; HRMS-ESI+ calcd for $C_{16}H_{15}O_3Cl$ $[M+H]^+$: 291.0788, found: 291.0785.

Spectral data for (3R,3aR,7aR)-3-(2-methoxybenzoyl)-7a-methyl-2,3,3a,7a-tetrahydrobenzofuran-5(4*H*)-one (3f)

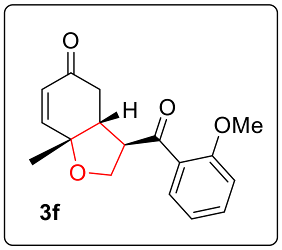

Compound **3f** was purified on silica gel column using ethyl acetate/hexane: (8: 92) as the eluent; yellow oil (37.5 mg, 0.130 mmol, 70%); ^{1}H NMR (700 MHz, $CDCl_3$): δ 7.60 (d, J = 7.6 Hz, 1H),

7.48 – 7.46 (m, 1H), 7.00 (t, J = 7.4 Hz, 1H), 6.94 (d, J = 8.4 Hz, 1H), 6.64 (d, J = 10.3 Hz, 1H), 6.01 (d, J = 10.3 Hz, 1H), 4.01 – 3.96 (m, 3H), 3.83 (s, 3H), 3.00 – 2.98 (m, 1H), 2.59 – 2.58 (m, 2H), 1.52 (s, 3H); ^{13}C NMR (175 MHz, $CDCl_3$): δ 201.4, 197.3, 158.2, 153.0, 134.0, 130.4, 129.0, 127.8, 120.9, 111.4, 80.4, 69.1, 55.9, 55.4, 46.7, 37.5, 23.3; HRMS-ESI+ calcd for $C_{17}H_{18}O_4$ $[M+H]^+$: 287.1283, found: 287.1289.

Spectral data for (3S,3aR,7aR)-3-((E)-(2-methoxyphenyl)(phenylimino)methyl)-7a-methyl-2,3,3a,7a-tetrahydrobenzofuran-5(4*H*)-one (3f')

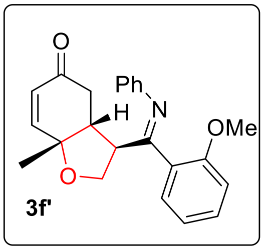

Compound **3f'** was purified on silica gel column using ethyl acetate/hexane: (8: 92) as the eluent; yellow oil (42.5 mg, 0.117 mmol, 63%); ^{1}H NMR (700 MHz, $CDCl_3$): δ 7.16 (t, J = 7.8 Hz, 1H), 7.07 (t, J = 7.9 Hz, 2H), 6.87 (t, J = 7.2 Hz, 1H), 6.77 (d, J = 8.3 Hz, 1H), 6.72 (t, J = 7.4 Hz, 1H), 6.67 (d, J = 7.3 Hz, 1H), 6.64 – 6.61 (m, 3H), 5.93 (d, J = 10.1 Hz, 1H), 4.13 – 4.10 (m, 1H), 4.04 – 4.01 (m, 1H), 3.73 (s, 3H), 3.46 (q, J = 8.8 Hz, 1H), 3.13 (s, 1H), 2.72 (d, J = 17.0 Hz, 1H), 2.61 (dd, J = 17.4, 5.5 Hz, 1H), 1.55 (s, 3H); ^{13}C NMR (175 MHz, $CDCl_3$): δ 197.6, 169.2, 155.8, 153.3, 150.4, 129.9, 128.2, 127.96, 127.93, 127.3, 123.3, 120.3, 119.8, 110.5, 80.3, 70.2, 55.1, 53.2, 46.9, 37.0, 23.8, two carbons merged with aromatic region; HRMS-ESI+ calcd for $C_{23}H_{23}NO_3$ $[M+H]^+$: 362.1756, found: 362.1756.

Spectral data for (3R,3aR,7aR)-3-benzoyl-7a-isopropyl-2,3,3a,7a-tetrahydrobenzofuran-5(4*H*)-one (3g)

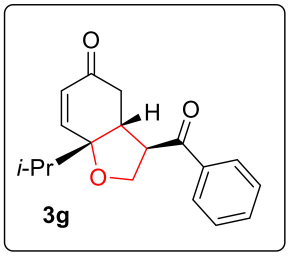

Compound **3g** was purified on silica gel column using ethyl acetate/hexane: (8: 92) as the eluent; pale yellow oil (33.1 mg, 0.116 mmol, 62%); ^{1}H NMR (700 MHz, $CDCl_3$): δ 7.86 (d, *J* = 7.2 Hz, 2H), 7.59 (t, *J* = 7.3 Hz, 1H), 7.49 – 7.46 (m, 2H), 6.70 (dd, *J* = 10.4, 1.8 Hz, 1H), 6.16 (d, *J* = 1.1 Hz, 1H), 4.17 (t, *J* = 8.6 Hz, 1H), 3.85 – 3.79 (m, 2H), 3.35 – 3.33 (m, 1H), 2.68 (dd, *J* = 17.8, 6.1 Hz, 1H), 2.53 – 2.50 (m, 1H), 2.12 (q, *J* = 6.9 Hz, 1H), 1.09 (dd, *J* = 16.4, 6.9 Hz, 6H); ^{13}C NMR (175 MHz, $CDCl_3$): δ 197.7, 197.6, 151.3, 136.2, 133.7, 129.9, 128.9, 128.4, 84.5, 69.0, 53.1, 41.1, 38.6, 35.7, 17.8, 17.0, two carbons merged; HRMS-ESI+ calcd for $C_{18}H_{20}O_3$ $[M+H]^+$: 285.1490, found: 285.1492.

Spectral data for (3R,3aR,7aR)-3-benzoyl-7a-butyl-2,3,3a,7a-tetrahydrobenzofuran-5(4*H*)-one (3h)

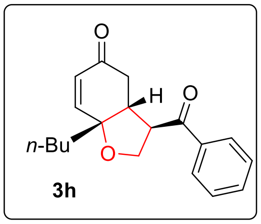

Compound **3h** was purified on silica gel column using ethyl acetate/hexane: (8: 92) as the eluent; colorless oil (37.5 mg, 0.125 mmol, 70%); ^{1}H NMR (700 MHz, $CDCl_3$): δ 7.85 (d, *J* = 8.4 Hz, 2H), 7.59 (t, *J* = 7.0 Hz, 1H), 7.47 (t, *J* = 7.2 Hz, 2H), 6.71 (d, *J* = 10.3 Hz, 1H), 6.08 (d, *J* = 10.5 Hz, 1H), 4.18 – 4.15 (m, 1H), 3.87 – 3.83 (m, 2H), 3.21 – 3.19 (m, 1H), 2.68 (dd, *J* = 17.6, 5.3 Hz, 1H), 2.55 (d, *J* = 19.3 Hz, 1H), 1.90 – 1.86 (m, 1H), 1.79 – 1.75 (m, 1H), 1.51 – 1.48 (m, 2H), 1.42 – 1.36 (m, 2H), 0.93 (t, *J* = 7.4 Hz, 3H); ^{13}C NMR (175 MHz, $CDCl_3$): δ 197.8, 197.4, 152.5, 136.2, 133.7, 129.2, 128.9, 128.4, 82.5, 69.0, 51.9, 43.8, 37.7, 37.2, 25.8, 23.1, 13.9, two carbons merged; HRMS-ESI+ calcd for $C_{19}H_{22}O_3$ $[M+H]^+$: 299.1647, found: 299.1649.

Spectral data for (3R,3aR,7aS)-3-benzoyl-4,6,7a-trimethyl-2,3,3a,7a-tetrahydrobenzofuran-5(4*H*)-one (3i)

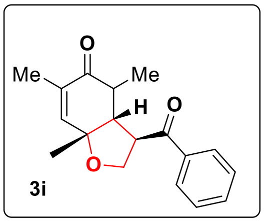

Compound **3i** was purified on silica gel column using ethyl acetate/hexane: (8: 92) as the eluent; colorless oil (38.5 mg, 0.135 mmol, 72%); ^{1}H NMR (700 MHz, CDCl$_3$): δ 7.85 (d, *J* = 7.1 Hz, 2H), 7.58 (t, *J* = 7.3 Hz, 1H), 7.47 (t, *J* = 7.8 Hz, 2H), 6.40 (s, 1H), 4.12 (t, *J* = 8.4 Hz, 1H), 3.81 – 3.76 (m, 2H), 2.98 (d, *J* = 8.2 Hz, 1H), 2.65 – 2.61 (m, 1H), 1.85 (s, 3H), 1.57 (s, 3H), 1.29 (d, *J* = 7.6 Hz, 3H); ^{13}C DEPT NMR (175 MHz, CDCl$_3$): δ 201.6, 198.0, 146.2, 136.2, 133.67, 133.66, 128.8, 128.4, 79.8, 68.4, 54.3, 51.2, 43.6, 26.6, 18.6, 16.0, two carbons merged with aromatic region; HRMS-ESI+ calcd for $C_{18}H_{20}O_3$ [M+H]$^+$: 285.1490, found: 285.1491.

Spectral (3R,3aR,7aS)-3-benzoyl-6,7a-dimethyl-2,3,3a,7a-tetrahydrobenzofuran-5(4*H*)-one (3j)

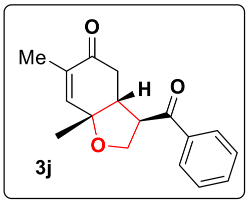

Compound **3j** was purified on silica gel column using ethyl acetate/hexane: (8: 92) as the eluent; colorless oil (40.2 mg, 0.148 mmol, 75%); ^{1}H NMR (700 MHz, CDCl$_3$): δ 7.83 (d, *J* = 7.0 Hz, 2H), 7.58 (t, *J* = 7.4 Hz, 1H), 7.47 (t, *J* = 7.7 Hz, 2H), 5.94 (s, 1H), 4.03 (t, *J* = 9.3 Hz, 1H), 3.89 – 3.81 (m, 2H), 3.08 – 3.05 (m, 1H), 2.67 (dd, *J* = 17.6, 5.5 Hz, 1H), 2.57 – 2.54 (m, 1H), 2.01 (s, 3H), 1.56 (s, 3H); ^{13}C DEPT NMR (175 MHz, CDCl$_3$): δ 198.1, 196.5, 163.5, 136.2, 133.7, 128.8, 128.4, 127.7, 82.6, 68.4, 51.3, 46.6, 37.3, 22.4, 17.8, two carbons merged with aromatic region; HRMS-ESI+ calcd for $C_{17}H_{18}O_3$ [M+H]$^+$: 271.1334, found: 271.1339.

Spectral data for (3R,3aR,7aR)-7a-methyl-3-(thiophene-2-carbonyl)-2,3,3a,7a-tetrahydrobenzofuran-5(4*H*)-one (3k)

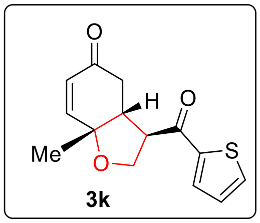

Compound **3k** was purified on silica gel column using ethyl acetate/hexane: (8: 92) as the eluent; brown oil (32.5 mg, 0.123 mmol, 60%); ^{1}H NMR (700 MHz, CDCl$_3$): δ 7.70 (d, *J* = 4.6 Hz, 1H),

7.60 (d, *J* = 3.3 Hz, 1H), 7.15 (t, *J* = 4.1 Hz, 1H), 6.68 (d, *J* = 10.2 Hz, 1H), 6.04 (d, *J* = 10.1 Hz, 1H), 4.11 (t, *J* = 9.1 Hz, 1H), 4.04 (t, *J* = 8.1 Hz, 1H), 3.74 – 3.70 (m, 1H), 3.03 – 3.01 (m, 1H), 2.68 – 2.60 (m, 2H), 1.56 (s, 3H); ^{13}C NMR (175 MHz, $CDCl_3$): δ 196.9, 190.9, 152.8, 143.9, 135.0, 132.6, 128.7, 128.5, 80.7, 69.3, 52.8, 46.8, 37.4, 23.3; HRMS-ESI+ calcd for $C_{14}H_{14}O_3S$ $[M+H]^+$: 263.0741, found: 263.0747.

Spectral data for (3R,3aR,7aR)-3-benzoyl-7a-phenyl-2,3,3a,7a-tetrahydrobenzofuran-5(4*H*)-one (3l)

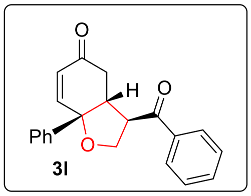

Compound **3l** was purified on silica gel column using ethyl acetate/hexane: (8: 92) as the eluent; pale yellow oil (41.4 mg, 0.130 mmol, 78%); ^{1}H NMR (700 MHz, $CDCl_3$): δ 7.88 (d, *J* = 7.7 Hz, 2H), 7.61 (t, *J* = 6.8 Hz, 1H), 7.51 – 7.48 (m, 4H), 7.41 (t, *J* = 7.0 Hz, 2H), 7.36 – 7.34 (m, 1H), 6.81 (d, *J* = 10.3 Hz, 1H), 6.34 (d, *J* = 10.7 Hz, 1H), 4.40 (t, *J* = 9.2 Hz, 1H), 4.19 (t, *J* = 7.9 Hz, 1H), 4.02 (q, *J* = 8.6 Hz, 1H), 3.31 – 3.29 (m, 1H), 2.70 (dd, *J* = 5.5, 1.6 Hz, 1H), 2.54 (d, *J* = 16.7 Hz, 1H); ^{13}C NMR (175 MHz, $CDCl_3$): δ 197.5, 197.3, 150.2, 139.9, 136.2, 133.8, 130.2, 128.9, 128.8, 128.45, 128.40, 125.2, 84.2, 69.9, 52.0, 48.5, 36.8, four carbons merged with aromatic region; HRMS-ESI+ calcd for $C_{21}H_{18}O_3$ $[M+H]^+$: 319.1334, found: 319.1334.

Spectral data for (5-methyl-2-phenylbenzofuran-3-yl)(phenyl)methanol (4a)

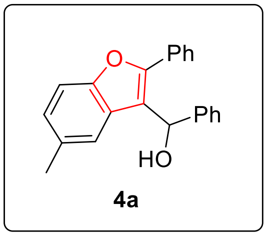

Compound **4a** was purified on silica gel column using ethyl acetate/hexane: (15: 85) as the eluent; colorless oil (45.5 mg, 0.144 mmol, 85%); ^{1}H NMR (700 MHz, DMSO): δ 7.77 (d, *J* = 7.5 Hz, 2H), 7.52 (d, *J* = 8.1 Hz, 2H), 7.45 (d, *J* = 7.2 Hz, 2H), 7.39 (d, *J* = 7.4 Hz, 2H), 7.30 – 7.28 (m, 2H), 7.21 – 7.19 (m, 2H), 7.07 (d, *J* = 8.4 Hz, 1H), 6.15 (s, 1H), 6.13 (broad s, 1H), 2.25

(s, 3H); ^{13}C NMR (175 MHz, DMSO): δ 152.5, 151.6, 143.8, 131.7, 130.5, 129.4, 128.6, 128.5, 127.8, 127.3, 126.3, 126.1, 122.1, 119.4, 111.0, 67.0, 21.4, five carbons merged with aromatic region; HRMS-ESI- calcd for $C_{22}H_{18}O_2$ [M-H]$^-$: 313.1228, found: 313.1229.

Spectral data for (5-methyl-2-phenylbenzofuran-3-yl)methanol (4b)

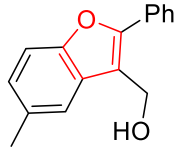

4b

Compound **4b** was purified on silica gel column using ethyl acetate/hexane: (12: 88) as the eluent; colorless oil (36.4 mg, 0.152 mmol, 90%); ^{1}H NMR (700 MHz, DMSO): δ 7.83 (d, J = 7.6 Hz, 2H), 7.53 (dd, J = 14.4, 6.8 Hz, 3H), 7.46 – 7.41 (m, 2H), 7.13 (d, J = 7.6 Hz, 1H), 5.27 (broad s, 1H), 4.71 (s, 2H), 2.40 (s, 3H); ^{13}C NMR (175 MHz, DMSO): δ 152.3, 152.1, 132.1, 130.4, 129.9, 129.3, 129.2, 127.3, 126.2, 120.5, 116.5, 110.9, 53.8, 21.4, two carbons merged with aromatic region; HRMS-ESI- calcd for $C_{16}H_{14}O_2$ [M-H]$^-$: 237.0915, found: 237.0910.

Spectral data for (3R,3aR,7aS)-3-benzoyl-7a-methyl-7-(*p*-tolylthio)hexahydrobenzofuran-5(4*H*)-one (5a)

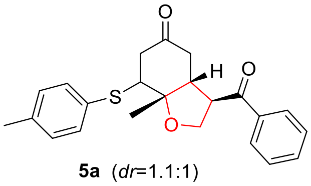

5a (*dr*=1.1:1)

Compound **5a** was purified on silica gel column using ethyl acetate/hexane: (10: 90) as the eluent; white solid (47.6 mg, 0.125 mmol, 80%); ^{1}H NMR for major isomer (400 MHz, $CDCl_3$): δ 7.91 – 7.86 (m, 4H), 7.62 – 7.58 (m, 2H), 7.51 – 7.46 (m, 4H), 7.37 (s, 2H), 7.15 – 7.12 (m, 4H), 4.33 – 4.26 (m, 2H), 3.89 (t, J = 8.9 Hz, 1H), 3.81 – 3.76 (m, 2H), 3.70 – 3.63 (m, 1H), 3.31– 3.27 (m, 1H), 3.20 – 3.15 (m, 1H), 2.84 – 2.74 (m, 2H), 2.56 – 2.45 (m, 2H), 2.33 (s, 3H), 1.72 (s, 3H); ^{1}H NMR for minor isomer (400 MHz, $CDCl_3$): δ 7.38 (s, 2H), 3.59 (dd, J = 9.9, 4.6 Hz, 1H), 3.05 – 3.0 (m, 1H), 2.71 – 2.61 (m, 2H), 2.40 – 2.35 (m, 2H), 2.34 (s, 3H), 1.61 (s, 3H),

rest of peaks merged with major isomer; ^{13}C NMR for major isomer (100 MHz, CDCl3): δ 209.6, 197.5, 138.3, 136.5, 133.8, 133.4, 130.7, 130.0, 128.9, 128.4, 84.0, 68.9, 54.5, 53.1, 47.18, 42.7, 41.1, 25.4, 21.1; ^{13}C NMR for minor isomer (100 MHz, $CDCl_3$): δ 208.3, 197.3, 138.1, 136.4, 133.9, 130.2, 83.2, 68.5, 54.0, 52.6, 47.13, 43.1, 41.6, 22.1, rest of carbons merged with major isomer; HRMS-ESI+ calcd for $C_{23}H_{24}O_3S$ $[M+H]^+$: 381.1524, found: 381.1527.

Spectral data for (3R,3aR,7aR)-3-benzoyl-7a-methylhexahydrobenzofuran-5(4*H*)-one

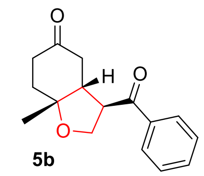

5b

Compound **5b** was purified on silica gel column using ethyl acetate/hexane: (10: 90) as the eluent; colorless oil (30.7 mg, 0.118 mmol, 76%); ^{1}H NMR (400 MHz, $CDCl_3$): δ 7.92 (dd, *J* = 8.5, 1.3 Hz, 2H), 7.62 – 7.58 (m, 1H), 7.51 – 7.47 (m, 2H), 4.26 – 4.22 (m, 1H), 3.80 – 3.68 (m, 2H), 3.01 – 2.97 (m, 1H), 2.61 – 2.46 (m, 2H), 2.35 – 2.28 (m, 2H), 2.13 – 2.07 (m, 1H), 1.97 – 1.91 (m, 1H), 1.45 (s, 3H); ^{13}C NMR (100 MHz, $CDCl_3$): δ 211.9, 197.9, 136.6, 137.7, 128.9, 128.4, 81.5, 68.2, 54.6, 46.2, 41.7, 35.1, 33.0, 26.3, two carbons merged with aromatic region; HRMS-ESI+ calcd for $C_{16}H_{18}O_3$ $[M+H]^+$: 259.1334, found: 259.1333.

Spectral data for (1aS,3aR,4R,6aS,6bR)-4-benzoyl-6a-methylhexahydrooxireno[2,3-g]benzofuran-2(1a*H*)-one (5c)

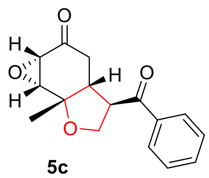

5c

Compound **5c** was purified on silica gel column using ethyl acetate/hexane: (15: 85) as the eluent; colorless oil (33.3 mg, 0.122 mmol, 78%); ^{1}H NMR (700 MHz, $CDCl_3$): δ 7.89 (d, *J* = 8.0 Hz, 2H), 7.60 (t, *J* = 7.4 Hz, 1H), 7.49 (t, *J* = 7.7 Hz, 2H), 4.21 – 4.19 (m, 1H), 3.85 (t, *J* = 8.7 Hz, 1H), 3.74 – 3.70 (m, 1H), 3.45 (dd, *J* = 4.0, 1.7 Hz, 1H), 3.36 (d, *J* = 3.8 Hz, 1H), 3.05 – 3.0 (m, 2H), 2.07 – 2.05 (m, 1H), 1.65 (s, 3H); ^{13}C NMR (175 MHz, $CDCl_3$): δ 206.5, 196.9, 136.2,

133.9, 128.9, 128.4, 78.7, 69.7, 64.5, 55.6, 52.0, 50.3, 34.2, 23.6, two carbons merged with aromatic region; HRMS-ESI+ calcd for $C_{16}H_{16}O_4$ $[M+H]^+$: 273.1126, found: 273.1127.

8. X-ray crystallographic structure and data for compound (2a, 2k, 3a, and 5a):

(a) X-ray crystallographic data of compound (2a):

Ellipsoid contour %probability level = 50%

Experimental: The sample was dissolved in appropriate amount of Dichloromethane followed by the addition of pentane to furnish a saturated solution. Afterwards, the mixture was allowed to stand at room temperature to form the crystals.

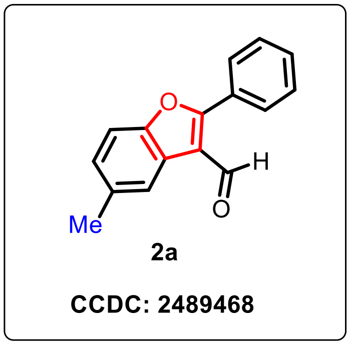

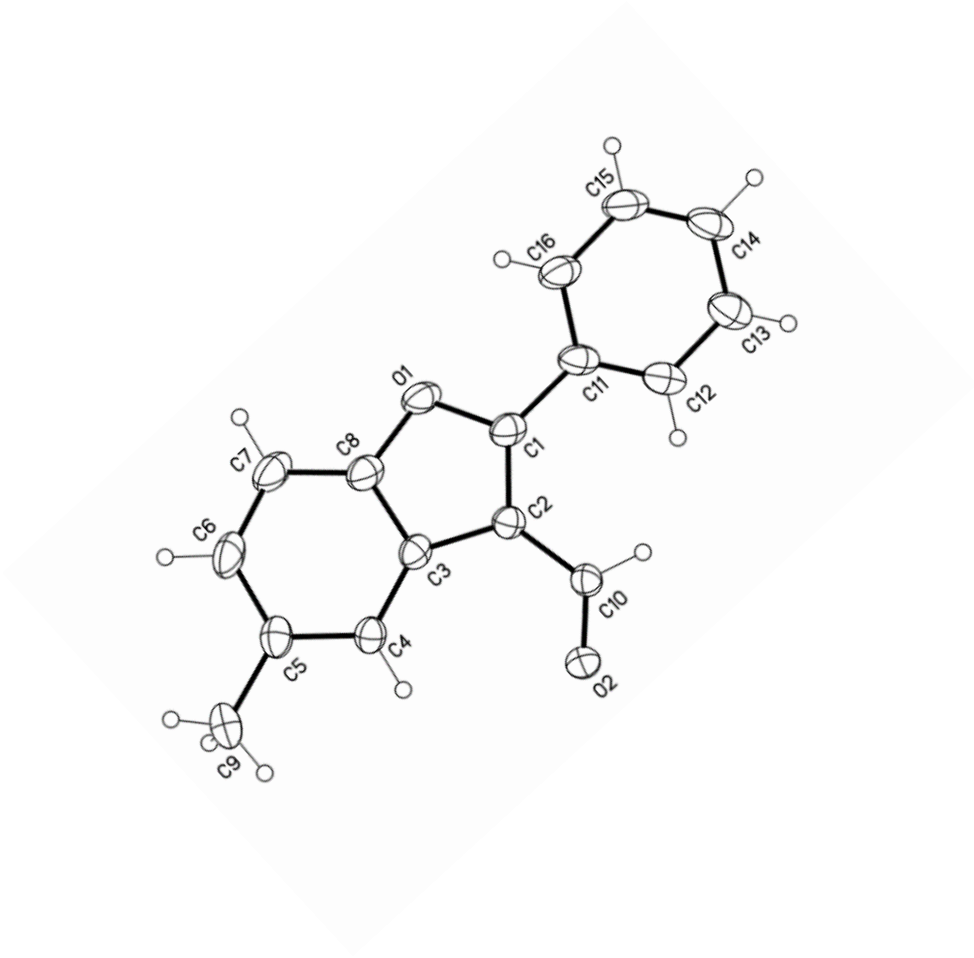

2501006lt2

Table 1 Crystal data and structure refinement for 2501006lt2.

Identification code	2501006lt2
Empirical formula	$C_{16}H_{12}O_2$
Formula weight	236.26
Temperature/K	99.99(10)
Crystal system	orthorhombic
Space group	$P2_12_12_1$
a/Å	6.84930(10)
b/Å	12.36290(10)
c/Å	13.7670(2)
α/°	90
β/°	90
γ/°	90
Volume/Å ^{3}	1165.75(3)
Z	4
ρ_{calc}g/cm^3	1.346
μ/mm^{-1}	0.705
F(000)	496.0
Crystal size/mm^3	0.09 × 0.08 × 0.08
Radiation	Cu Kα (λ = 1.54184)
2Θ range for data collection/°	9.616 to 145.922
Index ranges	$-8 \leq h \leq 7$, $-15 \leq k \leq 14$, $-16 \leq l \leq 16$
Reflections collected	9693
Independent reflections	2245 [R_{int} = 0.0149, R_{sigma} = 0.0117]
Data/restraints/parameters	2245/0/164
Goodness-of-fit on F^2	1.073

Final R indexes [I>=2σ (I)]	R_1 = 0.0299, wR_2 = 0.0761
Final R indexes [all data]	R_1 = 0.0305, wR_2 = 0.0767
Largest diff. peak/hole / e Å $^{-3}$	0.12/-0.20
Flack parameter	-0.04(5)

Table 2 Fractional Atomic Coordinates (×10^4) and Equivalent Isotropic Displacement Parameters (Å^2×10^3) for 2501006lt2. U_{eq} is defined as 1/3 of the trace of the orthogonalised U_{IJ} tensor.

Atom	*x*	*y*	*z*	U(eq)
O1	4561.7(19)	5654.2(9)	5872.1(9)	35.5(3)
O2	4174.6(19)	9117.9(8)	4660.1(8)	32.7(3)
C1	4568(2)	6716.5(12)	6158.4(12)	27.8(3)
C2	4425(2)	7395.6(12)	5374.1(11)	24.1(3)
C3	4295(2)	6710.5(12)	4524.7(12)	24.9(3)
C4	4124(2)	6880.6(12)	3525.2(11)	25.8(3)
C5	4032(2)	5991.2(13)	2906.9(12)	30.2(4)
C6	4110(3)	4940.0(14)	3301.3(15)	40.3(4)
C7	4288(3)	4753.4(14)	4286.1(15)	44.4(5)
C8	4387(3)	5654.6(13)	4873.4(12)	32.2(4)
C9	3809(3)	6145.6(16)	1827.6(12)	36.2(4)
C10	4494(2)	8571.4(12)	5375.7(11)	25.1(3)
C11	4682(2)	6885.1(13)	7211.3(12)	30.5(4)
C12	3719(2)	7751.9(15)	7649.2(12)	31.3(4)
C13	3783(3)	7881.0(16)	8653.8(13)	37.4(4)
C14	4798(3)	7141.9(15)	9218.7(13)	43.9(5)
C15	5764(4)	6284.0(15)	8790.0(14)	48.2(5)
C16	5718(3)	6152.1(14)	7790.7(14)	41.0(4)

Table 3 Anisotropic Displacement Parameters (Å ^{2}×10^3) for 2501006lt2. The Anisotropic displacement factor exponent takes the form: $-2\pi^2[h^2a^{*2}U_{11}+2hka^*b^*U_{12}+\ldots]$.

Atom	U_{11}	U_{22}	U_{33}	U_{23}	U_{13}	U_{12}
O1	48.0(7)	20.8(5)	37.7(6)	6.1(5)	-18.1(6)	-8.0(5)
O2	49.3(7)	23.2(5)	25.4(5)	3.1(4)	0.7(5)	-3.6(5)
C1	28.6(7)	22.3(7)	32.4(8)	3.4(6)	-6.6(7)	-7.1(6)
C2	24.0(7)	23.2(7)	25.1(7)	0.9(6)	-1.1(7)	-3.3(6)
C3	23.1(7)	21.3(7)	30.1(8)	-1.0(6)	-2.8(7)	-3.1(6)
C4	25.3(7)	23.4(7)	28.7(8)	-2.8(6)	1.4(6)	-2.8(6)
C5	26.0(8)	29.4(8)	35.2(8)	-8.5(7)	-2.6(6)	0.1(7)
C6	44.4(10)	25.4(8)	51.0(11)	-13.6(7)	-19.3(9)	2.1(8)
C7	58.4(12)	20.5(8)	54.4(11)	-1.3(7)	-28.5(10)	-2.6(9)
C8	35.4(9)	23.4(8)	37.7(9)	1.6(6)	-13.4(7)	-4.4(7)
C9	38.1(9)	38.7(9)	31.8(8)	-13.4(7)	-0.4(7)	-1.2(8)
C10	30.0(7)	23.3(7)	22.0(7)	-0.4(6)	2.1(7)	-3.3(6)
C11	34.0(8)	28.8(8)	28.8(8)	8.9(7)	-9.5(7)	-12.6(7)
C12	29.2(8)	37.3(9)	27.3(8)	8.9(7)	-4.9(6)	-8.0(7)
C13	39.8(9)	43.8(10)	28.5(8)	4.6(7)	-4.7(7)	-13.6(8)
C14	62.9(13)	40.6(10)	28.0(9)	10.2(8)	-18.9(8)	-25.6(10)
C15	75.9(14)	28.7(9)	40.1(10)	12.1(8)	-31.3(11)	-17.4(10)
C16	57.2(11)	24.2(8)	41.6(10)	7.8(7)	-21.8(9)	-9.5(8)

Table 4 Bond Lengths for 2501006lt2.

Atom	Atom	Length/Å	Atom	Atom	Length/Å
O1	C1	1.3712(19)	C5	C6	1.410(2)
O1	C8	1.3802(19)	C5	C9	1.506(2)
O2	C10	1.2145(19)	C6	C7	1.381(3)

Table 4 Bond Lengths for 2501006lt2.

Atom	Atom	Length/Å	Atom	Atom	Length/Å
C1	C2	1.371(2)	C7	C8	1.378(2)
C1	C11	1.466(2)	C11	C12	1.395(3)
C2	C3	1.447(2)	C11	C16	1.401(2)
C2	C10	1.454(2)	C12	C13	1.393(2)
C3	C4	1.397(2)	C13	C14	1.387(3)
C3	C8	1.392(2)	C14	C15	1.382(3)
C4	C5	1.392(2)	C15	C16	1.386(3)

Table 5 Bond Angles for 2501006lt2.

Atom	Atom	Atom	Angle/°	Atom	Atom	Atom	Angle/°
C1	O1	C8	106.63(12)	C7	C6	C5	122.39(16)
O1	C1	C11	114.86(13)	C8	C7	C6	116.44(16)
C2	C1	O1	111.09(14)	O1	C8	C3	110.35(14)
C2	C1	C11	134.03(15)	C7	C8	O1	126.03(15)
C1	C2	C3	106.41(13)	C7	C8	C3	123.61(15)
C1	C2	C10	127.47(15)	O2	C10	C2	123.29(15)
C3	C2	C10	126.04(14)	C12	C11	C1	120.74(14)
C4	C3	C2	135.50(14)	C12	C11	C16	119.37(15)
C8	C3	C2	105.52(13)	C16	C11	C1	119.86(16)
C8	C3	C4	118.98(14)	C13	C12	C11	120.14(16)
C5	C4	C3	119.16(14)	C14	C13	C12	119.81(19)
C4	C5	C6	119.41(15)	C15	C14	C13	120.40(17)
C4	C5	C9	120.52(15)	C14	C15	C16	120.21(17)
C6	C5	C9	120.06(15)	C15	C16	C11	120.06(19)

Table 6 Torsion Angles for 2501006lt2.

A	B	C	D	Angle/°	A	B	C	D	Angle/°
O1	C1	C2	C3	0.67(18)	C4	C3	C8	C7	-1.1(3)
O1	C1	C2	C10	-176.26(15)	C4	C5	C6	C7	-0.4(3)
O1	C1	C11	C12	-145.58(15)	C5	C6	C7	C8	0.0(3)
O1	C1	C11	C16	32.5(2)	C6	C7	C8	O1	179.85(18)
C1	O1	C8	C3	0.36(19)	C6	C7	C8	C3	0.8(3)
C1	O1	C8	C7	-178.8(2)	C8	O1	C1	C2	-0.65(18)
C1	C2	C3	C4	179.95(18)	C8	O1	C1	C11	178.13(15)
C1	C2	C3	C8	-0.42(18)	C8	C3	C4	C5	0.6(2)
C1	C2	C10	O2	-172.92(16)	C9	C5	C6	C7	-179.13(19)
C1	C11	C12	C13	177.78(15)	C10	C2	C3	C4	-3.1(3)
C1	C11	C16	C15	-177.42(17)	C10	C2	C3	C8	176.57(15)
C2	C1	C11	C12	32.8(3)	C11	C1	C2	C3	-177.79(18)
C2	C1	C11	C16	-149.08(19)	C11	C1	C2	C10	5.3(3)
C2	C3	C4	C5	-179.83(17)	C11	C12	C13	C14	-0.4(3)
C2	C3	C8	O1	0.04(19)	C12	C11	C16	C15	0.7(3)
C2	C3	C8	C7	179.24(19)	C12	C13	C14	C15	0.7(3)
C3	C2	C10	O2	10.7(3)	C13	C14	C15	C16	-0.3(3)
C3	C4	C5	C6	0.1(2)	C14	C15	C16	C11	-0.4(3)
C3	C4	C5	C9	178.84(16)	C16	C11	C12	C13	-0.3(2)
C4	C3	C8	O1	179.74(14)					

Table 7 Hydrogen Atom Coordinates (Å ×10^4) and Isotropic Displacement Parameters (Å ^{2}×10^3) for 2501006lt2.

Atom	*x*	*y*	*z*	U(eq)

Table 7 Hydrogen Atom Coordinates (Å ×10^4) and Isotropic Displacement Parameters (Å ^{2}×10^3) for 2501006lt2.

Atom	*x*	*y*	*z*	U(eq)
H4	4070.76	7594.28	3270.67	31
H6	4038.74	4337.98	2873.93	48
H7	4338.76	4041.73	4545.2	53
H9A	2458.36	5990.03	1638.23	54
H9B	4693.11	5652.91	1485.04	54
H9C	4129.52	6894.46	1657.09	54
H10	4804.54	8929.9	5966.17	30
H12	3017.35	8254.93	7261.4	38
H13	3133.54	8473.74	8951.16	45
H14	4830.14	7225.62	9904.51	53
H15	6462.16	5783.22	9181.75	58
H16	6390.44	5564	7498.4	49

(b) X-ray crystallographic data of compound (2k):

Ellipsoid contour %probability level = 50%

Experimental: The sample was dissolved in appropriate amount of ethyl acetate followed by the addition of pentane to furnish a saturated solution. Afterwards, the mixture was allowed to stand at room temperature to form the crystals.

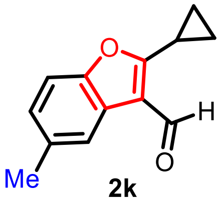

2k

CCDC: 2488740

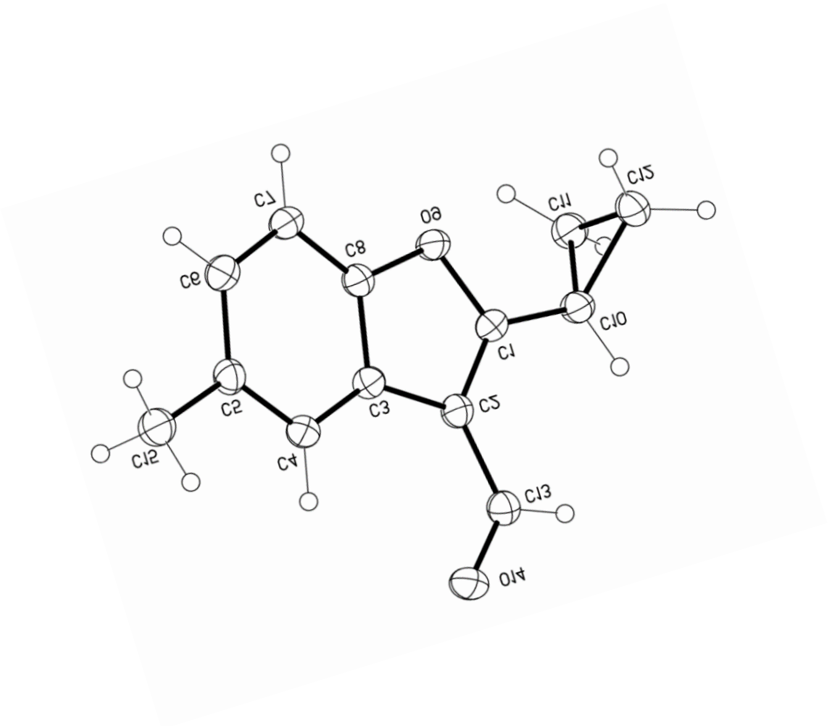

2507023lt_auto

Table 8 Crystal data and structure refinement for 2507023lt_auto.

Identification code	2507023lt_auto
Empirical formula	$C_{13}H_{12}O_2$
Formula weight	200.23
Temperature/K	100.00(12)
Crystal system	monoclinic
Space group	$P2_1/c$
a/Å	8.8522(4)
b/Å	12.0072(6)
c/Å	9.4376(5)
α/°	90
β/°	100.692(5)
γ/°	90
Volume/Å ^{3}	985.70(9)
Z	4
$\rho_{calc}g/cm^3$	1.349

μ/mm^{-1}	0.724
F(000)	424.0
Crystal size/mm^3	0.07 × 0.04 × 0.03
Radiation	Cu Kα (λ = 1.54184)
2Θ range for data collection/°	10.17 to 144.958
Index ranges	-10 ≤ h ≤ 10, -14 ≤ k ≤ 13, -11 ≤ l ≤ 10
Reflections collected	6749
Independent reflections	1888 [R_{int} = 0.0249, R_{sigma} = 0.0268]
Data/restraints/parameters	1888/0/137
Goodness-of-fit on F^2	1.049
Final R indexes [I>=2σ (I)]	R_1 = 0.0417, wR_2 = 0.1048
Final R indexes [all data]	R_1 = 0.0516, wR_2 = 0.1110
Largest diff. peak/hole / e Å^{-3}	0.23/-0.24

Table 9 Fractional Atomic Coordinates (×10^4) and Equivalent Isotropic Displacement Parameters (Å^2×10^3) for 2507023lt_auto. U_{eq} is defined as 1/3 of the trace of the orthogonalised U_{IJ} tensor.

Atom	*x*	*y*	*z*	U(eq)
O9	3304.5(11)	6003.4(9)	829.2(11)	22.6(3)
O14	-515.7(12)	4567.4(9)	-2695.8(12)	27.7(3)
C1	2146.1(17)	6298.2(13)	-280.5(16)	20.9(3)
C2	1401.5(17)	5383.0(13)	-935.6(16)	21.2(3)
C3	2137.4(17)	4419.9(13)	-174.9(16)	20.7(3)
C4	1891.9(17)	3273.7(13)	-248.2(16)	21.8(3)
C5	2780.4(17)	2586.6(13)	767.9(16)	22.4(4)
C6	3913.2(18)	3063.5(14)	1842.2(17)	24.8(4)
C7	4192.5(17)	4201.6(14)	1924.0(17)	24.2(4)

C8	3281.2(17)	4845.6(13)	898.9(16)	21.4(3)
C10	1918.7(17)	7485.9(13)	-559.4(16)	22.0(4)
C11	3294.1(18)	8262.7(13)	-362.4(17)	24.6(4)
C12	2211.1(18)	8313.3(13)	668.0(17)	24.5(4)
C13	93.5(17)	5400.0(13)	-2102.8(16)	22.3(4)
C15	2546.2(19)	1343.4(13)	720.1(18)	27.6(4)

Table 10 Anisotropic Displacement Parameters (Å ^{2}×10^3) for 2507023lt_auto. The Anisotropic displacement factor exponent takes the form: $-2\pi^2[h^2a^{*2}U_{11}+2hka^*b^*U_{12}+\ldots]$.

Atom	U_{11}	U_{22}	U_{33}	U_{23}	U_{13}	U_{12}
O9	22.8(5)	18.1(6)	23.9(6)	-0.5(4)	-3.1(4)	-0.2(4)
O14	28.1(6)	24.8(6)	27.6(6)	-4.9(5)	-1.8(5)	-2.7(5)
C1	20.2(7)	21.4(8)	19.7(7)	0.6(6)	0.7(6)	1.8(6)
C2	22.2(7)	17.7(8)	23.1(8)	0.2(6)	2.5(6)	0.7(6)
C3	20.1(7)	21.9(8)	19.6(7)	-0.5(6)	2.5(6)	1.3(6)
C4	21.5(7)	19.8(8)	23.3(8)	-1.8(6)	1.9(6)	-0.9(6)
C5	23.4(8)	18.5(8)	25.3(8)	0.3(6)	4.2(6)	1.6(6)
C6	26.2(8)	23.1(9)	23.7(8)	1.8(6)	1.4(6)	2.8(7)
C7	22.4(8)	22.7(9)	24.9(8)	-0.8(6)	-2.2(6)	1.3(6)
C8	23.3(8)	17.2(8)	22.8(8)	-0.9(6)	1.7(6)	0.7(6)
C10	23.0(8)	18.4(8)	22.7(8)	0.5(6)	-0.5(6)	-0.6(6)
C11	25.5(8)	19.6(8)	27.5(8)	1.0(6)	1.6(6)	-1.5(6)
C12	28.2(8)	18.4(8)	26.0(8)	-2.0(6)	2.8(6)	0.2(6)
C13	23.8(8)	20.2(8)	22.6(8)	-0.1(6)	3.4(6)	1.0(6)
C15	29.1(8)	20.9(9)	31.1(9)	1.3(7)	1.2(7)	-0.6(7)

Table 11 Bond Lengths for 2507023lt_auto.

Atom	Atom	Length/Å	Atom	Atom	Length/Å
O9	C1	1.3694(17)	C4	C5	1.393(2)
O9	C8	1.3921(18)	C5	C6	1.409(2)
O14	C13	1.2212(18)	C5	C15	1.507(2)
C1	C2	1.369(2)	C6	C7	1.388(2)
C1	C10	1.457(2)	C7	C8	1.377(2)
C2	C3	1.450(2)	C10	C11	1.518(2)
C2	C13	1.443(2)	C10	C12	1.512(2)
C3	C4	1.393(2)	C11	C12	1.488(2)
C3	C8	1.390(2)			

Table 12 Bond Angles for 2507023lt_auto.

Atom	Atom	Atom	Angle/˚	Atom	Atom	Atom	Angle/˚
C1	O9	C8	106.19(11)	C6	C5	C15	120.00(14)
O9	C1	C10	116.73(13)	C7	C6	C5	122.40(15)
C2	C1	O9	111.56(13)	C8	C7	C6	116.02(14)
C2	C1	C10	131.71(14)	C3	C8	O9	110.36(13)
C1	C2	C3	106.39(13)	C7	C8	O9	125.61(13)
C1	C2	C13	125.78(14)	C7	C8	C3	123.94(15)
C13	C2	C3	127.76(14)	C1	C10	C11	119.90(13)
C4	C3	C2	135.36(14)	C1	C10	C12	120.43(13)
C8	C3	C2	105.4(13)	C12	C10	C11	58.85(10)
C8	C3	C4	119.03(14)	C12	C11	C10	60.38(10)

C5	C4	C3	119.22(14)	C11	C12	C10	60.78(10)
C4	C5	C6	119.37(15)	O14	C13	C2	124.19(15)
C4	C5	C15	120.62(14)				

Table 13 Torsion Angles for 2507023lt_auto.

A	B	C	D	Angle/°	A	B	C	D	Angle/°
O9	C1	C2	C3	0.43(17)	C3	C4	C5	C6	-0.4(2)
O9	C1	C2	C13	177.55(13)	C3	C4	C5	C15	-179.97(14)
O9	C1	C10	C11	35.7(2)	C4	C3	C8	O9	-177.54(13)
O9	C1	C10	C12	-33.6(2)	C4	C3	C8	C7	-0.8(2)
C1	O9	C8	C3	1.11(16)	C4	C5	C6	C7	-0.5(2)
C1	O9	C8	C7	-175.56(15)	C5	C6	C7	C8	0.7(2)
C1	C2	C3	C4	176.14(17)	C6	C7	C8	O9	176.16(14)
C1	C2	C3	C8	0.26(16)	C6	C7	C8	C3	-0.1(2)
C1	C2	C13	O14	177.55(15)	C8	O9	C1	C2	-0.94(16)
C1	C10	C11	C12	-109.58(16)	C8	O9	C1	C10	179.10(13)
C1	C10	C12	C11	108.69(16)	C8	C3	C4	C5	1.0(2)
C2	C1	C10	C11	-144.28(17)	C10	C1	C2	C3	-179.62(15)
C2	C1	C10	C12	146.47(16)	C10	C1	C2	C13	-2.5(3)
C2	C3	C4	C5	-174.47(16)	C13	C2	C3	C4	-0.9(3)
C2	C3	C8	O9	-0.84(17)	C13	C2	C3	C8	-176.78(15)
C2	C3	C8	C7	175.89(14)	C15	C5	C6	C7	179.06(15)
C3	C2	C13	O14	-6.0(3)					

Table 14 Hydrogen Atom Coordinates (Å × 10^4) and Isotropic Displacement Parameters (Å ^{2}× 10^3) for 2507023lt_auto.

Atom	*x*	*y*	*z*	U(eq)
H4	1127.29	2964.46	-982.81	26
H6	4507.84	2589.07	2536.01	30
H7	4966.46	4517.2	2644.5	29
H10	1060.03	7681.74	-1367.83	26
H11A	4323.33	7936.2	-15.91	30
H11B	3272.51	8883.8	-1055.7	30
H12A	1520.63	8966.73	613.35	29
H12B	2571.17	8019.39	1652.85	29
H13	-320.48	6103.87	-2431.1	27
H15A	1455.67	1177.15	377.49	41
H15B	2873.81	1033.19	1688.89	41
H15C	3156.63	1011.11	62.8	41

(c) X-ray crystallographic data of compound (3a):

Ellipsoid contour %probability level = 50%

Experimental: The sample was dissolved in appropriate amount of Dichloromethane followed by the addition of pentane to furnish a saturated solution. Afterwards, the mixture was allowed to stand at room temperature to form the crystals.

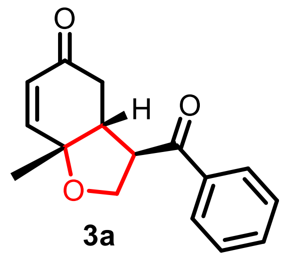

3a

CCDC: 2447419

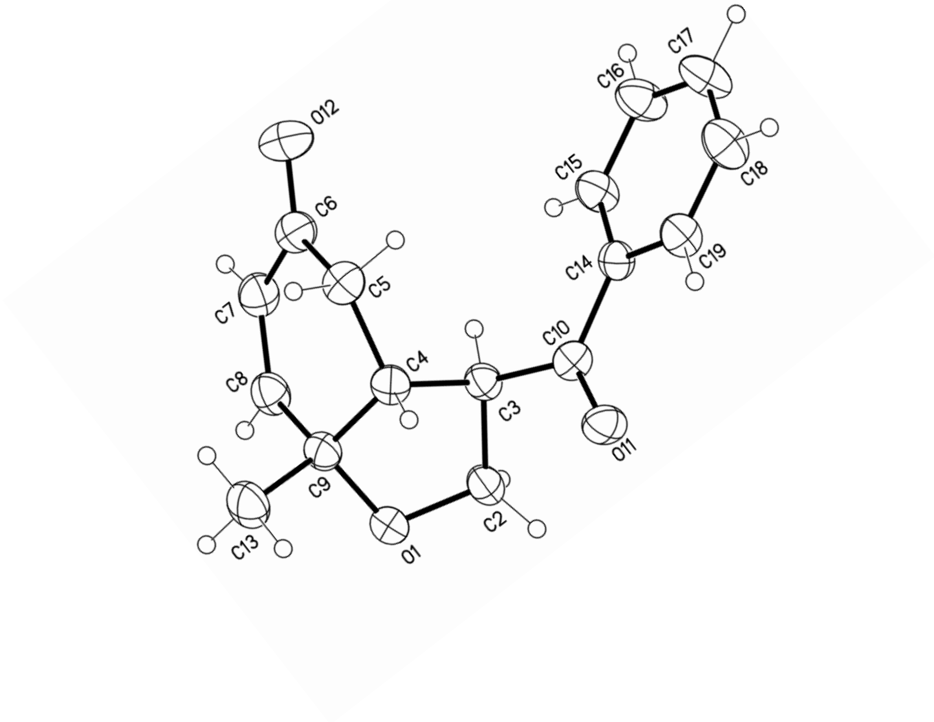

2503134lt_auto

Table 15 Crystal data and structure refinement for 2503134lt_auto.

Identification code	2503134lt_auto
Empirical formula	$C_{16}H_{16}O_3$
Formula weight	256.29
Temperature/K	99.97(13)
Crystal system	monoclinic
Space group	$P2_1/n$
a/Å	7.5157(4)
b/Å	17.5135(7)
c/Å	10.1424(5)
α/°	90
β/°	103.492(5)
γ/°	90
Volume/Å ^{3}	1298.17(10)
Z	4

ρ_{calc}g/cm^3	1.311
μ/mm^{-1}	0.727
F(000)	544.0
Crystal size/mm^3	0.06 × 0.04 × 0.03
Radiation	Cu Kα (λ = 1.54184)
2Θ range for data collection/°	10.102 to 146.502
Index ranges	-8 ≤ h ≤ 9, -18 ≤ k ≤ 21, -12 ≤ l ≤ 12
Reflections collected	6903
Independent reflections	2497 [R_{int} = 0.0266, R_{sigma} = 0.0373]
Data/restraints/parameters	2497/0/174
Goodness-of-fit on F^2	1.034
Final R indexes [I>=2σ (I)]	R_1 = 0.0482, wR_2 = 0.1044
Final R indexes [all data]	R_1 = 0.0722, wR_2 = 0.1137
Largest diff. peak/hole / e Å^{-3}	0.19/-0.22

Table 16 Fractional Atomic Coordinates (×10^4) and Equivalent Isotropic Displacement Parameters (Å^2×10^3) for 2503134lt_auto. U_{eq} is defined as 1/3 of the trace of the orthogonalised U_{IJ} tensor.

**Atom**	***x***	***y***	***z***	**U(eq)**
O1	4920(2)	4152.0(7)	929.2(14)	34.2(4)
O11	5158.9(19)	2223.9(8)	2837.4(13)	34.5(4)
O12	5561(2)	2154.7(9)	-2887.4(15)	42.6(4)
C2	3716(3)	3571.6(11)	1228(2)	32.5(5)
C3	4271(3)	2816.4(10)	677.1(19)	26.7(4)
C4	6198(3)	2996.4(11)	466.0(19)	28.1(4)
C5	6798(3)	2496.8(12)	-584(2)	33.0(5)

Table 16 Fractional Atomic Coordinates (×10^4) and Equivalent Isotropic Displacement Parameters (Å^2×10^3) for 2503134lt_auto. U_{eq} is defined as 1/3 of the trace of the orthogonalised U_{IJ} tensor.

Atom	*x*	*y*	*z*	U(eq)
C6	5743(3)	2649.0(12)	-2006(2)	32.4(5)
C7	4975(3)	3412.8(12)	-2323(2)	34.0(5)
C8	5113(3)	3952.0(12)	-1379(2)	32.1(5)
C9	6047(3)	3841.0(11)	89.3(19)	30.0(5)
C10	4396(3)	2143.2(11)	1635.2(19)	27.8(4)
C13	7860(3)	4262.6(13)	420(2)	40.5(5)
C14	3716(3)	1379.1(11)	1102.7(19)	26.8(4)
C15	2542(3)	1274.4(11)	-171(2)	32.5(5)
C16	1933(3)	552.4(12)	-608(2)	40.8(5)
C17	2475(3)	-71.1(12)	225(2)	42.6(6)
C18	3638(3)	21.5(12)	1487(2)	39.8(5)
C19	4260(3)	741.4(11)	1926(2)	32.4(5)

Table 17 Anisotropic Displacement Parameters (Å^2×10^3) for 2503134lt_auto. The Anisotropic displacement factor exponent takes the form: $-2\pi^2[h^2a^{*2}U_{11}+2hka^*b^*U_{12}+\ldots]$.

Atom	U_{11}	U_{22}	U_{33}	U_{23}	U_{13}	U_{12}
O1	42.9(8)	27.6(7)	34.5(8)	-3.9(6)	14.2(6)	-6.7(6)
O11	40.2(8)	35.8(8)	24.3(7)	-2.1(6)	1.1(6)	-1.1(6)
O12	37.8(9)	50.3(9)	37.4(9)	-14.3(7)	4.4(7)	4.1(7)
C2	35.2(11)	25.6(10)	39.0(11)	-0.7(9)	13.8(9)	-4.5(9)
C3	27.6(10)	26.1(10)	25.5(10)	0.5(8)	4.2(8)	-1.4(8)
C4	24.1(9)	33.3(11)	24.2(9)	0.0(8)	0.5(8)	-2.0(8)
C5	29.2(11)	37.1(12)	32.2(11)	0.1(9)	6.5(9)	2.6(9)

Table 17 Anisotropic Displacement Parameters (Å ^{2}×10^3) for 2503134lt_auto. The Anisotropic displacement factor exponent takes the form: $-2\pi^2[h^2a^{*2}U_{11}+2hka^*b^*U_{12}+\ldots]$.

Atom	U_{11}	U_{22}	U_{33}	U_{23}	U_{13}	U_{12}
C6	25.5(10)	39.5(12)	32.0(11)	-4.2(9)	6.5(8)	-2.3(9)
C7	34.4(11)	39.2(12)	25.8(10)	2.7(9)	2.0(9)	-4.7(9)
C8	33.3(11)	30.3(11)	31.5(11)	3.7(9)	5.1(9)	-2.5(9)
C9	30.4(10)	31.6(11)	27.8(10)	-2.0(8)	6.0(8)	-6.8(9)
C10	25.5(10)	31.8(11)	25.4(10)	0.4(8)	4.2(8)	2.3(8)
C13	36.5(12)	44.5(13)	38.2(12)	-1.0(10)	4.2(10)	-13.4(10)
C14	25.0(10)	28.4(10)	27.0(10)	1.6(8)	6.0(8)	-0.2(8)
C15	35.6(11)	27.9(11)	30.5(11)	3.1(8)	0.3(9)	-0.5(9)
C16	48.2(13)	32.1(11)	35.0(12)	-0.7(9)	-4.8(10)	-3.3(10)
C17	46.9(13)	24.6(11)	50.6(14)	-1.5(10)	-0.3(11)	-3.5(10)
C18	39.2(12)	31.3(11)	44.3(13)	10.9(10)	0.3(10)	2.0(10)
C19	32.1(11)	32.4(11)	29.6(11)	4.4(9)	0.9(9)	0.0(9)

Table 18 Bond Lengths for 2503134lt_auto.

Atom	Atom	Length/Å	Atom	Atom	Length/Å
O1	C2	1.440(2)	C7	C8	1.331(3)
O1	C9	1.441(2)	C8	C9	1.504(3)
O11	C10	1.228(2)	C9	C13	1.517(3)
O12	C6	1.229(2)	C10	C14	1.488(3)
C2	C3	1.531(3)	C14	C15	1.396(3)
C3	C4	1.545(3)	C14	C19	1.398(3)
C3	C10	1.517(3)	C15	C16	1.382(3)
C4	C5	1.525(3)	C16	C17	1.382(3)

Table 18 Bond Lengths for 2503134lt_auto.

Atom	Atom	Length/Å	Atom	Atom	Length/Å
C4	C9	1.525(3)	C17	C18	1.381(3)
C5	C6	1.499(3)	C18	C19	1.382(3)
C6	C7	1.463(3)			

Table 19 Bond Angles for 2503134lt_auto.

Atom	Atom	Atom	Angle/˚	Atom	Atom	Atom	Angle/˚
C2	O1	C9	110.05(14)	O1	C9	C13	107.88(16)
O1	C2	C3	107.14(15)	C8	C9	C4	111.33(16)
C2	C3	C4	102.62(15)	C8	C9	C13	110.09(16)
C10	C3	C2	114.78(16)	C13	C9	C4	113.96(17)
C10	C3	C4	109.10(15)	O11	C10	C3	119.43(17)
C5	C4	C3	114.76(16)	O11	C10	C14	120.32(17)
C5	C4	C9	113.48(16)	C14	C10	C3	120.11(16)
C9	C4	C3	102.42(15)	C15	C14	C10	122.91(17)
C6	C5	C4	113.19(16)	C15	C14	C19	118.81(18)
O12	C6	C5	121.26(19)	C19	C14	C10	118.27(17)
O12	C6	C7	120.87(19)	C16	C15	C14	120.47(18)
C7	C6	C5	117.85(17)	C15	C16	C17	119.9(2)
C8	C7	C6	122.01(19)	C18	C17	C16	120.4(2)
C7	C8	C9	124.09(19)	C17	C18	C19	119.94(19)
O1	C9	C4	103.78(15)	C18	C19	C14	120.45(19)
O1	C9	C8	109.49(16)				

Table 20 Torsion Angles for 2503134lt_auto.

A	B	C	D	Angle/˚	A	B	C	D	Angle/˚
O1	C2	C3	C4	16.66(19)	C4	C5	C6	C7	27.5(3)
O1	C2	C3	C10	134.86(16)	C5	C4	C9	O1	161.50(15)
O11	C10	C14	C15	167.34(19)	C5	C4	C9	C8	43.8(2)
O11	C10	C14	C19	-11.7(3)	C5	C4	C9	C13	-81.4(2)
O12	C6	C7	C8	178.24(19)	C5	C6	C7	C8	-3.4(3)
C2	O1	C9	C4	-28.04(19)	C6	C7	C8	C9	-0.2(3)
C2	O1	C9	C8	90.92(18)	C7	C8	C9	O1	-134.6(2)
C2	O1	C9	C13	-149.27(16)	C7	C8	C9	C4	-20.4(3)
C2	C3	C4	C5	-155.89(16)	C7	C8	C9	C13	106.9(2)
C2	C3	C4	C9	-32.47(18)	C9	O1	C2	C3	6.9(2)
C2	C3	C10	O11	-44.5(2)	C9	C4	C5	C6	-48.2(2)
C2	C3	C10	C14	139.69(18)	C10	C3	C4	C5	82.0(2)
C3	C4	C5	C6	69.1(2)	C10	C3	C4	C9	-154.62(15)
C3	C4	C9	O1	37.22(18)	C10	C14	C15	C16	-179.2(2)
C3	C4	C9	C8	-80.46(19)	C10	C14	C19	C18	178.82(19)
C3	C4	C9	C13	154.28(17)	C14	C15	C16	C17	0.5(3)
C3	C10	C14	C15	-16.9(3)	C15	C14	C19	C18	-0.3(3)
C3	C10	C14	C19	163.99(18)	C15	C16	C17	C18	-0.7(4)
C4	C3	C10	O11	70.0(2)	C16	C17	C18	C19	0.3(4)
C4	C3	C10	C14	-105.82(19)	C17	C18	C19	C14	0.2(3)
C4	C5	C6	O12	-154.09(19)	C19	C14	C15	C16	-0.1(3)

Table 21 Hydrogen Atom Coordinates (Å ×10^4) and Isotropic Displacement Parameters (Å ^{2}×10^3) for 2503134lt_auto.

**Atom**	***x***	***y***	***z***	**U(eq)**
H2A	2428.66	3695.83	789.23	39
H2B	3835.04	3535.16	2218.81	39
H3	3419.1	2694.07	-212.21	32
H4	7106.59	2938.32	1353.73	34
H5A	8114.52	2585.12	-527.67	40
H5B	6644.09	1953.73	-363.27	40
H7	4359.34	3527.75	-3230.32	41
H8	4588.35	4437.21	-1650.38	38
H13A	7662.52	4796.61	134.25	61
H13B	8703.21	4022.54	-60.96	61
H13C	8384.22	4241	1398.65	61
H15	2159.14	1702.52	-741.96	39
H16	1143.12	484.57	-1478.7	49
H17	2045.2	-566.31	-72.95	51
H18	4010.33	-409.45	2053.54	48
H19	5063.03	803.36	2794.06	39

d) X-ray crystallographic data of compound (5a):

Ellipsoid contour %probability level = 50%

Experimental: The sample was dissolved in appropriate amount of Dichloromethane followed by the addition of pentane to furnish a saturated solution. Afterwards, the mixture was allowed to stand at room temperature to form the crystals.

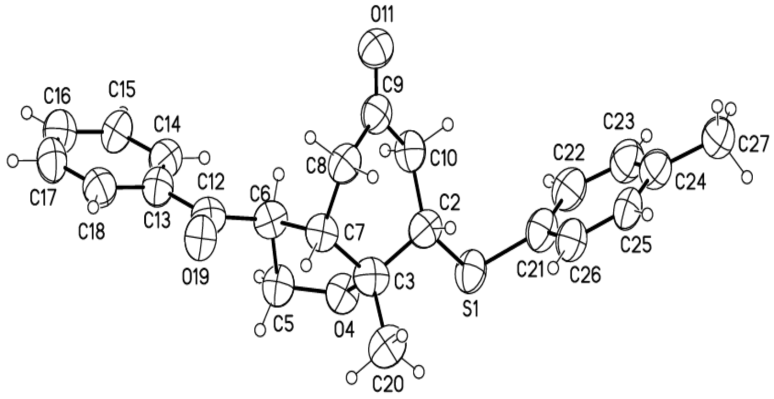

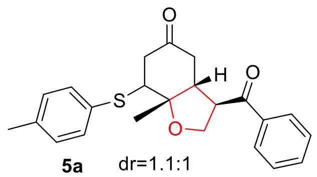

5a dr=1.1:1

CCDC: 2497015

2509056lt_2_twin1

Table 22 Crystal data and structure refinement for 2509056lt_2_twin1.

Identification code	2509056lt_2_twin1
Empirical formula	$C_{23}H_{24}O_3S$
Formula weight	380.48
Temperature/K	100.00(14)
Crystal system	monoclinic
Space group	$P2_1$
a/Å	15.0069(3)
b/Å	5.70610(10)
c/Å	23.2135(5)
α/°	90
β/°	92.671(2)
γ/°	90
Volume/Å ^{3}	1985.63(7)
Z	4
ρ_{calc}g/cm^3	1.273
μ/mm^{-1}	1.605
F(000)	808.0

Crystal size/mm^3	0.08 × 0.04 × 0.03
Radiation	Cu Kα (λ = 1.54184)
2Θ range for data collection/°	5.896 to 134.16
Index ranges	-17 ≤ h ≤ 17, -6 ≤ k ≤ 6, -27 ≤ l ≤ 27
Reflections collected	30284
Independent reflections	6990 [R_{int} = 0.0636, R_{sigma} = 0.0516]
Data/restraints/parameters	6990/1/491
Goodness-of-fit on F^2	1.065
Final R indexes [I>=2σ (I)]	R_1 = 0.0880, wR_2 = 0.2530
Final R indexes [all data]	R_1 = 0.0982, wR_2 = 0.2625
Largest diff. peak/hole / e Å^{-3}	0.64/-0.37
Flack parameter	-0.01(3)

Table 23 Fractional Atomic Coordinates (×10^4) and Equivalent Isotropic Displacement Parameters (Å^2×10^3) for 2509056lt_2_twin1. U_{eq} is defined as 1/3 of the trace of the orthogonalised U_{IJ} tensor.

**Atom**	***x***	***y***	***z***	**U(eq)**
S1	8040.5(15)	7922(4)	9493.7(10)	56.4(6)
C2	7271(6)	5418(16)	9439(4)	52(2)
C3	6943(6)	5135(15)	8815(4)	54(2)
O4	6624(4)	7399(11)	8607(3)	53.5(13)
C5	5788(6)	7115(16)	8288(4)	51.6(18)
C6	5308(6)	5155(15)	8615(4)	50.3(19)
C7	6087(6)	3508(14)	8773(4)	47.3(18)
C8	5946(6)	2024(16)	9307(4)	52.4(19)
C9	5947(6)	3473(15)	9850(4)	50.4(19)
C10	6544(6)	5631(16)	9863(4)	52(2)

Table 23 Fractional Atomic Coordinates (×10^4) and Equivalent Isotropic Displacement Parameters (Å^2×10^3) for 2509056lt_2_twin1. U_{eq} is defined as 1/3 of the trace of the orthogonalised U_{IJ} tensor.

Atom	*x*	*y*	*z*	U(eq)
O11	5505(4)	2978(11)	10257(3)	58.1(15)
C12	4551(6)	4060(15)	8255(4)	52(2)
C13	3681(6)	5293(16)	8193(4)	51(2)
C14	3508(6)	7372(18)	8482(4)	56(2)
C15	2684(6)	8470(17)	8414(4)	56(2)
C16	2015(7)	7492(17)	8062(4)	61(2)
C17	2182(7)	5410(18)	7769(4)	61(2)
C18	2999(6)	4340(18)	7832(4)	57(2)
O19	4668(4)	2178(11)	8014(3)	56.3(14)
C20	7653(7)	4259(18)	8428(4)	63(2)
C21	8660(6)	7188(17)	10142(4)	56(2)
C22	8662(6)	8744(17)	10607(4)	60(2)
C23	9201(6)	8230(20)	11103(5)	65(2)
C24	9681(6)	6217(19)	11152(4)	59(2)
C25	9665(6)	4635(18)	10693(4)	55(2)
C26	9165(6)	5132(17)	10187(4)	56(2)
C27	10259(7)	5650(20)	11697(4)	71(3)
S28	6891.4(15)	7193(4)	4586.1(10)	57.3(6)
C29	7630(6)	4688(17)	4495(4)	55(2)
C30	7860(6)	4403(15)	3867(4)	53(2)
O31	8155(4)	6692(10)	3666(3)	56.5(15)
C32	8893(6)	6357(16)	3321(4)	56(2)
C33	9433(6)	4396(15)	3626(4)	50.0(19)

Table 23 Fractional Atomic Coordinates (×10^4) and Equivalent Isotropic Displacement Parameters (Å^2×10^3) for 2509056lt_2_twin1. U_{eq} is defined as 1/3 of the trace of the orthogonalised U_{IJ} tensor.

Atom	*x*	*y*	*z*	U(eq)
C34	8690(6)	2761(15)	3795(4)	50.6(18)
C35	8930(6)	1295(16)	4330(4)	54(2)
C36	9042(6)	2702(17)	4870(4)	55(2)
C37	8458(6)	4842(17)	4905(4)	56(2)
O38	9554(4)	2166(12)	5268(3)	56.6(14)
C39	10094(6)	3299(15)	3223(4)	50.8(19)
C40	10989(6)	4386(17)	3170(4)	54(2)
C41	11576(6)	3393(17)	2801(4)	58(2)
C42	12394(7)	4360(20)	2712(5)	65(2)
C43	12656(7)	6390(20)	3013(4)	66(2)
C44	12086(6)	7399(18)	3394(4)	61(2)
C45	11251(6)	6441(18)	3467(4)	58(2)
O46	9876(4)	1558(11)	2938(3)	59.5(16)
C47	7055(7)	3622(18)	3496(5)	66(3)
C48	6346(6)	6297(17)	5212(4)	56(2)
C49	5848(6)	4265(18)	5226(4)	56(2)
C50	5402(6)	3649(18)	5707(4)	58(2)
C51	5408(6)	5099(18)	6188(4)	58(2)
C52	5893(6)	7180(19)	6171(4)	60(2)
C53	6369(7)	7770(18)	5694(4)	64(2)
C54	4889(7)	4470(20)	6709(5)	70(3)

Table 24 Anisotropic Displacement Parameters ($Å^2×10^3$) for 2509056lt_2_twin1. The Anisotropic displacement factor exponent takes the form: $-2\pi^2[h^2a^{*2}U_{11}+2hka^*b^*U_{12}+…]$.

Atom	U_{11}	U_{22}	U_{33}	U_{23}	U_{13}	U_{12}
S1	50.3(11)	47.7(11)	70.4(13)	5.7(9)	-5.0(9)	-4.1(9)
C2	49(5)	45(4)	63(5)	8(4)	6(4)	3(4)
C3	63(6)	41(5)	59(5)	7(4)	9(4)	9(4)
O4	56(3)	43(3)	61(3)	4(3)	2(2)	2(3)
C5	53(5)	38(4)	63(5)	2(4)	-2(4)	-3(4)
C6	53(5)	41(4)	57(5)	-1(4)	8(4)	2(4)
C7	54(5)	36(4)	52(4)	0(3)	4(3)	2(3)
C8	49(4)	42(4)	66(5)	3(4)	4(4)	-2(4)
C9	47(4)	45(5)	58(5)	4(4)	0(4)	5(3)
C10	54(5)	45(5)	57(5)	-2(4)	-4(4)	-2(4)
O11	59(4)	52(3)	64(4)	5(3)	6(3)	-1(3)
C12	60(5)	41(5)	56(5)	7(4)	6(4)	0(4)
C13	50(5)	46(4)	58(5)	4(4)	-1(4)	-2(4)
C14	53(5)	56(5)	60(5)	2(4)	5(4)	0(4)
C15	43(5)	54(5)	70(6)	-2(4)	0(4)	2(4)
C16	62(5)	52(6)	68(5)	3(4)	-3(4)	5(4)
C17	59(5)	60(6)	63(5)	2(4)	-13(4)	-6(4)
C18	61(5)	54(5)	57(5)	4(4)	1(4)	-7(4)
O19	56(3)	43(3)	69(4)	-4(3)	-6(3)	3(3)
C20	62(6)	49(5)	78(6)	-1(4)	9(5)	-3(4)
C21	49(4)	46(4)	72(5)	4(4)	-2(4)	3(4)
C22	54(5)	50(5)	77(6)	-2(4)	3(4)	-4(4)
C23	55(5)	68(6)	72(6)	-9(5)	2(4)	-7(5)

Table 24 Anisotropic Displacement Parameters (Å ^{2}×10^3) for 2509056lt_2_twin1. The Anisotropic displacement factor exponent takes the form: $-2\pi^2[h^2a^{*2}U_{11}+2hka^*b^*U_{12}+\ldots]$.

Atom	U_{11}	U_{22}	U_{33}	U_{23}	U_{13}	U_{12}
C24	51(5)	64(5)	63(5)	3(4)	2(4)	-7(5)
C25	39(4)	57(5)	68(5)	4(4)	-5(4)	2(4)
C26	50(5)	51(5)	67(5)	-2(4)	-3(4)	-2(4)
C27	58(6)	90(8)	65(6)	10(5)	-2(5)	-15(5)
S28	55.0(12)	47.7(11)	69.5(13)	5.0(10)	5.9(9)	5.4(10)
C29	51(5)	48(5)	66(5)	4(4)	2(4)	1(4)
C30	49(5)	38(4)	71(5)	7(4)	-9(4)	-4(4)
O31	66(4)	39(3)	65(4)	7(3)	4(3)	3(3)
C32	62(5)	43(4)	63(5)	5(4)	9(4)	2(4)
C33	48(5)	43(4)	59(5)	-1(4)	-4(4)	5(4)
C34	52(4)	40(4)	60(5)	2(3)	-1(4)	-4(4)
C35	54(5)	42(4)	66(5)	2(4)	2(4)	-1(4)
C36	61(5)	50(5)	54(5)	4(4)	8(4)	-4(4)
C37	61(5)	46(5)	61(5)	1(4)	2(4)	6(4)
O38	59(3)	50(3)	60(3)	5(3)	-3(3)	-1(3)
C39	53(5)	44(5)	54(5)	-1(4)	-4(4)	5(4)
C40	55(5)	49(5)	57(5)	3(4)	1(4)	2(4)
C41	58(5)	53(5)	62(5)	-3(4)	-5(4)	4(4)
C42	54(5)	66(6)	76(6)	5(5)	8(4)	7(5)
C43	57(5)	69(6)	70(6)	11(5)	-1(4)	-5(5)
C44	65(6)	54(5)	64(5)	-3(4)	2(4)	-9(5)
C45	54(5)	61(6)	60(5)	-2(4)	3(4)	-8(4)
O46	63(4)	48(4)	68(4)	-8(3)	6(3)	-4(3)

Table 24 Anisotropic Displacement Parameters (Å ^{2}×10^3) for 2509056lt_2_twin1. The Anisotropic displacement factor exponent takes the form: $-2\pi^2[h^2a^{*2}U_{11}+2hka^*b^*U_{12}+\ldots]$.

Atom	U_{11}	U_{22}	U_{33}	U_{23}	U_{13}	U_{12}
C47	57(6)	54(6)	86(7)	-8(5)	-5(5)	-2(4)
C48	52(5)	49(5)	66(5)	-1(4)	-4(4)	6(4)
C49	46(5)	60(5)	61(5)	-2(4)	-5(4)	-1(4)
C50	54(5)	60(6)	62(5)	3(4)	5(4)	3(4)
C51	54(5)	59(6)	61(5)	2(4)	-6(4)	8(4)
C52	55(5)	62(5)	63(5)	-10(5)	1(4)	-2(4)
C53	73(6)	50(5)	67(6)	-3(4)	-3(5)	-1(5)
C54	71(6)	68(6)	72(6)	9(5)	7(5)	7(5)

Table 25 Bond Lengths for 2509056lt_2_twin1.

Atom	Atom	Length/Å	Atom	Atom	Length/Å
S1	C2	1.837(9)	S28	C29	1.827(10)
S1	C21	1.782(9)	S28	C48	1.775(10)
C2	C3	1.518(13)	C29	C30	1.524(13)
C2	C10	1.508(12)	C29	C37	1.531(12)
C3	O4	1.452(10)	C30	O31	1.462(10)
C3	C7	1.585(13)	C30	C34	1.573(12)
C3	C20	1.510(13)	C30	C47	1.517(12)
O4	C5	1.436(10)	O31	C32	1.409(11)
C5	C6	1.548(12)	C32	C33	1.535(12)
C6	C7	1.531(11)	C33	C34	1.520(12)
C6	C12	1.513(13)	C33	C39	1.529(12)
C7	C8	1.522(12)	C34	C35	1.526(12)

Table 25 Bond Lengths for 2509056lt_2_twin1.

Atom	Atom	Length/Å	Atom	Atom	Length/Å
C8	C9	1.509(12)	C35	C36	1.491(13)
C9	C10	1.523(12)	C36	C37	1.507(13)
C9	O11	1.212(11)	C36	O38	1.212(11)
C12	C13	1.485(13)	C39	C40	1.490(13)
C12	O19	1.228(11)	C39	O46	1.229(10)
C13	C14	1.392(13)	C40	C41	1.378(13)
C13	C18	1.403(12)	C40	C45	1.407(13)
C14	C15	1.389(12)	C41	C42	1.370(14)
C15	C16	1.383(13)	C42	C43	1.401(16)
C16	C17	1.397(14)	C43	C44	1.384(14)
C17	C18	1.370(14)	C44	C45	1.385(13)
C21	C22	1.398(14)	C48	C49	1.381(14)
C21	C26	1.398(13)	C48	C53	1.399(13)
C22	C23	1.405(15)	C49	C50	1.375(13)
C23	C24	1.358(15)	C50	C51	1.389(14)
C24	C25	1.396(14)	C51	C52	1.394(15)
C24	C27	1.535(14)	C51	C54	1.511(14)
C25	C26	1.394(12)	C52	C53	1.387(14)

Table 26 Bond Angles for 2509056lt_2_twin1.

Atom	Atom	Atom	Angle/˚	Atom	Atom	Atom	Angle/˚
C21	S1	C2	100.2(4)	C48	S28	C29	100.1(4)
C3	C2	S1	108.9(6)	C30	C29	S28	111.1(6)

Table 26 Bond Angles for 2509056lt_2_twin1.

Atom	Atom	Atom	Angle/°	Atom	Atom	Atom	Angle/°
C10	C2	S1	111.3(6)	C30	C29	C37	112.8(7)
C10	C2	C3	114.8(7)	C37	C29	S28	111.3(7)
C2	C3	C7	110.2(7)	C29	C30	C34	112.3(7)
O4	C3	C2	108.0(7)	O31	C30	C29	107.1(7)
O4	C3	C7	104.3(7)	O31	C30	C34	104.2(7)
O4	C3	C20	109.2(7)	O31	C30	C47	109.0(7)
C20	C3	C2	113.4(8)	C47	C30	C29	111.4(8)
C20	C3	C7	111.3(8)	C47	C30	C34	112.3(7)
C5	O4	C3	109.7(7)	C32	O31	C30	108.5(6)
O4	C5	C6	104.1(7)	O31	C32	C33	104.5(7)
C7	C6	C5	101.1(7)	C34	C33	C32	100.9(7)
C12	C6	C5	112.4(7)	C34	C33	C39	114.4(7)
C12	C6	C7	115.1(7)	C39	C33	C32	111.1(7)
C6	C7	C3	105.2(6)	C33	C34	C30	104.8(7)
C8	C7	C3	114.8(7)	C33	C34	C35	113.5(7)
C8	C7	C6	113.7(7)	C35	C34	C30	113.5(7)
C9	C8	C7	112.4(7)	C36	C35	C34	113.7(8)
C8	C9	C10	116.0(8)	C35	C36	C37	115.9(8)
O11	C9	C8	123.0(8)	O38	C36	C35	123.1(9)
O11	C9	C10	121.0(8)	O38	C36	C37	120.9(8)
C2	C10	C9	111.4(7)	C36	C37	C29	112.2(8)
C13	C12	C6	119.5(8)	C40	C39	C33	119.5(7)
O19	C12	C6	119.6(8)	O46	C39	C33	119.7(8)
O19	C12	C13	120.9(9)	O46	C39	C40	120.7(8)

Table 26 Bond Angles for 2509056lt_2_twin1.

Atom	Atom	Atom	Angle/°	Atom	Atom	Atom	Angle/°
C14	C13	C12	122.7(8)	C41	C40	C39	118.9(8)
C14	C13	C18	118.1(9)	C41	C40	C45	118.4(9)
C18	C13	C12	119.2(8)	C45	C40	C39	122.6(8)
C15	C14	C13	121.0(9)	C42	C41	C40	122.1(9)
C16	C15	C14	120.2(9)	C41	C42	C43	119.4(10)
C15	C16	C17	119.2(9)	C44	C43	C42	119.7(9)
C18	C17	C16	120.5(9)	C43	C44	C45	120.3(10)
C17	C18	C13	121.0(9)	C44	C45	C40	120.1(9)
C22	C21	S1	119.1(7)	C49	C48	S28	122.2(8)
C26	C21	S1	121.3(8)	C49	C48	C53	118.6(9)
C26	C21	C22	119.6(8)	C53	C48	S28	119.1(8)
C21	C22	C23	118.7(9)	C50	C49	C48	121.3(9)
C24	C23	C22	121.9(10)	C49	C50	C51	121.0(10)
C23	C24	C25	119.4(9)	C50	C51	C52	118.0(9)
C23	C24	C27	121.8(10)	C50	C51	C54	121.1(10)
C25	C24	C27	118.8(9)	C52	C51	C54	120.9(9)
C26	C25	C24	120.1(9)	C53	C52	C51	121.2(9)
C25	C26	C21	120.1(9)	C52	C53	C48	119.9(10)

Table 27 Torsion Angles for 2509056lt_2_twin1.

A	B	C	D	Angle/°	A	B	C	D	Angle/°
S1	C2	C3	O4	-50.5(8)	S28	C29	C30	O31	49.9(8)
S1	C2	C3	C7	-163.9(5)	S28	C29	C30	C34	163.8(6)

Table 27 Torsion Angles for 2509056lt_2_twin1.

A	B	C	D	Angle/˚	A	B	C	D	Angle/˚
S1	C2	C3	C20	70.6(9)	S28	C29	C30	C47	-69.3(9)
S1	C2	C10	C9	-175.7(6)	S28	C29	C37	C36	175.5(6)
S1	C21	C22	C23	-175.7(8)	S28	C48	C49	C50	-177.4(7)
S1	C21	C26	C25	178.1(7)	S28	C48	C53	C52	175.3(8)
C2	S1	C21	C22	-120.8(8)	C29	S28	C48	C49	-60.2(8)
C2	S1	C21	C26	61.5(9)	C29	S28	C48	C53	124.3(8)
C2	C3	O4	C5	-135.4(7)	C29	C30	O31	C32	139.0(7)
C2	C3	C7	C6	108.2(8)	C29	C30	C34	C33	-108.7(8)
C2	C3	C7	C8	-17.7(10)	C29	C30	C34	C35	15.7(10)
C3	C2	C10	C9	60.0(10)	C30	C29	C37	C36	-58.9(10)
C3	O4	C5	C6	36.6(8)	C30	O31	C32	C33	-38.8(9)
C3	C7	C8	C9	53.4(10)	C30	C34	C35	C36	-52.6(10)
O4	C3	C7	C6	-7.5(8)	O31	C30	C34	C33	6.9(9)
O4	C3	C7	C8	-133.4(7)	O31	C30	C34	C35	131.4(7)
O4	C5	C6	C7	-38.9(8)	O31	C32	C33	C34	41.1(9)
O4	C5	C6	C12	-162.2(7)	O31	C32	C33	C39	162.7(7)
C5	C6	C7	C3	27.6(8)	C32	C33	C34	C30	-28.1(8)
C5	C6	C7	C8	154.1(7)	C32	C33	C34	C35	-152.4(7)
C5	C6	C12	C13	-79.3(10)	C32	C33	C39	C40	85.8(10)
C5	C6	C12	O19	100.0(9)	C32	C33	C39	O46	-92.9(10)
C6	C7	C8	C9	-67.9(9)	C33	C34	C35	C36	67.0(10)
C6	C12	C13	C14	-3.8(13)	C33	C39	C40	C41	-179.4(8)
C6	C12	C13	C18	176.3(8)	C33	C39	C40	C45	-1.7(13)
C7	C3	O4	C5	-18.1(8)	C34	C30	O31	C32	19.7(9)

Table 27 Torsion Angles for 2509056lt_2_twin1.

A	B	C	D	Angle/˚	A	B	C	D	Angle/˚
C7	C6	C12	C13	165.6(7)	C34	C33	C39	C40	-160.9(7)
C7	C6	C12	O19	-15.1(11)	C34	C33	C39	O46	20.4(11)
C7	C8	C9	C10	-32.1(10)	C34	C35	C36	C37	32.7(11)
C7	C8	C9	O11	148.0(8)	C34	C35	C36	O38	-148.6(9)
C8	C9	C10	C2	-21.8(10)	C35	C36	C37	C29	21.7(11)
C10	C2	C3	O4	75.1(9)	C37	C29	C30	O31	-75.8(9)
C10	C2	C3	C7	-38.3(10)	C37	C29	C30	C34	38.1(10)
C10	C2	C3	C20	-163.8(8)	C37	C29	C30	C47	165.0(8)
O11	C9	C10	C2	158.1(8)	O38	C36	C37	C29	-157.0(8)
C12	C6	C7	C3	149.0(7)	C39	C33	C34	C30	-147.4(7)
C12	C6	C7	C8	-84.5(9)	C39	C33	C34	C35	88.2(9)
C12	C13	C14	C15	-179.9(8)	C39	C40	C41	C42	177.1(9)
C12	C13	C18	C17	179.2(9)	C39	C40	C45	C44	-178.8(9)
C13	C14	C15	C16	0.8(14)	C40	C41	C42	C43	1.4(15)
C14	C13	C18	C17	-0.8(13)	C41	C40	C45	C44	-1.2(14)
C14	C15	C16	C17	-1.0(14)	C41	C42	C43	C44	-0.2(15)
C15	C16	C17	C18	0.3(15)	C42	C43	C44	C45	-1.7(15)
C16	C17	C18	C13	0.6(15)	C43	C44	C45	C40	2.3(15)
C18	C13	C14	C15	0.0(13)	C45	C40	C41	C42	-0.7(14)
O19	C12	C13	C14	176.9(8)	O46	C39	C40	C41	-0.7(13)
O19	C12	C13	C18	-3.0(13)	O46	C39	C40	C45	177.0(8)
C20	C3	O4	C5	100.9(9)	C47	C30	O31	C32	-100.3(9)
C20	C3	C7	C6	-125.1(8)	C47	C30	C34	C33	124.8(8)
C20	C3	C7	C8	109.0(9)	C47	C30	C34	C35	-110.7(9)

Table 27 Torsion Angles for 2509056lt_2_twin1.

A	B	C	D	Angle/°	A	B	C	D	Angle/°
C21	S1	C2	C3	-157.1(6)	C48	S28	C29	C30	153.7(6)
C21	S1	C2	C10	75.3(7)	C48	S28	C29	C37	-79.8(7)
C21	C22	C23	C24	-3.3(15)	C48	C49	C50	C51	2.6(14)
C22	C21	C26	C25	0.4(14)	C49	C48	C53	C52	-0.5(14)
C22	C23	C24	C25	2.0(15)	C49	C50	C51	C52	-1.0(14)
C22	C23	C24	C27	-179.2(9)	C49	C50	C51	C54	177.5(9)
C23	C24	C25	C26	0.5(14)	C50	C51	C52	C53	-1.3(14)
C24	C25	C26	C21	-1.7(14)	C51	C52	C53	C48	2.0(15)
C26	C21	C22	C23	2.1(14)	C53	C48	C49	C50	-1.8(14)
C27	C24	C25	C26	-178.3(9)	C54	C51	C52	C53	-179.8(9)

Table 28 Hydrogen Atom Coordinates (Å ×10^4) and Isotropic Displacement Parameters (Å ^{2}×10^3) for 2509056lt_2_twin1.

Atom	*x*	*y*	*z*	U(eq)
H2	7624.37	3984.45	9546.32	63
H5A	5882.84	6642.11	7885.44	62
H5B	5438.45	8586.47	8285.7	62
H6	5069.13	5817.33	8975.98	60
H7	6153.57	2413.49	8441.59	57
H8A	5368.87	1188.7	9257.29	63
H8B	6424.27	833.2	9345.25	63
H10A	6178.56	7036.34	9769.23	62
H10B	6816.47	5835.98	10256.76	62
H14	3961.03	8048.55	8728.39	68

Table 28 Hydrogen Atom Coordinates (Å ×10^4) and Isotropic Displacement Parameters (Å ^{2}×10^3) for 2509056lt_2_twin1.

Atom	*x*	*y*	*z*	U(eq)
H15	2580.45	9898.78	8609.51	67
H16	1447.55	8227.49	8018.98	73
H17	1726.94	4730.11	7525.54	73
H18	3103.97	2932.61	7627.39	69
H20A	8180.89	5265.07	8471.91	95
H20B	7423.58	4296.24	8025.55	95
H20C	7814.67	2647.8	8534.63	95
H22	8307.46	10123.14	10588.84	72
H23	9229.49	9322.13	11411.79	78
H25	9994.86	3217.59	10725.85	66
H26	9168.44	4073	9870.96	67
H27A	10887.74	5599.58	11601.84	107
H27B	10083.74	4119.91	11848.56	107
H27C	10173.64	6857.87	11988.92	107
H29	7294.02	3254.54	4604.3	66
H32A	9250.82	7809.63	3300.94	67
H32B	8696.14	5882.33	2925.22	67
H33	9757.91	5023.34	3978.56	60
H34	8551.25	1670.24	3466.17	61
H35A	8455.14	116.26	4379.3	65
H35B	9491.45	441.13	4268.94	65
H37A	8808.41	6246.44	4807.14	67
H37B	8269.05	5026.53	5304.93	67

Table 28 Hydrogen Atom Coordinates (Å ×10^4) and Isotropic Displacement Parameters (Å ^{2}×10^3) for 2509056lt_2_twin1.

Atom	*x*	*y*	*z*	U(eq)
H41	11407.48	1995.69	2602.06	69
H42	12780.28	3656.9	2449.23	78
H43	13222.57	7075.44	2956.18	79
H44	12268.38	8753.63	3606.56	74
H45	10853.43	7173.08	3718.5	70
H47A	7195.55	3699.37	3088.21	99
H47B	6549.4	4653.78	3564.85	99
H47C	6901.14	2007	3595.16	99
H49	5812.28	3274.27	4896.85	67
H50	5084.03	2208.99	5711.33	70
H52	5896.1	8214.37	6491.97	72
H53	6711.2	9170.95	5695.02	76
H54A	5172.22	3135.04	6908.26	105
H54B	4275.67	4060.02	6583.98	105
H54C	4880.81	5817.43	6970.91	105

9. Computational Details:

(Computational Details For Potential energy surface diagram of the rearrangement of gold-π-alkyne to π-allene and a subsequent Claisen rearrangement reaction.)

I. Computational details

All DFT calculations were performed using Orca (version 6.0.1)[1]. Geometry optimization calculations, intrinsic reaction coordinate (IRC) calculations, and geometry optimization for transition states calculations utilized the TPSS[2] functional with D4[3] dispersion correction, along with the def2-TZVPP[4,5] basis set for gold atom and the def2-SVP[4,5] basis set for the other atoms.

Analytical frequencies calculations utilized the B3LYP[6,7] functional with D4[3] dispersion correction along with the def2-TZVPP[4,5] basis set for gold atom and the def2-SVP[4,5] basis set for the other atoms. Single-point calculations utilized the ωB97x-D4[8] functional, along with the def2-TZVPP basis set for all atoms. Solvent effects were accounted for using the SMD[9] method for DCE (1,2-dichloroethane). The free energy of each state in the chemical progress and the free energy differences (relative to A) were defined by the following equation:

$$\mathbf{G_i = E_{sp} + E_{disp} + E_{solv} + E_{thermo} + E_{ZPV} - TS_i} \; ; \; \Delta\mathbf{G_i = G_i - G_{\mathit{A}}}$$

G: Gibbs free energies; SP: single point energies; disp: dispersion corrections; solv: corrections for solvent effect; thermo: thermal corrections; ZPV: zero-point vibrational corrections; TS: entropic energies at 363.15 K; i: each state in the chemical progress.

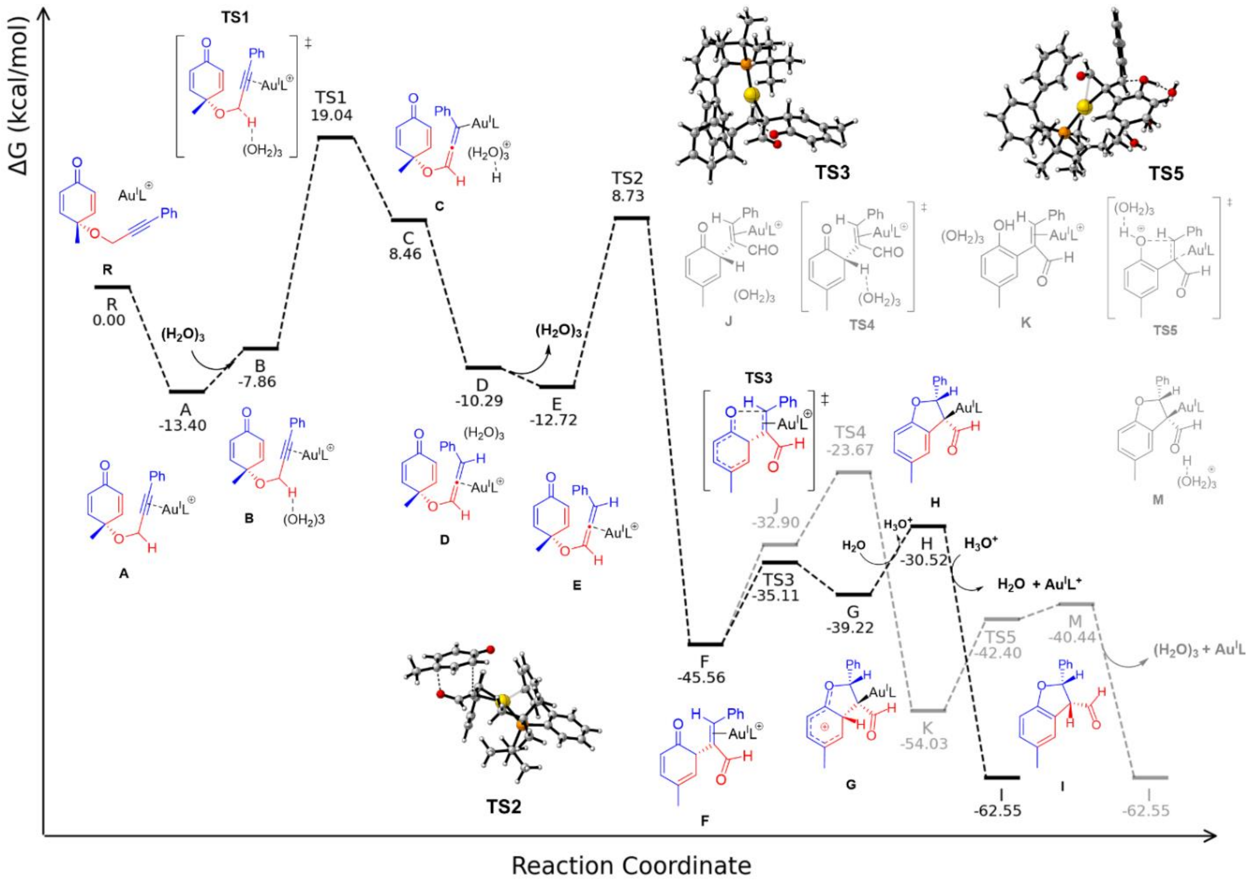

9.1. Figure S1. Potential energy surface diagram of the rearrangement of gold-π-alkyne to π-allene and a subsequent Claisen rearrangement reaction for the system of three water molecules using ωB97X-D4/def2-TZVPP/SMD(DCE)//TPSS-D4/def2-TZVPP/def2-SVP. TS = transition state. L = P(*t*-Bu)$_2$(*o*-biphenyl)).

Systems containing one and two water molecules were calculated (Figure S2, S3). For the one-water system, the key transition state TS1' could not be located; the resulting hydronium-like moiety lacked sufficient stabilization without additional water molecules, preventing the geometry optimization from converging.

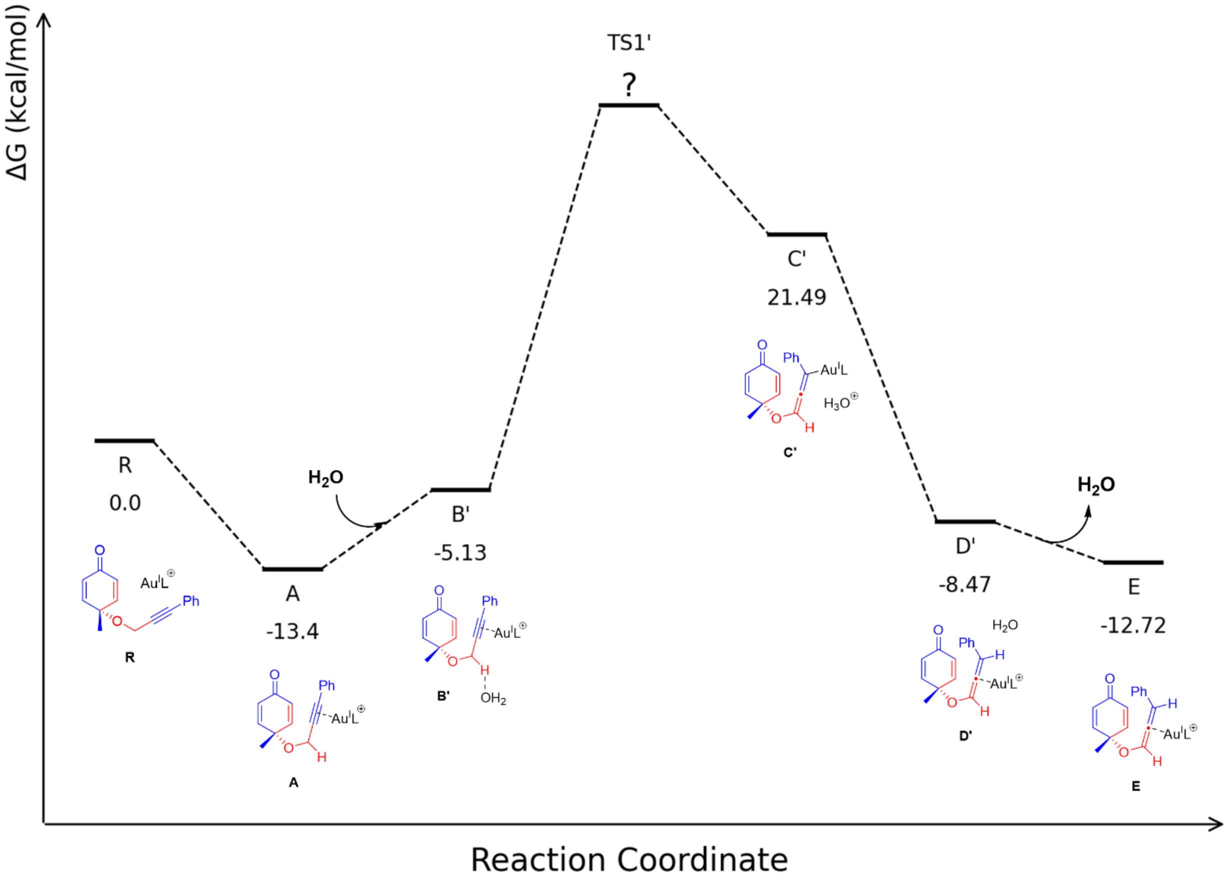

9.2. Figure S2. Potential energy surface diagram of the rearrangement of gold-π-alkyne to π-allene for the system of one water molecule using ωB97X-D4/def2-TZVPP/SMD(DCE)//TPSS-D4/def2-TZVPP/def2-SVP. TS = transition state. L = P(*t*-Bu)$_2$(*o*-biphenyl)).

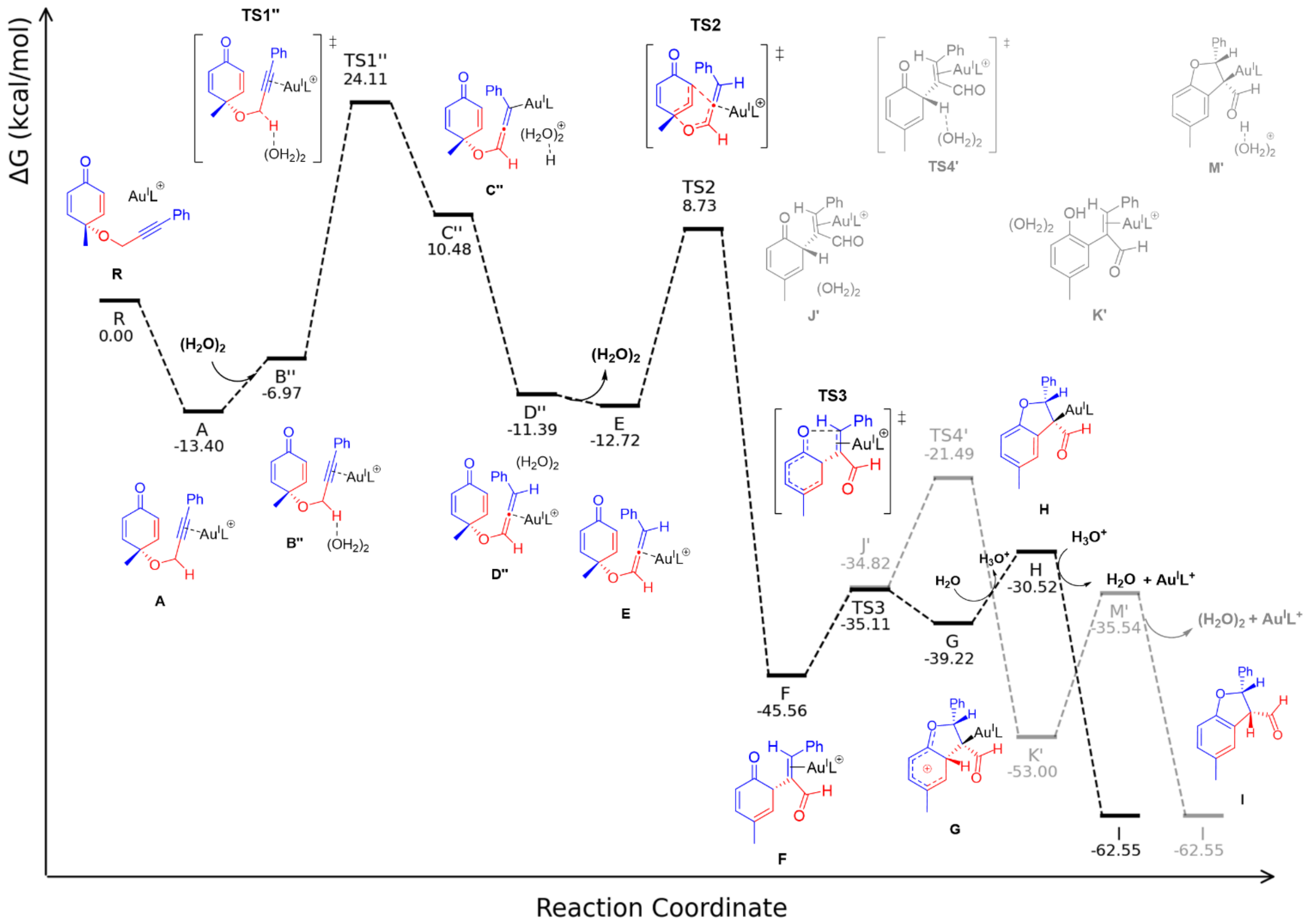

9.3. Figure S3. Potential energy surface diagram of the rearrangement of gold-π-alkyne to π-allene and a subsequent Claisen rearrangement reaction for the system of two water molecules using ωB97X-D4/def2-TZVPP/SMD(DCE)//TPSS-D4/def2-TZVPP/def2-SVP. TS = transition state. L = P(*t*-Bu)$_2$(*o*-biphenyl)).

In the two-water system, the activation Gibbs free energy of TS1" was found to be 4.18 kcal/mol higher than that of the three-water system. Furthermore, most intermediates in the two-water pathway exhibited higher relative energies compared to their three-water counterparts. These results suggest that a minimum of three water molecules is required to adequately simulate the proton transfer process. Consequently, the more energetically favorable three-water system was selected for our primary mechanism, consistent with water cluster models reported in previous work (see manuscript, ref. 10).

II. Computational results

Table S2. Absolute single point energies (SPE), zeroth point vibrational energies (ZPVE), thermal corrections (thermo), entropic energy (TS), enthalpies (H), and Gibbs free energies (G) of. Entropic energy was calculated at 363.15 K. Units are in Eh (Hartree).

State	SPE	ZPVE	thermo	TS	H	G	[a]I.F.
R	-769.105	0.254	0.022	0.078	-768.828	-768.905	N/A
A	-2025.354	0.674	0.058	0.147	-2024.622	-2024.769	N/A
B	-2254.839	0.750	0.069	0.167	-2254.019	-2254.186	N/A
TS1	-2254.791	0.744	0.067	0.164	-2253.980	-2254.144	-1098.36
C	-2254.811	0.747	0.067	0.164	-2253.997	-2254.160	N/A
D	-2254.843	0.750	0.068	0.166	-2254.024	-2254.190	N/A
E	-2025.352	0.673	0.058	0.147	-2024.620	-2024.768	N/A
TS2	-2025.318	0.671	0.058	0.145	-2024.588	-2024.734	-189.55
F	-2025.405	0.674	0.058	0.147	-2024.673	-2024.820	N/A
TS3	-2025.390	0.674	0.057	0.144	-2024.659	-2024.803	-201.33
G	-2025.398	0.676	0.057	0.145	-2024.665	-2024.810	N/A
H	-2024.993	0.664	0.057	0.144	-2024.272	-2024.417	N/A
I	-769.207	0.257	0.021	0.075	-768.929	-769.005	N/A
J	-2254.879	0.750	0.068	0.166	-2254.060	-2254.226	N/A
TS4	-2254.861	0.746	0.067	0.163	-2254.048	-2254.212	-1348.63
K	-2254.914	0.752	0.068	0.166	-2254.094	-2254.260	N/A
TS5	-2254.894	0.749	0.067	0.163	-2254.079	-2254.241	-332.71
M	-2254.894	0.751	0.066	0.161	-2254.078	-2251.238	N/A
*AuL^+	-1256.194	0.418	0.034	0.099	-1255.743	-1255.842	N/A
H_2O	-76.485	0.021	0.003	0.027	-76.460	-76.488	N/A
H_3O^+	-76.877	0.034	0.004	0.028	-76.839	-76.867	N/A
$(H_2O)_2$	-152.970	0.047	0.006	0.039	-152.917	-152.955	N/A
$(H_2O)_3$	-229.460	0.072	0.010	0.049	-229.377	-229.426	N/A
B’	-2101.851	0.699	0.062	0.154	-2101.089	-2101.243	N/A
C’	-2101.807	0.697	0.060	0.149	-2101.052	-2101.201	N/A
D’	-2101.855	0.699	0.062	0.154	-2101.094	-2101.249	N/A
B’’	-2178.343	0.724	0.066	0.161	-2177.553	-2177.714	N/A
TS1’’	-2178.290	0.719	0.064	0.157	-2177.507	-2177.664	-350.22
C’’	-2178.315	0.722	0.064	0.156	-2177.530	-2177.686	N/A
D’’	-2178.351	0.724	0.065	0.160	-2177.561	-2177.721	N/A
J’	-2178.389	0.725	0.065	0.159	-2177.599	-2177.758	N/A
TS4’	-2178.364	0.719	0.063	0.156	-2177.581	-2177.737	-957.84
K’	-2178.420	0.727	0.065	0.159	-2177.629	-2177.787	N/A
M’	-2178.393	0.725	0.063	0.154	-2177.605	-2177.759	N/A

*L = P(*t*-Bu)$_2$(*o*-biphenyl); a: I.F. = Imaginary frequencies, units are in cm^{-1}.

Table S3. Relative enthalpies (ΔH) and Gibbs free energies (ΔG). Numbers are referenced to A. Units are in kcal/mol

State	ΔH	ΔG
R	0	0
A	-31.99	-13.4
B	-44.82	-7.86
TS1	-19.92	19.04
C	-30.53	8.46
D	-47.71	-10.29
E	-31.32	-12.72
TS2	-11.03	8.73
F	-64.27	-45.56
TS3	-55.55	-35.11
G	-59.56	-39.22
H	-50.65	-30.52
I	-63.87	-62.55
J	-70.46	-32.90
TS4	-62.85	-23.67
K	-91.85	-54.03
TS5	-81.99	-42.40
M	-81.35	-40.44
B’	-36.67	-5.13
C’	-13.08	21.49
D’	-39.64	-8.47
B”	-41.31	-6.97
TS1”	-12.34	24.11
C”	-26.70	10.48
D”	-46.31	-11.39
J’	-70.10	-34.82
TS4’	-58.66	-21.47
K’	-88.43	-53.00
M’	-73.87	-35.54

III. References

(1) Neese, F. Software Update: The ORCA Program System—Version 5.0. *WIREs Comput. Mol. Sci.* **2022**, *12* (5), e1606.

(2) Tao, J.; Perdew, J. P.; Staroverov, V. N.; Scuseria, G. E. Climbing the Density Functional Ladder: Nonempirical Meta–Generalized Gradient Approximation Designed for Molecules and Solids. *Phys. Rev. Lett.* **2003**, *91* (14), 146401.

(3) Caldeweyher, E.; Bannwarth, C.; Grimme, S. Extension of the D3 Dispersion Coefficient Model. *J. Chem. Phys.* **2017**, *147* (3), 034112.

(4) Weigend, F.; Ahlrichs, R. Balanced Basis Sets of Split Valence, Triple Zeta Valence and Quadruple Zeta Valence Quality for H to Rn: Design and Assessment of Accuracy. *Phys. Chem. Chem. Phys.* **2005**, *7* (18), 3297.

(5) Weigend, F.; Häser, M.; Patzelt, H.; Ahlrichs, R. RI-MP2: Optimized Auxiliary Basis Sets and Demonstration of Efficiency. *Chem. Phys. Lett.* **1998**, *294* (1–3), 143–152.
(6) Becke, A. D. Density-Functional Exchange-Energy Approximation with Correct Asymptotic Behavior. *Phys. Rev. A* **1988**, *38* (6), 3098–3100.
(7) Lee, C.; Yang, W.; Parr, R. G. Development of the Colle-Salvetti Correlation-Energy Formula into a Functional of the Electron Density. *Phys. Rev. B* **1988**, *37* (2), 785–789.
(8) Chai, J.-D.; Head-Gordon, M. Systematic Optimization of Long-Range Corrected Hybrid Density Functionals. *J. Chem. Phys.* **2008**, *128* (8), 084106.
(9) Marenich, A. V.; Cramer, C. J.; Truhlar, D. G. Universal Solvation Model Based on Solute Electron Density and on a Continuum Model of the Solvent Defined by the Bulk Dielectric Constant and Atomic Surface Tensions. *J. Phys. Chem. B* **2009**, *113* (18), 6378–6396.

IV. Cartesian Coordinates

A

C	-6.57089116576685	6.99377117391616	-5.11350315013972
C	-7.61622450329214	7.38714241696095	-6.08822038321046
C	-6.81265890888795	5.73023681063267	-4.37140031705918
C	-8.70640513030055	6.62566593972748	-6.30753161780860
C	-8.99833741183669	5.34742587277517	-5.56058656924538
C	-7.89923645742523	4.96497516138934	-4.59883047893230
C	-10.33393399662548	5.45883706407806	-4.79487531045464
H	-10.27366964712918	6.24417108611006	-4.02347106044156
H	-10.57265550818725	4.50196920829993	-4.29770711845283
H	-11.14516994982719	5.70322007001443	-5.49984075273920
O	-5.54302459089156	7.65157554955043	-4.95212668801858
O	-9.18637566452728	4.36916341192126	-6.64783123859716
C	-8.95920676958855	3.00546100278323	-6.38610066368654
C	-7.49715299372160	2.69341654345770	-6.45285394117981
H	-9.49756352285198	2.44833682025018	-7.16980714344930
H	-9.35614344174949	2.66683447466021	-5.40770423686436
C	-6.26937018591675	2.95267830763573	-6.36070215181941
H	-7.43827160027698	8.31495399101695	-6.64167560685274

H	-9.45336660087097	6.90319021308925	-7.06107651825491
H	-6.05039560153541	5.45036845726599	-3.63708389927822
H	-8.04726048833815	4.04079068132541	-4.02772897147580
C	-4.97248662968319	3.54778230887270	-6.26421233076589
C	-4.78723848939898	4.83250152529379	-6.82968318842001
C	-3.56421769860301	5.48760105477777	-6.66891276778817
C	-2.51389066087721	4.86096643749042	-5.98111980930760
C	-3.91056641281645	2.91718687095775	-5.57513216652113
C	-2.68542494734356	3.57467447149841	-5.44349265357446
H	-5.61252395688044	5.30790016580613	-7.36646342501415
H	-3.43429440350101	6.49536521164833	-7.07344974437165
H	-1.55727502249600	5.37889349043887	-5.86056909830833
H	-4.06668939270451	1.92498358697144	-5.14366916062492
H	-1.86222126112288	3.08990011287541	-4.90937619875470
Au	-6.53621082381512	0.80581216023217	-6.90858754382892
P	-5.83530621403781	-1.28238454810352	-7.66397589831029
C	-7.14771829306735	-1.89262650450085	-8.89431769774077
C	-7.20624965245356	-0.89881680840813	-10.07171997171033
C	-6.93252464017918	-3.32631675300929	-9.41455320158421
C	-8.47321293189722	-1.84894771081870	-8.10078122615240
C	-4.08537714435710	-1.03336855531624	-8.39033162966603
C	-3.13705830590443	-0.99654814081301	-7.17155605246902
C	-3.64528561311206	-2.12458599499738	-9.38300275890627
C	-4.04276265340781	0.34155476531544	-9.09533875751547
C	-5.69384402954686	-2.55803930163043	-6.35525949126276
C	-5.18462062835269	-3.82964623349601	-6.70512916394880
C	-4.99577429281555	-4.82951200637726	-5.74745208775062
C	-5.31163956996517	-4.57114574476709	-4.40780818143453

C	-5.81213353264836	-3.31727431113248	-4.04459285165073
C	-6.01310721562234	-2.29649283032022	-4.99699603257908
C	-6.55085224594480	-1.00330478975727	-4.46981306233813
C	-5.68959252104552	-0.09368768929827	-3.82150409469288
C	-6.20062832735103	1.05512046510332	-3.20115754865624
C	-7.58073388481243	1.30138741503097	-3.20576232500004
C	-8.44707183525347	0.40303371205688	-3.84578869403032
C	-7.93660652761703	-0.74055743827138	-4.47825437255363
H	-6.31377865055165	-0.96671565567766	-10.71557032347331
H	-7.30837265303741	0.14412207722778	-9.72114925719727
H	-8.08557205597991	-1.13701981353405	-10.69635349637793
H	-5.97424922731855	-3.45018005247110	-9.94177839103678
H	-7.73850098322363	-3.55424439534881	-10.13530518907815
H	-6.99605156050434	-4.06994887197887	-8.60473016648104
H	-8.72222740071632	-0.82427245620109	-7.76862540875963
H	-8.43470657974676	-2.50591824779635	-7.21457701094208
H	-9.29323011030251	-2.20396343755755	-8.75048833645525
H	-3.06083763137437	-1.97625287411044	-6.67350017977414
H	-3.47072693675861	-0.25024355565482	-6.42827615799029
H	-2.12892214517359	-0.70227788380377	-7.51427130392313
H	-3.63579729585282	-3.13066247959691	-8.93561619891104
H	-2.61210128681082	-1.90321091521733	-9.70625742012813
H	-4.27547273610115	-2.14083315225740	-10.28695220433673
H	-4.25787755669017	1.16478757960526	-8.39160095384605
H	-4.75173799515332	0.41126340805266	-9.93499661068492
H	-3.02607946950003	0.49711890144820	-9.49860045129826
H	-4.92221354170013	-4.04384082222938	-7.74265313180297
H	-4.60007366930907	-5.80426025094479	-6.04885704365587

H	-5.16785738517618	-5.34342832562503	-3.64559482064321
H	-6.06109011654823	-3.11063265902527	-2.99893413150705
H	-4.61585444952642	-0.30628922814143	-3.78850534818796
H	-5.52166134316280	1.74806932162613	-2.69400513319080
H	-7.98228075305127	2.17503262281095	-2.68213185572611
H	-9.52887459165319	0.57303761298717	-3.82948880648044
H	-8.61824797689318	-1.45581909679930	-4.94799966291596

B

C	-8.15958505467673	6.26115595452341	-6.45685174362143
C	-7.55037997368839	5.25369501241757	-7.34354466565333
C	-9.57425164858524	6.06700463984806	-6.08368997501294
C	-8.21779123606262	4.12981302028765	-7.67976727951235
C	-9.65506980760741	3.86348648372614	-7.29412430321217
C	-10.25762147545147	4.97022281178086	-6.46715645184632
C	-10.49167244171402	3.62913820017383	-8.56582409972888
H	-10.48258055193067	4.52466967674954	-9.20762014428415
H	-11.52962885631029	3.39166380480807	-8.28131585349790
H	-10.07528284212543	2.77649707778259	-9.12720518519508
O	-7.48786184793288	7.20866431182898	-6.01395676001269
O	-9.76510648073655	2.59872339548476	-6.56925742745141
C	-9.29571082518826	2.60394793490334	-5.21775158986521
C	-7.82448723608543	2.64860329093153	-5.10738056930918
H	-9.69340469954564	1.68156665530823	-4.76570784038230
H	-9.64000988749801	3.48449768519595	-4.63157101586844
C	-6.63032412607353	3.00414126939408	-4.95149937308993
H	-6.51138900085466	5.42940349475411	-7.63923840567896
H	-7.73328780889044	3.34785790664118	-8.27707452623592

H	-10.02899738929173	6.83775113886503	-5.45498154559120
H	-11.30058466997067	4.82147935328561	-6.16147730515016
C	-5.51282696226382	3.84277902356962	-4.62702097689031
C	-5.82065286718498	5.09600281268754	-4.04582158759973
C	-4.79188218097107	5.98791356792860	-3.73494721633026
C	-3.45606505839367	5.63991644611238	-3.99624219211708
C	-4.17125504018276	3.49410277643134	-4.88936737672301
C	-3.14974439431829	4.39520789010977	-4.57101101731396
H	-6.86844523128299	5.33277011033439	-3.84509110199713
H	-5.04894715356558	6.96085022894334	-3.30420804445029
H	-2.65018915259475	6.34126634732523	-3.75705008824338
H	-3.94296475993261	2.52234701114449	-5.33804849956522
H	-2.10763742335298	4.12914076909039	-4.77442484278427
Au	-6.58870501251196	1.10730074038003	-6.06457075963075
P	-6.08768029762573	-0.74441144893242	-7.37718408081626
C	-7.69821713171320	-1.21721891835336	-8.27106408118049
C	-8.12547772122076	-0.03342190624464	-9.16126159347943
C	-7.61494481260003	-2.51222242246527	-9.10015286532989
C	-8.72819806450210	-1.40966807310762	-7.13466854196566
C	-4.62988671490924	-0.24764242699501	-8.50022960767504
C	-3.38218705114686	-0.32698845213655	-7.59226877676011
C	-4.44192549446783	-1.12424221910402	-9.75153442481094
C	-4.83791363610857	1.22084642217793	-8.93674818536415
C	-5.57936931348947	-2.22706774994911	-6.42614686672753
C	-5.18051617938695	-3.38389156495701	-7.13370375462543
C	-4.80877887890885	-4.55391497913046	-6.46616105413835
C	-4.83616768556302	-4.58881183139388	-5.06626136631512
C	-5.22071579341903	-3.44973345103472	-4.35216225264886

C	-5.58961201491459	-2.25622694880914	-5.00620350568793
C	-5.93977256958859	-1.09248707965881	-4.13349838649779
C	-4.93208261525171	-0.19789604396727	-3.71363421802507
C	-5.23186463512874	0.84214361561495	-2.82141834197449
C	-6.53778307794463	1.00826011950716	-2.33765716287822
C	-7.54013852909389	0.11344680551768	-2.73965857170836
C	-7.24563380686650	-0.93409541488584	-3.62534289255040
H	-7.45950759808810	0.09179694485070	-10.03106700605265
H	-8.15455467000172	0.90837113398080	-8.58559702605654
H	-9.14321482404861	-0.22615964869833	-9.54538252724758
H	-6.83778984164375	-2.47032345297385	-9.87891415134490
H	-8.58591486275633	-2.66066728530396	-9.60647409109468
H	-7.43734018765418	-3.39377047245657	-8.46413378281930
H	-8.88077493521985	-0.47508559666808	-6.56582598445967
H	-8.41451385248016	-2.20373878210465	-6.43526572187874
H	-9.69741891018042	-1.70431874954629	-7.57534962811536
H	-3.14309580048460	-1.36448773534331	-7.30813615285906
H	-3.52388427253051	0.26115941861453	-6.66657648682026
H	-2.51610719552860	0.09380970011981	-8.13398214951167
H	-4.26665146379072	-2.18308304398523	-9.50556876264869
H	-3.55133148933361	-0.76628551573460	-10.29912937723178
H	-5.30081565380980	-1.05432513037779	-10.43891664289901
H	-4.90058474925632	1.89750521836757	-8.06560492212699
H	-5.74943703498528	1.35463311958012	-9.54015300194389
H	-3.97570059816660	1.53239129323988	-9.55334078456415
H	-5.15941044383343	-3.37409118543783	-8.22509067119222
H	-4.50264011333734	-5.43433370556266	-7.03956158989472
H	-4.55300695157583	-5.49990539568707	-4.52966658596919

H	-5.23313065210746	-3.46851624634549	-3.25786622164891
H	-3.90897244440441	-0.33353675772970	-4.08000007468564
H	-4.44017216148801	1.52898626027838	-2.50591537220430
H	-6.77386810544034	1.84342296435091	-1.67012409323156
H	-8.55974970690614	0.22763882444062	-2.35658956273327
H	-8.02778803518816	-1.63943147018629	-3.92358868633594
O	-9.02025168704749	4.98364240678547	-3.22963461359792
H	-8.51147066609164	4.62778609863703	-2.46534606004340
H	-8.82003622977016	5.95356526914108	-3.20605474462500
O	-7.22608279134521	4.08848413339800	-1.20649799859322
H	-6.40882718605839	4.54367448480558	-1.48751937463121
H	-7.38596897749901	4.41839876303063	-0.30288886046551
O	-8.03022053628049	7.55576988228878	-3.38449249035550
H	-7.84684712600086	7.64877163399520	-4.35767167291027
H	-8.45742912503864	8.39100574979584	-3.12686283220066

TS1

C	-8.23097955985853	6.55899925257371	-6.43231047722281
C	-7.64834179127561	5.51512725847609	-7.28620516018371
C	-9.61151858170689	6.35548158651358	-5.95118950135184
C	-8.26777865700944	4.32107269302464	-7.43357924839169
C	-9.64610285369007	4.02680411682454	-6.89294851238332
C	-10.25595940627503	5.18831006009135	-6.15441880526961
C	-10.56560252513549	3.58644246342419	-8.04836527200896
H	-10.68478087668635	4.39652601735348	-8.78555914152763
H	-11.55226827561754	3.31186851646006	-7.64116159495053
H	-10.12874097964350	2.70269273428706	-8.54185174881895
O	-7.55533046354374	7.53719118622289	-6.05429023741650

O	-9.64089340418896	2.88266742267166	-5.99694630826046
C	-8.73645234509144	2.90288836467152	-4.89703477161814
C	-7.37732376556224	2.89841987583293	-5.11824180713579
H	-9.12443975742714	2.20913526173133	-4.13535256989561
H	-8.71343602891630	4.10181497742245	-4.01399043417574
C	-6.09572263143330	2.95309442990538	-5.26119334745698
H	-6.65017193079158	5.70890983899838	-7.69021532773285
H	-7.78663547187578	3.50435901366909	-7.98434683828727
H	-10.06641563585055	7.17410606232347	-5.38493766734870
H	-11.26332500306184	5.01697273194532	-5.75629591227793
C	-5.06320437092165	3.95559478682213	-5.02822473437871
C	-5.42607207387600	5.27393118536796	-4.65739344358528
C	-4.44761579638599	6.24767125558889	-4.43209278960076
C	-3.08897182374596	5.91986340527644	-4.57208596967579
C	-3.69431518471197	3.64106487085493	-5.16896474659266
C	-2.71721708899526	4.61667496222508	-4.93977386688871
H	-6.48186834472411	5.52722784578673	-4.55113588617231
H	-4.75348308472736	7.26277052756909	-4.16010030804489
H	-2.32092748612938	6.68075048451310	-4.40047558184204
H	-3.41491347969202	2.62264282107216	-5.45784670858719
H	-1.65820256830521	4.36250027269985	-5.05089040252543
Au	-5.93253618237804	1.10089626629228	-6.23013281359459
P	-5.85118855657486	-0.84770866688134	-7.48764014849178
C	-7.50086283142358	-0.95170946857574	-8.43548386753690
C	-7.56415732296581	0.23423813327890	-9.41876790813725
C	-7.74947123940290	-2.27226894840505	-9.18711022963824
C	-8.58716871479937	-0.78189723685570	-7.34763406566108
C	-4.29310861670308	-0.78466000278286	-8.58397418759959

C	-3.12263500815971	-1.05545216398977	-7.61258126471522
C	-4.26134263466974	-1.78448181319855	-9.75347198918134
C	-4.14961506126555	0.65028728433227	-9.14018267468908
C	-5.74652307338319	-2.38942566912246	-6.49079247699316
C	-5.66892635615249	-3.63416845848699	-7.15583421607399
C	-5.62571375477719	-4.84027960252678	-6.45089010753206
C	-5.66945483022515	-4.82454415031189	-5.05143347876136
C	-5.73792437465541	-3.60198974492513	-4.37697946928450
C	-5.76659391024514	-2.37391031079373	-5.07012554536415
C	-5.78770374938990	-1.13986361321860	-4.22481140450304
C	-4.58455188139822	-0.46913203229966	-3.91545420496885
C	-4.58401731862168	0.62273921886992	-3.03564481950440
C	-5.78114377838825	1.05597734825789	-2.44810693607588
C	-6.97741334216845	0.38661368682861	-2.74061445485059
C	-6.98373431657299	-0.70588723139086	-3.61908633594899
H	-6.89586345406184	0.08810525492532	-10.28349994593433
H	-7.29640320811213	1.18414132214454	-8.92210658366106
H	-8.59524477535891	0.32535452574905	-9.80612556709240
H	-6.96510646752527	-2.49736447090458	-9.92711878102159
H	-8.70543031506066	-2.18337544231222	-9.73495685896665
H	-7.84495378629806	-3.12479501065017	-8.49634962544256
H	-8.53716045608010	0.21197304334649	-6.86902208653497
H	-8.49921834590530	-1.55639165970570	-6.56570489564166
H	-9.58252234127164	-0.88610032675529	-7.81680898225657
H	-3.15313785880085	-2.07794985084760	-7.20162943090256
H	-3.13509058361625	-0.33860897386688	-6.77141656839055
H	-2.16797545838937	-0.92782503425256	-8.15412618649693
H	-4.30053876526217	-2.83258879533209	-9.41816608213352

H	-3.30913379827236	-1.65390977341611	-10.29928403128341
H	-5.07841964513152	-1.60749505205064	-10.47180534164301
H	-4.16753958476092	1.40010897867792	-8.32882791532099
H	-4.94339195563197	0.90397640297009	-9.85952892557473
H	-3.18102066687303	0.73031123971191	-9.66606201972791
H	-5.64571423579247	-3.66787106229976	-8.24607710445963
H	-5.56288480801662	-5.78726892835697	-6.99600551800699
H	-5.64296713688359	-5.76093438547978	-4.48505265120890
H	-5.75730292050053	-3.58173945758688	-3.28271170018821
H	-3.64644274473086	-0.82252387886807	-4.35505737206442
H	-3.64280584746582	1.13305672461215	-2.80653628566110
H	-5.78972939207548	1.91903686699845	-1.77661480341051
H	-7.91321841859731	0.71095528051429	-2.27275009861107
H	-7.91852178943712	-1.23187264876301	-3.83789588053354
O	-8.68223018300781	4.88878075608290	-3.08805790303928
H	-7.85871824807223	4.64805862663219	-2.55044558462592
H	-8.54649713607938	5.87343229775745	-3.32682506296936
O	-6.40122177065151	4.33637823674765	-1.85132190811485
H	-5.70921317027187	4.51805918763023	-2.52451356209071
H	-6.16110911105508	4.89677874751782	-1.08981425306996
O	-8.03204128493781	7.38930419048599	-3.45770753550786
H	-7.86898580533083	7.69398295859538	-4.39879145158155
H	-8.54318642949193	8.09881100405132	-3.03000977610419

C

C	-7.74541064606022	6.16341209300013	-7.10854378233858
C	-7.69552210772103	4.87840428755175	-7.79145137345712
C	-8.83864006209126	6.40721887058865	-6.16598702613219

C	-8.56486503837208	3.88601298555994	-7.47027917499294
C	-9.76655340766739	4.11032882366672	-6.59246936943940
C	-9.72188384159913	5.42877925387463	-5.86515238383542
C	-11.02732435050045	4.09789090068448	-7.50060421226745
H	-10.98213494618395	4.91021896151616	-8.24197186321126
H	-11.92133482463777	4.22044757474492	-6.86835530577587
H	-11.08896715364193	3.12562389946953	-8.01554242613481
O	-6.84566829220932	7.03942232528145	-7.25398687826956
O	-9.99903360322940	3.03662508493111	-5.66206491103229
C	-8.92633731089777	2.64211702307369	-4.86944711322578
C	-7.64385520223365	2.87141327281244	-5.09899849996810
H	-9.28854021752383	2.06087503815248	-4.01348807919630
H	-5.12623664302889	3.10483990447398	-7.64975003794950
C	-6.33720928453993	2.90081279790194	-5.34297172873043
H	-6.88768811556228	4.69909208558867	-8.50592555131321
H	-8.48500039487450	2.89896173760621	-7.93932874879608
H	-8.84813376677304	7.37951136483900	-5.66381462656151
H	-10.49916441100053	5.57630058573891	-5.10607772747858
C	-5.45647890449545	4.01486082473224	-4.95380683298205
C	-5.97840790997996	5.24504579226695	-4.49077934992541
C	-5.13635838495440	6.33068507744675	-4.23089357323055
C	-3.74531779722984	6.20647951424632	-4.41125705978687
C	-4.05603056541528	3.89641594742770	-5.09770747904555
C	-3.20582828396834	4.97539175962549	-4.83137548956607
H	-7.06030871492582	5.33548173130794	-4.35362704233323
H	-5.55948688022622	7.28248756286020	-3.89394628855177
H	-3.08518066314571	7.05588939624183	-4.20713261786923
H	-3.65626975945640	2.93211627793078	-5.42974632298237

H	-2.12432393329854	4.86668956871703	-4.96251780055501
Au	-5.77149985655089	1.21100619099425	-6.39375570001761
P	-5.66357587954977	-0.80784000917427	-7.59683520210552
C	-7.33329476610772	-0.85754185318157	-8.52425869012165
C	-7.34431312968315	0.31099332487547	-9.53078035219330
C	-7.67269186030557	-2.17802181282256	-9.24006171265028
C	-8.39613043911475	-0.61388776474332	-7.42639814788578
C	-4.14361954530535	-0.97186627234317	-8.73628781379070
C	-2.98532254989904	-1.43743702323494	-7.82748518706145
C	-4.30275716359333	-1.92825443168794	-9.93218526339122
C	-3.81253796662002	0.43866620128375	-9.27004027295488
C	-5.69142741026718	-2.30244957366229	-6.52287496412866
C	-5.69767402527217	-3.58164501622078	-7.12405834841731
C	-5.82158048171887	-4.74894629396212	-6.36554636043099
C	-5.95994284457171	-4.65659409800208	-4.97495359721638
C	-5.94011294731106	-3.40085681433080	-4.36191985446696
C	-5.78898891249176	-2.21253724232087	-5.10852707043597
C	-5.69702919526532	-0.94951797514152	-4.31505992079849
C	-4.44816491749946	-0.31595072143253	-4.12611134675658
C	-4.32757220347688	0.77705754019446	-3.25622383750077
C	-5.45086966960631	1.25880580292197	-2.57065964226392
C	-6.69382719229154	0.63853703797420	-2.75317510806563
C	-6.81694419922644	-0.46158589339685	-3.61289690941572
H	-6.68011761397035	0.12336763563414	-10.39138220452722
H	-7.03291950304726	1.25604802792148	-9.04936244891444
H	-8.36851796610668	0.44156358275938	-9.92550172653437
H	-6.92847342283793	-2.45953308929662	-10.00010338613232
H	-8.64176913929047	-2.05152342665847	-9.75699242295443

H	-7.78822077499795	-3.00994532836608	-8.52748773843921
H	-8.25444757906567	0.35750912888496	-6.91862317431700
H	-8.36885912448582	-1.40816246234887	-6.66039611835209
H	-9.39973477819737	-0.62222074476219	-7.88931104458007
H	-3.12236592945058	-2.47174440991361	-7.47320154330359
H	-2.88873082276674	-0.78147098241038	-6.94306524180213
H	-2.03781763815198	-1.39058134383032	-8.39538372010625
H	-4.53217380152237	-2.96118442033052	-9.62836969943355
H	-3.34790658603229	-1.95823067483021	-10.48860395830513
H	-5.08056699928203	-1.58389847627296	-10.63267093713244
H	-3.52295995379188	1.11603708781137	-8.45152876234601
H	-4.65325032255992	0.90194538575624	-9.80957189508870
H	-2.96106569917084	0.36393380170206	-9.97243173309159
H	-5.61195258242586	-3.67204002265048	-8.20836543858598
H	-5.81919246006019	-5.72427275954340	-6.86211035494701
H	-6.06998826917482	-5.56023184815346	-4.36701068748779
H	-6.01966144636173	-3.32271953887596	-3.27297894734544
H	-3.56487437217239	-0.71549138390566	-4.63444637574243
H	-3.35057448233361	1.24779598954744	-3.10662256040114
H	-5.35638232410217	2.11392996275624	-1.89450810240290
H	-7.57239420477824	1.00730331305929	-2.21398238032266
H	-7.78684169607694	-0.95200959180357	-3.74445362641973
O	-4.60654575013470	3.73876634515739	-8.21709155662208
H	-4.61247126468532	5.10179754059575	-7.80026460032731
H	-3.66081782036070	3.43607270654613	-8.11148006939115
O	-4.57534525481586	6.14598799068513	-7.55121445730784
H	-4.22076926906230	6.21243894404246	-6.63253867657311
H	-5.58691793688536	6.56749023405104	-7.52901501305300

O	-2.01681332502634	3.09599426400116	-7.76495737185534
H	-1.82633915129276	2.34621387533787	-7.17190967608234
H	-1.44424816234798	2.94062906125602	-8.53943637076585

D

C	-8.93340886994701	4.05275574683464	-8.90507935314247
C	-9.18065105332207	3.16132209777515	-7.75164997853210
C	-8.27700468061992	5.34824222015361	-8.62523601643000
C	-8.71319946860311	3.45333167847637	-6.52189945319874
C	-7.98648771603141	4.73389623485996	-6.20759524986688
C	-7.83668361545386	5.65707934325439	-7.38869519945292
C	-8.61298254278443	5.45064218788624	-5.00687097545043
H	-9.64065512519584	5.76423896010758	-5.25053929797594
H	-8.01617128019132	6.34044351055018	-4.74762259623363
H	-8.63870522837061	4.77356782416535	-4.13749433453597
O	-9.24335940985935	3.70667056069657	-10.05192946221583
O	-6.62064530809250	4.35151119527478	-5.71580701188683
C	-5.76019805780626	3.91551587786453	-6.64311720946477
C	-4.61075519840697	3.23606679784505	-6.29310611680342
C	-3.37625891332068	3.30832040574949	-5.75647411259963
H	-9.72109737336745	2.23471568506034	-7.96074090138820
H	-8.84812813050763	2.76167719946566	-5.68256157274606
H	-8.14312336934777	6.02586142987204	-9.47351936843547
H	-7.32810614865512	6.60885673034322	-7.19165429135017
C	-2.65341861306318	4.53584411203990	-5.40011156970398
C	-3.22250688015297	5.82502995030336	-5.51396482814856
C	-2.48747280481744	6.95644922996630	-5.15532963114232
C	-1.17206228559796	6.82530091124534	-4.67640072946092

C	-1.33055992188297	4.41753571050614	-4.91642677283409
C	-0.59621156173724	5.55278265137225	-4.55638195759430
H	-2.93875822883112	7.94990157202978	-5.24526191807959
H	-0.60032947258402	7.71574788622909	-4.39627189009119
H	-0.88185773572210	3.42195546941703	-4.82413255995466
H	0.42666053877467	5.44500133933950	-4.18181126288744
Au	-5.49980223788881	1.47360685948746	-7.06626557229943
P	-5.87100662604661	-0.62795858400832	-8.02447666978235
C	-7.58153599284179	-0.66118073554195	-8.85900109945137
C	-7.73927413195685	0.62645589107301	-9.69490379112511
C	-7.85479120788859	-1.88860869590672	-9.74529782249628
C	-8.58084173135058	-0.62730216593423	-7.68182545606790
C	-4.39919698220275	-0.89236823917071	-9.20042769151650
C	-3.15445865570099	-0.53797514267446	-8.35282182335715
C	-4.23578513834803	-2.32186954062907	-9.74768749599454
C	-4.54640365610223	0.10670790418396	-10.36731235173828
C	-5.82778267287106	-2.00500081122753	-6.80655597932369
C	-5.96081057239486	-3.32709004934549	-7.28977834231932
C	-5.85993412973492	-4.43336006907268	-6.44163722491134
C	-5.60586595037867	-4.23683034284532	-5.07906091997704
C	-5.48551231857338	-2.93636943405861	-4.58060045468908
C	-5.60922244288817	-1.80578028661882	-5.41581299980747
C	-5.54117852908381	-0.47870541042280	-4.73188957912623
C	-4.33034612351759	-0.02439520340079	-4.17282496250956
C	-4.29259008119944	1.15788197184448	-3.41927082303302
C	-5.46250753431926	1.89944079784196	-3.20693862415090
C	-6.67498817259716	1.44785434986570	-3.74490097047856
C	-6.71685532426077	0.26927301816239	-4.50298484615717

H	-7.21514821749361	0.57736029137549	-10.65959141984867
H	-7.35854870548197	1.51336202300374	-9.16008685081183
H	-8.81020937509937	0.78454502380496	-9.91363665132205
H	-7.13127380914083	-1.97182365777376	-10.57267305420009
H	-8.85638985759862	-1.77028212950777	-10.19770651105280
H	-7.86396358421778	-2.83098578572791	-9.17543953574039
H	-8.41636310077623	0.26604678179082	-7.05439233482965
H	-8.50317758070714	-1.52393146286192	-7.04407747442674
H	-9.60834704937422	-0.58103907048694	-8.08600561402330
H	-3.11826706551974	-1.12030163173178	-7.41469464165190
H	-3.13037162060263	0.53913157966649	-8.11490093004893
H	-2.24644032676743	-0.77448597066678	-8.93646533082891
H	-3.98350330669895	-3.04378682073519	-8.95471146640205
H	-3.39768029095184	-2.31521951043835	-10.46823244769711
H	-5.12816356550858	-2.67817053393078	-10.28723588543363
H	-4.82741106168658	1.11513281545429	-10.02166468737140
H	-5.29481430330492	-0.23902569791456	-11.09872649621767
H	-3.57638202957412	0.18673274019659	-10.88945490688523
H	-6.13854133196094	-3.50033172525175	-8.35159861303425
H	-5.97145381643895	-5.44318890108563	-6.84857428898807
H	-5.51306010001049	-5.09228704195895	-4.40248521458222
H	-5.31346671672046	-2.77597026209381	-3.51149533048477
H	-3.41754932830284	-0.60973196738375	-4.32614389168932
H	-3.34541045062507	1.49708741921927	-2.98729346152713
H	-5.43074602374238	2.82096643333960	-2.61785797630226
H	-7.59706593979181	2.00764943201253	-3.55836299995276
H	-7.66910660538177	-0.10009176298657	-4.89420468351784
H	-2.82853655774208	2.37252662536757	-5.59407434318159

H	-4.24879708925526	5.93738913787844	-5.87701800925271
H	-5.99707921769645	4.09702448198002	-7.71317814646443
O	-5.79146178011945	3.57411335789124	-9.56666223242747
H	-6.17279424391001	3.42649111049047	-10.47169000707744
H	-4.82666739652086	3.37787352957330	-9.62617766290973
O	-3.09950497048738	2.99859712236930	-9.12608885603119
H	-3.16756191720733	3.19587509708477	-8.16842897355200
H	-2.38696499229475	3.58114344043216	-9.44614466494214
O	-7.21363312237752	3.12315870620370	-11.83975786187658
H	-8.07055791860074	3.36832159032796	-11.41723109653997
H	-7.15311696266217	3.70534459278748	-12.61805432495147

E

C	-7.51984142192917	7.48791867241825	-7.91059418990902
C	-8.36309102997192	6.33463020763969	-8.33249202380869
C	-7.24585148139917	7.60427015626940	-6.45860092513117
C	-8.67257946479454	5.33476619448939	-7.48113916680907
C	-8.18874064374523	5.31674273769991	-6.05596193970531
C	-7.56907195203064	6.61560372959881	-5.60310835568375
C	-9.23630273906189	4.78114016990213	-5.08521231276491
H	-10.09896247885477	5.46795133409345	-5.05744651118085
H	-8.81314421612814	4.70573125567867	-4.07061809415512
H	-9.58798916582971	3.78640193099932	-5.40572985177945
O	-7.06453726170520	8.28347036675522	-8.72977073459094
O	-6.98117328261594	4.38430995584824	-5.90432887524661
C	-6.50751849350400	3.68977996420624	-6.91769687940101
C	-5.28845644722429	3.03206632377763	-6.79785242615612
C	-4.08197122656298	3.52367869416204	-6.37375310103032

H	-8.70794597303207	6.34117707947527	-9.37182989893148
H	-9.30085628399505	4.49599621631001	-7.80684287118241
H	-6.74286414032450	8.51700702089627	-6.12519830944104
H	-7.32203611721637	6.68090287576409	-4.53730157414156
C	-3.66293758274068	4.91798191153615	-6.45800024902936
C	-4.31493046941085	5.86510243302481	-7.28666216190273
C	-3.90936022655220	7.20091924163921	-7.30132736466537
C	-2.83440425435387	7.61497074467413	-6.49660744770498
C	-2.55631733607915	5.34555453802546	-5.68288655159117
C	-2.15785422251126	6.68441354262291	-5.69094565386413
H	-4.41858516907248	7.91657517212791	-7.95489216937127
H	-2.50802147491034	8.65976031031013	-6.51720973345771
H	-2.02720588789893	4.61848434466227	-5.05707735549089
H	-1.31155214751011	7.00528424358298	-5.07539233309315
Au	-5.61873811420441	1.02060346522874	-7.20536209614995
P	-5.77982349628210	-1.22886839299605	-7.82351275285659
C	-7.26343753122903	-1.42270297056696	-9.00774302651273
C	-7.32148284666339	-0.15998074773251	-9.89739328436337
C	-7.22860842507925	-2.66817551152844	-9.91186831420714
C	-8.50960084522189	-1.44917410990661	-8.09550808223327
C	-4.09549693292240	-1.70673093989705	-8.56692306977804
C	-3.06987672081437	-1.30901944856309	-7.48057624105308
C	-3.91755664644884	-3.20169465710712	-8.88846742600198
C	-3.87037660838183	-0.85319268855595	-9.83147362373374
C	-6.05105910790474	-2.39458655118272	-6.42780604954127
C	-6.22898926668848	-3.76751803530991	-6.71309018386546
C	-6.42605910426309	-4.70653259103570	-5.69712199781154
C	-6.44460570510966	-4.28522879349387	-4.36176279995516

C	-6.27926331814705	-2.92949352592389	-4.06238920583654
C	-6.08764380732062	-1.96664857796940	-5.07499103836718
C	-5.96524576652612	-0.54435602101751	-4.62830986433664
C	-4.71678972472496	-0.00410032116265	-4.25931243350829
C	-4.63102495791751	1.29593355373953	-3.73932238019018
C	-5.78810283083541	2.07110776006610	-3.57888847827758
C	-7.03505048496620	1.53695552945497	-3.93248948064716
C	-7.12554738410312	0.23837044650893	-4.45182065390007
H	-6.43421593466477	-0.05868213197697	-10.54167643112451
H	-7.40383433428370	0.75464853499725	-9.28367556541247
H	-8.21036694559171	-0.22349832044294	-10.55089455701260
H	-6.37299169616058	-2.64981532621631	-10.60650022116722
H	-8.14893891495320	-2.68192476923081	-10.52386878136899
H	-7.20528101422594	-3.61033854264206	-9.34267990570883
H	-8.51508685185057	-0.58791714861928	-7.40228518312394
H	-8.56584604192956	-2.37332921521569	-7.49778457223865
H	-9.41638711604182	-1.38760634917415	-8.72405786319488
H	-3.23747577158039	-1.86948066244649	-6.54447370441165
H	-3.11674939834466	-0.22775112032070	-7.25702667332259
H	-2.05225926402155	-1.54405882270692	-7.84117456601195
H	-3.96598408510081	-3.82302861985700	-7.98024092081448
H	-2.91496199452661	-3.34283303074267	-9.33163324951185
H	-4.65506119591840	-3.57440017822122	-9.61595147725808
H	-4.05189694603771	0.21846164910046	-9.63258606120871
H	-4.51692266337408	-1.17253887262432	-10.66569018317932
H	-2.82202783079604	-0.96752396912640	-10.16064252810823
H	-6.21604033411096	-4.11178956262844	-7.74841173652492
H	-6.56250617830894	-5.76244714995104	-5.95045656131220

H	-6.59318601357026	-5.00903686077759	-3.55413381695725
H	-6.30322789893329	-2.59277092166190	-3.02109508636819
H	-3.81419483162084	-0.61356357903411	-4.36634769714550
H	-3.65580401362957	1.69878266429505	-3.44656157629595
H	-5.71851923612908	3.08565730437708	-3.17491107597623
H	-7.94474858743511	2.12934268362157	-3.79391248037598
H	-8.10120841047661	-0.18558497051042	-4.71083546763839
H	-3.32665003624573	2.81868467627895	-6.00364388860066
H	-5.10957134413409	5.53195392208548	-7.96345473224636
H	-7.16088289928148	3.56531045013232	-7.79448189203055

TS2

C	-8.40841432798785	6.36927123437061	-5.01039146565949
C	-8.71091924176755	6.80403886666273	-6.38090286047550
C	-8.58203147298241	4.92612121165952	-4.73172101348286
C	-9.00790230770069	5.90415399110471	-7.35768650194158
C	-9.04043419757308	4.47434091746216	-7.08589044514989
C	-8.96499770875188	4.05023686348699	-5.71048935294191
C	-9.55207720432330	3.53861713134874	-8.13715411784290
H	-10.65881121248325	3.58605055872645	-8.15739145619180
H	-9.25757165137807	2.49914392241499	-7.92592480678521
H	-9.18073633282120	3.82722956276827	-9.13261892912655
O	-8.01913442769381	7.17122863430856	-4.14122533801118
O	-7.03948227770297	4.37652499698219	-7.61052896322369
C	-6.28996704956775	4.40693056055679	-6.56869954079234
C	-5.84032408264095	5.57551607410388	-5.98847713095902
C	-5.55224215535811	6.92783202415442	-5.96289255934015
H	-8.64899542600292	7.87734329383158	-6.58544378797906

H	-9.17614085651666	6.23124484105645	-8.38890308881439
H	-8.45512126757348	4.61202623716844	-3.69075441798194
H	-9.11712837812531	2.98928628215719	-5.48337256300817
C	-4.47670757650419	7.57496216827347	-6.74017892428606
C	-3.47330087813720	6.82475681997271	-7.39382990644175
C	-2.47162219470578	7.47058104424066	-8.12079071381655
C	-2.46063887926293	8.87323228960866	-8.21610201892042
C	-4.46348897814495	8.98078921802999	-6.84900779141144
C	-3.46339827302030	9.62460831329597	-7.58754257622664
H	-1.69677299129432	6.88200481854513	-8.62237309393308
H	-1.67554556068551	9.37686867654093	-8.78904659236071
H	-5.25562954716262	9.56359448838474	-6.37027985280440
H	-3.46931173692542	10.71575141605436	-7.67666039089768
H	-3.49143104379354	5.73150329709560	-7.32844893236270
H	-6.06443045604993	3.46142621550479	-6.03708194577815
H	-6.25534433091400	7.59522675280178	-5.43466003378037
Au	-4.68148201103850	5.70953879764438	-4.23801917760766
P	-3.49001058758839	4.13559737771937	-2.93436581253595
C	-4.67640232723867	2.85186337488478	-2.18152480697036
C	-5.77040906309177	2.55371234746025	-3.22975936826939
C	-4.01602523997913	1.52612298943705	-1.75947557068588
C	-5.32754632522783	3.54797843428056	-0.96678226187650
C	-2.14202850727918	3.42237961321767	-4.07282522242593
C	-1.43129958357044	4.66341565288995	-4.65937300293768
C	-1.09983012108341	2.54883082785093	-3.35110946409171
C	-2.82693517633830	2.62952146604643	-5.20268379903044
C	-2.56737256468568	4.94244342723570	-1.57453071930096
C	-1.85402291842202	4.16082628043601	-0.63978043353249

C	-1.03542903453461	4.75133452282000	0.32728364831241
C	-0.90086056747064	6.14525513622679	0.36085372916045
C	-1.61028840597568	6.93316671446750	-0.55008711572333
C	-2.46480346843102	6.35703363450177	-1.51443433075688
C	-3.24697868013876	7.30804736222745	-2.35676304158377
C	-2.58702152786391	8.27426359803695	-3.14619710875015
C	-3.30393535551181	9.32430469932589	-3.73291054688282
C	-4.69099878450861	9.42900083187982	-3.54650988243932
C	-5.37424730515355	8.45744423202361	-2.80512463264142
C	-4.65976612455656	7.39495138937634	-2.21603186862141
H	-5.36162347279072	2.07298635807569	-4.13292413906832
H	-6.29184521858750	3.47635454910447	-3.53385318740803
H	-6.51143866079443	1.86428275654881	-2.78668875904726
H	-3.59345756040369	0.98482001752373	-2.62138179536520
H	-4.79177493440796	0.87875277941748	-1.31191062048119
H	-3.22758645961686	1.65732344286089	-1.00224620222171
H	-5.79757353241749	4.50424598162812	-1.25884503853879
H	-4.60109577695474	3.74677552318488	-0.16269016113998
H	-6.12095333352667	2.89401246157962	-0.56269593267191
H	-0.95931780338773	5.27190254162621	-3.86887979377959
H	-2.13093277154077	5.30638960366875	-5.22219839533772
H	-0.63829110232098	4.32949213964120	-5.35224300538817
H	-0.52992781791932	3.12716567759159	-2.60644557515626
H	-0.37959824751645	2.17799186084776	-4.10284759981785
H	-1.54314152330668	1.67077062372924	-2.85718028169071
H	-3.60885246736258	3.23045310677968	-5.69969674750089
H	-3.27845928067882	1.69241182461621	-4.83648492466007
H	-2.07117511192294	2.35981328290492	-5.96175856928741

H	-1.92760347455830	3.07182121932030	-0.66904427025597
H	-0.49807087047151	4.12264967926947	1.04386813168704
H	-0.25492504049448	6.62102551110594	1.10541800604219
H	-1.53344241163478	8.02395732160627	-0.50368268296202
H	-1.50158808739147	8.21171969076347	-3.27229574490924
H	-2.77349417622889	10.07224763800874	-4.32965626323037
H	-5.24148525661293	10.27155796536156	-3.97594299845055
H	-6.45736465397994	8.51506050934727	-2.67227310498194
H	-5.17406324792857	6.70751050122217	-1.53640844245688

F

C	-8.64861162480878	6.00724469572376	-7.78647213729136
C	-9.21232209572559	5.53073058340634	-9.03811439977639
C	-7.35332305614761	5.27772442760823	-7.29277488202998
C	-8.55073122159335	4.61480043459524	-9.80772835600870
C	-7.25320509587315	4.04150763270960	-9.46756890327951
C	-6.66698603394216	4.40096751126703	-8.29590354851885
C	-6.59754721552121	3.10916979432692	-10.45490959283879
H	-7.24270221306655	2.23702247748893	-10.66711776077326
H	-5.62933043180860	2.74185458067767	-10.07880654354978
H	-6.41932423473500	3.62291093039378	-11.41734997470085
O	-9.13657960256139	6.89714091919312	-7.09136719151998
O	-5.32319712470005	6.78403425409496	-8.62237747783961
C	-5.52548449462861	7.06379178892359	-7.45162283762179
C	-6.51719994998841	6.33569396471786	-6.60868370536464
C	-7.02096548939241	6.88277014829214	-5.40832793127234
H	-10.16239944711026	5.97221264950282	-9.35304332325584
H	-9.00355547426950	4.30593547129889	-10.75820577150077

H	-7.76315453029009	4.60880120468803	-6.50322037220151
H	-5.68533819571221	3.99765637109212	-8.02490279500243
C	-6.79250235543089	8.22717403828234	-4.86314645926201
C	-6.69857083742311	9.36003060344441	-5.70223527159489
C	-6.51271418082145	10.63101843901245	-5.14929399728637
C	-6.42145230273329	10.78905242470983	-3.75741066933622
C	-6.74240409642868	8.40318022383107	-3.46070349825436
C	-6.54278324035520	9.67257256605419	-2.91282513931209
H	-6.46008481422294	11.50589262487177	-5.80479917065035
H	-6.28060100361583	11.78621543029315	-3.32840898387684
H	-6.84635900632335	7.53210814192921	-2.80436650986407
H	-6.49440846221453	9.79844416217679	-1.82669088254742
H	-6.83148227413666	9.24652323725187	-6.78187749343065
H	-4.93763237673040	7.85134618522404	-6.92879966103663
Au	-5.17703862585977	5.47155475487792	-5.02470725282861
P	-3.98452946358614	3.86920809277749	-3.81950665026997
C	-4.97328005768482	3.52442954349922	-2.22306516012730
C	-6.47709816691126	3.68615880472705	-2.54296069034356
C	-4.75089659114425	2.12933150639888	-1.60818843515040
C	-4.55327960443416	4.63321054323713	-1.23327572281762
C	-3.85276938205793	2.40009612789174	-5.02303697862554
C	-3.18671635948948	2.99817716362664	-6.28359989319819
C	-2.99398889237597	1.22989534197785	-4.51018746923205
C	-5.27711142208021	1.91512884151037	-5.35827463729564
C	-2.25756500937747	4.26334932945463	-3.36217311420810
C	-1.55748179484130	3.34124979923665	-2.55070299469427
C	-0.19919275549573	3.50227306017300	-2.26718801463971
C	0.49407402546826	4.58961954813594	-2.81399509579250

C	-0.18738970212837	5.51602227619239	-3.60772630167330
C	-1.56656617716453	5.38850992943248	-3.88283601758512
C	-2.17771075366929	6.50497884747485	-4.66241668772746
C	-1.76695278674467	6.75269971544045	-5.98745895646179
C	-2.16524019737046	7.92159198055448	-6.65074998910938
C	-2.97094014406425	8.86307665489613	-5.99348145887054
C	-3.40611952050460	8.61730002873442	-4.68273265400632
C	-3.02313233825996	7.44059781482743	-4.02408143654499
H	-6.82975944771364	2.96186250496326	-3.29394977393668
H	-6.70040960262524	4.70239363032676	-2.91269657652720
H	-7.05670287509241	3.52238563961399	-1.61698049666140
H	-5.09477638074704	1.32469825479179	-2.27803492131483
H	-5.34634668413364	2.06227580840171	-0.67993600120344
H	-3.70222087419418	1.93902924941536	-1.33356266177007
H	-4.68083908814180	5.63622820666761	-1.68004294044396
H	-3.50488476079960	4.52905165670681	-0.91111013236085
H	-5.19786868604481	4.57729064302136	-0.33779496637337
H	-2.17950434964310	3.39098453564656	-6.06267261968796
H	-3.79082612317236	3.82079724096060	-6.70966770694516
H	-3.08591311674548	2.20911691367289	-7.05013305596068
H	-1.94719993557481	1.53135495008819	-4.34726954481233
H	-2.99476144021038	0.43800033695738	-5.28095993512595
H	-3.38649193159026	0.78712893938759	-3.58188250845657
H	-5.92668999601115	2.75100183099991	-5.67662366518574
H	-5.75228590720893	1.40685822868892	-4.50293438716761
H	-5.22436423922773	1.18987158662908	-6.18967015337812
H	-2.07854614716477	2.47547463921023	-2.13831411987876
H	0.31446026968314	2.77548489419512	-1.63053818431180

H	1.56130577762303	4.72457344710876	-2.61186532026193
H	0.34334104592364	6.38382210555402	-4.01160503524263
H	-1.10952212238336	6.03561302040869	-6.48985485786563
H	-1.83609314462056	8.10211557761146	-7.67882111552259
H	-3.25612846317396	9.79114474705792	-6.49922874211736
H	-4.02869571330428	9.35057889112672	-4.16290201485176
H	-3.31779768518627	7.27684524720991	-2.98246980657406
H	-7.77186017643180	6.28266965141728	-4.87739790406016

TS3

C	-8.62132882067702	6.20322430450886	-7.42582392921320
C	-9.42658975310488	5.88168529702480	-8.55789585087828
C	-7.42911038230713	5.30879110787842	-7.09757061401190
C	-8.98124024978604	4.90829290217658	-9.43072868755714
C	-7.74173276530918	4.17461221619505	-9.29185980114191
C	-6.96390777803242	4.41088102528551	-8.19113042745509
C	-7.33528464970439	3.21433550951377	-10.38297867384872
H	-8.10843772756757	2.44140267710483	-10.54324675407788
H	-6.38715156513794	2.71064469693748	-10.13751188910511
H	-7.20020391738991	3.74724971930189	-11.34166486424649
O	-8.80931797989550	7.18193462842249	-6.65312572321317
O	-5.31351234468224	6.44659093709319	-8.53761232318023
C	-5.48857239465985	6.88216232719880	-7.40409609009353
C	-6.44317902594220	6.27770832723334	-6.44203790364876
C	-7.19204111948381	7.09122416821415	-5.49496582497947
H	-10.33616803649388	6.45695058138372	-8.74885066578199
H	-9.59278012455657	4.69849889138783	-10.31735446471247
H	-7.83770375799958	4.64000959285711	-6.30297669434158

H	-6.03027362564725	3.86178258526334	-8.03550045544720
C	-6.90614949454686	8.47740698679763	-5.11929613822328
C	-6.60574562009344	9.46600789026149	-6.08262249504936
C	-6.33578604063767	10.77717242043822	-5.68240424323396
C	-6.36168698360851	11.11781770654162	-4.31960020344914
C	-6.97366595997729	8.84027554564950	-3.75588540431824
C	-6.68618682011380	10.14908832984247	-3.35655274453971
H	-6.11826839491191	11.54256546575318	-6.43387916417534
H	-6.14871968750900	12.14602435762786	-4.01003609688663
H	-7.23686902538291	8.08287602453264	-3.00960637560146
H	-6.72668959779619	10.41987704173317	-2.29694091893615
H	-6.63192543565734	9.21475969920848	-7.14669950613465
H	-4.87142240031913	7.71970452507816	-7.00853470207544
Au	-5.03551809159948	5.28904984985816	-5.12847204074869
P	-3.84608426856264	3.72401398145486	-3.87936408351557
C	-4.95763515110660	3.23720384229722	-2.40511483954394
C	-6.42779093980505	3.27738203508528	-2.88325876156954
C	-4.68518669086870	1.85131947873550	-1.78942643751727
C	-4.74652577111165	4.34995633396816	-1.35479245521955
C	-3.46521229830596	2.30555992904844	-5.09185293189226
C	-2.72640378776369	2.98934316731749	-6.26538917311741
C	-2.56832827926634	1.19488737696217	-4.51658098271391
C	-4.79912291997494	1.71591131960002	-5.59057625068039
C	-2.21004385640339	4.21789919063785	-3.21737124314720
C	-1.53157866304251	3.33086246350727	-2.35084333791750
C	-0.23389774517075	3.59421848089102	-1.90532370219188
C	0.42209194439548	4.75337903610147	-2.33886867192601
C	-0.23898995187349	5.64453342919186	-3.18852449928171

C	-1.55946293851081	5.40925328609652	-3.62872493775784
C	-2.16702982695009	6.48478557552889	-4.46837214345600
C	-1.69503910589876	6.71832016778651	-5.77463536608225
C	-2.10881894025976	7.85068659227572	-6.49013307562508
C	-2.99076347550953	8.76947364702595	-5.90319122478881
C	-3.48641108005999	8.53551917069702	-4.61173511243735
C	-3.08675111839589	7.39495100474913	-3.90155865099528
H	-6.63207414204164	2.54156755748683	-3.67716649331157
H	-6.69528991987828	4.27887328128497	-3.26490666685315
H	-7.08630414350247	3.04537146966902	-2.02701209408160
H	-4.86602212582173	1.03648988977942	-2.50911387014616
H	-5.38208066065671	1.70497775713805	-0.94439667540162
H	-3.66565582559367	1.75000199345276	-1.38729978542351
H	-4.90679547274681	5.35026284710053	-1.79743501610479
H	-3.73485899280455	4.32207182523348	-0.91900939582989
H	-5.47906450828510	4.21812560461175	-0.53822310444411
H	-1.78692850579471	3.46155708339140	-5.93066653802155
H	-3.35248495143130	3.76366022326132	-6.74498319533623
H	-2.47322722050560	2.22700043144129	-7.02400640985188
H	-1.57092450318051	1.57645928176241	-4.24658205705351
H	-2.42802141801327	0.42338798761727	-5.29555167962009
H	-3.01048625154161	0.70065335396448	-3.63769704574477
H	-5.48483244111180	2.51129376399901	-5.93538822291602
H	-5.30909091526166	1.12498444018378	-4.81160940616078
H	-4.59998837723282	1.04222385187928	-6.44328648672502
H	-2.01855233872392	2.41036101788463	-2.02478557807819
H	0.26385266862234	2.89054595135157	-1.23101510674415
H	1.44317587202596	4.96895062584650	-2.00860740048778

H	0.25989920892410	6.56319529586954	-3.51274385994370
H	-0.97890181344561	6.02063429400752	-6.22071473681558
H	-1.72893111609561	8.02222659818376	-7.50229286403878
H	-3.28997008795015	9.67147607552250	-6.44658163371344
H	-4.16970189063834	9.25189360236323	-4.14761151898152
H	-3.43845373451138	7.23540344242567	-2.87694570857109
H	-7.75739195782373	6.52892360602212	-4.74158789791296

G

C	-8.55896077889069	6.19933517004299	-7.51550526701502
C	-9.32485648254903	6.12059486585898	-8.67587235701160
C	-7.43622690909824	5.25414634016288	-7.24546194547585
C	-8.86875461637765	5.24442841614103	-9.67118939295418
C	-7.66855202720077	4.47246296591297	-9.59670422979546
C	-6.92413942002654	4.52995634928787	-8.43018788303669
C	-7.22383256024942	3.66393979082970	-10.79248276207709
H	-8.02050927758228	2.97791461432821	-11.12983609011210
H	-6.32938388956520	3.06629324146687	-10.55725317707818
H	-6.97565856752752	4.32712295634141	-11.64066325928157
O	-8.67433665294013	7.11895038374657	-6.58724528916629
O	-5.38873985082444	6.49367677038860	-8.49745767864814
C	-5.60649525017673	6.87234042905657	-7.34383119527305
C	-6.49577537367526	6.15040257711973	-6.41266324628790
C	-7.50980220654542	6.95572668098267	-5.59763740334756
H	-10.18790801162220	6.77174422609947	-8.83415136137462
H	-9.45090721456258	5.18706852698615	-10.59923955929120
H	-7.85859119583243	4.48079938973572	-6.55937534770892
H	-6.03609041084052	3.90458009554808	-8.29622505126393

C	-7.12159278166949	8.30389753612071	-5.06724567244816
C	-6.98650097301369	9.41497632059248	-5.91931000300135
C	-6.60264553903328	10.65658105848684	-5.39808345532664
C	-6.34999166827272	10.79698773252872	-4.02431803815575
C	-6.87607467677690	8.45100784124704	-3.69088221634135
C	-6.48748609407883	9.69240241520288	-3.17068832468389
H	-6.50912156858012	11.52049335777681	-6.06370138160807
H	-6.05514169414622	11.77000565714400	-3.61866826028355
H	-6.99420264609311	7.58911159775181	-3.02448809059103
H	-6.30235410074270	9.80097113837879	-2.09731275004640
H	-7.20593396560785	9.31480115783934	-6.98740341882311
H	-5.07612386867347	7.75912863573930	-6.93516574060426
Au	-5.08259507143418	5.17557024872577	-5.16076543638823
P	-3.85667722776201	3.72267094367411	-3.80856433281209
C	-4.93366527641846	3.35326886553025	-2.27560245015871
C	-6.41419304788072	3.35793556510866	-2.72370518831339
C	-4.64846355610314	2.01976471249601	-1.55909969124218
C	-4.70416781651436	4.54609405750840	-1.32125166075694
C	-3.48313355213117	2.20353566859247	-4.89622245891586
C	-2.76673747604566	2.78858639535411	-6.13514927957478
C	-2.56985716974389	1.14998148198101	-4.24461074551526
C	-4.82064305038541	1.56597906458477	-5.32127890818592
C	-2.20714113764992	4.26738640794584	-3.21760039384947
C	-1.51193528641948	3.45898463808907	-2.28946612681087
C	-0.20244517612230	3.75110428929620	-1.89960135265729
C	0.45056162646707	4.85946906736016	-2.45336721242885
C	-0.22723121374135	5.67605610798446	-3.36221858084682
C	-1.56056446646117	5.41403677192860	-3.74650759363299

C	-2.17998613158058	6.42980603538112	-4.64981855831892
C	-1.71718767529191	6.58119256557997	-5.97178442887618
C	-2.12076549799040	7.67660538641001	-6.74783481822671
C	-2.97990126661562	8.64263650505839	-6.20485189878177
C	-3.47383959761107	8.48573210090825	-4.90106795798176
C	-3.08706552416924	7.38003191316223	-4.13145985949903
H	-6.63355511440365	2.56725676767984	-3.45872427381426
H	-6.69090428716075	4.32849325042080	-3.17287497699295
H	-7.05444044128969	3.18835901931992	-1.83919712997467
H	-4.83980010612714	1.15192198200950	-2.21100929461024
H	-5.32893954000001	1.93733904837022	-0.69215280938474
H	-3.62129554642115	1.94910229572781	-1.17003107983652
H	-4.87307608378672	5.50749185734424	-1.84061136331483
H	-3.68420541783337	4.55409825922131	-0.90460841469753
H	-5.42094442234825	4.48081220033822	-0.48283857306925
H	-1.82500110265087	3.29149228219987	-5.85562864192792
H	-3.40403352857223	3.52008210298489	-6.66462541867324
H	-2.52068595535167	1.96782467165905	-6.83310450610556
H	-1.57113307959361	1.55853748621244	-4.02333110015431
H	-2.43624925473305	0.31724910918515	-4.95911420735943
H	-2.99366163248414	0.72761334942639	-3.32013705755484
H	-5.50490970930903	2.32007642375142	-5.75230632875850
H	-5.32979159872139	1.07030705892246	-4.47794709965541
H	-4.62685883319761	0.79757593046976	-6.09140528562505
H	-1.99577517204065	2.57686875131053	-1.86687018378604
H	0.30634774210707	3.10800617162944	-1.17485366668369
H	1.48097162936133	5.09549909864297	-2.16915696503951
H	0.26751415430940	6.55992914688453	-3.77703237502030

H	-1.01283836406030	5.84967674206535	-6.38120604619982
H	-1.74759521118443	7.78497017218537	-7.77111486229506
H	-3.26531385783055	9.51885413250955	-6.79635610813885
H	-4.14971105923939	9.23262904186613	-4.47612936275922
H	-3.43617767734208	7.28306467678267	-3.09821991102245
H	-7.94188694829609	6.33683131377362	-4.79353421588575

H

C	-8.32469551210948	5.90866190866358	-7.42209258247868
C	-9.27224020181703	5.26488686475538	-8.21486213227668
C	-6.95899774141331	5.57378417426097	-7.47528460384870
C	-8.81309674784419	4.25812533453025	-9.08620963519602
C	-7.45308525564244	3.90941800896158	-9.17693906984126
C	-6.52048393287229	4.59045077744219	-8.36112392493566
C	-6.98271872766861	2.83395098879997	-10.13186177889300
H	-7.83027374929004	2.37162339840471	-10.66535141285994
H	-6.43906014192857	2.03001545052098	-9.60026835600168
H	-6.28953414938680	3.24420677468057	-10.89017064954501
O	-8.59453417468749	6.93744983083769	-6.55609755819184
O	-4.69288998805408	7.22234412278958	-8.30790688443848
C	-5.30413472787397	7.46343542563550	-7.27044092169924
C	-6.22495349365958	6.52331527518526	-6.57486675925970
C	-7.39759047677656	7.12654571815061	-5.74184054500069
H	-10.32982880648578	5.53891486233564	-8.16178897823902
H	-9.54039653653102	3.73025472406065	-9.71444645319632
C	-7.24041856512934	8.57236241113671	-5.34757163352488
C	-7.83792433531479	9.60257650951873	-6.09155201375182
C	-7.62638448143483	10.94218208019094	-5.73639796833676

C	-6.81722664689735	11.26390173322121	-4.63722012365769
C	-6.43794009554619	8.89780223336687	-4.23882196826911
C	-6.22462112835989	10.23622260117675	-3.88757681382563
H	-8.09841569773095	11.73970782056211	-6.32069498936794
H	-6.65455058516314	12.31119566639737	-4.36026921327207
H	-5.96927434922206	8.09375354190359	-3.65921136721657
H	-5.59895945231664	10.47927381590712	-3.02173347488126
H	-8.47064860490632	9.33949588152232	-6.94392499196494
H	-5.14924219096945	8.43081181510465	-6.72853335887522
Au	-4.86178678597712	5.46677043759787	-5.32969550989511
P	-3.80669895409741	3.78756795171742	-4.09827074197943
C	-4.88908840704315	3.48127699948686	-2.55267214448826
C	-6.37537437542242	3.60443834417789	-2.95526295166072
C	-4.66664074448758	2.13754991128293	-1.83988979536852
C	-4.53808332417210	4.65631465172443	-1.61147438748948
C	-3.72442620745629	2.31855703748542	-5.31309049225273
C	-2.96342847436081	2.88686943876487	-6.53368371495980
C	-3.00086768008794	1.04786003495388	-4.83188925896799
C	-5.16890713376600	1.96493838276565	-5.72525050394655
C	-2.10584350190058	4.00275591709073	-3.41391346861161
C	-1.48398009225906	2.93113255574760	-2.73300547472792
C	-0.24606801429902	3.07850815939114	-2.09999192964913
C	0.39879829844622	4.32101961205502	-2.12448973014977
C	-0.20343359210996	5.39394496366744	-2.78658862072216
C	-1.44693045926297	5.26093237130874	-3.44156841197401
C	-1.98108943275476	6.48864486051942	-4.09850063314608
C	-1.99258620905507	6.62703396413469	-5.50131711708327
C	-2.38519749713735	7.83562463136815	-6.09318281516445

C	-2.79321141459382	8.91007207031666	-5.29012983721487
C	-2.79944325309774	8.77644371909093	-3.89566015319776
C	-2.38864302512969	7.57562074442780	-3.30204018921734
H	-6.70832237217999	2.77484680564349	-3.59756128095975
H	-6.56546640915110	4.54895274060797	-3.49519216694420
H	-6.99326153470738	3.59234773115532	-2.03851561909576
H	-4.91995684659833	1.28234978584974	-2.48846138121975
H	-5.33086784071589	2.08916668701570	-0.95668040236322
H	-3.63344665143848	2.01898094149528	-1.47685171014205
H	-4.63458230588653	5.62436736807759	-2.13695183627900
H	-3.51145730246584	4.57647378382990	-1.21688963880543
H	-5.23901929284341	4.65779735345805	-0.75651830971921
H	-1.94201638683938	3.20515548025818	-6.25870762983428
H	-3.49485379654879	3.75274904694006	-6.96699899405603
H	-2.88040486012672	2.10316380772455	-7.30906278485149
H	-1.92137386607465	1.21584877223178	-4.69130155011148
H	-3.11184389874079	0.27242367737480	-5.61268598338904
H	-3.42392209617228	0.63821210336946	-3.90001351850306
H	-5.74459586288538	2.86218579705299	-6.01519240317652
H	-5.70634222402606	1.44303923983905	-4.91555648107624
H	-5.13650032889193	1.28533699007478	-6.59659661645553
H	-1.97770651643512	1.96062281591771	-2.68190179590826
H	0.20614251631267	2.22485977521778	-1.58452197985017
H	1.36704570691405	4.45464747186517	-1.63067765931830
H	0.29314444448478	6.36913032137453	-2.81619844819517
H	-1.66461314433189	5.79292922208843	-6.12938844019097
H	-2.41171792836132	7.92206545830848	-7.18304646196146
H	-3.11683024470172	9.84821218115267	-5.75189401630889

H	-3.12230708867811	9.61222095994918	-3.26666465635244
H	-2.38110178521401	7.47363145449539	-2.21135010744437
H	-7.55858564851411	6.51922074986699	-4.82962490932248
H	-5.45292565812472	4.35922496866482	-8.43221517345169

I

C	-10.11015497628680	5.85331016429809	-1.69180239147703
C	-11.05513214531238	4.87427874339243	-1.99474100974151
C	-9.12293624660637	5.64950742175353	-0.71768950210740
C	-10.97563485248406	3.66269644641233	-1.28589311636585
C	-9.98885624974115	3.42208456956942	-0.30951705272183
C	-9.05442163347001	4.44286676316624	-0.02287148486809
O	-10.04955780351256	7.09220123297900	-2.27982035597513
C	-8.81308035984449	7.72635952328794	-1.86856991863842
C	-8.31989774431804	6.92133718062226	-0.61348002555925
C	-9.03202673439663	9.19894430490345	-1.62011893968624
C	-6.80745152827999	6.77581719972856	-0.54928615584899
O	-6.22550495526276	5.78252129611045	-0.17019071412347
C	-10.24820819215991	9.65953180339623	-1.08515906848668
C	-7.99806713840182	10.11758593094711	-1.87002594421214
C	-10.42085000050529	11.01910622385874	-0.79693398288869
C	-9.38078607136309	11.93050451323930	-1.03530675110613
C	-8.16840489550573	11.47705341850200	-1.57356123828923
H	-11.05999708208027	8.94579886741124	-0.91485011394564
H	-7.35626316982321	12.18409461843766	-1.77341149970548
H	-7.05517222719939	9.76793001180086	-2.30740049827376
H	-11.37392603761734	11.37107213025955	-0.38761659654702
H	-9.51838251614109	12.99324291186894	-0.80931728924585

C	-9.90738930674273	2.09047580251650	0.40426546424121
H	-10.83456798762588	1.50753223432395	0.27419473358879
H	-9.73448536478237	2.22305794679251	1.48702140639449
H	-9.07098787016480	1.47932535287217	0.01464101347441
H	-11.70558437055463	2.87532445548292	-1.50718745852930
H	-11.82167520508499	5.04706112743748	-2.75508716258383
H	-8.27337399893066	4.28683261574370	0.72689095421546
H	-6.23812838636639	7.69620657641835	-0.85890589209687
H	-8.08499990954291	7.59358598099650	-2.69443003040739
H	-8.59211503989198	7.48792263147037	0.30419062151691

J

C	-8.76137787596466	5.61558224561701	-7.63592579516080
C	-9.24152225679866	5.19563172319272	-8.93720084259725
C	-7.38392720423005	5.08776060751287	-7.19962898860610
C	-8.47957923071625	4.40563869696544	-9.76063132631102
C	-7.15652813292379	3.91963942943829	-9.41222267801449
C	-6.62479334399670	4.30540431219405	-8.21328577410090
C	-6.38948870713051	3.09053695902287	-10.41423095525881
H	-6.96410800934704	2.20150094125960	-10.73153392944703
H	-5.42720384304327	2.75145400462028	-9.99884499730462
H	-6.17805078256607	3.68212411296130	-11.32382356381804
O	-9.43423662274008	6.32027244271676	-6.86299038767194
O	-5.36070733822426	6.64302252104938	-8.48462347326972
C	-5.61319677392967	6.93258459807199	-7.32516732639931
C	-6.61186198000480	6.18864992158972	-6.50413741777520
C	-7.06286575182842	6.68102700141880	-5.26081719211733
H	-10.22844355232629	5.55853590732657	-9.24039474013203
H	-8.88328871679816	4.12565708874806	-10.74199242614674

H	-7.67688712034864	4.35795582054408	-6.37770520843458
H	-5.61725388442986	3.97738535563862	-7.93665164523732
C	-6.84228696819135	8.02808534413591	-4.70718174738101
C	-6.81584402818198	9.17801966606502	-5.52722584372979
C	-6.64985200976718	10.44590344552342	-4.95998356717085
C	-6.50946661973570	10.58411103583050	-3.57031349439688
C	-6.74417624902976	8.18361135014224	-3.30487374353793
C	-6.56225217578843	9.44966450633344	-2.74287532826709
H	-6.64753740355270	11.33223322194788	-5.60219137914978
H	-6.38209407909479	11.57785781606292	-3.12928458656878
H	-6.80135552657091	7.29890909971063	-2.66186366228578
H	-6.47490862535931	9.55850988921224	-1.65723973336741
H	-6.97856754742187	9.08002688003395	-6.60466989511377
H	-5.06512003450820	7.74395521691241	-6.79767818259282
Au	-5.23790964565583	5.34573124525893	-4.89514648684412
P	-3.90805613752316	3.84939343097833	-3.69079211978990
C	-4.78094560888795	3.55424502718400	-2.01904469232395
C	-6.30207229102462	3.70321489470439	-2.24718284464357
C	-4.50863104587961	2.17795558595709	-1.38212318293825
C	-4.30200703619023	4.69079265438711	-1.09013750458194
C	-3.78390095932147	2.32295922362356	-4.82307435045430
C	-3.22127513248751	2.87598023608866	-6.15226728619725
C	-2.84270734426747	1.21994904098094	-4.30705815826209
C	-5.20422480898611	1.76452840118213	-5.03471239458343
C	-2.16807840151076	4.32015460221284	-3.36427806534037
C	-1.39951560445768	3.45532167091756	-2.55186078572397
C	-0.02447663644725	3.63625313391235	-2.38431172227965
C	0.61782843465091	4.68561090118439	-3.05343587188554

C	-0.13157743417470	5.56222278340927	-3.84214038826166
C	-1.52815779508024	5.41785418176549	-3.99665539899530
C	-2.20806254584128	6.50967351123222	-4.75227363337942
C	-1.87430440229329	6.76561492294225	-6.09716291998563
C	-2.32578003434449	7.92937081892789	-6.73435657019876
C	-3.10777139477597	8.85752681987427	-6.03077929076865
C	-3.46913914326168	8.60103372197383	-4.69964658205841
C	-3.03351463364106	7.42916533200746	-4.06754742694611
H	-6.70875144985326	3.00390670387743	-2.99330364747974
H	-6.55167154940202	4.72468909514693	-2.58548342321912
H	-6.81880203102347	3.52874884566507	-1.28600065497112
H	-4.89550012667306	1.35432633578149	-2.00318008592168
H	-5.03927713169132	2.13762394793810	-0.41387136311984
H	-3.44262159107998	1.99660154655138	-1.17455444621465
H	-4.46315875099455	5.68017925027575	-1.55588877803959
H	-3.23586107701705	4.60033629046702	-0.82779484823002
H	-4.89208823961584	4.65890810098224	-0.15654332172230
H	-2.21453867432521	3.30881581010489	-6.02191805470189
H	-3.87776844626296	3.65784193138566	-6.57728203470959
H	-3.14690582221208	2.05060108604835	-6.88296882233528
H	-1.80027845719344	1.56819290880271	-4.23296010874890
H	-2.86401644735648	0.38613868571547	-5.03220283441173
H	-3.15628779081678	0.81620354689714	-3.33230750723259
H	-5.93512378359697	2.55033944972238	-5.29073847146857
H	-5.57719113695946	1.24558326017526	-4.13629807550894
H	-5.17778230571045	1.02644808561901	-5.85676565573271
H	-1.88049903876829	2.61751096466043	-2.04385033600457
H	0.54078713529311	2.95317660332763	-1.74292288590878

H	1.69704649645523	4.83394139822217	-2.94658870272570
H	0.35863274761824	6.40935890220311	-4.33209461085396
H	-1.23551938899674	6.05842926840123	-6.63647713677291
H	-2.05806564107015	8.11597789035103	-7.77900988967260
H	-3.43435155638920	9.78221010221028	-6.51757241674818
H	-4.07553514799358	9.32242580860674	-4.14511543095449
H	-3.27143025656096	7.25532913640649	-3.01328820116761
H	-7.80765963562344	6.08161395227796	-4.72030012283240
O	-8.12152254767212	3.28860266752195	-4.95180698194103
H	-8.54622388667983	2.73099193344894	-5.65174650377821
H	-8.83872187554687	3.89834802776347	-4.63516834736033
O	-9.77169206976254	5.35183215743633	-4.35914509289135
H	-10.61573179315372	5.47901448536668	-3.89315878691235
H	-9.85899735085539	5.81972812989120	-5.22927625000488
O	-9.01587571207809	2.12373806765068	-7.27323134946658
H	-8.34186596223345	2.38276387229567	-7.93321314329753
H	-9.85485508210083	2.41886309704449	-7.67114316779863

TS4

C	-8.72814006573245	5.66373206876278	-7.54212146621382
C	-9.36208439375366	5.02193222078851	-8.67045799846854
C	-7.32951644157427	5.20940991347810	-7.23948472413711
C	-8.69193348593508	4.11244395974157	-9.46114020477760
C	-7.32670344747706	3.71964711384942	-9.22792140189336
C	-6.66455119482901	4.31684752457176	-8.17204384741660
C	-6.63979225930956	2.75980276310436	-10.17342358461862
H	-7.20010972217268	1.81253880709019	-10.27857802072312
H	-5.62223458091889	2.51947296006802	-9.82550888564134
H	-6.55621497465826	3.19827451543589	-11.18461069876686

O	-9.32149621210067	6.49138221409035	-6.80012689880764
O	-5.00613905351560	6.54215089939162	-8.34689601254066
C	-5.40693711568791	6.90415406311989	-7.25201558977435
C	-6.54008688111069	6.24832708155625	-6.52557617098703
C	-7.04294333292654	6.79664317536526	-5.32206592286041
H	-10.39313281431254	5.31617579736364	-8.88969401748940
H	-9.21901905425461	3.67041539899555	-10.31661366087661
H	-7.73166388510754	4.37410541955338	-6.24202626030421
H	-5.61446712297818	4.07176107962980	-7.98847919063580
C	-6.82331641224393	8.15092842889916	-4.78731685612527
C	-6.71186387890962	9.27711975161297	-5.63218275712052
C	-6.54712462989360	10.55490917543096	-5.08670726647093
C	-6.49636081474472	10.72848194803574	-3.69490592186806
C	-6.80799268478341	8.34249937141047	-3.38613222565938
C	-6.63293786013700	9.61853863713924	-2.84431318231356
H	-6.47752286133433	11.42296342175991	-5.74986669646093
H	-6.37201518079317	11.73044202037714	-3.27205476139152
H	-6.91827872966090	7.47566125011769	-2.72474274063190
H	-6.61136779525354	9.75378971049157	-1.75831202324149
H	-6.80574581473526	9.15296549870349	-6.71502123420459
H	-4.90603440255426	7.72774580898740	-6.69454359739063
Au	-5.22900500912016	5.43551608303648	-4.84593050226249
P	-3.90262243144499	3.87019907860367	-3.73508209918916
C	-4.80526833110131	3.43391595577312	-2.10967327218138
C	-6.32207812071871	3.59336476467424	-2.36070932442363
C	-4.54405670298154	2.01169299804550	-1.57787054898082
C	-4.35698111339527	4.49584919518545	-1.08211136207202
C	-3.73674141493189	2.43655442895687	-4.97992671609937

C	-3.13057379120825	3.09310810123919	-6.24158523408935
C	-2.81001792026146	1.29686063597035	-4.51883664180207
C	-5.14544305563319	1.89389386575335	-5.28687323875473
C	-2.17328017373620	4.32120314413234	-3.33643190420372
C	-1.42623112806726	3.41298724370163	-2.55202528081153
C	-0.05773976196629	3.58740910490650	-2.33263725184534
C	0.59942482291811	4.67546076657188	-2.92076983591588
C	-0.12860122690528	5.59114470253246	-3.68464122322260
C	-1.51883442213327	5.45141518206580	-3.89215820955702
C	-2.17601437417467	6.57018779016993	-4.62827090796982
C	-1.79206103012272	6.87547499815380	-5.94905646367405
C	-2.22580749851589	8.05838903801859	-6.56259097596655
C	-3.04075111241504	8.95659556266826	-5.85812438644428
C	-3.45098090943239	8.65208811083393	-4.55138257710447
C	-3.03286876544530	7.46097671200018	-3.94350826215799
H	-6.69736690627698	2.94154616304833	-3.16370949512458
H	-6.57026920330028	4.63447191143397	-2.63503636211707
H	-6.86259994186239	3.34503416996181	-1.42937026616002
H	-4.90705159544687	1.23958821463156	-2.27515757916865
H	-5.10020208927055	1.88905258583932	-0.63098275043498
H	-3.48325891741830	1.81808041420040	-1.35635096051232
H	-4.50370322381500	5.51843444496150	-1.47552537226045
H	-3.29889017095972	4.38458027859779	-0.79691074540585
H	-4.97337278872140	4.39269837930217	-0.17094758408686
H	-2.12839531463765	3.50841504900329	-6.03983018957054
H	-3.76754646630836	3.91017071105873	-6.62717626263064
H	-3.02967014985873	2.32869601143918	-7.03290267866123
H	-1.77444538191687	1.64269327421227	-4.37299343977159

H	-2.79439961211776	0.52571322810364	-5.31031834115087
H	-3.15930523611589	0.81346988477799	-3.59403564974683
H	-5.84717642722728	2.70135059598617	-5.55367882419509
H	-5.56965390602371	1.34143121239171	-4.43211690575087
H	-5.08340156286132	1.19222685578991	-6.13801433564405
H	-1.91953663921134	2.54573598324519	-2.10941708501396
H	0.49114170442330	2.87021468850797	-1.71472795293008
H	1.67398693571990	4.82105485491345	-2.77160440200337
H	0.37337222135158	6.46357383827620	-4.11452883822075
H	-1.12965401490975	6.19075162181616	-6.48866662921866
H	-1.92084704237689	8.28088773114326	-7.58970112658519
H	-3.35711030962539	9.89461193115119	-6.32538948938958
H	-4.08328427575549	9.35084283707303	-3.99692586265418
H	-3.30727685787145	7.25114557278960	-2.90453023188976
H	-7.82848165747988	6.22288073023959	-4.81722353398479
O	-8.14375534838574	3.63516474320548	-5.24909718485885
H	-8.51586461663979	2.86625980058314	-5.79272266128582
H	-8.92911237999020	4.17469845322461	-4.88150684066950
O	-10.08232822735277	5.23577219868336	-4.63307873877311
H	-10.22103500346831	5.79140351312059	-3.84691284555239
H	-9.97719776255647	5.86149329205581	-5.41077573574525
O	-8.82601734996307	1.84597001468619	-7.03361265358674
H	-8.35060900286639	2.24501111271700	-7.79490304502304
H	-9.75410090470484	1.79935423590673	-7.32995736167643

K

C	-8.42783014752203	5.13578124768826	-7.12793511042514
C	-9.03385905488614	4.16900071233646	-7.96031190029878
C	-7.06534376451079	5.49771693549307	-7.35291159652654

C	-8.29613091240861	3.51769362015746	-8.95253062530809
C	-6.92595881116664	3.79904268786489	-9.15122809394246
C	-6.34802252092448	4.79002963634090	-8.35233785152086
C	-6.12984804832599	3.08685395857538	-10.21972484135867
H	-6.25985663654845	1.99172624378204	-10.15812192656249
H	-5.05462057824669	3.31230170532424	-10.13358176692413
H	-6.45746130839207	3.39707674950287	-11.22897541629946
O	-9.12286702325889	5.71709214668757	-6.12662619228786
O	-4.88959598265182	7.23373257665524	-8.41903572691960
C	-5.27140302156431	7.36268749423140	-7.26961319489359
C	-6.40932300661697	6.60772294093313	-6.62385228255549
C	-6.85388878481658	7.10037822191240	-5.36843616701843
H	-10.09127100954895	3.93926187539817	-7.79632621242049
H	-8.79732501891818	2.78208260368564	-9.59367347608945
H	-8.73210397459392	3.98467433419075	-4.64369710159090
H	-5.30294164292320	5.05032813536864	-8.52301224777997
C	-6.60141019270546	8.43456408358476	-4.79342808188880
C	-6.46429898950787	9.59418935008840	-5.59000597869949
C	-6.25873196208245	10.84325145038220	-4.99373475591847
C	-6.19942678724218	10.95823674781686	-3.59664951273464
C	-6.58138050929214	8.57150831424545	-3.38475298160815
C	-6.37047076787107	9.81852693085302	-2.79232608785564
H	-6.16515712622244	11.73468747254419	-5.62204086695154
H	-6.04452563202238	11.93782248367864	-3.13342560168186
H	-6.71856046867536	7.68322669189620	-2.75806568734352
H	-6.34720889293893	9.90846807678801	-1.70167399777049
H	-6.56429834715691	9.52361687207210	-6.67753902338076
H	-4.75050253790628	8.05076326370646	-6.56583795347886

Au	-5.14374330591338	5.65888087045589	-4.94348976826053
P	-3.98057354090919	3.94853755420356	-3.87424901380706
C	-4.91751143202401	3.56574661311557	-2.25335288096221
C	-6.42094980614375	3.82440623136066	-2.49547202650409
C	-4.74820831973239	2.12614210396556	-1.73133614234572
C	-4.39526421918943	4.58770264655278	-1.22072449107636
C	-3.95726913022003	2.53156462803151	-5.14526235570803
C	-3.30399576830215	3.14737239904808	-6.40314450340458
C	-3.14257822691308	1.29914560009573	-4.71128719793533
C	-5.41393406952558	2.13743582698507	-5.45012389359537
C	-2.22067240043853	4.25112166428586	-3.47190873905500
C	-1.53369967006434	3.25364891312436	-2.74241097405388
C	-0.15602741333118	3.32488303909079	-2.52288147029263
C	0.57002077343042	4.39800994116742	-3.05467363634100
C	-0.09756933284445	5.40046131664141	-3.76297984576057
C	-1.49450561934308	5.36492526112040	-3.96958458007849
C	-2.07616178465277	6.56387753518785	-4.64138974728556
C	-1.67496442193545	6.91128857712284	-5.94717597972231
C	-2.02321153239871	8.15341794371549	-6.49491634951394
C	-2.76938381052422	9.06946105106287	-5.73914942413207
C	-3.19865800152714	8.72582030049267	-4.44833790317736
C	-2.86581191876437	7.47695731151312	-3.90608863655215
H	-6.85782481866118	3.16394491934417	-3.25950176492816
H	-6.59815338782780	4.87189006202528	-2.79937758964113
H	-6.96891711997072	3.64700702819067	-1.55282512485891
H	-5.16336062941938	1.38467793534039	-2.43266407752854
H	-5.30967715259664	2.03455702572477	-0.78417484227869
H	-3.70148630062570	1.86399741645819	-1.51339345419344

H	-4.48928397487703	5.62170048230824	-1.59959846477497
H	-3.34122312980139	4.41114687130412	-0.95329011738245
H	-5.00324082454930	4.50876454590611	-0.30160428411651
H	-2.27227548905477	3.48455072677291	-6.20387553099496
H	-3.88769787338026	4.00565265070245	-6.77990913759119
H	-3.26728578630846	2.38246190607929	-7.19944154219875
H	-2.07574069155177	1.53815861728114	-4.57763412340138
H	-3.21462753999743	0.54309061907137	-5.51405237726550
H	-3.52480947066246	0.83738902504882	-3.78837374523622
H	-6.02356025375510	3.01507870176208	-5.72971055557271
H	-5.89936096917484	1.63129436819298	-4.60035601578053
H	-5.43280030562062	1.43652416097256	-6.30211256469774
H	-2.08180044256043	2.39710056048738	-2.34580389516744
H	0.34603055714441	2.54010861061951	-1.94883649775371
H	1.65226826304008	4.46399957103972	-2.90475535780941
H	0.46043657538365	6.25949292781477	-4.14840917007822
H	-1.06463959499591	6.21007199503304	-6.52576864666985
H	-1.70703191007971	8.40746141096000	-7.51115204606537
H	-3.01898160308803	10.05069676695049	-6.15503347121548
H	-3.78124708473695	9.43667605103072	-3.85575052718364
H	-3.15217974980266	7.23429097935285	-2.87771322396026
H	-7.65744436459595	6.54377852106593	-4.87951477243672
O	-9.24657208116095	3.17856703803414	-4.44766618909641
H	-8.89559727011195	2.51019575767759	-5.10156255286470
H	-10.69396247285233	3.75255142846884	-5.13901756134753
O	-11.25752413919329	4.31476959785203	-5.75583672656245
H	-12.01471120980134	4.62678101541897	-5.22870818173260
H	-10.06257960189326	5.31863523871770	-6.06385280738641

O	-8.32903649092485	1.49781146779585	-6.38676286002929
H	-8.29950105963191	2.09050462096406	-7.17006307832380
H	-8.96689681419312	0.80268183571672	-6.63303630185277

TS5

C	-8.42733683874956	5.65521460081009	-7.48068212982542
C	-9.35902507547765	4.99972639293549	-8.27690949566655
C	-7.08300746907991	5.25516054274392	-7.39595317997597
C	-8.90562980825700	3.89670836861144	-9.02694109535275
C	-7.55896039996170	3.48370608337781	-9.00638647803532
C	-6.64642351922390	4.17906026390686	-8.17857518214242
C	-7.07858450606495	2.33702824674581	-9.86517230466258
H	-7.91897960623203	1.73627616561821	-10.24862239272151
H	-6.40053299417327	1.67119847568303	-9.30330709844126
H	-6.51274115237529	2.71815782788887	-10.73525676318096
O	-8.70490802617814	6.79019322945750	-6.70877889580534
O	-4.70349596441881	6.60353846941725	-8.28059374073988
C	-5.24689049976337	6.97145178975180	-7.24735603842063
C	-6.30537384462940	6.19379914655125	-6.53225586887909
C	-7.30570747133683	6.87285547088838	-5.65828753807686
H	-10.40162611165242	5.32649189495686	-8.31877913782864
H	-9.62192506352497	3.35763574480831	-9.65630523841179
H	-9.33678146241314	4.15339071191595	-3.10592962832901
H	-5.59350696690521	3.88661799489655	-8.14990207145325
C	-7.18793549767399	8.27528826659389	-5.20113594469556
C	-6.95002802130525	9.33786473404133	-6.09730421651876
C	-6.82060392419191	10.64377457384146	-5.61471274364322
C	-6.92808779505672	10.90210785603719	-4.23792218635061
C	-7.32933658816575	8.54698599673479	-3.82502876175441

C	-7.18864797806736	9.85384546584796	-3.34280944917597
H	-6.64077622074479	11.46693366482302	-6.31338435285951
H	-6.82183555290061	11.92586148851192	-3.86488896846170
H	-7.52800593211042	7.72022976742394	-3.13363613968563
H	-7.28535167961731	10.05687522817843	-2.27152537514637
H	-6.89418608317641	9.14218812534482	-7.17219760149825
H	-4.91714595860163	7.89426608832004	-6.71608830114507
Au	-4.96046115769837	5.17384819591354	-5.18832101220689
P	-3.67405469451895	3.62858225379052	-4.01098984291220
C	-4.68293159108780	2.97079408050091	-2.52958463847425
C	-6.17311537458831	2.96775961079332	-2.93688466664117
C	-4.30233006525000	1.55939739933826	-2.04448588994581
C	-4.47976545471886	4.00342377209122	-1.40018635307739
C	-3.25163658722061	2.31094831551371	-5.32523458915588
C	-2.59954113462362	3.11178629487156	-6.47750588387142
C	-2.26866118555905	1.21637993002917	-4.87207206062979
C	-4.56916277377708	1.67422371996907	-5.80834762420533
C	-2.04112041884730	4.17295414973579	-3.37164968445805
C	-1.26338406804292	3.24560146576905	-2.64106559312408
C	0.04346576755919	3.53608305471086	-2.24147460972116
C	0.61124594785594	4.76839697870587	-2.58864720147462
C	-0.14903326339039	5.70249642095897	-3.29641253756378
C	-1.48285657579011	5.44092114222737	-3.68178791481003
C	-2.19713535041658	6.57491795281636	-4.33747477751835
C	-1.71993166855603	7.10128628178931	-5.55384804278262
C	-2.23946350763025	8.29689552164323	-6.06726695016561
C	-3.23753237270888	8.98753347470511	-5.36547566910843
C	-3.74104385476157	8.46028725302138	-4.16698739172346

C	-3.23227456911781	7.25660348251598	-3.65998397981308
H	-6.38293888993674	2.30606534123720	-3.79101164086872
H	-6.52013020987107	3.98068272539112	-3.20264321756116
H	-6.76990282024783	2.61046049731653	-2.07646236884832
H	-4.47048898161997	0.79596082656888	-2.82135929599853
H	-4.94577958890154	1.30057725394710	-1.18356705049967
H	-3.25978547230948	1.48995753134673	-1.69817753632468
H	-4.73048172049635	5.02256202269912	-1.74587991202423
H	-3.44420254567603	4.00967765873444	-1.02355055933784
H	-5.15249246094200	3.75605561474606	-0.55884469359039
H	-1.67933625051719	3.62164086166343	-6.14363855452829
H	-3.28350950779813	3.87552433427557	-6.89052899875721
H	-2.32540849828042	2.41464971368056	-7.28994875124876
H	-1.28179234412519	1.63529233993401	-4.62010684151480
H	-2.12461399093111	0.51207794888222	-5.71174429636360
H	-2.63945375787011	0.63513090849264	-4.01365069353424
H	-5.31321036092339	2.44402241484059	-6.07705019438020
H	-5.01418200211858	1.01270903773223	-5.04636677593703
H	-4.36695510699182	1.06272185412185	-6.70642411261494
H	-1.67989994348690	2.26928810416573	-2.38817209854441
H	0.61634402722040	2.79737100383933	-1.67237948975299
H	1.63855350461849	5.00824670976304	-2.29644654722780
H	0.27719930321376	6.68032124688536	-3.54156582601693
H	-0.92231408817748	6.57667191613148	-6.08995300525270
H	-1.85940707818497	8.69089912053294	-7.01483067943224
H	-3.62483373478429	9.93661090165200	-5.74953696769804
H	-4.51920965891501	8.99686389359136	-3.61703214167374
H	-3.58745640441877	6.87265875886523	-2.69824248181703

H	-7.71741501674930	6.21462942694379	-4.88050774952371
O	-9.15450267954304	4.60603395370507	-3.94796205794469
H	-8.84707369060550	3.88491834617490	-4.58634667297534
H	-10.17481421586092	5.61156857772018	-4.62536911202222
O	-10.60382121220119	6.30407017120753	-5.24054425281136
H	-10.90234689395299	7.04363007075289	-4.67969882792645
H	-9.60423697009881	6.64905069232980	-6.06951251565054
O	-8.22614567500487	2.84843639585982	-5.68980925144871
H	-7.63743248954669	3.35996695539632	-6.29117482303244
H	-8.85501323363891	2.42035679039689	-6.30013577261348

M

C	-8.60559346855930	6.01544893333109	-6.27252045761296
C	-9.86790485079308	5.58927425903866	-6.68078522471010
C	-7.42077110230952	5.58950546573351	-6.90088708226054
C	-9.93006687714982	4.70028200989229	-7.77819647774623
C	-8.77042467486070	4.28169473086806	-8.46262672007605
C	-7.50195274325282	4.73249152004970	-8.00973506070153
C	-8.86364071212330	3.37102225196920	-9.66645278840333
H	-9.91192265653876	3.16729330260846	-9.93586184746579
H	-8.36860774019398	2.40108609814108	-9.47536385161504
H	-8.36456584945649	3.82386108428008	-10.54097888408068
O	-8.36042170867923	6.88184806438331	-5.22744880780799
O	-4.67693302457261	5.85500681726072	-7.96706271735935
C	-5.05436727063655	6.54040136882316	-7.01604492358386
C	-6.27644322683932	6.25556398578358	-6.21717098526220
C	-6.96934538774904	7.41268871969442	-5.45707781369307
H	-10.77539254774577	5.97643442952214	-6.20545575246771
H	-10.90989078163241	4.36477814728599	-8.13404482482854

H	-9.85562748891351	3.82781387001755	-5.27956357353552
H	-6.58237653279019	4.46175506862855	-8.53879708652674
C	-7.09147055700028	8.72681767120426	-6.19183160611701
C	-7.04361947102440	8.81141871585188	-7.59559601193086
C	-7.21109944173403	10.04803982984167	-8.23358960393199
C	-7.43081022557182	11.20827915826094	-7.47801963985026
C	-7.31873400492713	9.89644316442449	-5.44160486752732
C	-7.48668502574793	11.13132071181199	-6.07791355225571
H	-7.16902774197020	10.10470948827153	-9.32601340186713
H	-7.55774450285581	12.17325926590868	-7.97924982479515
H	-7.37176252977354	9.83299362489991	-4.34853569790519
H	-7.66040796299121	12.03457559639775	-5.48408766226362
H	-6.87664099119238	7.91429363075584	-8.20011452558698
H	-4.45606082932769	7.41768904393270	-6.66981646751601
Au	-5.16818751204792	4.91245480853327	-4.92231657322891
P	-3.83048503430450	3.29052969127538	-3.86119720715605
C	-4.80462066427291	2.33052370400766	-2.53379323350443
C	-6.04630569507196	1.70617677578665	-3.19716951588877
C	-4.02075975049851	1.23974161246131	-1.77848910571381
C	-5.24415355943231	3.40884862288589	-1.51719653994695
C	-3.18349874033610	2.25788610261843	-5.34380586855872
C	-2.23083752115381	3.22116459037736	-6.09110368574153
C	-2.45086685761834	0.95297752796412	-4.98838654885387
C	-4.36566020861823	1.92463377554899	-6.27883317386840
C	-2.30787706610880	3.90996965910961	-3.04061136621436
C	-1.38288335563068	2.96379364429421	-2.54031819500900
C	-0.13776558879330	3.34845606261044	-2.03579834931879
C	0.21688198998330	4.70271854773874	-2.03374116613273

C	-0.68865840661292	5.65160881939235	-2.51471565331274
C	-1.95900996029661	5.28533271911139	-3.01115680498047
C	-2.83443353428602	6.41974626113080	-3.42586920190654
C	-2.42805661649996	7.26680017616436	-4.47611202816221
C	-3.11270670024906	8.46325482431110	-4.73259613290359
C	-4.20169676916446	8.83720570538795	-3.93389955348351
C	-4.62778906898629	7.99034621315276	-2.89899878714748
C	-3.95722683752059	6.78487541508177	-2.65117254557855
H	-5.78696237328096	0.84349362211183	-3.83342136823254
H	-6.58787799928561	2.44982962885871	-3.80697463217374
H	-6.73781354426465	1.34508101420822	-2.41542327761901
H	-3.59239665000103	0.47731406179799	-2.44785717577810
H	-4.72005460765706	0.72610895227477	-1.09401395256572
H	-3.21675108599421	1.66891221711033	-1.16153034402902
H	-5.88797788491446	4.17805439878639	-1.97559735627250
H	-4.37139746148283	3.90608966898548	-1.06035436247862
H	-5.82133939370868	2.92575748591999	-0.70835394230930
H	-1.34027747618622	3.46647922009204	-5.49006940487913
H	-2.74129303203600	4.16134407709328	-6.37059542816877
H	-1.89410520603459	2.73465255783910	-7.02404045358455
H	-1.54892066131703	1.12909441989809	-4.38406844034987
H	-2.12202520505096	0.47186098990907	-5.92741461645792
H	-3.10405211242401	0.23658465157834	-4.46352231533823
H	-4.90009071340133	2.84428081615664	-6.58310154565119
H	-5.06537770542470	1.20618117202758	-5.81546593631549
H	-3.97622325123333	1.44739012438453	-7.19538240768079
H	-1.62731440085326	1.90177289516081	-2.55516992552921
H	0.55261396899185	2.58958541644869	-1.65461142196307

H	1.19128915827807	5.02150407037355	-1.65035045572484
H	-0.42831654535066	6.71442102995761	-2.49286668377593
H	-1.55754040992443	6.99243740995397	-5.08077871800900
H	-2.77712627855162	9.11841135004543	-5.54292975568816
H	-4.71265908860059	9.78685376141901	-4.11729224504257
H	-5.46772342653764	8.28176679407833	-2.25900459751007
H	-4.26087637692710	6.15239060232394	-1.81239678245523
H	-6.57639085640558	7.58751052962852	-4.44833446360914
O	-9.31933764151412	3.28557510333717	-4.65141710221868
H	-8.63318374418914	2.78605111348850	-5.27852975684602
H	-8.73541754524501	4.02142678772518	-4.05863476508602
O	-7.99074752553678	4.96794290062801	-3.41308333604135
H	-7.04383150836555	4.85268773509644	-3.71820340473683
H	-8.23819083600878	5.82914496553663	-3.86473592367138
O	-7.68186794472645	2.28055977545700	-6.28090994698446
H	-6.75788776937839	2.12788980548248	-6.00205690797009
H	-7.62158547902153	2.99635358700615	-6.96427786935487

AuL^+

Au	1.93040236281960	-1.82461541760501	0.29583876245063
P	3.78009958598331	-0.47693958491515	0.16324654573726
C	3.24672666423731	1.33410289772452	0.42067276268626
C	2.28864728163556	1.35704587588572	1.63465675861973
C	4.40615949186168	2.31780625513025	0.67896009337991
C	2.46260150813038	1.73635389979962	-0.84765330340374
C	5.20495815726765	-1.09440321200702	1.24675635375958
C	5.31809962755277	-2.60522519832418	0.94930578312130
C	6.54588559532418	-0.41255260516914	0.91016734541347
C	4.82460573191010	-0.87090981370053	2.72343662833670

C	4.32771254886224	-0.70628886120643	-1.59218678192783
C	5.27025158318955	0.22053902829805	-2.08859728102832
C	5.79707677661413	0.11597071649226	-3.38006484590253
C	5.39394592645935	-0.93749142455858	-4.20787467979022
C	4.46440745946786	-1.87057496528888	-3.73602917383717
C	3.91011926177622	-1.77655369844403	-2.44467888797544
C	2.94037229823277	-2.83790745572168	-2.03120126057289
C	3.38488236980921	-4.12363771456212	-1.68972540591608
C	2.50663540097452	-5.07062655433818	-1.12465406226609
C	1.16992491610094	-4.74253961140063	-0.88357862549488
C	0.67303389569882	-3.47814198977414	-1.28711563247452
C	1.54879479271428	-2.54141511647919	-1.90053797899772
H	2.77563897219077	1.02311962755607	2.56411374779469
H	1.40389373447962	0.71981838651933	1.45958996381648
H	1.94136016500665	2.39419493949672	1.78786200833950
H	4.94846570030689	2.08677867817619	1.60993709853543
H	3.97700972166485	3.32946082334001	0.79017952628802
H	5.12811202955770	2.36189975585510	-0.15122506873080
H	1.65870656104934	1.01169949659636	-1.07100475654106
H	3.11205910882161	1.82053173420701	-1.73333554238858
H	1.98903419013829	2.71839933787524	-0.67192617581096
H	5.57335859575180	-2.78807108711598	-0.10812902012154
H	4.38169340019951	-3.14155796166256	1.18579078289805
H	6.12482202549467	-3.03079757048035	1.57205896685531
H	6.86256570764393	-0.62286835063234	-0.12419340577119
H	7.31866603604394	-0.82315678783996	1.58452795425364
H	6.51774542213597	0.67675395633780	1.06247141293902
H	3.83643550909277	-1.30432056816823	2.95857820430384

H	4.81619321738721	0.19792592341665	2.99331486715106
H	5.57506757861558	-1.36862077882263	3.36279920784320
H	5.60719701217934	1.04257295604954	-1.45295723830542
H	6.52340402345338	0.85509365967916	-3.73145961957777
H	5.79928993840463	-1.03447645127125	-5.21978310370712
H	4.14379908368867	-2.69401264577033	-4.38204163599566
H	4.44187216222135	-4.37532712441303	-1.82117623287720
H	2.88285586142969	-6.06209856821603	-0.85485309671411
H	0.49424904883430	-5.46722649663530	-0.41982378032381
H	-0.39878696923271	-3.26142920499618	-1.22671680487950
H	1.15223192681750	-1.61577112891646	-2.33257537319093
H_2O			
H	-6.55838292565059	2.41074620341420	-7.25618941975477
O	-6.41198813612710	3.28498502475719	-6.85187376297092
H	-5.80346893822231	3.07618877182861	-6.12024681727429
H_3O^+			
O	-0.62626579701475	-0.77816330788305	0.08805399209473
H	0.34355660896699	-0.75248302484197	-0.11518363228482
H	-0.86338438576570	-1.56525757344292	0.64193545741291
H	-1.17435642618654	-0.73248609383206	-0.73661581722282
$(H_2O)_3$			
O	-7.57262654549561	5.81046629735177	-2.88113534708068
H	-6.97756268386370	5.51707105252181	-2.15815142784497
H	-7.27807274691895	6.72466030960187	-3.07853738087013
O	-6.43987897069863	4.12605371560502	-0.92739274933673
H	-6.37620321658253	3.56160749307589	-1.72130053298137
H	-7.38981304310216	4.06926937895211	-0.71040600208822
O	-7.72220301521688	8.25996196021390	-4.20762154807110

H	-7.67818658025350	7.57637941644359	-4.90349302320301
H	-8.60554319786791	8.09666037623394	-3.82476198852373

$(H_2O)_2$

O	-8.42557309187042	5.09923714712154	-3.24424415982804
H	-8.59553912062145	4.42108420812026	-2.55633268601227
H	-8.94136115477821	5.86196074312372	-2.92463123283770
O	-7.17743083472774	4.00587092143956	-1.04754346803127
H	-6.93894487939558	4.58214387450492	-1.80417553130760
H	-7.29379091860651	4.64525310568995	-0.32101292198310

B’

C	-8.99211095860512	6.13516570347454	-6.33740060431382
C	-8.22668178917576	5.25941426929581	-7.24726930395800
C	-10.38130367664131	5.72963938316647	-6.02208589126827
C	-8.67666624315674	4.02542561585700	-7.55526766052032
C	-10.00317894971325	3.48192252383449	-7.07814107297709
C	-10.84459497755664	4.51064208542563	-6.36393144578573
C	-10.78283969971511	2.85103281080516	-8.23870247175061
H	-11.03325035617265	3.61497343216886	-8.99259838592650
H	-11.71272967965521	2.39994861718855	-7.85563281521842
H	-10.17524777196099	2.06299340949017	-8.71145150597643
O	-8.46836569658116	7.12093594804395	-5.80083580179837
O	-9.77003642941359	2.36052703947172	-6.15544845341013
C	-9.11337266653568	2.68031590006070	-4.92998821046392
C	-7.64270482316391	2.79047577344811	-5.03972663612875
H	-9.36643746230058	1.85981314260817	-4.23871133747748
H	-9.45942007908701	3.63814538714606	-4.49032329359989
C	-6.43874003169777	3.12475890334797	-4.92901211361206
H	-7.24272569518370	5.62123267780616	-7.56213245470866

H	-8.06350785329649	3.33684338029752	-8.14921403786957
H	-10.98657174365523	6.44617661058998	-5.45759971138377
H	-11.85430048352230	4.18797466833306	-6.08101984043691
C	-5.21841108331399	3.83325144666756	-4.67417954792760
C	-5.30735706307963	5.12697827688797	-4.10385285645763
C	-4.13340972389858	5.84351619679184	-3.85331875871926
C	-2.87960853780983	5.29353161076502	-4.16435549914160
C	-3.95486150880857	3.27772837198528	-4.97781179747977
C	-2.79221377489756	4.00974835711482	-4.72569942902787
H	-6.29254808319055	5.53557682026088	-3.85482206079415
H	-4.19753888531607	6.84344481115916	-3.41249621894831
H	-1.96760504372820	5.86597644100325	-3.96705767744906
H	-3.90200340251131	2.27285637404694	-5.40838237824449
H	-1.81454633298362	3.57967077356277	-4.96486201223249
Au	-6.52317124255910	1.22692973528902	-6.09521089436063
P	-6.01892536841441	-0.61387126941780	-7.41978260772899
C	-7.57027473762420	-0.96576935851123	-8.46093788196717
C	-7.77944784081048	0.21280364774378	-9.43150244960862
C	-7.53588390995706	-2.29336380689161	-9.24008588022073
C	-8.72564815720359	-1.00925911280114	-7.43374752730935
C	-4.44469065019850	-0.19950947572316	-8.41266219371824
C	-3.28021482795081	-0.37872346238223	-7.41341786326941
C	-4.22331258990885	-1.07321657532105	-9.66069529404573
C	-4.51476410674365	1.28607523404352	-8.83352828109315
C	-5.67900551637506	-2.14254212008438	-6.46511018346874
C	-5.31926594232144	-3.31307942006545	-7.17172599067609
C	-5.07277788834576	-4.51877342674866	-6.51018542758608
C	-5.19021139976362	-4.57748964295017	-5.11585407315146

C	-5.53246698180680	-3.42545318668689	-4.40137219341117
C	-5.77070227507451	-2.19547145963418	-5.04908776721242
C	-6.06468986033429	-1.02654756639112	-4.16232918522424
C	-5.00877238002655	-0.21062149490300	-3.70167973489943
C	-5.25056040462769	0.81281719774004	-2.77491819588030
C	-6.54823236167577	1.03009256546846	-2.28983304702464
C	-7.60029758337972	0.21360363185132	-2.72830578506362
C	-7.36385769335447	-0.80812706578202	-3.66004946688769
H	-7.03305536145454	0.22012553543524	-10.24271266248027
H	-7.73936481227294	1.18163626504834	-8.90357851516540
H	-8.77635155233349	0.11843390245891	-9.89796198249215
H	-6.68831250352720	-2.35332810656213	-9.94099058970038
H	-8.46428673474908	-2.36695062374241	-9.83511623323772
H	-7.50649458584530	-3.16341474918272	-8.56558212378425
H	-8.87562962380280	-0.03073374701080	-6.94255476655109
H	-8.54780653761995	-1.77422512462546	-6.65753629999563
H	-9.66127758703586	-1.27326495922144	-7.95919980114566
H	-3.13395806688473	-1.43326751673573	-7.12969286815335
H	-3.45237565794705	0.20729834057892	-6.49211005338670
H	-2.34813266866878	-0.01220104081251	-7.87961027213725
H	-4.12536953769664	-2.14218997079199	-9.41590573215995
H	-3.27928165370817	-0.76141018314846	-10.14298360768585
H	-5.02921767161420	-0.94798749417820	-10.40223347960979
H	-4.62303707150948	1.94980243942587	-7.95713750230854
H	-5.34716161601090	1.49176161192324	-9.52410811602772
H	-3.57492842547783	1.55167790779533	-9.34981513260756
H	-5.23012339451547	-3.28584186297893	-8.25901845677067
H	-4.79402356179261	-5.40770911128874	-7.08437210678848

H	-5.00707889330668	-5.51621274119181	-4.58346694052013
H	-5.60867882905041	-3.46199611177811	-3.31008113307437
H	-3.99165899918116	-0.39928313878848	-4.06032336828001
H	-4.42239324974474	1.43762705890769	-2.42687724330586
H	-6.73486553278865	1.82540167171998	-1.56176209267799
H	-8.61029862978694	0.35991322065949	-2.33069578063034
H	-8.18462204188243	-1.45244932654493	-3.99083087666711
O	-8.50801781680825	5.62642705237762	-3.43121966099446
H	-8.70620526656729	5.94917983541482	-2.53456725800870
H	-8.53626789162936	6.43302063688926	-3.99320213483519

C’

C	-7.03882584193780	6.43751476058570	-6.90169709008538
C	-7.05498185863147	5.19423646235478	-7.63721786041871
C	-8.24349946055918	6.84798899030480	-6.18666874977298
C	-8.16051742444633	4.39356399298678	-7.63015135312068
C	-9.48589418638456	4.85655737221527	-7.08315218190682
C	-9.35460895629210	6.07736912881150	-6.21029185462497
C	-10.39857610597175	5.21125627610919	-8.29186717273230
H	-9.95826159995962	6.02520916367776	-8.88766228863124
H	-11.38562410846778	5.51868695841205	-7.91116999349889
H	-10.52396547660029	4.31382969069231	-8.91850956593150
O	-5.99785722352659	7.17094941717361	-6.81102926658517
O	-10.21216483264740	3.83574797013548	-6.38313221516816
C	-9.50476722516012	3.18605455868359	-5.36935944698657
C	-8.19176237871063	3.03685592294861	-5.34919180937626
H	-10.18718653863775	2.77633446630697	-4.61628061645182
C	-6.89024248623637	2.77625224194206	-5.45112005028772
H	-6.13501365151486	4.86535810752830	-8.12998631711584

H	-8.15624523306094	3.43386720127605	-8.15836219620322
H	-8.17928411631778	7.76742623743269	-5.59704207162401
H	-10.25101937444882	6.34977317756327	-5.64134438143379
C	-5.87102379204399	3.72753169205069	-4.96691625338961
C	-6.21040783105583	4.86235974845346	-4.19206301988997
C	-5.23449669426663	5.75857378798533	-3.74446662487440
C	-3.88179686520623	5.54103596745957	-4.05205836279612
C	-4.50788863935993	3.52482812803624	-5.27924951588584
C	-3.51835406755809	4.41879800464101	-4.82942867508048
H	-7.26458872447165	5.02339535103583	-3.94226493335932
H	-5.52418659719466	6.62717135978235	-3.14472855337998
H	-3.11294068004667	6.22651820662590	-3.67971748709673
H	-4.23880201456953	2.64014961553103	-5.86736057332946
H	-2.46050488910567	4.20317199894276	-5.02384840691701
Au	-6.42054792800349	0.99925177538354	-6.39324549421987
P	-6.04038400667301	-1.00378382937114	-7.53652310049245
C	-7.71504017765212	-1.58070769107853	-8.23384257528061
C	-8.21260560314551	-0.50754442172984	-9.22307126837850
C	-7.72448261671331	-2.96620635786897	-8.90341092397845
C	-8.64636567320550	-1.61853366323242	-7.00007819264548
C	-4.69705061307257	-0.68445828029169	-8.85739418372638
C	-3.37304349331268	-0.63315995092816	-8.06148863721368
C	-4.60043925487989	-1.71818285722292	-9.99238314553854
C	-4.95700804213708	0.71048442503524	-9.47084317201630
C	-5.42445556219043	-2.39821199121721	-6.50612092925287
C	-5.09873312540629	-3.62586923476987	-7.12643192826186
C	-4.60267995924852	-4.70868864248172	-6.39513244703460
C	-4.42156080139231	-4.58309320983557	-5.01222534854244

C	-4.73817055936150	-3.37604633154366	-4.38203810938059
C	-5.23928060835242	-2.27235690661572	-5.10376567554661
C	-5.53094502719110	-1.04021525890086	-4.30579901943263
C	-4.49170317722036	-0.14164980013620	-3.98661338464499
C	-4.73013604591884	0.94838980869226	-3.13826413896235
C	-6.00760343375776	1.15813429070984	-2.60136979399885
C	-7.04565905919277	0.26865267091927	-2.91001397334047
C	-6.80896506046960	-0.82769916960098	-3.75076234810024
H	-7.63672219787762	-0.51617912317317	-10.16381090634047
H	-8.15234777122266	0.50300499179797	-8.77907776514874
H	-9.26885116254981	-0.71099624473624	-9.47624628916599
H	-7.02981429459556	-3.03520538738953	-9.75523855398867
H	-8.74228868273699	-3.16000174471400	-9.28926909511048
H	-7.48844271525384	-3.76732358452674	-8.18520433194071
H	-8.70910096227575	-0.63000942818862	-6.51055068300561
H	-8.29678631689008	-2.35679087795187	-6.25753733730170
H	-9.66156546649144	-1.91467579205218	-7.32150544328741
H	-3.09607615386330	-1.61985398676435	-7.65598207187858
H	-3.44655830583915	0.07984600596435	-7.21945944144226
H	-2.56119963952023	-0.29307947941880	-8.73010314075600
H	-4.37359562204012	-2.73157890449464	-9.62683990451740
H	-3.77546779995193	-1.42456635628866	-10.66744163502699
H	-5.52165316680674	-1.75549802511556	-10.59676362330698
H	-5.00417233411175	1.48775537708358	-8.68692409527287
H	-5.89697914747058	0.74792326115738	-10.04364869452463
H	-4.12975554404732	0.95500529127682	-10.16231660249009
H	-5.23356867414103	-3.74254386983847	-8.20280026330357
H	-4.36007139036357	-5.64574381650377	-6.90614867388041

H	-4.03493945901417	-5.42207782323556	-4.42478020585817
H	-4.60010420658685	-3.27105315491263	-3.30118493024371
H	-3.49086626491645	-0.31426896503588	-4.39603670263807
H	-3.91288699376860	1.63343738259017	-2.89083093371230
H	-6.19184662474878	2.01045668806200	-1.93977396807119
H	-8.04434557005721	0.42239820128899	-2.48892246010077
H	-7.61566466414825	-1.53242605226415	-3.97359842904192
H	-3.78267876522706	5.48631575311439	-6.33607224524580
O	-3.91004829659371	6.10557080090089	-7.20427509639025
H	-3.18560451786617	6.77537917555351	-7.17768659621283
H	-4.87821858613380	6.60697835621492	-7.09268320285016

D’

C	-9.07441308415626	4.06017934923717	-8.79397476851458
C	-9.27089091996321	3.09193128654240	-7.69183244873682
C	-8.57368179741846	5.40486189552358	-8.42347996852301
C	-8.78850455814718	3.33498753173572	-6.45660229689110
C	-8.10925942906032	4.62750997513032	-6.08535714878915
C	-8.11521130754813	5.66036975483815	-7.18188527335281
C	-8.65231016261379	5.19578014409549	-4.77202310772494
H	-9.71202834795673	5.47308470674476	-4.89171471335702
H	-8.07588327458433	6.09010222248494	-4.48436260424154
H	-8.56509179045449	4.44391974843646	-3.97108207197683
O	-9.24436483289666	3.73595429700728	-9.97420954386852
O	-6.67934198945910	4.29635388809702	-5.75890038034047
C	-5.90056078840118	3.89393450464570	-6.77255172396256
C	-4.68730810860948	3.28379106297266	-6.53634734259362
C	-3.42205747570171	3.41168785706420	-6.09799377040370
H	-9.75429319210008	2.14580695232845	-7.95043926755385

H	-8.85391574633068	2.58206883518990	-5.66291705208474
H	-8.54864288644046	6.15567924616301	-9.21956608455980
H	-7.69641288987339	6.64025359748135	-6.92125670516735
C	-2.72791944411401	4.66651381303357	-5.78828210800948
C	-3.35730072122720	5.93125620176825	-5.84058923194791
C	-2.64568720747785	7.08886557793696	-5.52040705933792
C	-1.29369225471039	7.00911267215052	-5.14276072275949
C	-1.36887082220757	4.60007769790925	-5.40635317776019
C	-0.65755798540456	5.76135718312902	-5.08564515766827
H	-3.14325202811815	8.06325538612870	-5.56242948488412
H	-0.74014212608239	7.92000383567643	-4.89346311377344
H	-0.87258353071125	3.62401202636060	-5.36456697796731
H	0.39478506251859	5.69335854182384	-4.79217699844720
Au	-5.55134454855999	1.45758363708553	-7.16876055531564
P	-5.89027508913847	-0.68714985993147	-8.04720675753387
C	-7.51433324665744	-0.69813845525341	-9.04281258747837
C	-7.62460597874707	0.66353276894462	-9.75924562179176
C	-7.65455493805148	-1.82744640470965	-10.07890599812187
C	-8.62742491740252	-0.79940040438396	-7.97672432397013
C	-4.31850751731604	-1.04086100988954	-9.05968303774744
C	-3.15993471233424	-0.81555972836982	-8.06081618733936
C	-4.21340888684701	-2.46900583948424	-9.62460767433036
C	-4.23823788018215	-0.00437794003582	-10.19910347850413
C	-5.98690187203106	-2.03410683578995	-6.80501743422561
C	-6.22567319710766	-3.35252092436441	-7.25505572153880
C	-6.25898303522050	-4.43223335692848	-6.36855736741947
C	-6.04080675677391	-4.21115846817789	-5.00293867204757
C	-5.81219620203711	-2.91162334982269	-4.54039598251239

C	-5.79202017284278	-1.80710937512607	-5.41724789898492
C	-5.59121172885879	-0.46393902551938	-4.79199849182005
C	-4.30680425023565	-0.03854042517078	-4.39985514360225
C	-4.13864995422422	1.17078275751508	-3.70984315124668
C	-5.24869390172481	1.96648693751240	-3.39547061793606
C	-6.53150253475494	1.54471586768085	-3.76976322918764
C	-6.70454443910121	0.34050771683884	-4.46652153065384
H	-6.87391161990959	0.78368308127189	-10.55486660456264
H	-7.49197559231064	1.49760921618788	-9.05172184874316
H	-8.62466321481235	0.75560705585469	-10.21856374037314
H	-6.87951276097344	-1.76666041526359	-10.85969583774705
H	-8.63408931943743	-1.71791886132268	-10.57887561567950
H	-7.63106877339308	-2.83108981562584	-9.62661231347890
H	-8.50924451171769	-0.01742583407214	-7.20455084120693
H	-8.63522433433668	-1.78107837762932	-7.47578590626492
H	-9.60738925605456	-0.65353011266415	-8.46637466574253
H	-3.22319990673587	-1.51047266434189	-7.20552779754619
H	-3.15729319799613	0.21928658572667	-7.67238501418545
H	-2.19892739999824	-0.99317902362616	-8.57605607606233
H	-4.15740033574320	-3.22332818415416	-8.82396267822147
H	-3.27917066856522	-2.53984619137259	-10.21085401079170
H	-5.04573580743804	-2.72419224972036	-10.29883664477287
H	-4.39970697611790	1.02404118292439	-9.82850054007295
H	-4.97550612838880	-0.20987352718347	-10.99303090110033
H	-3.23402325810617	-0.05374109489956	-10.65710136017757
H	-6.38610040089971	-3.54191647603289	-8.31773397530208
H	-6.44946700982603	-5.44153936999302	-6.74630544092573
H	-6.05629221519005	-5.04745071345506	-4.29683356459983

H	-5.65684908722947	-2.73118263909850	-3.47197755938878
H	-3.44007635596670	-0.66647648446055	-4.62954607796960
H	-3.13581054183657	1.48874910147174	-3.40698859206873
H	-5.11466748238368	2.90927402790747	-2.85677436806143
H	-7.40364610328237	2.15104215578812	-3.50610113616002
H	-7.70869507257206	-0.00221184583082	-4.73739556511860
H	-2.82074244719989	2.50284507700968	-5.98108450466900
H	-4.41137200214686	6.00367800491825	-6.12601104288072
H	-6.24872412971507	4.07592215381126	-7.80831631723697
O	-6.30251713114206	3.75723544131223	-9.91255271067626
H	-7.16289331751379	3.76811134683007	-10.38014457586687
H	-5.72653424166048	4.32733137547681	-10.45221040383898

B’’

C	-9.10060577099178	6.20700916089867	-5.90088152325127
C	-8.24810977057681	5.50815395007124	-6.88722341805342
C	-10.48582159095912	5.70595495348271	-5.74298090035504
C	-8.62712227643279	4.32902042512934	-7.42159489770696
C	-9.95738338714527	3.67483830231015	-7.12942048079312
C	-10.87429265296968	4.54137185376687	-6.30031786919269
C	-10.64414639799836	3.24192515637998	-8.43163194596426
H	-10.88298700083604	4.12107208958817	-9.05161321883162
H	-11.57486346662939	2.70084651494745	-8.19476781534122
H	-9.97889869680420	2.57039208188551	-8.99894810418057
O	-8.65591852805336	7.12184460625559	-5.19710123263494
O	-9.73811127598560	2.41054664450719	-6.41066174418035
C	-9.17253553501690	2.52936322499133	-5.10597984080655
C	-7.70617197784347	2.70878131303892	-5.11596167207456
H	-9.43070836922086	1.59617958105156	-4.58056062918017

H	-9.56752856579173	3.39017368879560	-4.52784237899894
C	-6.51421606069592	3.08470445933442	-5.00973845672096
H	-7.26819779941232	5.95152122664135	-7.09141809405659
H	-7.95634713009843	3.77168399906986	-8.08752527672424
H	-11.15032172483665	6.29617597301769	-5.10374916375794
H	-11.88029766449828	4.13867870190677	-6.12846388620760
C	-5.36003067994868	3.90235481526004	-4.76473088229849
C	-5.56678744828892	5.26166445729913	-4.41808958751714
C	-4.46220954808514	6.08533884931641	-4.17407798721278
C	-3.15703773556161	5.57586390191044	-4.27828480659824
C	-4.04587294338262	3.39550252540243	-4.87171214643983
C	-2.95228969008247	4.23203187432179	-4.62886458443150
H	-6.58740977106997	5.64379048187344	-4.33675734556961
H	-4.62335844142219	7.13665273727793	-3.91421137134522
H	-2.29849717753049	6.22884888118404	-4.09178301787927
H	-3.89901444207796	2.34560831340561	-5.14297230223285
H	-1.93518064759570	3.83664095187522	-4.71319569111059
Au	-6.47948001196083	1.17596638614081	-6.11811252058509
P	-5.99481422036807	-0.69025973077615	-7.40911452711409
C	-7.54165347628531	-1.00532264169558	-8.46968863851231
C	-7.72100353387419	0.18748375164703	-9.43009859442475
C	-7.52391499641003	-2.32753678279506	-9.25856238323165
C	-8.71051813441180	-1.03385382434461	-7.45704793080448
C	-4.39396132740987	-0.32648776792853	-8.37661883019147
C	-3.25596111846801	-0.50260616504112	-7.34671782972807
C	-4.16086730495198	-1.23266222527874	-9.59881234914531
C	-4.42722529369845	1.14990955352988	-8.83250582361725
C	-5.71472132714022	-2.21725812323439	-6.43024659035835

C	-5.38933883018806	-3.40907076768923	-7.11757464916549
C	-5.19660414443709	-4.61462781166770	-6.43776079475145
C	-5.33544351633647	-4.65163849765244	-5.04483909982324
C	-5.64398448857978	-3.47876310148904	-4.34895576657445
C	-5.82694049050434	-2.24887120442000	-5.01422947782058
C	-6.08484653386990	-1.05631193271115	-4.14775688074155
C	-5.00360246257909	-0.26623817189705	-3.70173965347328
C	-5.21827850876059	0.79465857075583	-2.81054841795519
C	-6.51025093923725	1.08066557397723	-2.34583737627204
C	-7.58463353675122	0.28172742069365	-2.76269751195480
C	-7.37748597206925	-0.77997652301495	-3.65633265202920
H	-6.97099782317725	0.18543293699211	-10.23812172721651
H	-7.66435566625008	1.15010830085237	-8.89163867837956
H	-8.71836324814316	0.11832420082907	-9.90008898675094
H	-6.66426390571690	-2.40516940178852	-9.94256357268831
H	-8.44143447725298	-2.37291373199596	-9.87287876960411
H	-7.53157425208309	-3.20288049321423	-8.59031333458304
H	-8.86566151367590	-0.04619952498145	-6.98779584035404
H	-8.54722812191923	-1.78804229192402	-6.66726407969862
H	-9.63902928183014	-1.30157755655159	-7.99298269316589
H	-3.14132263201447	-1.55150412688553	-7.02887169052804
H	-3.43470685542705	0.11620168065211	-6.44820620697016
H	-2.30542772527698	-0.17255305383324	-7.80284023189051
H	-4.10061307885198	-2.29846759078218	-9.32948785036689
H	-3.19482748172562	-0.95770492062377	-10.05949901959031
H	-4.94106498744207	-1.10056477907807	-10.36605054575625
H	-4.54331836237666	1.83519761016542	-7.97370203618590
H	-5.24022853215492	1.35405439009816	-9.54617146328284

H	-3.47130541073935	1.38681877926147	-9.33321972603409
H	-5.28474859247232	-3.39993674977997	-8.20381650966410
H	-4.94325598930682	-5.52053308364642	-6.99703511866502
H	-5.19498246086437	-5.58981324575760	-4.49868645841998
H	-5.73595095172792	-3.49846029733915	-3.25842751824195
H	-3.99130717933863	-0.50261182923768	-4.04574941518769
H	-4.37034698263569	1.39956792279744	-2.47350661733773
H	-6.67346732990216	1.93068181293900	-1.67609161139718
H	-8.59245928664537	0.48240452813880	-2.38386881850378
H	-8.21765211512981	-1.40628868743403	-3.97328917105801
O	-8.71505207513272	4.95135530932292	-3.23944077128560
H	-8.05930578434306	4.80795922635647	-2.51697907180577
H	-8.76172062536320	5.91815527222630	-3.36402325017145
O	-6.61490429114169	4.36918421000389	-1.48427250606607
H	-5.81803106217208	4.60235697800978	-1.99980893045065
H	-6.48650758162961	4.80548652490199	-0.62195720877399

TS1’’

C	-7.96645879237129	7.00017881743514	-6.13414693351022
C	-7.59773890616197	5.94644195924866	-7.10417001403872
C	-9.28828437189777	6.84484743379034	-5.46303286623730
C	-8.37600293829149	4.85902573213672	-7.30903877405491
C	-9.74547163285357	4.69438505165711	-6.69807177030390
C	-10.09051886087634	5.78420518260054	-5.71099536689519
C	-10.80601307044281	4.67368602805294	-7.81946256576817
H	-10.81231917790809	5.62920288966840	-8.36714817500670
H	-11.79971261659291	4.49356575591016	-7.37797102947689
H	-10.57458542435420	3.85153070726857	-8.51596082761722
O	-7.20981916639849	7.92740070665065	-5.83962885000167

O	-9.91474494162153	3.40542557610297	-6.04406123056666
C	-8.94314731751990	2.98631168850994	-5.11652242260916
C	-7.61383625711668	2.93060356458213	-5.36173039283116
H	-9.39850054841361	2.28093303463232	-4.40576347629599
H	-8.68754597756546	4.05981927840852	-3.93498267577516
C	-6.32219443779785	2.82297490324454	-5.52058590462594
H	-6.62995646230880	6.06445894481225	-7.60078044018569
H	-8.06435343677593	4.06762036756406	-8.00035619483625
H	-9.57779945514225	7.65095461089626	-4.77652117272578
H	-11.06104527437373	5.67041632417857	-5.21032417413973
C	-5.24824362346037	3.74898197619913	-5.14390191099990
C	-5.53504632730969	5.08882350575847	-4.78807634295842
C	-4.51302055325511	5.99317865889954	-4.46928526795777
C	-3.17585714139413	5.56199808191087	-4.47742513765459
C	-3.89673653510533	3.34154221690801	-5.16578680685866
C	-2.87485590191603	4.23478924184604	-4.82327919989114
H	-6.57462986754489	5.42109971030117	-4.79486890054570
H	-4.76433969769885	7.03541194355199	-4.24621687701274
H	-2.37128695308293	6.26305075614038	-4.23402217132444
H	-3.66743064322541	2.31136676426159	-5.45717709802639
H	-1.83271021103106	3.89957276262700	-4.84036906447268
Au	-6.03469258829501	0.98695673528351	-6.45976026256089
P	-5.78257039030320	-1.03924265344667	-7.57136714144264
C	-7.42418608997416	-1.40522123411918	-8.46113345682570
C	-7.66755879321443	-0.28671656425818	-9.49438277380603
C	-7.51107004032691	-2.78645776344609	-9.13422608032690
C	-8.49369450527072	-1.32514196195637	-7.34792452980754
C	-4.26486178148894	-0.90115064666160	-8.71993504918596

C	-3.04252493252428	-1.06041566686938	-7.78836986356598
C	-4.19593507247856	-1.91442079191868	-9.87684674528474
C	-4.25407239328241	0.52836246164066	-9.30992852886253
C	-5.49822426108931	-2.48485643616733	-6.46939322360957
C	-5.21288777405407	-3.74211502969610	-7.04864135857620
C	-5.00567746688383	-4.88112973074910	-6.26581694947074
C	-5.08752660008506	-4.78437504871428	-4.87129952867360
C	-5.36583017803672	-3.54815811627207	-4.28116526314967
C	-5.56949181031813	-2.38594946662186	-5.05484024327618
C	-5.83137262967890	-1.12809486848744	-4.28609046930577
C	-4.75577670332595	-0.31498542314044	-3.86947380692773
C	-4.97921422026145	0.78701307751862	-3.03261565509624
C	-6.27718348324234	1.09488489254669	-2.60171638525611
C	-7.35008994698818	0.28753721797641	-3.00452631654510
C	-7.13047094092633	-0.81897090657622	-3.83638599830757
H	-6.98767561033968	-0.36948577128831	-10.35859563355300
H	-7.54302310912819	0.71299629841865	-9.04011927739780
H	-8.70184191120984	-0.36668137790383	-9.87518793999878
H	-6.73931883305454	-2.93499080249917	-9.90538699746853
H	-8.49571108285899	-2.87065318634994	-9.62993113055775
H	-7.44207083647438	-3.60400253887587	-8.39887047823367
H	-8.49047935419187	-0.33757856127014	-6.85234794884236
H	-8.33374387705618	-2.10263076490795	-6.58084420628576
H	-9.49142049302387	-1.48843110428087	-7.79437215033666
H	-2.95251871050851	-2.08575244149332	-7.39452821418049
H	-3.10247654725011	-0.36250131096368	-6.93299821379400
H	-2.12392524369439	-0.82457344718786	-8.35551237197721
H	-4.16771721211916	-2.95978783784004	-9.53238114679942

H	-3.26323423397019	-1.73470327549677	-10.44207488619280
H	-5.03470583459320	-1.79630475868780	-10.58230812522963
H	-4.25117283215560	1.29240081085215	-8.51249643994035
H	-5.12186606175434	0.71983245710618	-9.96030858079670
H	-3.34033649237380	0.65433397949008	-9.91862883349841
H	-5.15061203471593	-3.83750610889915	-8.13403293377313
H	-4.78423440666432	-5.83922091507078	-6.74626542008245
H	-4.93148612984073	-5.66750648631972	-4.24354882805257
H	-5.42427704434840	-3.46386384890833	-3.19133446168519
H	-3.73976527381866	-0.56707539867838	-4.19033150538520
H	-4.13379379620402	1.40405838976324	-2.71212546013669
H	-6.44601509276653	1.96022599495117	-1.95335343284918
H	-8.36493985029457	0.50619822076924	-2.65453596769414
H	-7.96755938617745	-1.45885208708170	-4.13136371645602
O	-8.69810146933278	4.80595046864532	-3.09749600863413
H	-7.76350084600648	4.79740398224059	-2.61143446559031
H	-8.83929589573212	5.66453101642247	-3.56316240664553
O	-6.43419229078140	4.62106207931649	-1.99913019912677
H	-5.73630106168971	4.75518805250662	-2.68542715436092
H	-6.16505802200734	5.14359402190037	-1.22046179937097

C’’

C	-7.73725145589818	6.19339889893867	-7.01920203538257
C	-7.87195217877895	4.92824056987380	-7.72662351410125
C	-8.71495920514486	6.51638846657167	-5.98013301605106
C	-8.78502848228395	4.00525197489048	-7.33290688010507
C	-9.86298020236112	4.30449461438733	-6.32750677437714
C	-9.64396463550846	5.60797351967483	-5.60447812607730
C	-11.21476119516466	4.39000749858104	-7.08825593634977

H	-11.19622344715993	5.20571339620003	-7.82724543404919
H	-12.02136092028496	4.56691754292651	-6.35889770304650
H	-11.40136183479528	3.43006646333322	-7.59600211663485
O	-6.76897505802069	6.98026196831114	-7.22913670854181
O	-10.06404261253385	3.23766551817477	-5.38181569710915
C	-8.94896584161509	2.78422847299978	-4.68448381453761
C	-7.67824834304158	2.92909575572241	-5.02200057349708
H	-9.27023439045420	2.23616591695460	-3.79110888487913
H	-5.46063521449850	2.86083982152620	-7.81055995814611
C	-6.39339828824590	2.89987282446352	-5.36161286297569
H	-7.15546927926296	4.69376590589421	-8.51720593800942
H	-8.83683283186272	3.02473063799260	-7.82072868315719
H	-8.59684591489103	7.47777763663058	-5.47094953246724
H	-10.33073426574435	5.80722389187958	-4.77348446328108
C	-5.44442873034934	3.98508134675846	-5.04945623260316
C	-5.87986668997878	5.21479110264299	-4.50189824422787
C	-4.98897014924604	6.27338794624756	-4.30609772870707
C	-3.62799971433187	6.12190391534333	-4.63029705859835
C	-4.06852888337624	3.83596712692267	-5.33958645938558
C	-3.16693289267558	4.88917304323405	-5.13109501502999
H	-6.93826108789972	5.32603267383889	-4.24735809062806
H	-5.35059217329510	7.22500565807828	-3.90367589795842
H	-2.92834968996628	6.94955931788961	-4.47535114995721
H	-3.72190939099770	2.87405134323636	-5.73623953937738
H	-2.10326952282364	4.75349188914409	-5.35452852248110
Au	-5.86200194174619	1.18519710889570	-6.38415058975633
P	-5.61130591625599	-0.81949994267467	-7.59552955013102
C	-7.19088206506534	-0.91781911593266	-8.66328458826228

C	-7.15477943228826	0.24845314298346	-9.67200903968472
C	-7.42976563356416	-2.24739491411259	-9.40129177887524
C	-8.34494904060735	-0.70241396729617	-7.65516229457434
C	-3.98739687784864	-0.91284043204046	-8.59572539603616
C	-2.89509988980127	-1.34540432175896	-7.59423383440614
C	-4.00619625941116	-1.86482511914548	-9.80610647729980
C	-3.65603748410764	0.51426340021771	-9.08668658419868
C	-5.67033456559651	-2.31676882514604	-6.53029972031864
C	-5.60836072090749	-3.59518071254346	-7.12918792107847
C	-5.77173953312605	-4.76401579248065	-6.38037476041248
C	-6.02029115974386	-4.67279156940478	-5.00497506137398
C	-6.06227707906768	-3.41736811246656	-4.39240178014688
C	-5.86836732357340	-2.22688264232933	-5.12571069117747
C	-5.82621391884266	-0.96434790579101	-4.32882814034075
C	-4.60226294387747	-0.27985562086632	-4.14413582100801
C	-4.51745758510517	0.80842755770630	-3.26365729840794
C	-5.65238593707924	1.23344695162356	-2.56033704858578
C	-6.87054570109708	0.56405398123081	-2.73848489413181
C	-6.95889249202675	-0.52836446950858	-3.61207339101637
H	-6.41145841415016	0.08106631428849	-10.47047645838701
H	-6.92905781192209	1.20912213659220	-9.17464288534239
H	-8.14375993545428	0.33816459936152	-10.15721926099216
H	-6.61612079802629	-2.50653637851371	-10.09573028563748
H	-8.35520172460306	-2.15023652628701	-9.99809994177093
H	-7.57945325960634	-3.08214143701987	-8.69867788947236
H	-8.26898884834490	0.27769281262225	-7.14951868746140
H	-8.35192200080922	-1.48833945094688	-6.88016907021504
H	-9.30867311259911	-0.74602708345732	-8.19452213433997

H	-3.04659659963982	-2.37600980144318	-7.23539751735341
H	-2.86858130795339	-0.67165641448163	-6.71961048034852
H	-1.90904814562004	-1.29342641670980	-8.09065051328925
H	-4.21831102826407	-2.90721043119732	-9.52181292714047
H	-3.00688100480191	-1.85338755423286	-10.27850943386517
H	-4.73585186345276	-1.55030795297135	-10.56979822452712
H	-3.53144233888831	1.19608777219970	-8.22646803922919
H	-4.43942586254365	0.91092718776132	-9.75513491339681
H	-2.70629119747940	0.49274164254169	-9.65132588168384
H	-5.43825364164356	-3.68413562311840	-8.20380967596229
H	-5.71444497134261	-5.73960401694490	-6.87308171492265
H	-6.16415814882530	-5.57794941486473	-4.40641226180330
H	-6.21890315976363	-3.34192027602108	-3.31168250165253
H	-3.70662377743430	-0.64045254981848	-4.65893561135371
H	-3.55981193790804	1.31941342488273	-3.12308971047132
H	-5.58626366598303	2.08274462124727	-1.87352300584698
H	-7.75742997736247	0.88732483390923	-2.18386323341509
H	-7.90990622494649	-1.05542657153953	-3.74047113832593
O	-5.15217008490635	3.52453031904074	-8.50768574222029
H	-4.81174387531291	4.87814587067417	-8.05838300956602
H	-4.48330540492359	3.04469985288446	-9.03080549452906
O	-4.63990901121650	5.90070738560690	-7.77853174549393
H	-4.17763978355333	5.88254981127543	-6.90666796059275
H	-5.59947305748733	6.40900397625664	-7.62991339643708

D’’

C	-9.16328863608929	3.81858006215558	-8.79850865991087
C	-9.34161487814665	3.05050958384520	-7.55125271359591
C	-8.42284924586110	5.09424112538903	-8.70825824517356

C	-8.75809734949757	3.43902490275465	-6.39919917707025
C	-7.98322597706167	4.72342927093768	-6.26405906413034
C	-7.86915139661445	5.50172271758797	-7.54790703036455
C	-8.55894085503266	5.59388214878418	-5.13758900869378
H	-9.59215590664943	5.89083623284992	-5.37788695385774
H	-7.94078571726988	6.49798797013986	-5.01394837814533
H	-8.55449641680635	5.02759509393696	-4.19213920027875
O	-9.57710478404580	3.37803199542272	-9.88180953113532
O	-6.62162036660753	4.36711005958641	-5.77358122488253
C	-5.74393725942000	3.91262127657826	-6.68532693569889
C	-4.60003620289404	3.24653844765129	-6.31027603531288
C	-3.35993127741171	3.29449986831757	-5.79516576298374
H	-9.91943276686664	2.12503628803657	-7.62646744555919
H	-8.83740724042487	2.83095970946057	-5.49140572768087
H	-8.31705155063643	5.66997763628968	-9.63204226721026
H	-7.30176976822671	6.43900064030438	-7.49292505359654
C	-2.62079875446645	4.51013390424785	-5.43415038295540
C	-3.20229129219625	5.79849005279672	-5.45976978932000
C	-2.45154882865292	6.92046310759054	-5.10379973454442
C	-1.10786119285096	6.78089818444628	-4.71462944334650
C	-1.27145692335478	4.38243952044610	-5.03454638693370
C	-0.52068529436553	5.50849408222878	-4.67917510163199
H	-2.91319262180366	7.91310330055793	-5.12365129681095
H	-0.52351580828052	7.66370903484454	-4.43624545439420
H	-0.81259454093979	3.38761418281900	-5.00913644653432
H	0.52436412206638	5.39330830965069	-4.37443722796467
Au	-5.51993218866537	1.48856227443583	-7.06597560371920
P	-5.86238444856642	-0.62725736000701	-8.00376495675368

C	-7.52665996520619	-0.66830355508517	-8.92808849268281
C	-7.69353510041678	0.68243094525973	-9.65377403850326
C	-7.69371606326902	-1.81337645124670	-9.94290206912170
C	-8.59100902082177	-0.77982332999100	-7.81483995500819
C	-4.33356507192262	-0.89920748341506	-9.10265430853684
C	-3.13018444340294	-0.60194120754020	-8.17864764827850
C	-4.17683853108607	-2.31982460528825	-9.67487383366049
C	-4.38514722878649	0.13409896672667	-10.24886974038537
C	-5.86735394588927	-2.00465121819083	-6.78786044205532
C	-6.02470854542009	-3.32377976239560	-7.27135464349033
C	-5.96450413380304	-4.42938804524991	-6.41887031208848
C	-5.72878849398765	-4.23450582221565	-5.05247389824649
C	-5.58449021840035	-2.93609342962689	-4.55495739000201
C	-5.66582810584239	-1.80558356916550	-5.39538828442537
C	-5.56783867638061	-0.47604464084197	-4.71953848750475
C	-4.34065067295324	-0.03467461506788	-4.18670161385594
C	-4.27307252072052	1.15152005683697	-3.44150926238311
C	-5.42942166297535	1.90820913500477	-3.21024302283639
C	-6.65746680495753	1.47030643872119	-3.72360119401496
C	-6.72902252918894	0.28964817487839	-4.47645330613676
H	-7.03204903895225	0.78382680992015	-10.52600822333748
H	-7.48106076285228	1.52548383686432	-8.97522295579522
H	-8.73433252612229	0.77948410625037	-10.00857498744271
H	-6.94648123390067	-1.75902055881609	-10.75063939417288
H	-8.69037511675227	-1.71606309265264	-10.41073240035322
H	-7.64961858919969	-2.80992251090929	-9.47633851539130
H	-8.47797243550947	0.03325456410953	-7.07545328272254
H	-8.53625234249258	-1.74403697578565	-7.28320899257775

H	-9.59423479745599	-0.69188210616829	-8.26981886074798
H	-3.12135887013376	-1.26304574656143	-7.29396383105321
H	-3.13920550179880	0.44724006926076	-7.83188378691918
H	-2.19403293196020	-0.77155074922151	-8.74067461707650
H	-3.99684462799955	-3.06505560512794	-8.88385598151978
H	-3.29491947608539	-2.32615290193481	-10.34117292624375
H	-5.04398833862496	-2.63689722454061	-10.27536031092331
H	-4.63906991534553	1.14857402573825	-9.89116384969581
H	-5.11968065607957	-0.15424532400698	-11.01895831110001
H	-3.39338072864320	0.17261673735834	-10.73488413634614
H	-6.19147251691344	-3.49441399001652	-8.33586720379928
H	-6.09378281778335	-5.43758089055043	-6.82453921947379
H	-5.66828769810299	-5.09004568249057	-4.37231039250207
H	-5.42412990757983	-2.77667164316530	-3.48391036211584
H	-3.43837140666447	-0.63260412281299	-4.35286924675941
H	-3.31324959988070	1.48205876434828	-3.03154157359629
H	-5.37440744675982	2.83207356026036	-2.62663762891448
H	-7.56790162590521	2.04343033120246	-3.52133733499326
H	-7.69285815156631	-0.06724934148654	-4.85282923781838
H	-2.82182007550029	2.35134340640278	-5.64790480922313
H	-4.25210596129609	5.91566981255400	-5.74589667237479
H	-5.94328432140350	4.10375371367806	-7.76011888576298
O	-5.60017634733100	3.56717706821338	-9.65917976206637
H	-6.23099386893915	3.58591970504547	-10.43203774691194
H	-4.73053656958219	3.77114596060944	-10.04408097695804
O	-7.47463206204415	3.54593530554818	-11.60603410031799
H	-8.37426521119241	3.54330531856804	-11.19224378113324
H	-7.55462941857549	4.09986576412236	-12.40162351845110

J’

C	-8.74735206846520	5.63371951436480	-7.65622789263164
C	-9.19949415568539	5.24636095086182	-8.97212909752041
C	-7.38384969398764	5.07509869994140	-7.19478099305577
C	-8.43456368992288	4.44395021277169	-9.78082433721454
C	-7.12319663953278	3.94078517204012	-9.41838391467926
C	-6.61080081193783	4.29789598452999	-8.20774371958453
C	-6.36064930300396	3.10006332115174	-10.41196067324456
H	-6.93525688242839	2.20115619348384	-10.70070778634480
H	-5.39197783156575	2.77460602915322	-10.00016521534255
H	-6.16374348083696	3.67187921963332	-11.33730546537097
O	-9.42530493138488	6.33303245507847	-6.87988787383010
O	-5.39749974920443	6.68870516730331	-8.48341849419040
C	-5.63344633379346	6.94600658492180	-7.31385213764354
C	-6.61663331310370	6.17856524612160	-6.49254280461658
C	-7.06119647206325	6.66993904994791	-5.24642928848870
H	-10.17752363259156	5.61869910509838	-9.29102953844665
H	-8.82794417438380	4.17170164714203	-10.76844184350215
H	-7.70325489230496	4.33723357622488	-6.40444626018817
H	-5.61317397174494	3.95417379488289	-7.91426448695345
C	-6.84479208307421	8.02107995288878	-4.69857337089219
C	-6.83871277895953	9.16865491215930	-5.52218319560132
C	-6.67481263970362	10.43949615323070	-4.96075712397129
C	-6.51815799149268	10.58319710543375	-3.57342310403666
C	-6.73070795741861	8.18191795174698	-3.29815993240463
C	-6.55233953026519	9.45099881300260	-2.74192477623229
H	-6.68751181409116	11.32374617273303	-5.60568585331061
H	-6.39298122933207	11.57927172847706	-3.13704798550996

H	-6.77241205190366	7.29900586186163	-2.65141846187963
H	-6.45277696936060	9.56384412469083	-1.65774444262365
H	-7.01500628873374	9.06615184664676	-6.59707896494502
H	-5.08130157518693	7.74643130579367	-6.77331041788474
Au	-5.22170657136003	5.35155202340147	-4.89731637772751
P	-3.89770182815504	3.85211132053735	-3.69264400031984
C	-4.77150090824439	3.56057580589217	-2.02062766742319
C	-6.29276274900265	3.71164666234042	-2.24705917304573
C	-4.50015110917795	2.18541443903597	-1.38106503210072
C	-4.29034176271968	4.69814163531658	-1.09410228182877
C	-3.78325596973439	2.32557842059558	-4.82533964939440
C	-3.21221348927770	2.87489517950425	-6.15269990948409
C	-2.85378833222348	1.21321937554221	-4.30805875084967
C	-5.20782681299268	1.77929754076761	-5.04248290862870
C	-2.15647005269623	4.31733301015281	-3.36871857404597
C	-1.38532746213790	3.45021855463767	-2.56130732622668
C	-0.00984565555073	3.63179178999190	-2.39779602469590
C	0.62943523956309	4.68371365290465	-3.06593556734119
C	-0.12274835465210	5.56208525759750	-3.85009569392674
C	-1.51960197759056	5.41722591087388	-4.00070868235009
C	-2.20359415439697	6.50851907682086	-4.75364605314323
C	-1.87607526612105	6.76349502328444	-6.10036605338550
C	-2.33198586993262	7.92591461982834	-6.73686168250823
C	-3.11185586082081	8.85398404072751	-6.03087747786956
C	-3.46710698384893	8.59875610459126	-4.69790615009064
C	-3.02738113780811	7.42793440613775	-4.06633783261352
H	-6.70274957433646	3.00799685358954	-2.98733422941472
H	-6.54116370507138	4.73158660671563	-2.59044453710490

H	-6.80888509303482	3.54339052542339	-1.28443037157836
H	-4.88997132423203	1.36077907567351	-1.99909553292816
H	-5.02745196175543	2.14789188035435	-0.41086707157387
H	-3.43366528565383	2.00317207479414	-1.17695796029817
H	-4.45151823585068	5.68694487471359	-1.56093649262686
H	-3.22365773947461	4.60715604752429	-0.83414657710686
H	-4.87815373422514	4.66805117052232	-0.15901498453199
H	-2.20071099876195	3.29528953597439	-6.01911090552622
H	-3.85868693894430	3.66487167889760	-6.57780504275420
H	-3.14638385658998	2.05038672203202	-6.88508685836657
H	-1.80912278215400	1.55333967783734	-4.22852432347346
H	-2.87767577418888	0.38171027871747	-5.03573734883564
H	-3.17450867624719	0.80906415087527	-3.33578036580599
H	-5.92754055675233	2.57036347193636	-5.31487426444884
H	-5.59150004925973	1.27217059814953	-4.14156899146815
H	-5.18208287029051	1.03446258852947	-5.85835694744499
H	-1.86402521099205	2.61033863267983	-2.05438975201479
H	0.55819463273402	2.94736361777708	-1.76032912003312
H	1.70889594772239	4.83228427698982	-2.96196975116756
H	0.36561821805230	6.41051871102576	-4.33962564161588
H	-1.23907922355187	6.05642280886262	-6.64192682480031
H	-2.06986416372953	8.11140389077405	-7.78309033883336
H	-3.44227669840380	9.77725391573401	-6.51765764019559
H	-4.07249821364989	9.31992314751035	-4.14189597665034
H	-3.26004567098039	7.25540251067621	-3.01068190835207
H	-7.80214742521033	6.07425919457788	-4.69696871051977
O	-8.25585702215867	3.21230738618905	-4.90935951515890
H	-8.79028670255944	2.51216743899386	-5.32447136004051

H	-8.92401508093730	3.89066259727638	-4.60842386891132
O	-9.77991505583311	5.35001326251641	-4.40529992076417
H	-10.63792506895683	5.50641226568757	-3.97497165792836
H	-9.81736209837694	5.81362682666779	-5.28581288658471

TS4’

C	-8.73692367166964	5.67328108648582	-7.48013985262050
C	-9.41377714442303	4.98489352290583	-8.54671711040117
C	-7.32625881236931	5.24857682981771	-7.23855646061268
C	-8.77196567349597	4.04215586684055	-9.32744939226115
C	-7.39536007141121	3.68388524182369	-9.15534499570026
C	-6.69043028094925	4.33064673420746	-8.15861403383035
C	-6.75268127673859	2.66894100465403	-10.07039647272328
H	-7.27563924495016	1.69582968911088	-10.02549205710502
H	-5.69608792842395	2.50414363683890	-9.80352367862208
H	-6.78731888751659	3.00576322134486	-11.12242962292370
O	-9.31049587514818	6.51694101374545	-6.72288650869814
O	-4.95891479863313	6.55233074540812	-8.32108988076396
C	-5.37739912220587	6.91490308752452	-7.23348275856619
C	-6.52853075761584	6.27009508825128	-6.52141685092739
C	-7.01657018617597	6.82848763562725	-5.31462888341524
H	-10.46084211196620	5.25005447281232	-8.72240353223823
H	-9.34186285404270	3.54824670002891	-10.12450937020595
H	-7.81362605165297	4.30304037983872	-6.25209211350016
H	-5.62889971309039	4.11037624900546	-8.01556921580542
C	-6.78913446554970	8.18754687025171	-4.79436595027754
C	-6.68412603235148	9.30350702004896	-5.65346599675860
C	-6.51081405144397	10.58718403747698	-5.12466607085215
C	-6.44662892942102	10.77712053414527	-3.73553226515867

C	-6.75859615276749	8.39543973462562	-3.39590334503128
C	-6.57690414820566	9.67748817276494	-2.87065371732949
H	-6.44564539419244	11.44714210921387	-5.79872163907760
H	-6.31641886345743	11.78373299045302	-3.32570858954451
H	-6.86198501344258	7.53643516450703	-2.72298092246404
H	-6.54427439179038	9.82546010626177	-1.78658440294570
H	-6.78920747855158	9.16637114365139	-6.73372174945248
H	-4.87953178152557	7.73298114391008	-6.66456851547038
Au	-5.21114674539075	5.46124253480087	-4.83895735078933
P	-3.88986731122041	3.87621058322965	-3.75468414062139
C	-4.82732341446799	3.38192087479426	-2.16633457905219
C	-6.33992604133482	3.52448083057807	-2.45354267271330
C	-4.56050979719030	1.95076593004393	-1.66204675881774
C	-4.42399866446776	4.42557426995063	-1.10164108034174
C	-3.67886281608490	2.48261878167720	-5.03673646410076
C	-3.04821174949735	3.17964819135815	-6.26447735122013
C	-2.75009111463265	1.34237361592553	-4.58078928186655
C	-5.07214762975580	1.93170671287857	-5.39474400584092
C	-2.17643323531391	4.33167985358042	-3.30094107017350
C	-1.44309165501699	3.42207303494285	-2.50517731850529
C	-0.08546283353257	3.61321194226404	-2.23797158631436
C	0.57435237157070	4.71963295060908	-2.78782022562485
C	-0.14006240542981	5.63313147656183	-3.56713437481494
C	-1.51962898704333	5.47469077748674	-3.82521808239005
C	-2.16320334838349	6.58225996656785	-4.59103167990779
C	-1.76325425384382	6.85353667660318	-5.91435978293056
C	-2.17997003802312	8.02570871512180	-6.55950377848261
C	-2.99211772493085	8.94772160082094	-5.88333231038501

C	-3.41851882792115	8.67698900644909	-4.57439784040669
C	-3.01923575866930	7.49550611892866	-3.93519160295115
H	-6.68047557545570	2.88548648558598	-3.28245108622718
H	-6.59824544466795	4.56844802888811	-2.70717338997333
H	-6.90116425868419	3.24173574928116	-1.54455113755297
H	-4.88835190972819	1.19108545791897	-2.38992664231091
H	-5.14321202431602	1.79497547179832	-0.73621732516558
H	-3.50420111837384	1.76760839078190	-1.41258251362973
H	-4.56901214165648	5.45510508017824	-1.47767291012935
H	-3.37376679897886	4.31912656241083	-0.78735585929827
H	-5.06623272078824	4.29550846176741	-0.21203370232896
H	-2.05437224705838	3.59453307498775	-6.02470851833238
H	-3.67911408257497	4.00316054491182	-6.64580597820341
H	-2.92134395183815	2.43788294465869	-7.07333619300726
H	-1.72492444050089	1.69970643849710	-4.39461980623263
H	-2.69886335606816	0.59392540567795	-5.39223774500814
H	-3.11808247019790	0.82907189278892	-3.67955907394173
H	-5.77038187111407	2.73994723373707	-5.67180643848734
H	-5.51367343724332	1.35910068270716	-4.56190827010730
H	-4.97694317351314	1.24835231420358	-6.25762458690026
H	-1.93781833945151	2.54077075578089	-2.09290194214023
H	0.45357308741915	2.89487170547614	-1.61278372665924
H	1.64078793205645	4.87904032287667	-2.59964741629787
H	0.36409172330802	6.51542811583062	-3.97362186220857
H	-1.10032431115165	6.15166402705187	-6.43074760067553
H	-1.86345055220311	8.22154915308731	-7.58846366078013
H	-3.29514739173639	9.87759269132627	-6.37495748168912
H	-4.04981482528674	9.39399763629351	-4.04264111280508

H	-3.30842595830273	7.31088899422177	-2.89520541276570
H	-7.79935577753317	6.26413207777411	-4.79692102743373
O	-8.29461136663677	3.59264385865043	-5.35247094711275
H	-8.75479914174329	2.86463828081913	-5.81551000192823
H	-9.05678682533647	4.22092777226044	-4.94666556693925
O	-10.07567892193732	5.21018758093163	-4.67310792284997
H	-10.12673969329676	5.72403641601652	-3.84842990052889
H	-9.93952546965023	5.87085471206567	-5.44167394681457

K’

C	-8.27363365191824	5.01204365237995	-7.07528051856008
C	-8.75426317314820	3.91817633048432	-7.82241523585451
C	-6.97001970288075	5.52813195792224	-7.33395693826968
C	-7.94933841997267	3.29965679617045	-8.77919760035495
C	-6.63967495535775	3.75663622030379	-9.03627228729214
C	-6.18716146288210	4.86542595825407	-8.31658129126735
C	-5.76807595745382	3.08620877953640	-10.07263544307776
H	-5.71139006163642	1.99559433073731	-9.90549572214292
H	-4.74381804089825	3.49253608884195	-10.05721749272508
H	-6.17428161159928	3.23737681921652	-11.08942602682475
O	-9.03287293490390	5.55688972467138	-6.08501350159929
O	-4.92897999066474	7.42865596808151	-8.41615287488253
C	-5.29521618049596	7.50421284605674	-7.25740366078045
C	-6.40525742173712	6.69914742581871	-6.62271818003204
C	-6.88659824735010	7.18083113729921	-5.37673132350680
H	-9.77365149061373	3.56681392475343	-7.63405395396592
H	-8.34918307986656	2.44634209549445	-9.33879154293352
H	-8.70207351913146	3.94438293853381	-4.81758694756511
H	-5.18924567370570	5.24984756867499	-8.52916095187905

C	-6.65515633255448	8.51241001531822	-4.78538666836207
C	-6.50642478497325	9.68377720382780	-5.56225533196091
C	-6.29890327540611	10.92254747258287	-4.94531493834411
C	-6.25173310780904	11.01652688993504	-3.54634441407446
C	-6.65056237906189	8.62943502603133	-3.37459788340512
C	-6.43888871260301	9.86615641504020	-2.76121884075524
H	-6.19437112206446	11.82260264227759	-5.55937788577705
H	-6.09478584194509	11.98803594031856	-3.06708264782804
H	-6.79767573243487	7.73238707728111	-2.76279303952216
H	-6.42686233625736	9.93959607798661	-1.66914082073086
H	-6.59763507541956	9.63149213925301	-6.65155928376769
H	-4.78217868200852	8.17996858967979	-6.53688415082081
Au	-5.17656221292623	5.74067339326806	-4.92328411161832
P	-4.06014398095674	3.98404933677040	-3.88875455968880
C	-4.92307160936789	3.65832836613001	-2.21629291690024
C	-6.41879962842421	4.01432318609848	-2.36856710700691
C	-4.80840491851783	2.21117713914232	-1.70243056027668
C	-4.27976556796090	4.64260126263532	-1.21551097847949
C	-4.17463530074767	2.56071406682984	-5.15568632287827
C	-3.49875184550046	3.12022039242259	-6.42780935629438
C	-3.45431798014916	1.26552280516711	-4.74027698512999
C	-5.66541435660447	2.28398304571239	-5.43406074988288
C	-2.26845383623345	4.20080530026886	-3.58501916388868
C	-1.58390010924101	3.15570202646506	-2.92325638853959
C	-0.19341685435722	3.16216575571177	-2.78803322505638
C	0.54508358687562	4.21782346230668	-3.33736303085433
C	-0.11915288560899	5.26853843062114	-3.97549771258859
C	-1.52637193281922	5.29945756176741	-4.09613875276314

C	-2.09791806012227	6.54117514428149	-4.69333824977864
C	-1.70742176869307	6.95334239674236	-5.98361467447850
C	-2.05139152117842	8.22463374550955	-6.46260687494462
C	-2.78728674708751	9.10461032268593	-5.65548958349521
C	-3.20879843115706	8.69789415239787	-4.38057002811960
C	-2.87514944673300	7.42165720171105	-3.90508810531278
H	-6.94575070282370	3.38850259524474	-3.10525138403291
H	-6.54441832619561	5.07228921427419	-2.66198310149382
H	-6.91741345651230	3.87094044627896	-1.39303505149136
H	-5.31036745480502	1.49593872888410	-2.37375023462009
H	-5.31278821439612	2.15181977803732	-0.72120696736859
H	-3.76682385878701	1.88788394189499	-1.55108336272358
H	-4.34463585939466	5.68302942491334	-1.58143558858060
H	-3.22140066024347	4.40824113040647	-1.01939868592479
H	-4.82970771460322	4.58792882466407	-0.25893622009020
H	-2.43366759972581	3.34921256121885	-6.25279163240007
H	-4.00393743787925	4.03430537363854	-6.78401413419866
H	-3.56040998686581	2.36238764387979	-7.22928389059530
H	-2.36964290546053	1.42052531873129	-4.62890110126771
H	-3.59860950600415	0.52087425713957	-5.54406516076449
H	-3.85125820831409	0.83029224584540	-3.81076757333889
H	-6.19718359155619	3.20993610575159	-5.71948610750719
H	-6.17038862364592	1.83016838057009	-4.56472075077839
H	-5.74578441371958	1.57873550500453	-6.28059741364350
H	-2.14464427221407	2.31379355553136	-2.51234308537613
H	0.30783361055673	2.34202172399390	-2.26500547018097
H	1.63629117754998	4.23376860967215	-3.25312878343596
H	0.45271958838783	6.11510665291516	-4.36803328513895

H	-1.11054337590889	6.27764406652970	-6.60511837914894
H	-1.74230100473758	8.52875156585749	-7.46721163435491
H	-3.03502794262381	10.10673741396314	-6.01934155244013
H	-3.78559662394167	9.37956994124156	-3.74894390705601
H	-3.14771466533961	7.13033853337364	-2.88574761902326
H	-7.68787548078684	6.60689771996537	-4.90415995369463
O	-9.03802623230866	3.06576179517835	-4.53743398872793
H	-8.64583829550915	2.45879787714049	-5.19288183015815
H	-10.70684021091120	3.53507112205495	-5.18571193715586
O	-11.19757239461293	4.12620873314494	-5.81450037619069
H	-12.01042193677436	4.40622185433685	-5.35678822866082
H	-9.96343505362720	5.14898281131350	-6.07882477159752

M’

C	-8.39654876631876	6.11110324458145	-6.56560818774105
C	-9.54718049457031	5.48720826042359	-7.03658046902591
C	-7.10939688257403	5.76346508339689	-7.00475402662993
C	-9.37588940628192	4.46513678916490	-7.99293452050194
C	-8.09920578006161	4.10879143969490	-8.48816513167614
C	-6.95533156311467	4.77768944687970	-7.98753784945379
C	-7.95641424845490	3.06191348308276	-9.56933252779455
H	-8.79350710313650	2.34482441089872	-9.55423670872530
H	-7.01095803289722	2.50314160334539	-9.46716839284927
H	-7.94712387543550	3.54005152604320	-10.56660839059937
O	-8.36663059720911	7.01050771462359	-5.51294353373900
O	-4.32174785856662	6.31985292090818	-7.76888454037862
C	-4.86748595942926	6.94969990803929	-6.86754510688780
C	-6.11810746283916	6.51149837306139	-6.17873712123215
C	-6.98259515100240	7.59395243713971	-5.48357283414184

H	-10.53730961959394	5.75997882970781	-6.66170345090964
H	-10.26036806940810	3.94560309388442	-8.37635958896341
H	-8.06884124369906	3.53446972364288	-6.24284858769030
H	-5.95275088389490	4.51727557733066	-8.33627937707806
C	-7.01684270361605	8.94582905572151	-6.15582235782258
C	-6.97208309519818	9.07536028521468	-7.55762252016534
C	-7.03494539895353	10.34258237042128	-8.15015652037586
C	-7.13906896634246	11.49149724532383	-7.35155315124629
C	-7.12771604168284	10.10251517764371	-5.36366615742261
C	-7.18625848873670	11.37080736874322	-5.95532079144625
H	-6.99952416614596	10.43428843411634	-9.24040541147464
H	-7.18319656456842	12.48094879491473	-7.81799715697364
H	-7.17274990541617	10.00550496766395	-4.27244727004389
H	-7.27013623871707	12.26444751738386	-5.32837390116838
H	-6.88945994462732	8.18747623127825	-8.19312870298584
H	-4.42465879309156	7.89817994642220	-6.48397111823009
Au	-5.14307721270394	5.11394769842726	-4.83965258463634
P	-3.86326607121833	3.37632684094957	-3.86758183779419
C	-4.73015047425423	2.42548265309658	-2.44969732551225
C	-6.24555864828586	2.43804707617889	-2.73353038843642
C	-4.27551130321632	0.96397406792810	-2.27253907919124
C	-4.46762117121521	3.22361859572842	-1.15494532899764
C	-3.40536207382819	2.30183734186584	-5.38275335542567
C	-2.83365992580701	3.30603601223095	-6.41188394549360
C	-2.34891372245679	1.21060625297964	-5.12541304633566
C	-4.69384495320566	1.66465194306866	-5.93775772103267
C	-2.24754472307748	3.94395893940677	-3.19956871328526
C	-1.36729699662167	2.96522667150144	-2.68441177224326

C	-0.06927850390960	3.28292884978565	-2.27702065301956
C	0.38391327837980	4.60258865870580	-2.39382967446989
C	-0.48174139296278	5.58775024600398	-2.87396037055369
C	-1.80855664427995	5.29326328213218	-3.26249476547493
C	-2.64719930860601	6.47307253583175	-3.61764422058002
C	-2.19050728282800	7.39960649258373	-4.57827343023158
C	-2.83219057278328	8.63375331525085	-4.74339163201704
C	-3.94403284184967	8.96586310498245	-3.95566587032467
C	-4.42648745759301	8.04405283297854	-3.01615693597479
C	-3.78874842443352	6.80492066469281	-2.84958843975453
H	-6.50428698822053	1.92264211316240	-3.67126296174272
H	-6.62136804982993	3.47502645204596	-2.78602847645997
H	-6.77195034686858	1.92413361091548	-1.90895409895747
H	-4.49513087850477	0.35043347511307	-3.16083658371707
H	-4.83313509176128	0.52750555597228	-1.42393433558840
H	-3.20432148295287	0.87329356306912	-2.03496922471425
H	-4.78630788499065	4.27585730604709	-1.25939070338703
H	-3.40601262815535	3.21145420883403	-0.86276158293878
H	-5.05652667995511	2.77521244269276	-0.33443059312791
H	-1.95729759017234	3.84186910128695	-6.00833257778438
H	-3.58230971181929	4.05340161985055	-6.73022355185442
H	-2.50666978008163	2.74917068297558	-7.30839027244908
H	-1.37492567754685	1.64399131049044	-4.85065245553823
H	-2.20429076047959	0.65016601088239	-6.06684721699528
H	-2.65135736595610	0.48618323435055	-4.35413952726337
H	-5.46322971781221	2.42628561462056	-6.15051457220426
H	-5.12075784600917	0.91044382124160	-5.25573616999541
H	-4.45949890576223	1.15775763807093	-6.89095377367537

H	-1.69598016458358	1.92777523658342	-2.60671089629896
H	0.58417396432148	2.50037158861892	-1.87939122014138
H	1.40133332980614	4.86966559532380	-2.09136123663042
H	-0.14751092017238	6.62886135803959	-2.91799285872639
H	-1.31423663894108	7.15389527318124	-5.18633497984250
H	-2.45379281903001	9.34537496575301	-5.48411689645759
H	-4.42714453538135	9.94010157878097	-4.07313778170611
H	-5.27877169666281	8.30152618909094	-2.37817283811701
H	-4.11477418945973	6.12688025005151	-2.05513304773944
H	-6.76233206978242	7.72259312080552	-4.41566660238242
O	-7.75719353190665	3.42176671066973	-5.31208667706377
H	-6.79133698069015	3.88337206319952	-5.27918346445769
H	-8.33534424904073	4.08896294015418	-4.66600081269664
O	-8.94173963367748	5.04000636841053	-3.87106422565622
H	-8.66948222459370	5.09730447003060	-2.93767887635555
H	-8.77297052098493	5.93891919267694	-4.29824241117503

10. ^{1}H ,^{13}C spectral data of Compounds

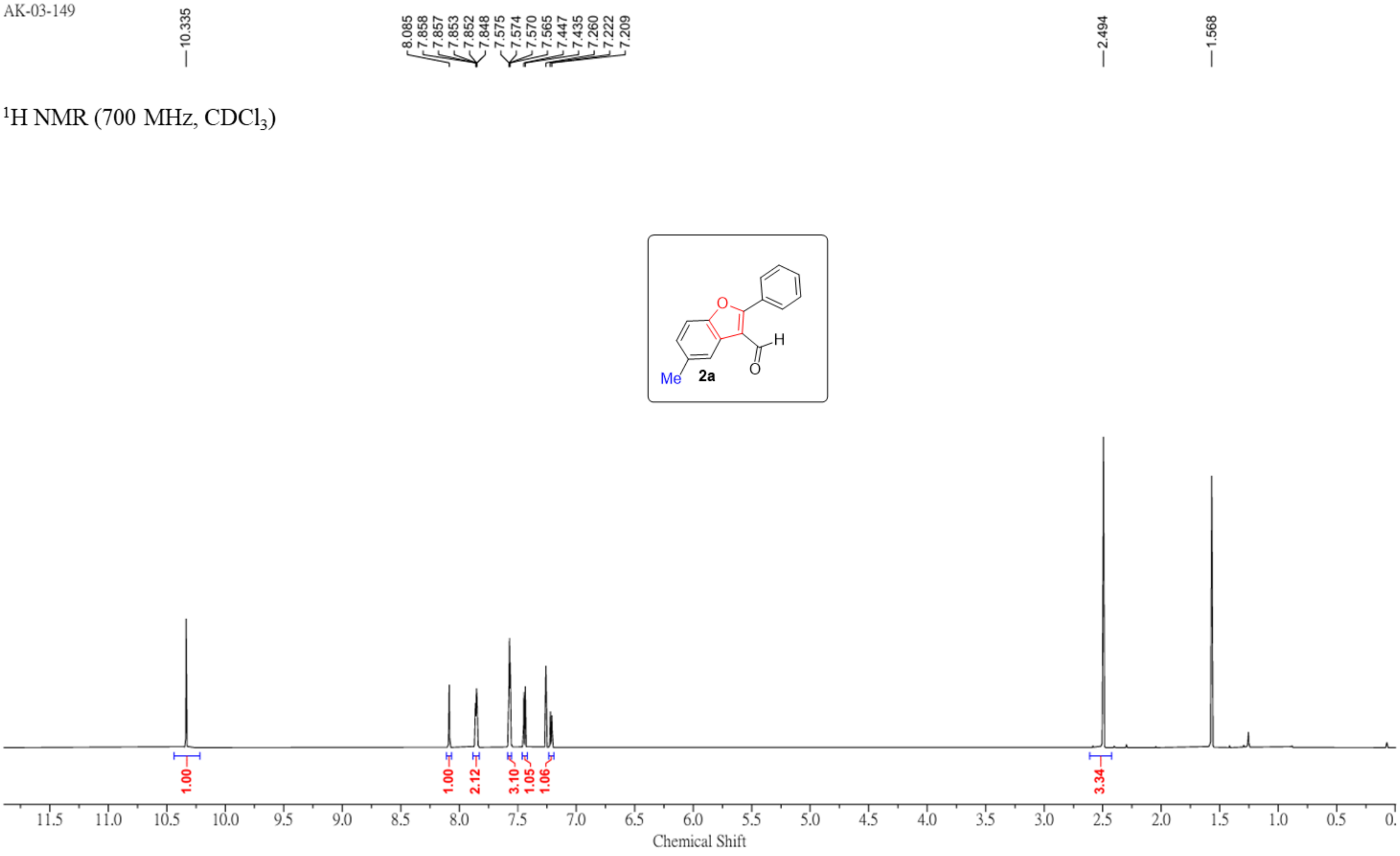

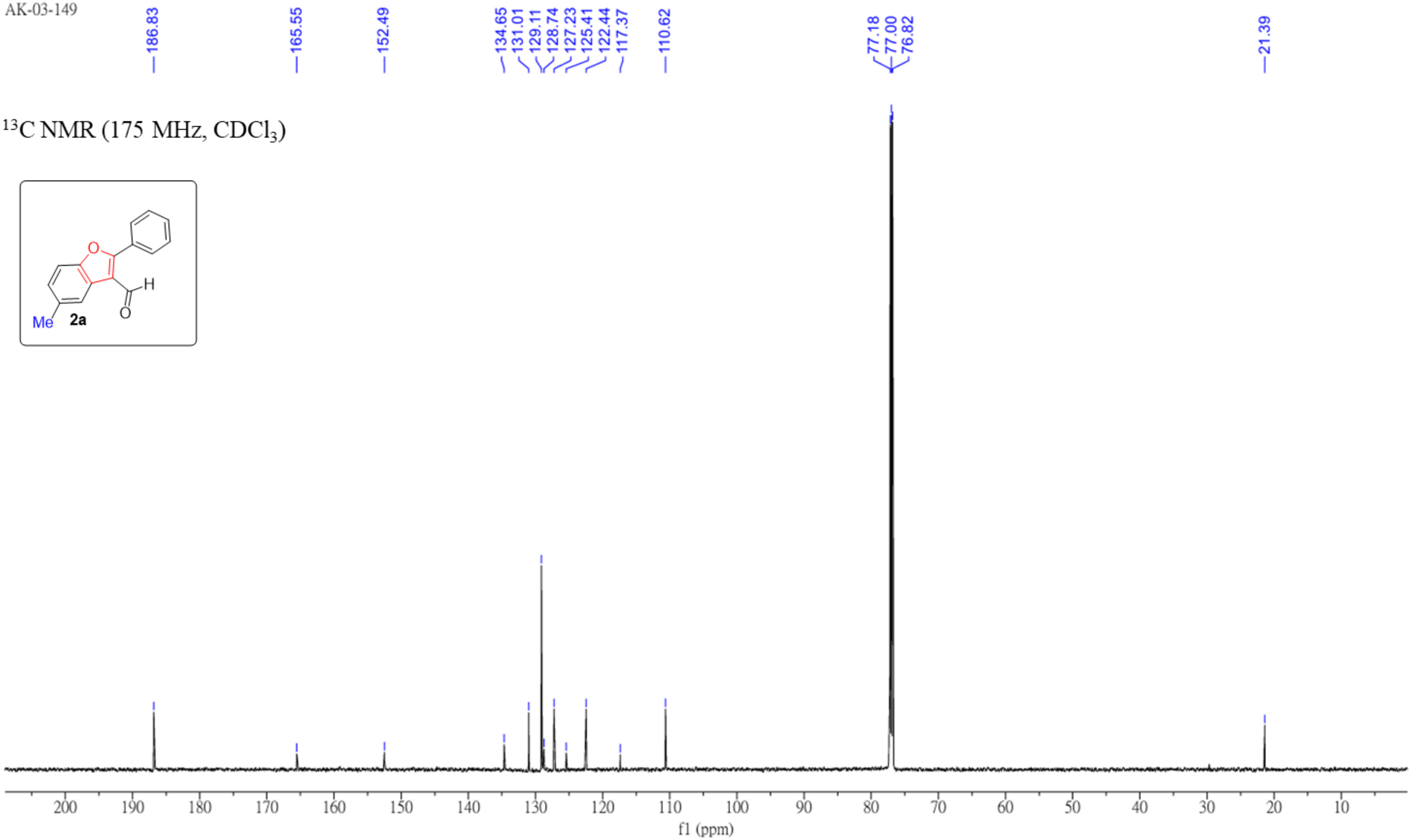
AK-03-149
186.83
165.55
152.49
134.65
131.01
129.11
128.74
127.23
125.41
122.44
117.37
110.62
77.18
77.00
76.82
21.39
^{13}C NMR (175 MHz, $CDCl_3$)
Me
2a
O
H
200
190
180
170
160
150
140
130
120
110
100
90
80
70
60
50
40
30
20
10
f1 (ppm)

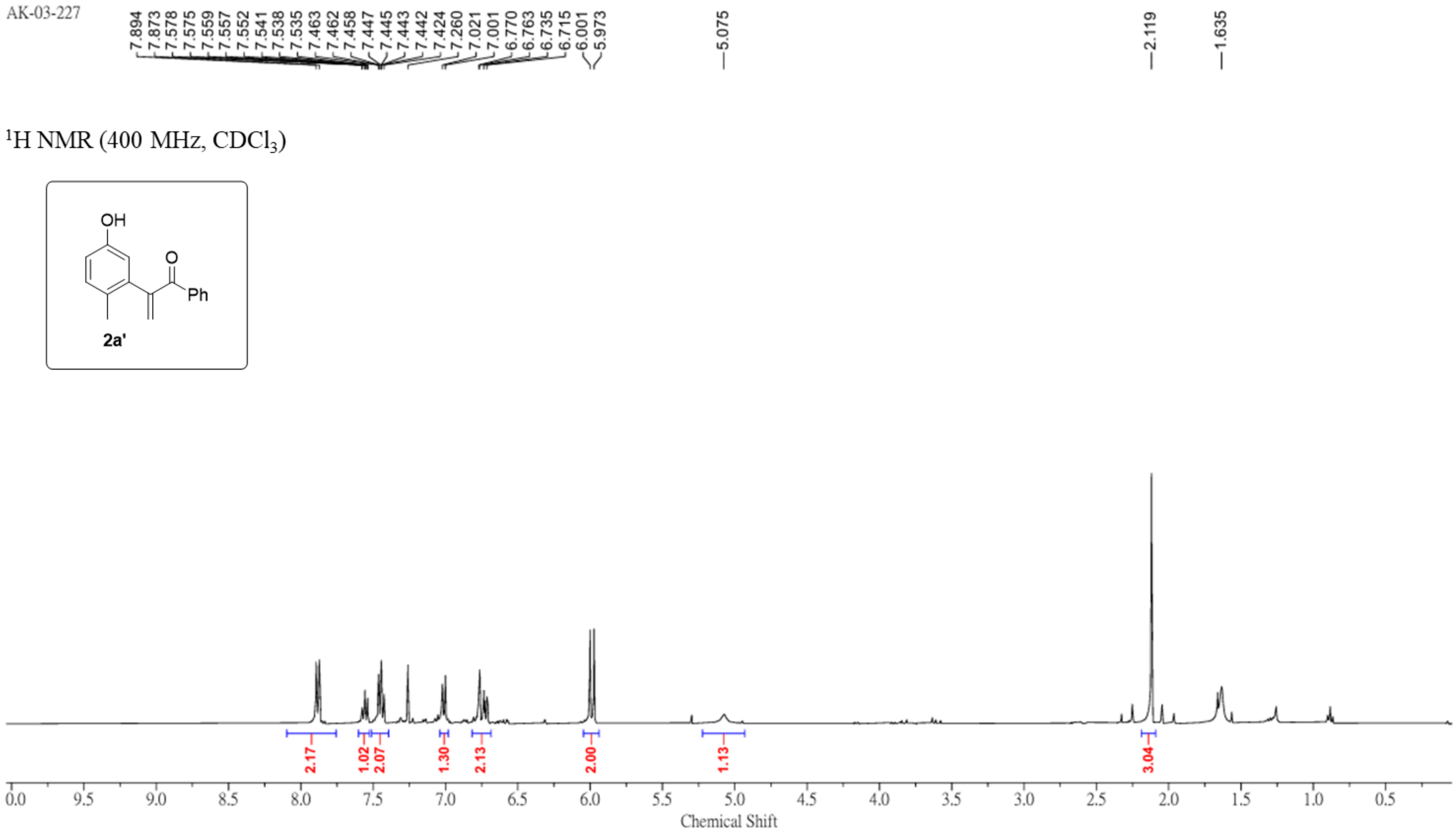
AK-03-227
7.894
7.873
7.578
7.575
7.559
7.557
7.552
7.541
7.538
7.535
7.463
7.462
7.458
7.447
7.445
7.443
7.442
7.424
7.260
7.021
7.001
6.770
6.763
6.735
6.715
6.001
5.973
5.075
2.119
1.635
1H NMR (400 MHz, CDCl3)
OH
O
Ph
2a'
2.17
1.02
2.07
1.30
2.13
2.00
1.13
3.04
0.0
9.5
9.0
8.5
8.0
7.5
7.0
6.5
6.0
5.5
5.0
4.5
4.0
3.5
3.0
2.5
2.0
1.5
1.0
0.5
Chemical Shift

AK-03-227

^{13}C NMR (100 MHz, $CDCl_3$)

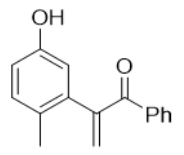

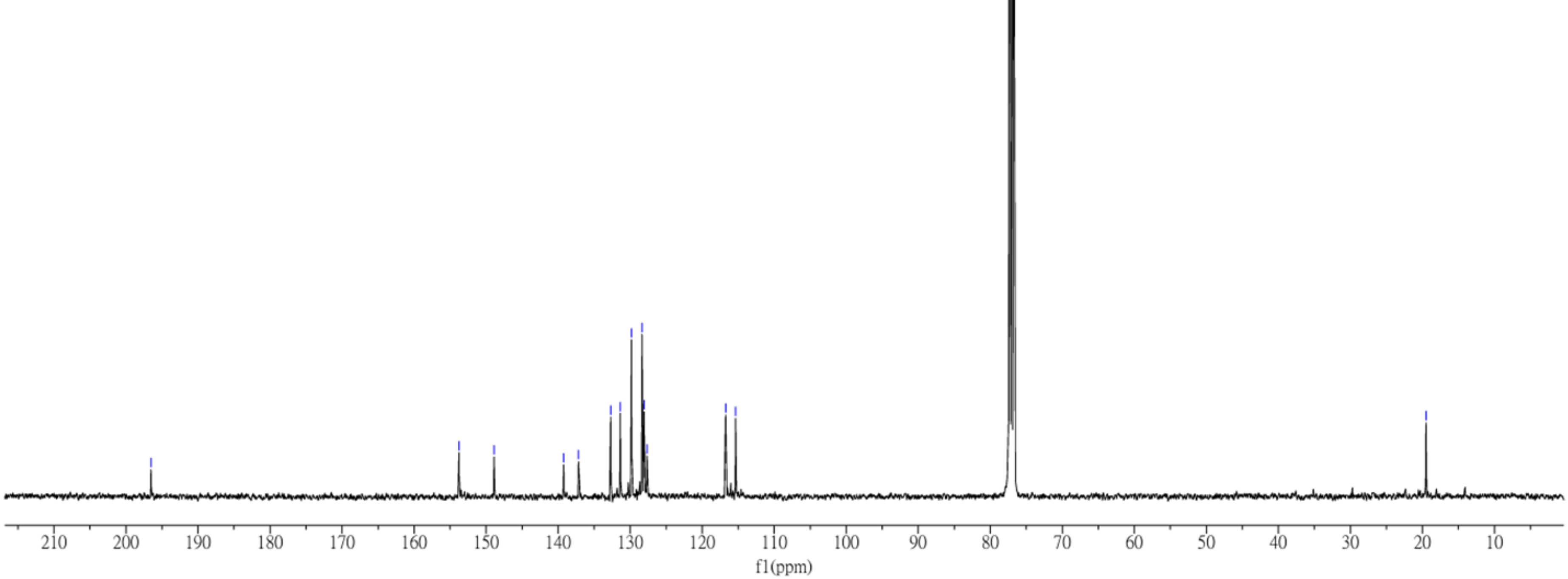

AK-03-151-POME

^1H NMR (700 MHz, $CDCl_3$)

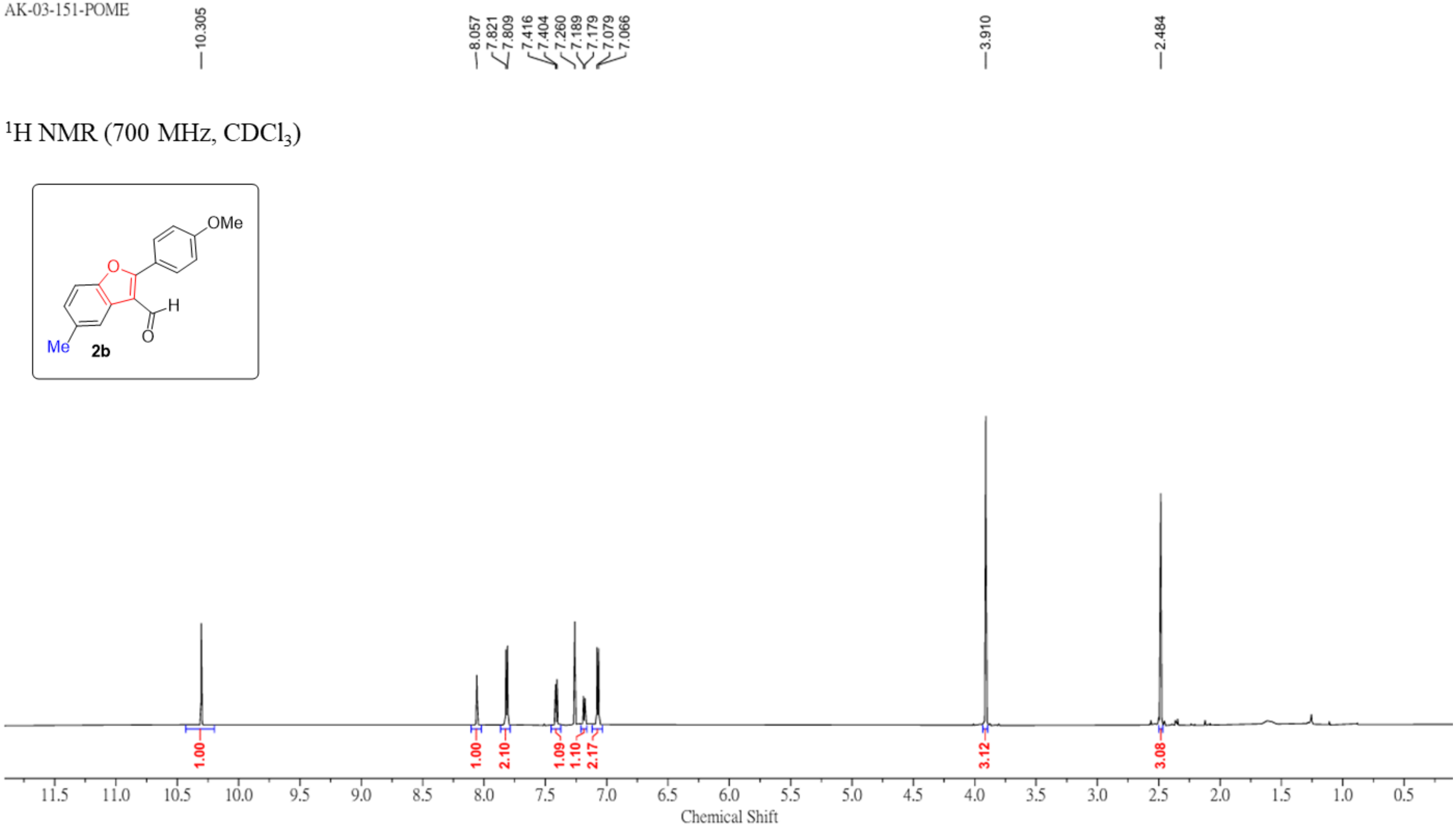

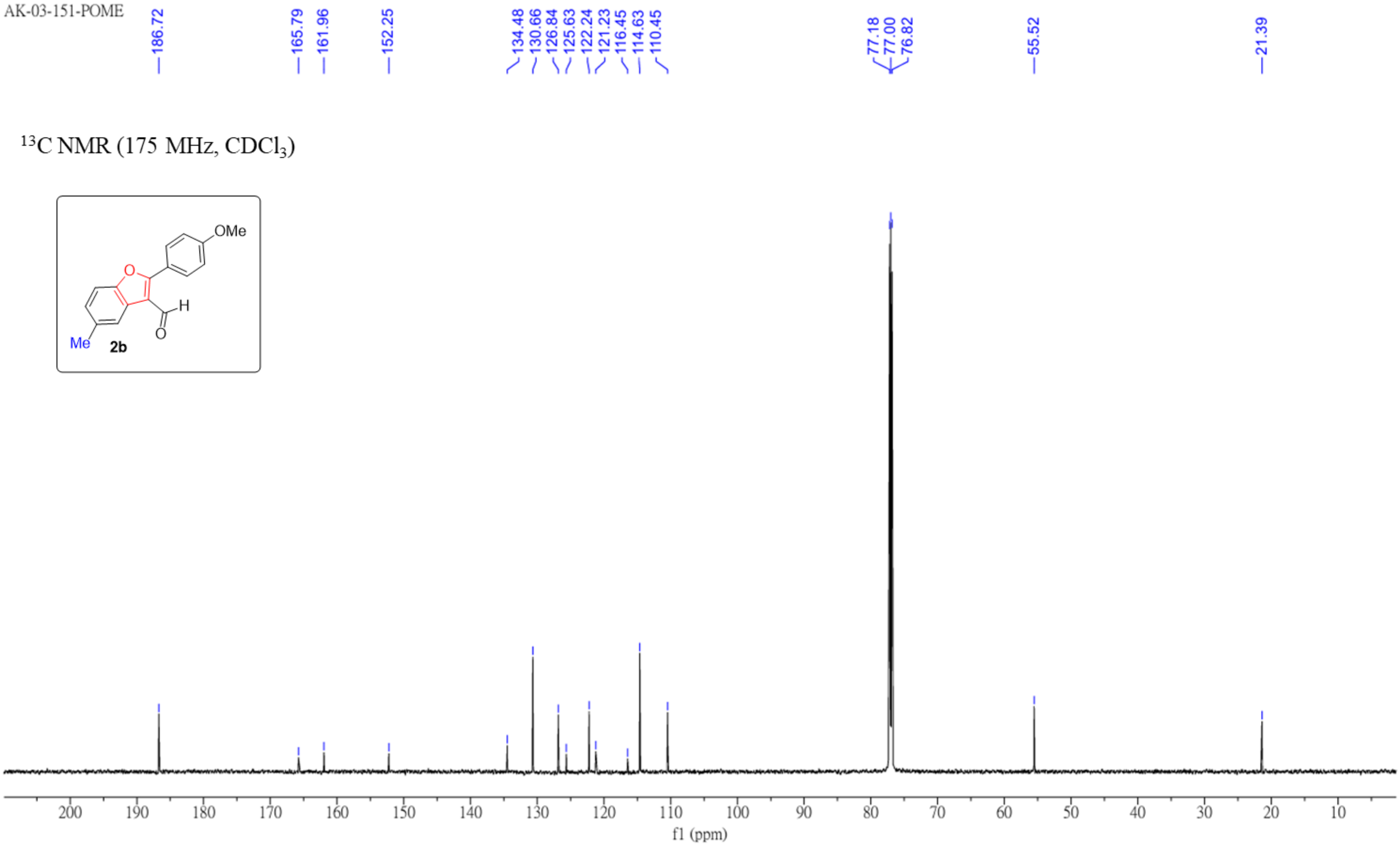
AK-03-151-POME
186.72
165.79
161.96
152.25
134.48
130.66
126.84
125.63
122.24
121.23
116.45
114.63
110.45
77.18
77.00
76.82
55.52
21.39
^{13}C NMR (175 MHz, $CDCl_3$)
OMe
O
H
O
Me
2b
200 190 180 170 160 150 140 130 120 110 100 90 80 70 60 50 40 30 20 10
f1 (ppm)

AK-03-154

^1H NMR (500 MHz, $CDCl_3$)

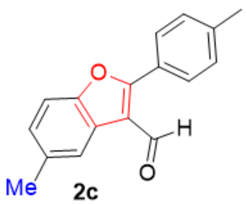

2c

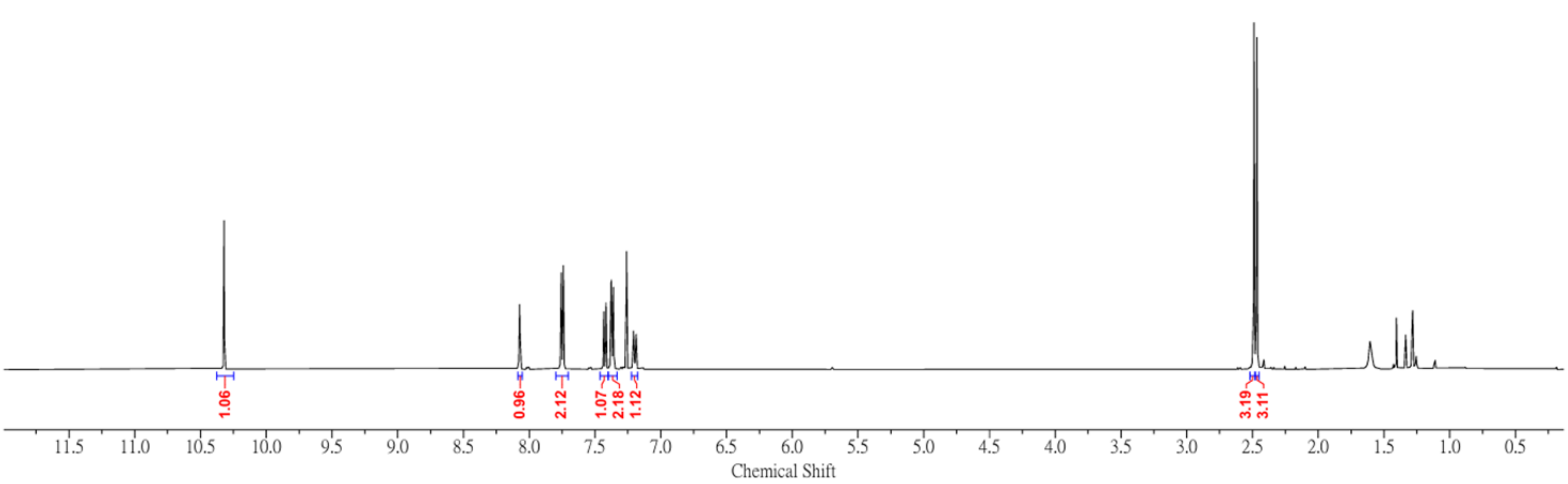

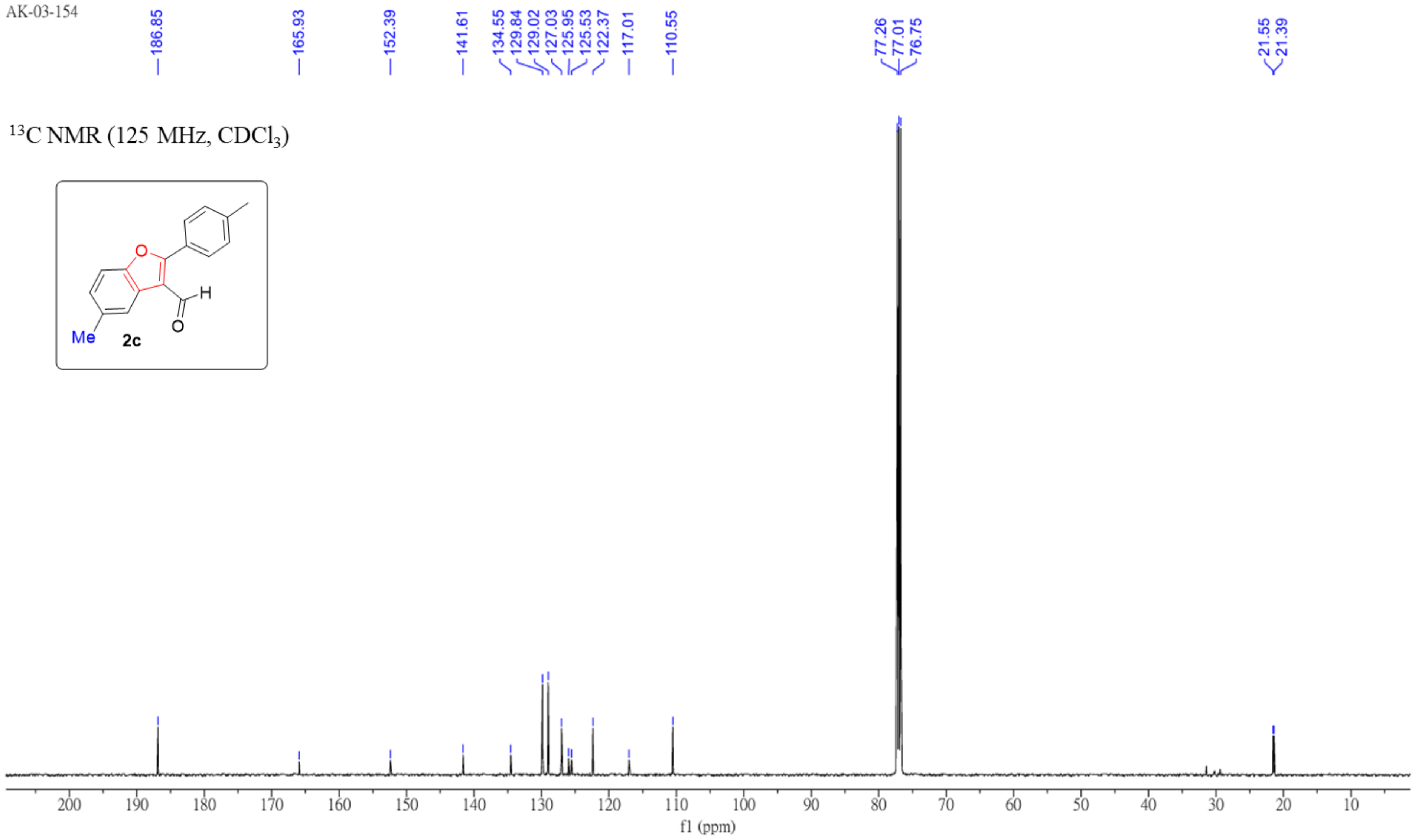
AK-03-154
^{13}C NMR (125 MHz, CDCl$_3$)
186.85
165.93
152.39
141.61
134.55
129.84
129.02
127.03
125.95
125.53
122.37
117.01
110.55
77.26
77.01
76.75
21.55
21.39
Me
2c
O
H
f1 (ppm)

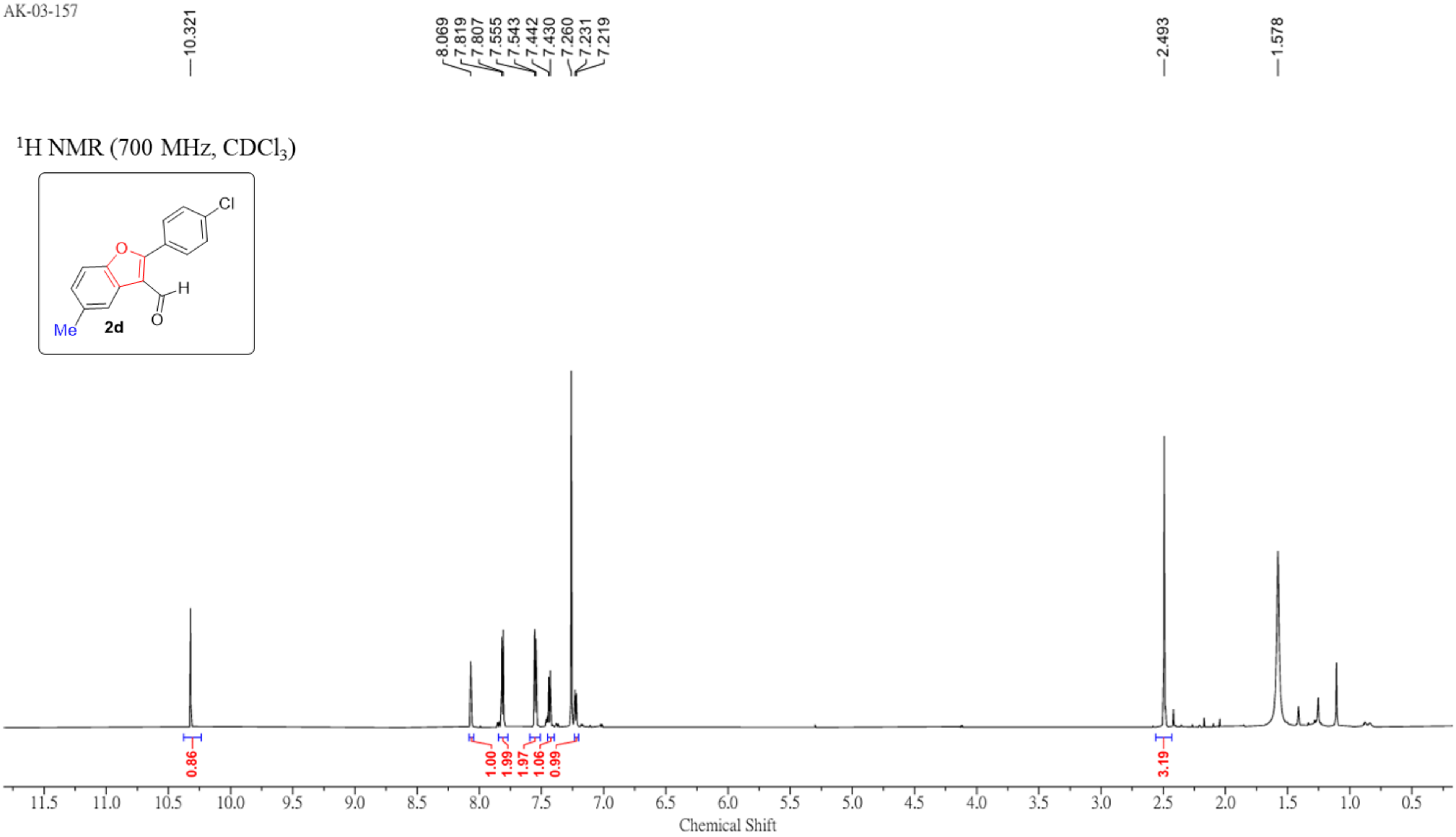
AK-03-157
1H NMR (700 MHz, CDCl3)
Cl
O
H
O
Me
2d
10.321
8.069
7.819
7.807
7.555
7.543
7.442
7.430
7.260
7.231
7.219
2.493
1.578
0.86
1.00
1.99
1.97
1.06
0.99
3.19
11.5
11.0
10.5
10.0
9.5
9.0
8.5
8.0
7.5
7.0
6.5
6.0
5.5
5.0
4.5
4.0
3.5
3.0
2.5
2.0
1.5
1.0
0.5
Chemical Shift

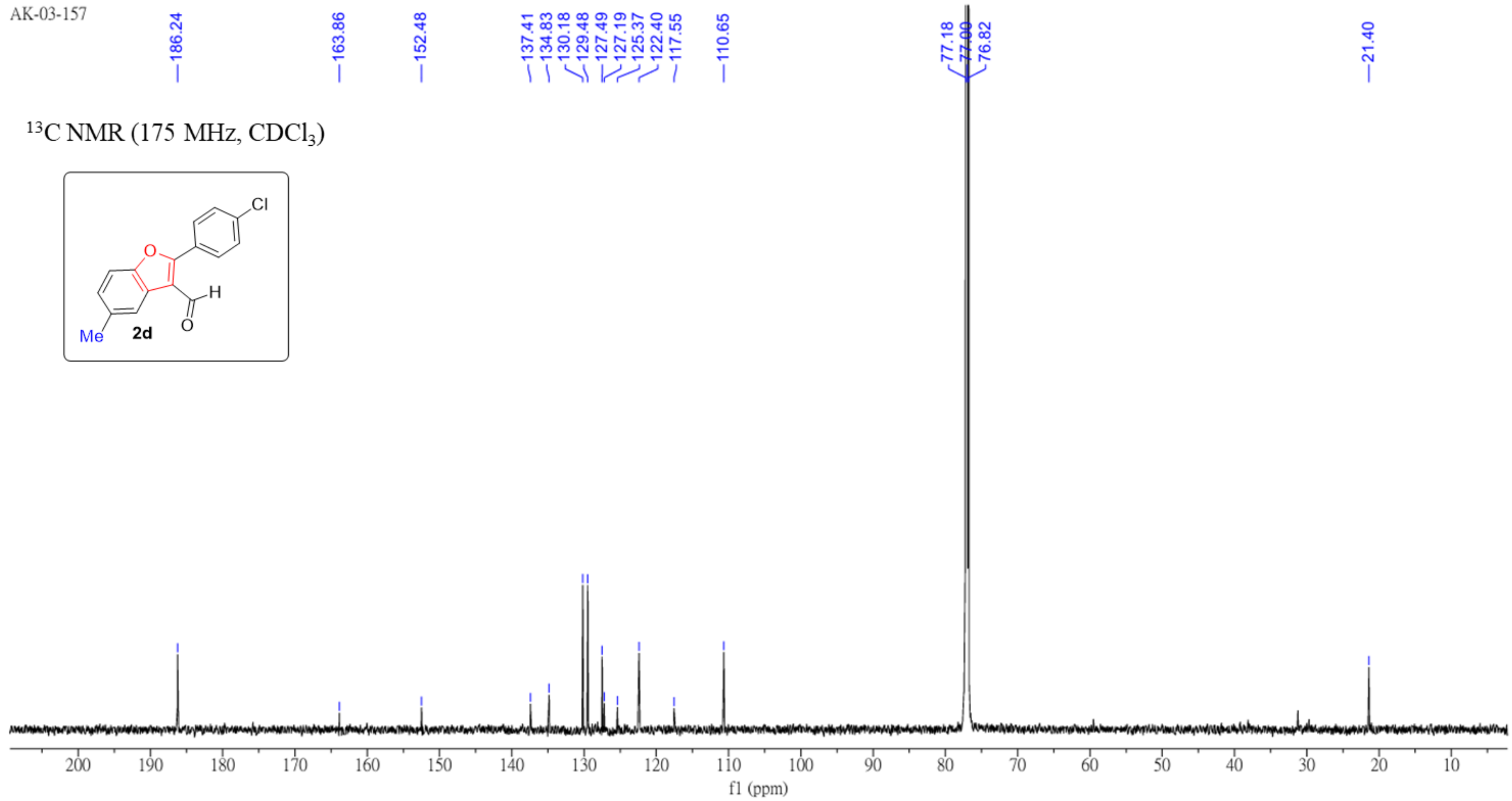
AK-03-157
186.24
163.86
152.48
137.41
134.83
130.18
129.48
127.49
127.19
125.37
122.40
117.55
110.65
77.18
77.00
76.82
21.40
13C NMR (175 MHz, CDCl3)
Cl
O
H
O
Me
2d
f1 (ppm)

AK-03-160

^{1}H NMR (500 MHz, $CDCl_3$)

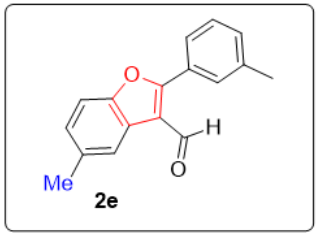

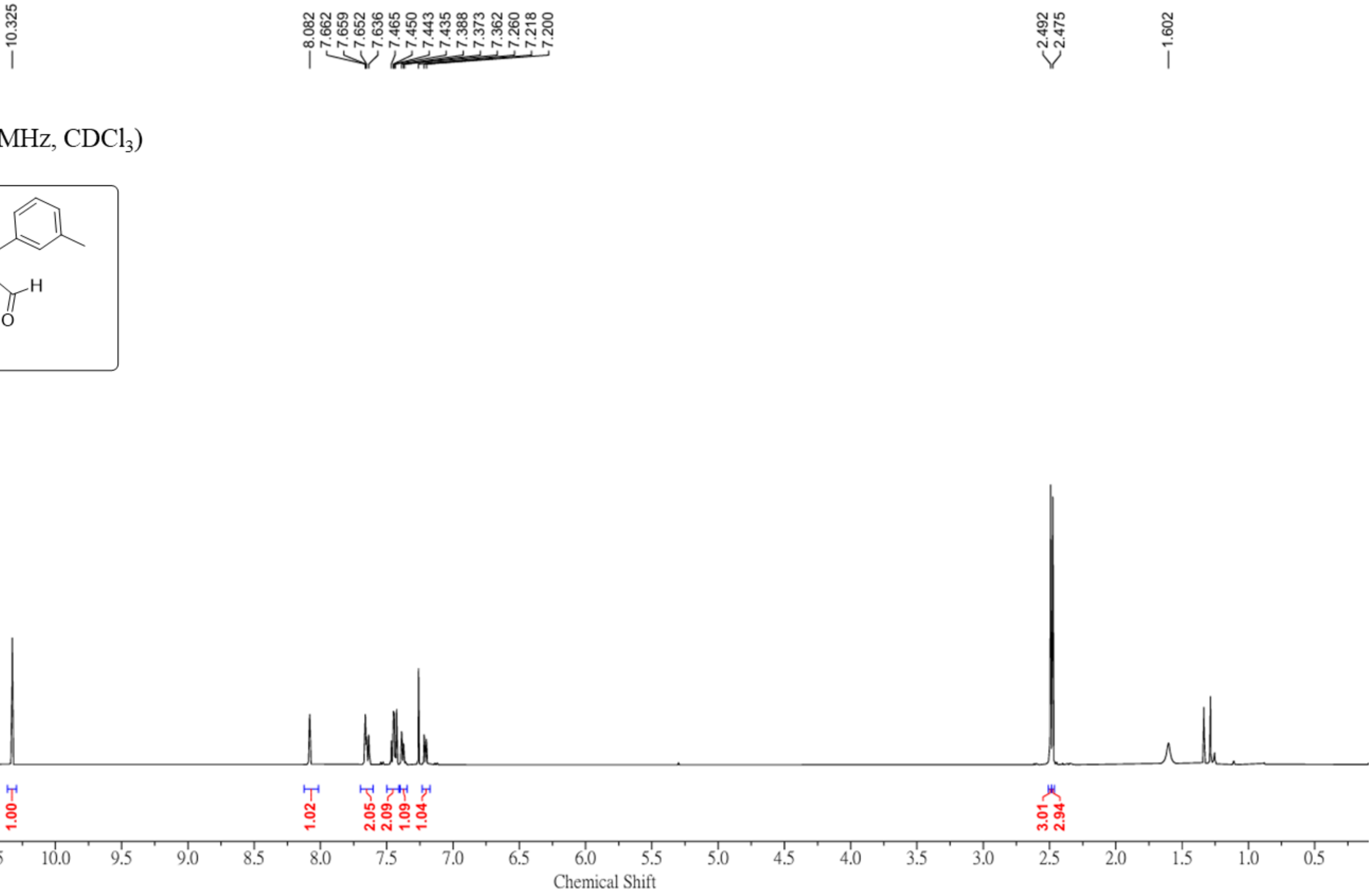

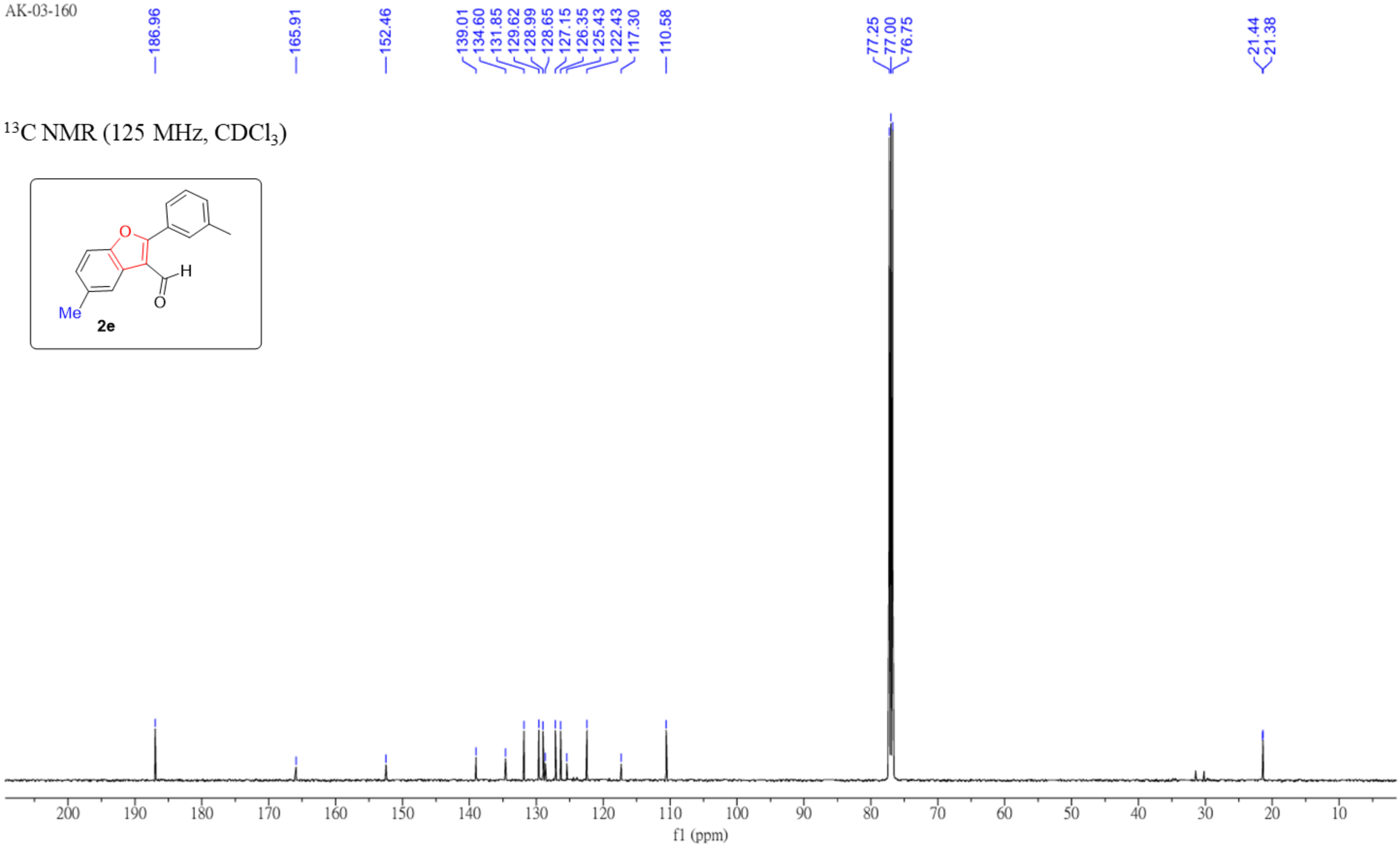
AK-03-160
186.96
165.91
152.46
139.01
134.60
131.85
129.62
128.99
128.65
127.15
126.35
125.43
122.43
117.30
110.58
77.25
77.00
76.75
21.44
21.38
13C NMR (125 MHz, CDCl3)
O
H
O
Me
2e
200
190
180
170
160
150
140
130
120
110
100
90
80
70
60
50
40
30
20
10
f1 (ppm)

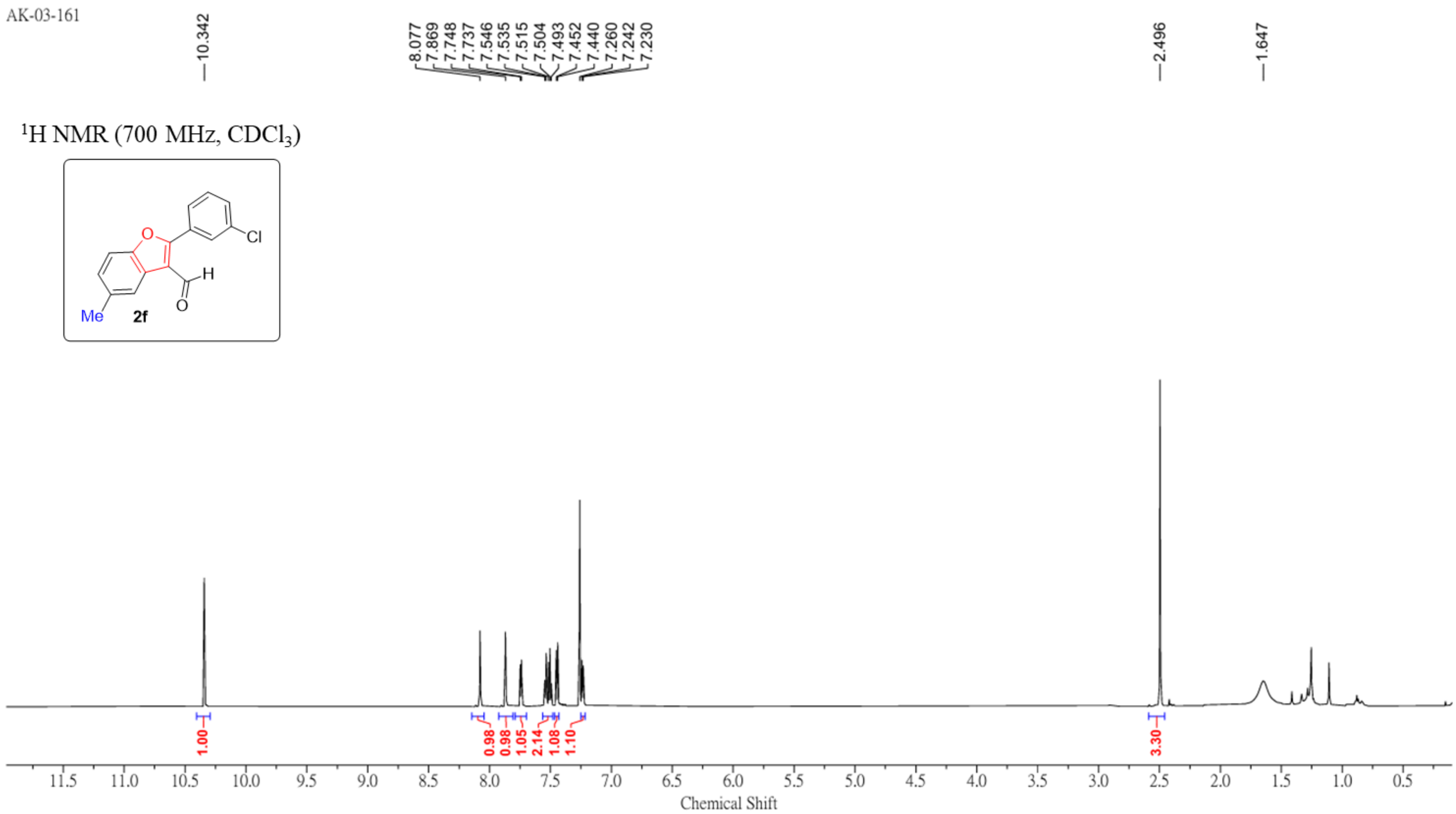
AK-03-161
10.342
8.077
7.869
7.748
7.737
7.546
7.535
7.515
7.504
7.493
7.452
7.440
7.260
7.242
7.230
2.496
1.647
¹H NMR (700 MHz, CDCl₃)
O
Cl
H
O
Me
2f
1.00
0.98
0.98
1.05
2.14
1.08
1.10
3.30
11.5
11.0
10.5
10.0
9.5
9.0
8.5
8.0
7.5
7.0
6.5
6.0
5.5
5.0
4.5
4.0
3.5
3.0
2.5
2.0
1.5
1.0
0.5
Chemical Shift

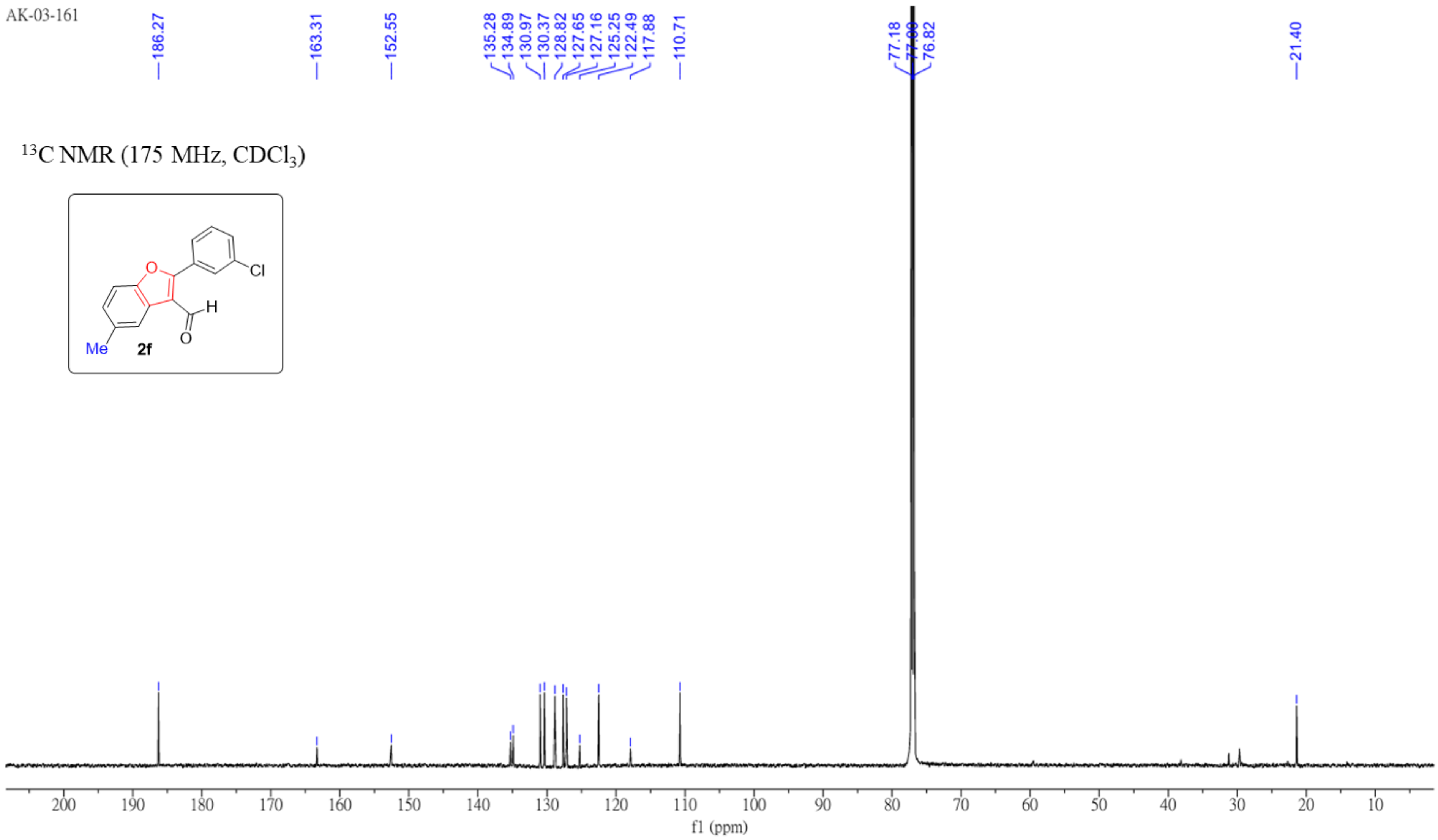

AK-03-161
186.27
163.31
152.55
135.28
134.89
130.97
130.37
128.82
127.65
127.16
125.25
122.49
117.88
110.71
77.18
76.82
21.40
^{13}C NMR (175 MHz, $CDCl_3$)
O
Cl
H
O
Me
2f
200
190
180
170
160
150
140
130
120
110
100
90
80
70
60
50
40
30
20
10
f1 (ppm)

AK-03-165-HP

^{1}H NMR (700 MHz, $CDCl_3$)

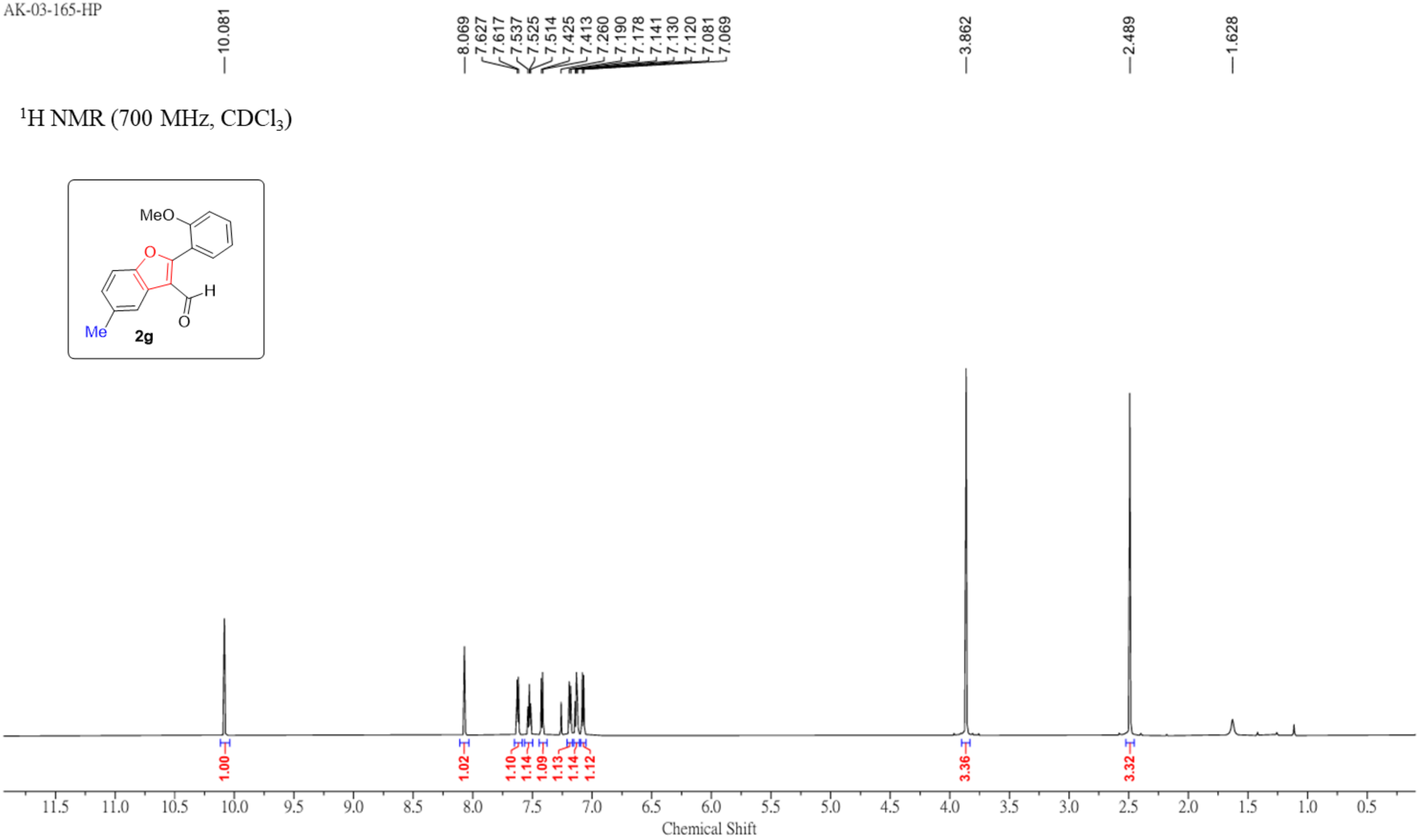

AK-03-165-HP

^{13}C NMR (175 MHz, $CDCl_3$)

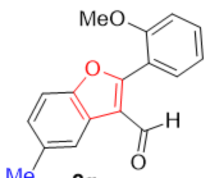

2g

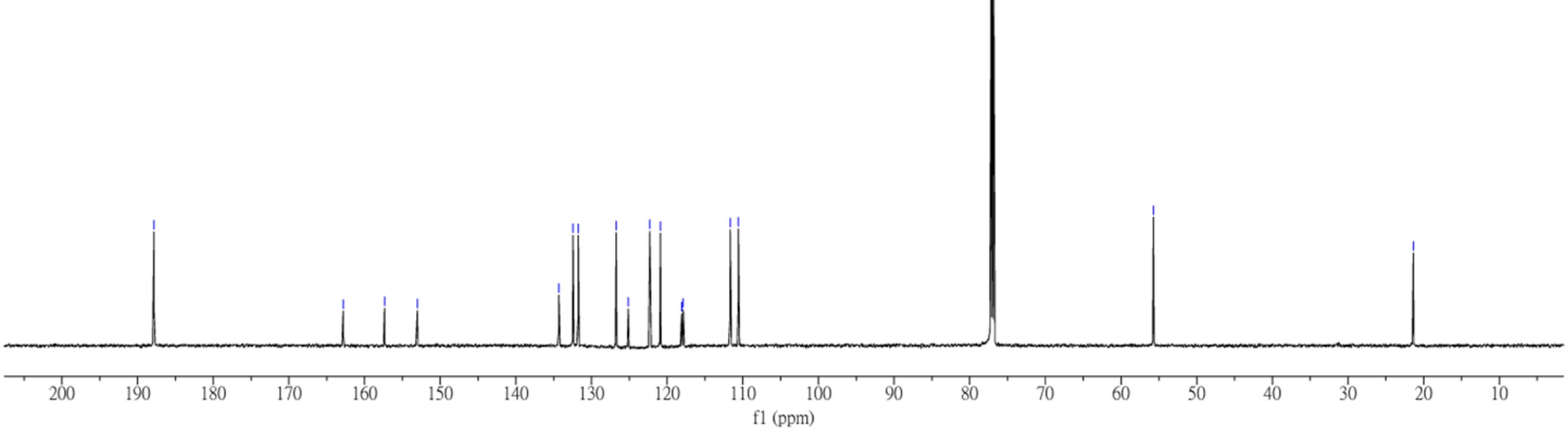

AK-03-172

^1H NMR (700 MHz, $CDCl_3$)

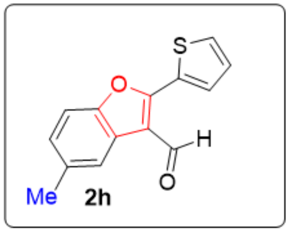

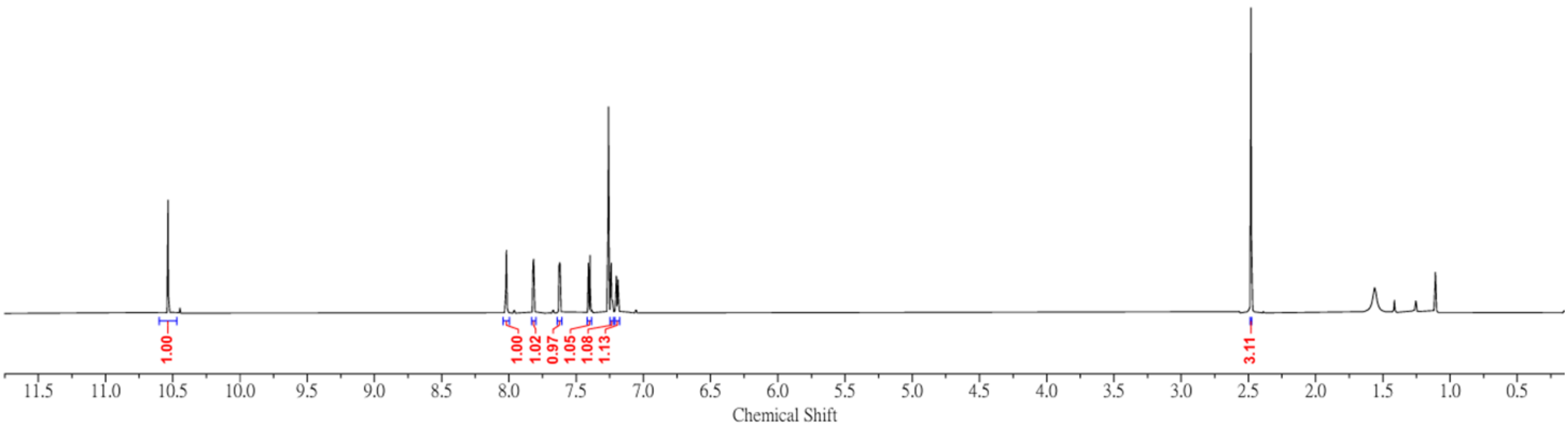

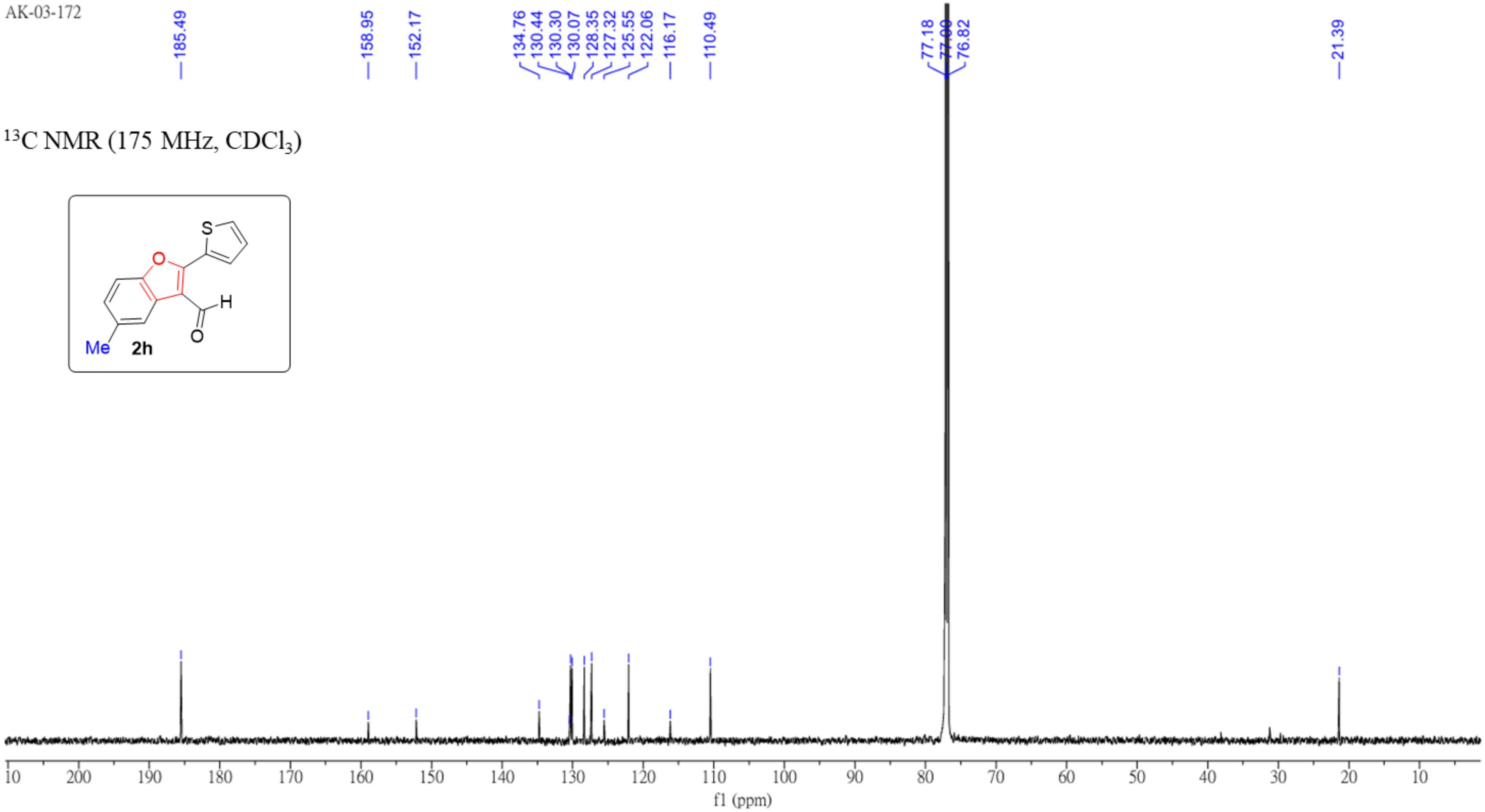
AK-03-172
^{13}C NMR (175 MHz, CDCl$_3$)
185.49
158.95
152.17
134.76
130.44
130.30
130.07
128.35
127.32
125.55
122.06
116.17
110.49
77.18
77.00
76.82
21.39
S
O
H
O
Me
2h
f1 (ppm)

AK-03-183

^{1}H NMR (700 MHz, $CDCl_3$)

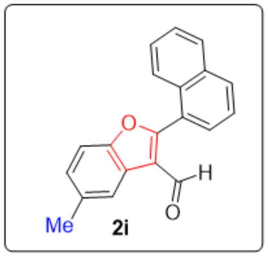

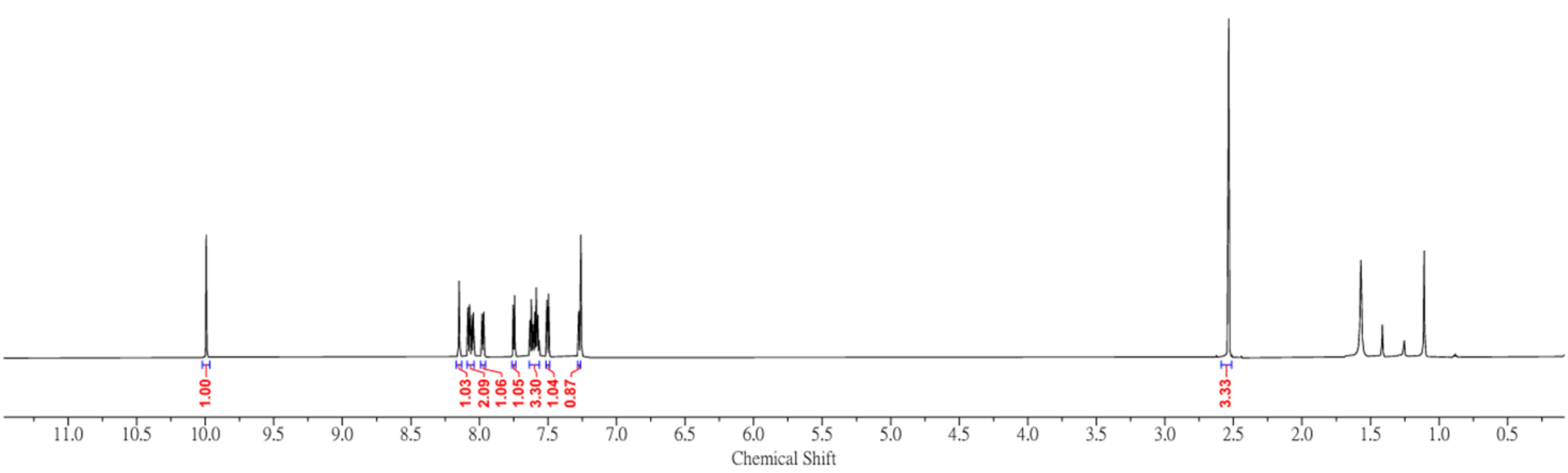

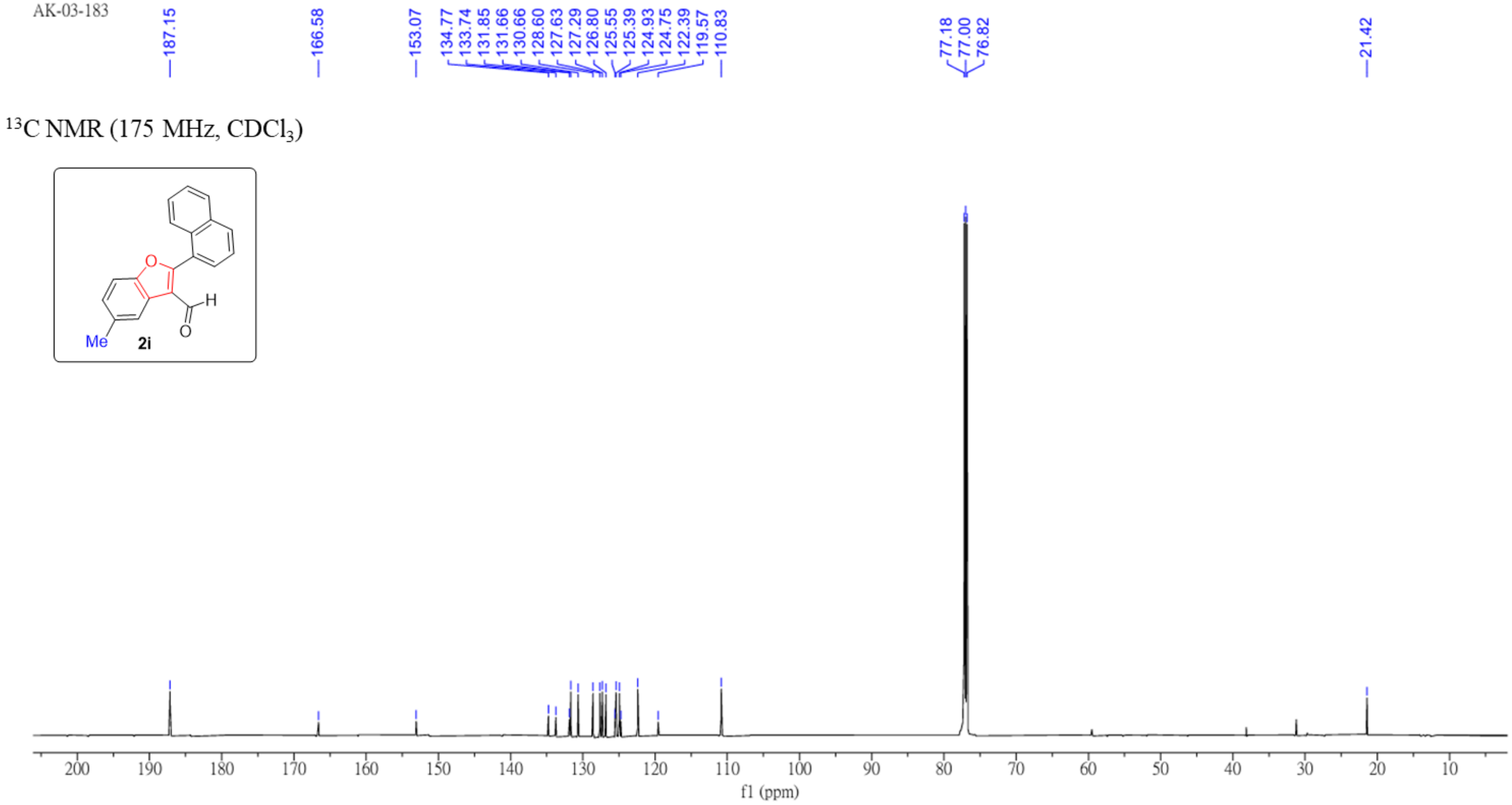
AK-03-183
187.15
166.58
153.07
134.77
133.74
131.85
131.66
130.66
128.60
127.63
127.29
126.80
125.55
125.39
124.93
124.75
122.39
119.57
110.83
77.18
77.00
76.82
21.42
^{13}C NMR (175 MHz, $CDCl_3$)
O
H
O
Me
2i
200 190 180 170 160 150 140 130 120 110 100 90 80 70 60 50 40 30 20 10
f1 (ppm)

AK-03-176

^{1}H NMR (700 MHz, $CDCl_3$)

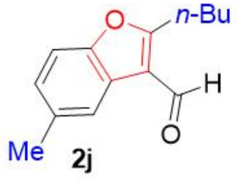

2j

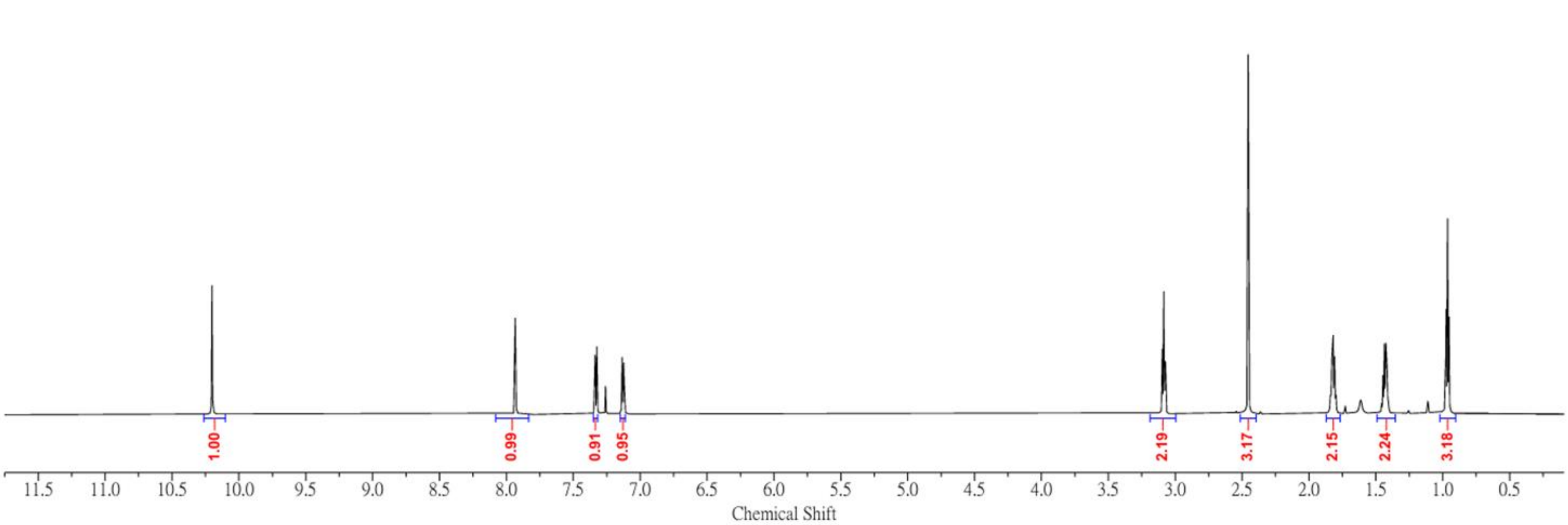

AK-03-176

^{13}C NMR (175 MHz, $CDCl_3$)

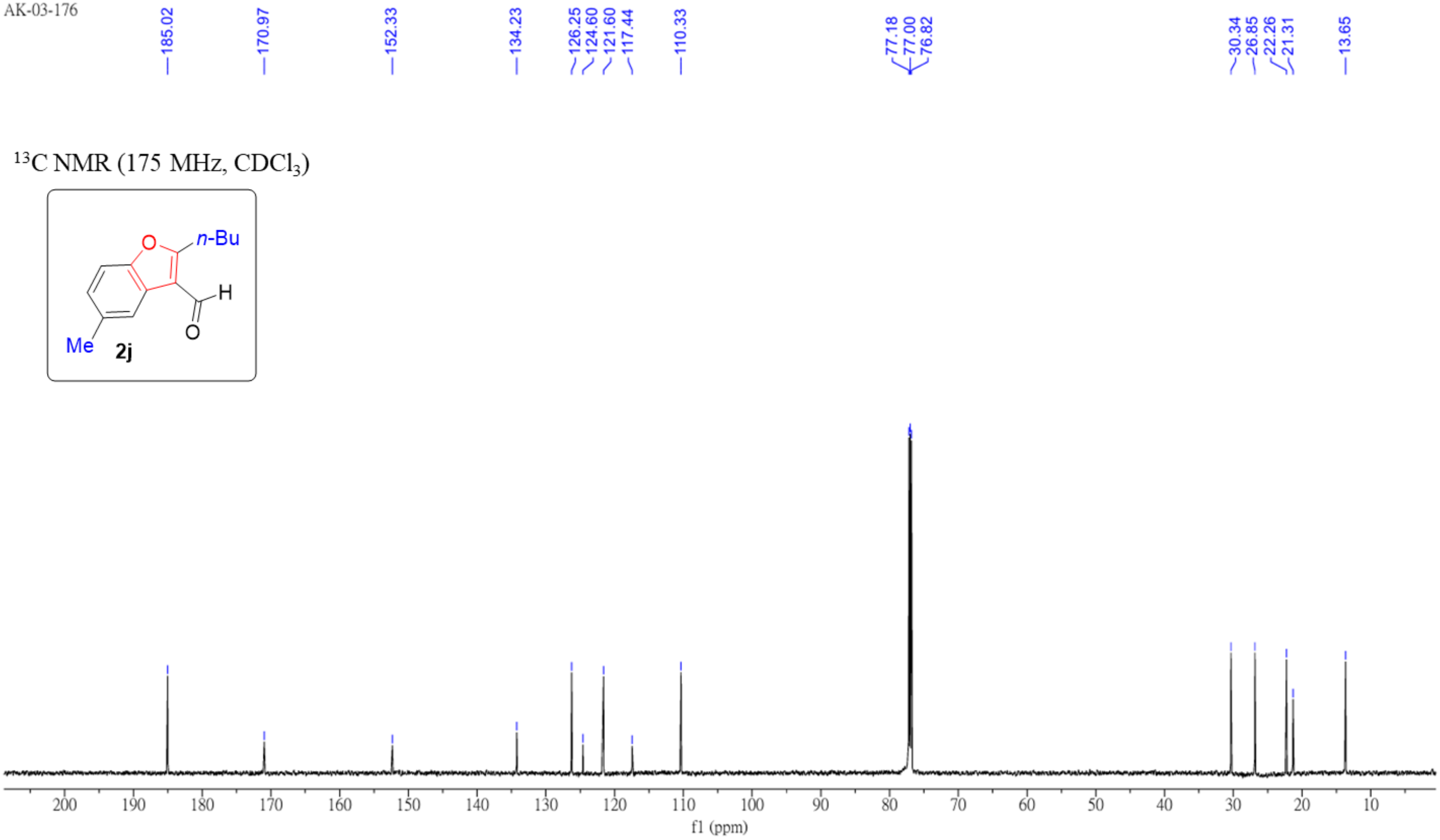

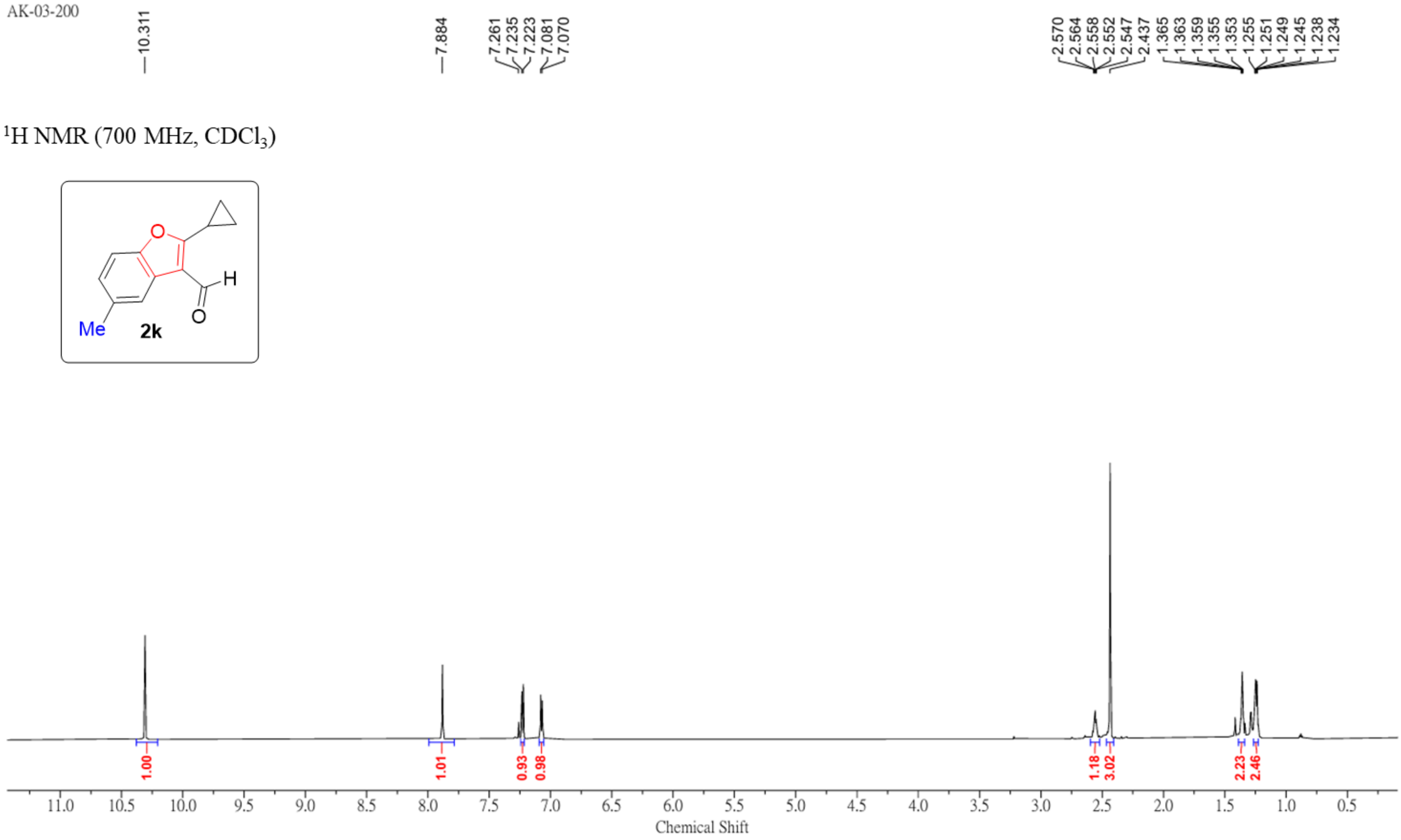

AK-03-200
10.311
7.884
7.261
7.235
7.223
7.081
7.070
2.570
2.564
2.558
2.552
2.547
2.437
1.365
1.363
1.359
1.355
1.353
1.255
1.251
1.249
1.245
1.238
1.234
¹H NMR (700 MHz, CDCl₃)
O
H
O
Me
2k
1.00
1.01
0.93
0.98
1.18
3.02
2.23
2.46
11.0
10.5
10.0
9.5
9.0
8.5
8.0
7.5
7.0
6.5
6.0
5.5
5.0
4.5
4.0
3.5
3.0
2.5
2.0
1.5
1.0
0.5
Chemical Shift

AK-03-200

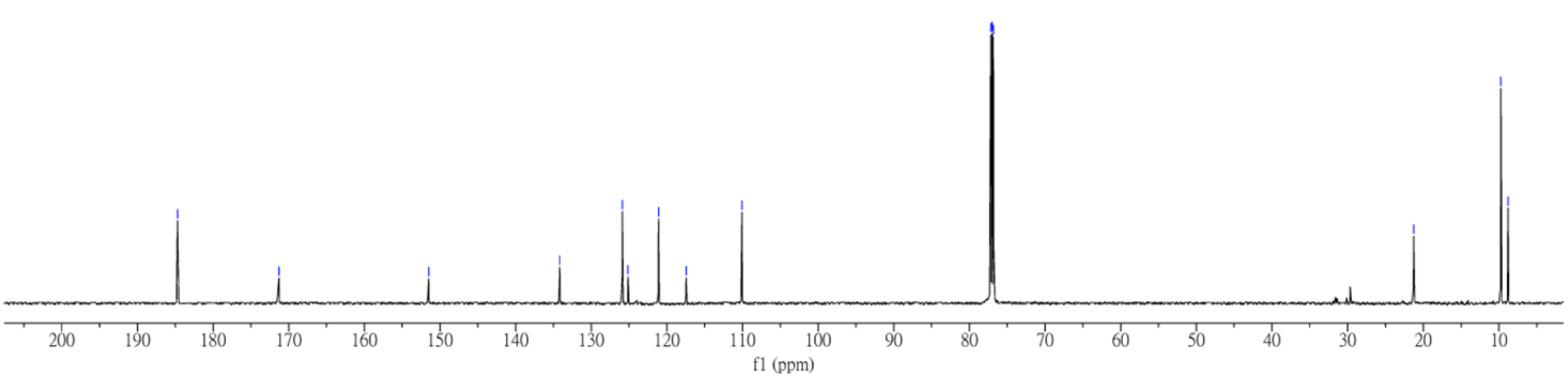

^{13}C NMR (175 MHz, $CDCl_3$)

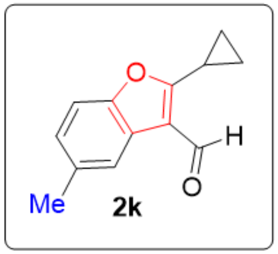

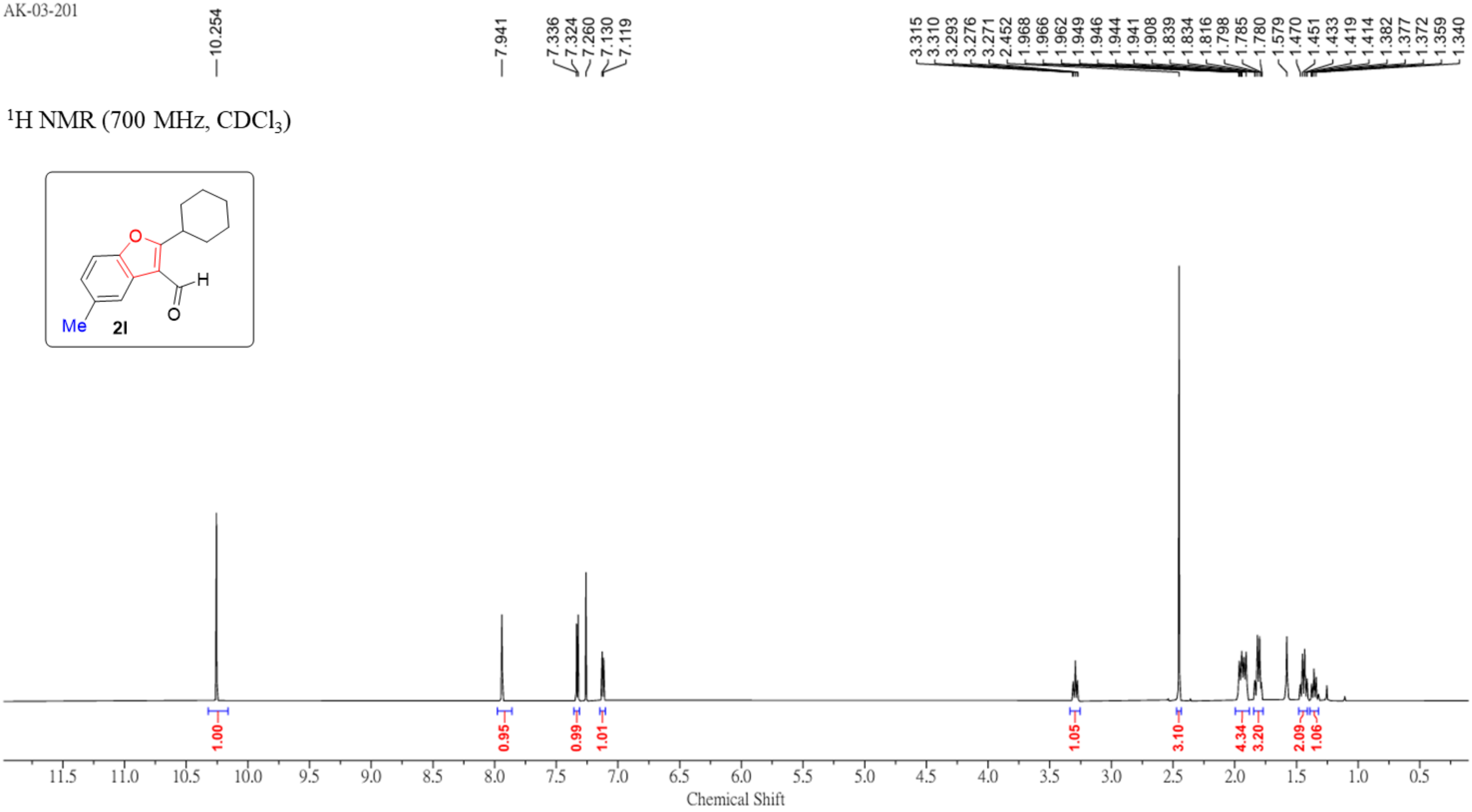
AK-03-201
^{1}H NMR (700 MHz, $CDCl_3$)
10.254
7.941
7.336
7.324
7.260
7.130
7.119
3.315
3.310
3.293
3.276
3.271
2.452
1.968
1.966
1.962
1.949
1.946
1.944
1.941
1.908
1.839
1.834
1.816
1.798
1.785
1.780
1.579
1.470
1.451
1.433
1.419
1.414
1.382
1.377
1.372
1.359
1.340
O
H
O
Me
2l
1.00
0.95
0.99
1.01
1.05
3.10
4.34
3.20
2.09
1.06
11.5
11.0
10.5
10.0
9.5
9.0
8.5
8.0
7.5
7.0
6.5
6.0
5.5
5.0
4.5
4.0
3.5
3.0
2.5
2.0
1.5
1.0
0.5
Chemical Shift

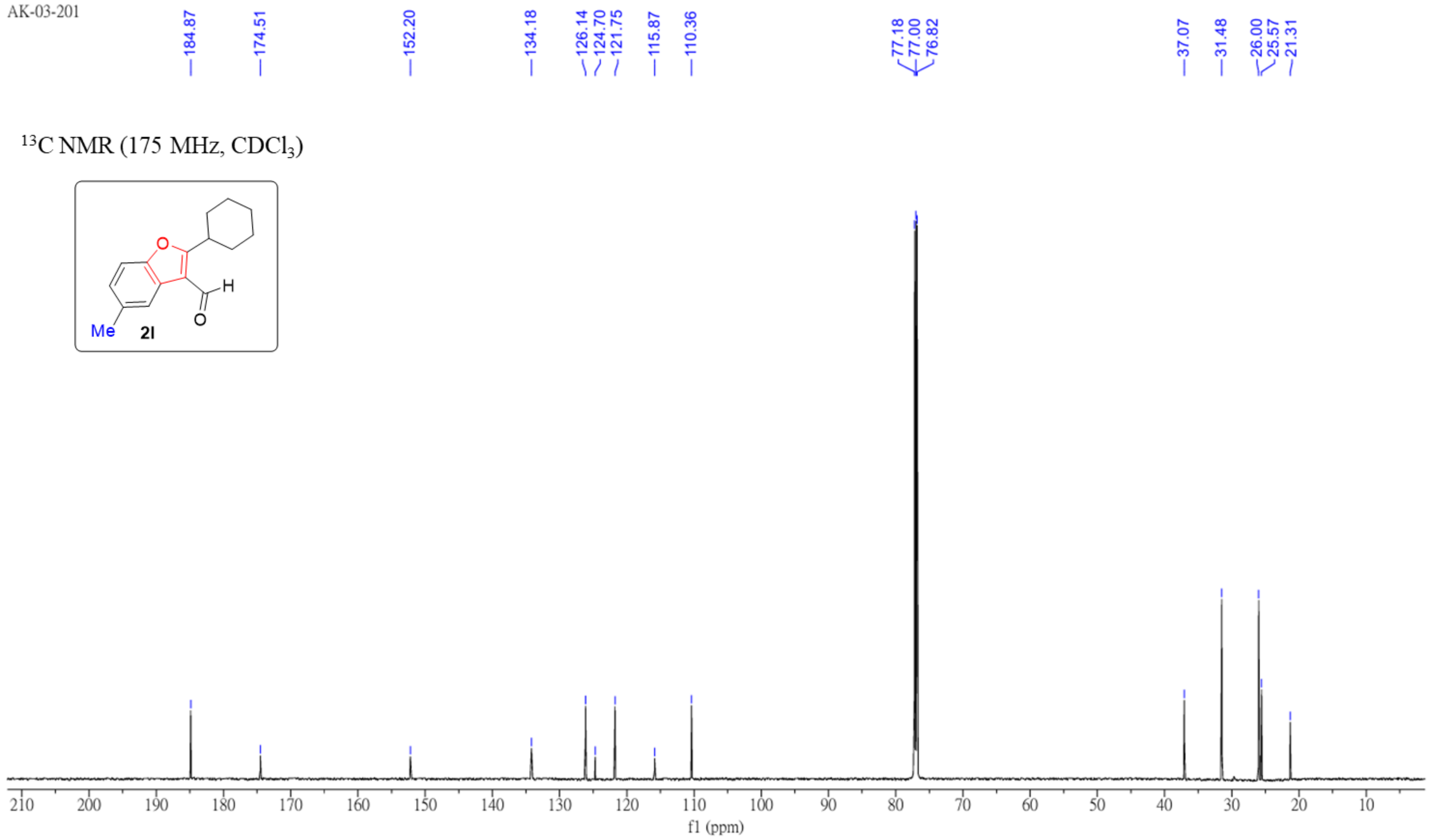

AK-03-201
184.87
174.51
152.20
134.18
126.14
124.70
121.75
115.87
110.36
77.18
77.00
76.82
37.07
31.48
26.00
25.57
21.31
13C NMR (175 MHz, CDCl3)
O
H
O
Me
2l
210 200 190 180 170 160 150 140 130 120 110 100 90 80 70 60 50 40 30 20 10
f1 (ppm)

AK-03-167

^1H NMR (700 MHz, $CDCl_3$)

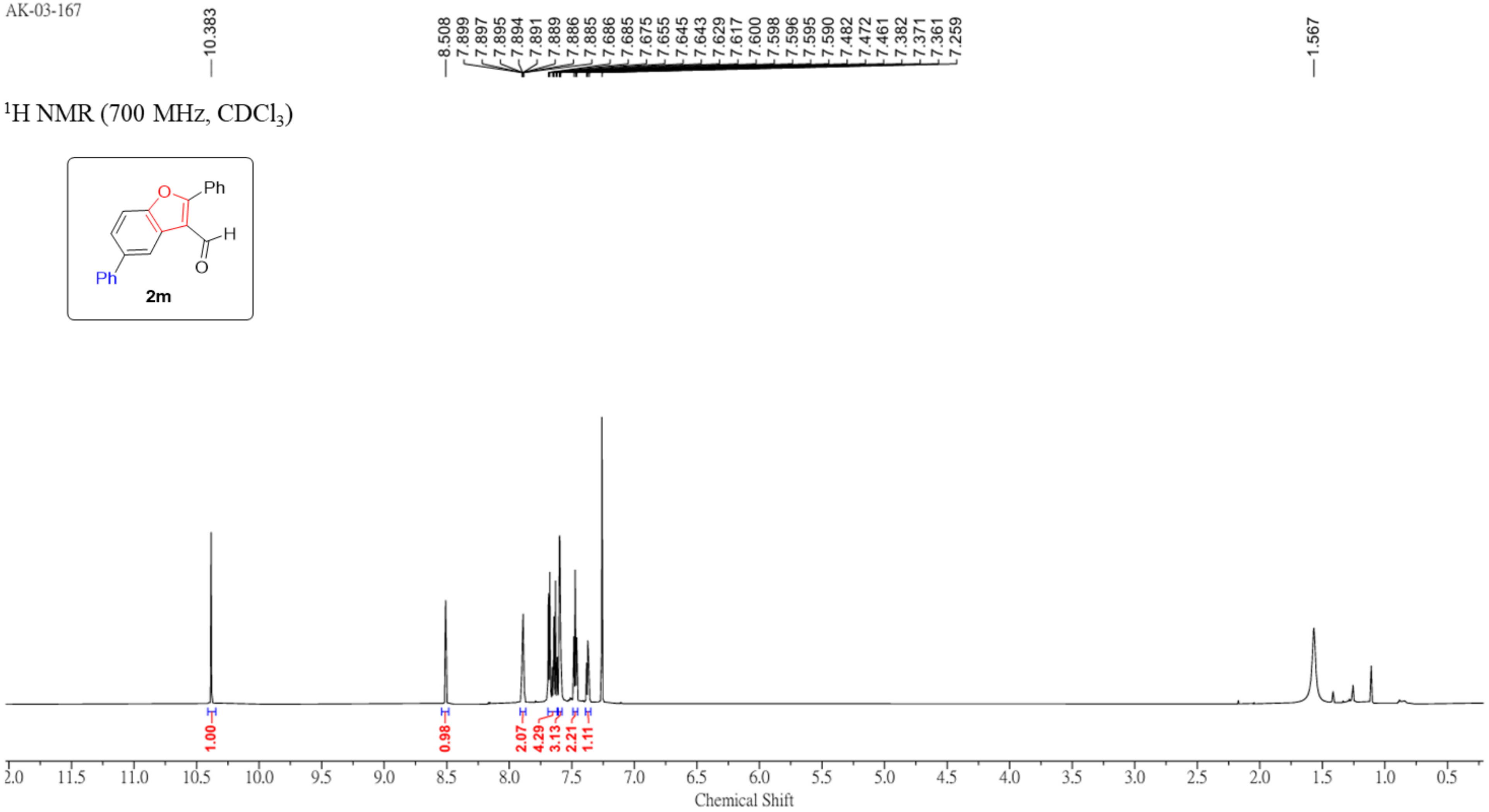

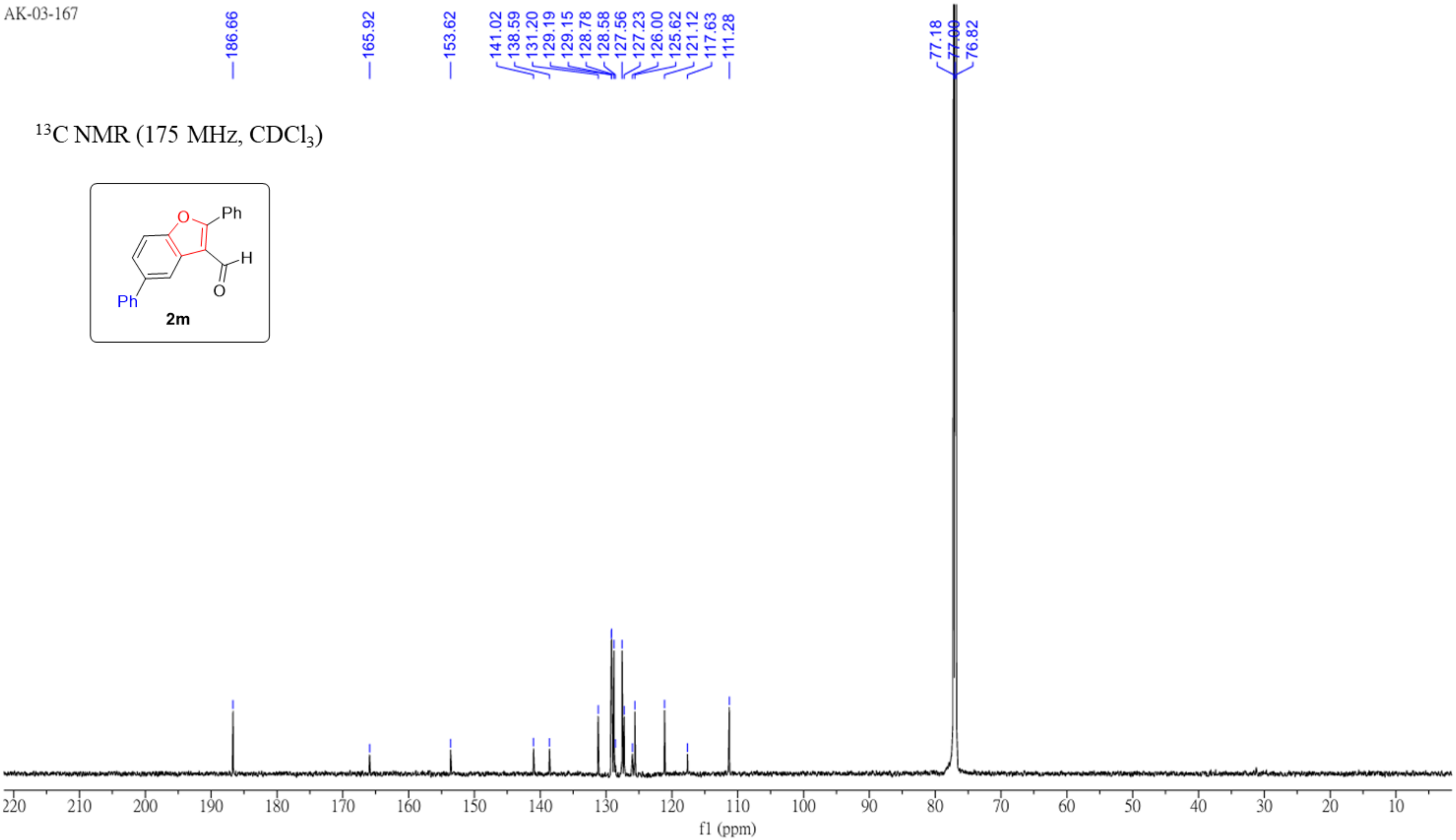
AK-03-167
186.66
165.92
153.62
141.02
138.59
131.20
129.19
129.15
128.78
128.58
127.56
127.23
126.00
125.62
121.12
117.63
111.28
77.18
77.00
76.82
^{13}C NMR (175 MHz, CDCl$_3$)
O
Ph
H
O
Ph
220 210 200 190 180 170 160 150 140 130 120 110 100 90 80 70 60 50 40 30 20 10
f1 (ppm)

2m

AK-03-170

^{1}H NMR (700 MHz, $CDCl_3$)

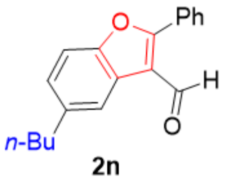

2n

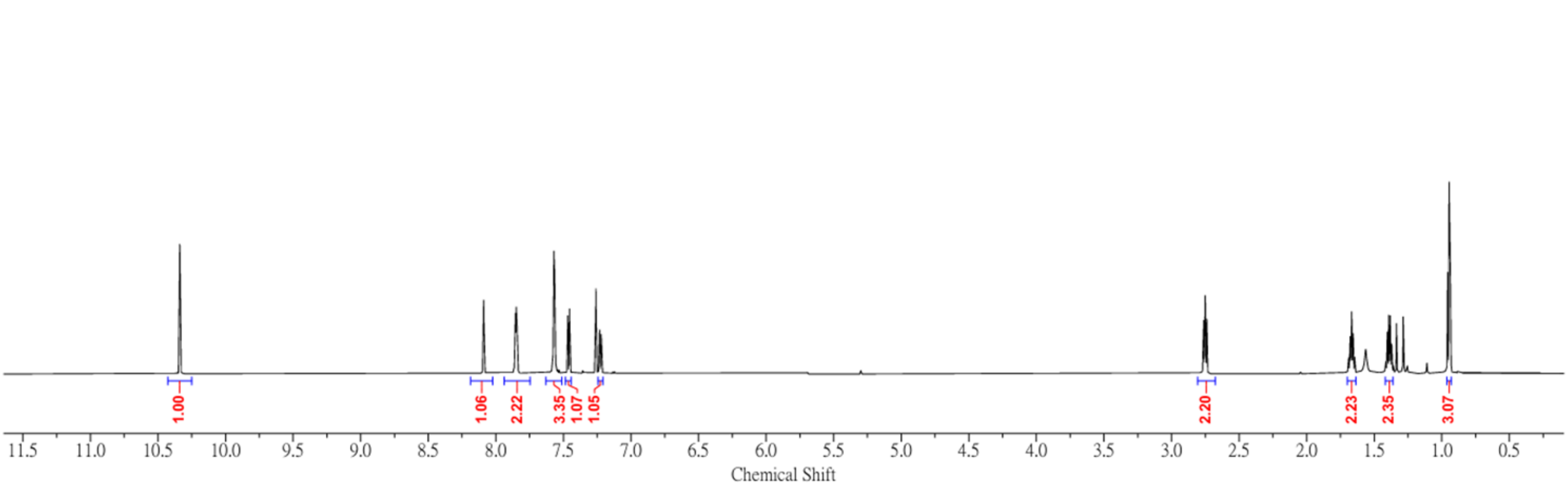

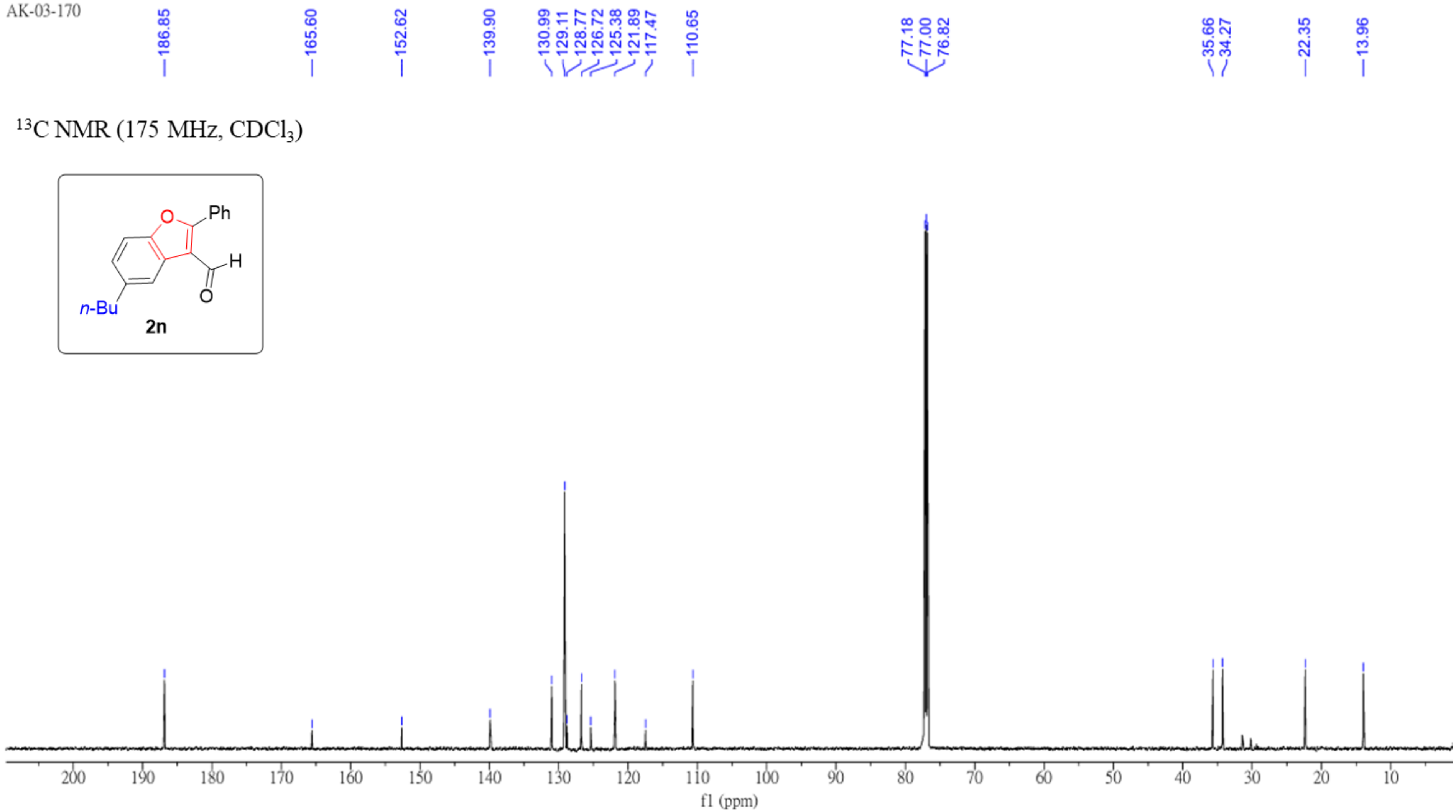
AK-03-170
186.85
165.60
152.62
139.90
130.99
129.11
128.77
126.72
125.38
121.89
117.47
110.65
77.18
77.00
76.82
35.66
34.27
22.35
13.96
^{13}C NMR (175 MHz, $CDCl_3$)
O
Ph
H
O
n-Bu
2n
200
190
180
170
160
150
140
130
120
110
100
90
80
70
60
50
40
30
20
10
f1 (ppm)

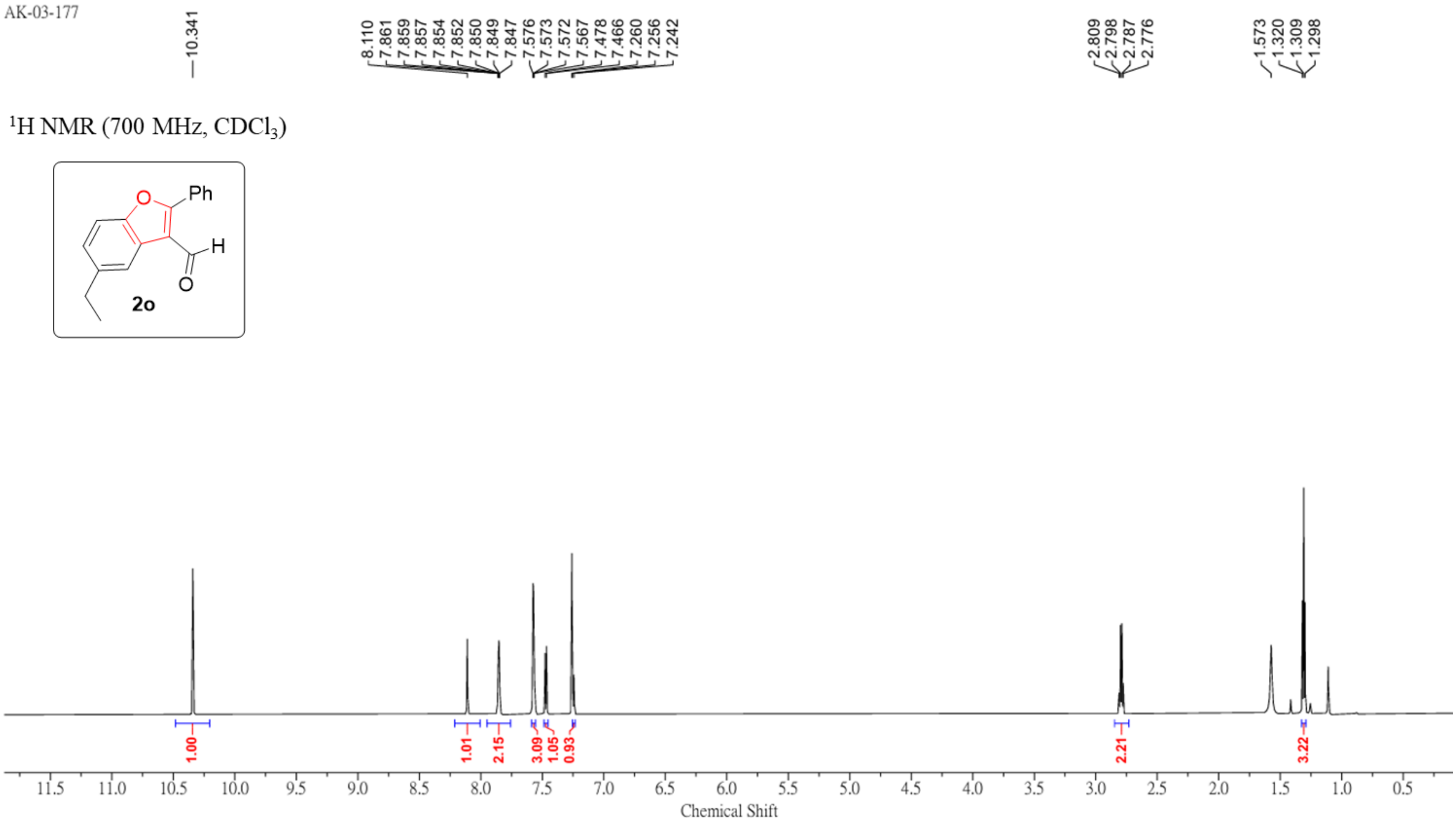
AK-03-177
10.341
8.110
7.861
7.859
7.857
7.854
7.852
7.850
7.849
7.847
7.576
7.573
7.572
7.567
7.478
7.466
7.260
7.256
7.242
2.809
2.798
2.787
2.776
1.573
1.320
1.309
1.298
1H NMR (700 MHz, CDCl3)
O
Ph
H
O
2o
1.00
1.01
2.15
3.09
1.05
0.93
2.21
3.22
11.5
11.0
10.5
10.0
9.5
9.0
8.5
8.0
7.5
7.0
6.5
6.0
5.5
5.0
4.5
4.0
3.5
3.0
2.5
2.0
1.5
1.0
0.5
Chemical Shift

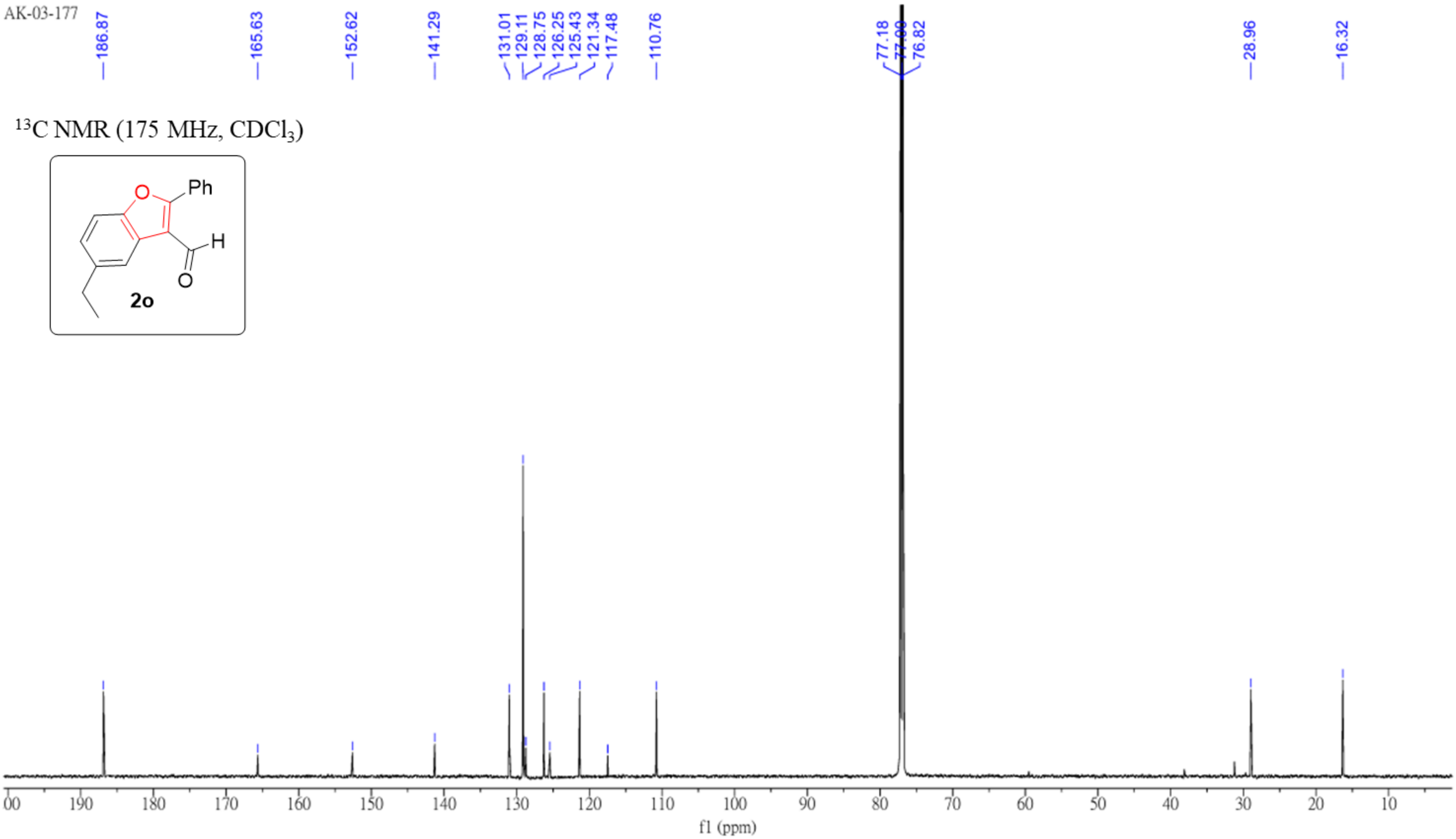

AK-03-177
186.87
165.63
152.62
141.29
131.01
129.11
128.75
126.25
125.43
121.34
117.48
110.76
77.18
77.00
76.82
28.96
16.32
^{13}C NMR (175 MHz, $CDCl_3$)
O
Ph
H
O
2o
f1 (ppm)

AK-03-181

^{1}H NMR (700 MHz, $CDCl_3$)

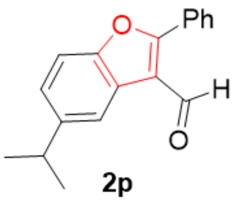

2p

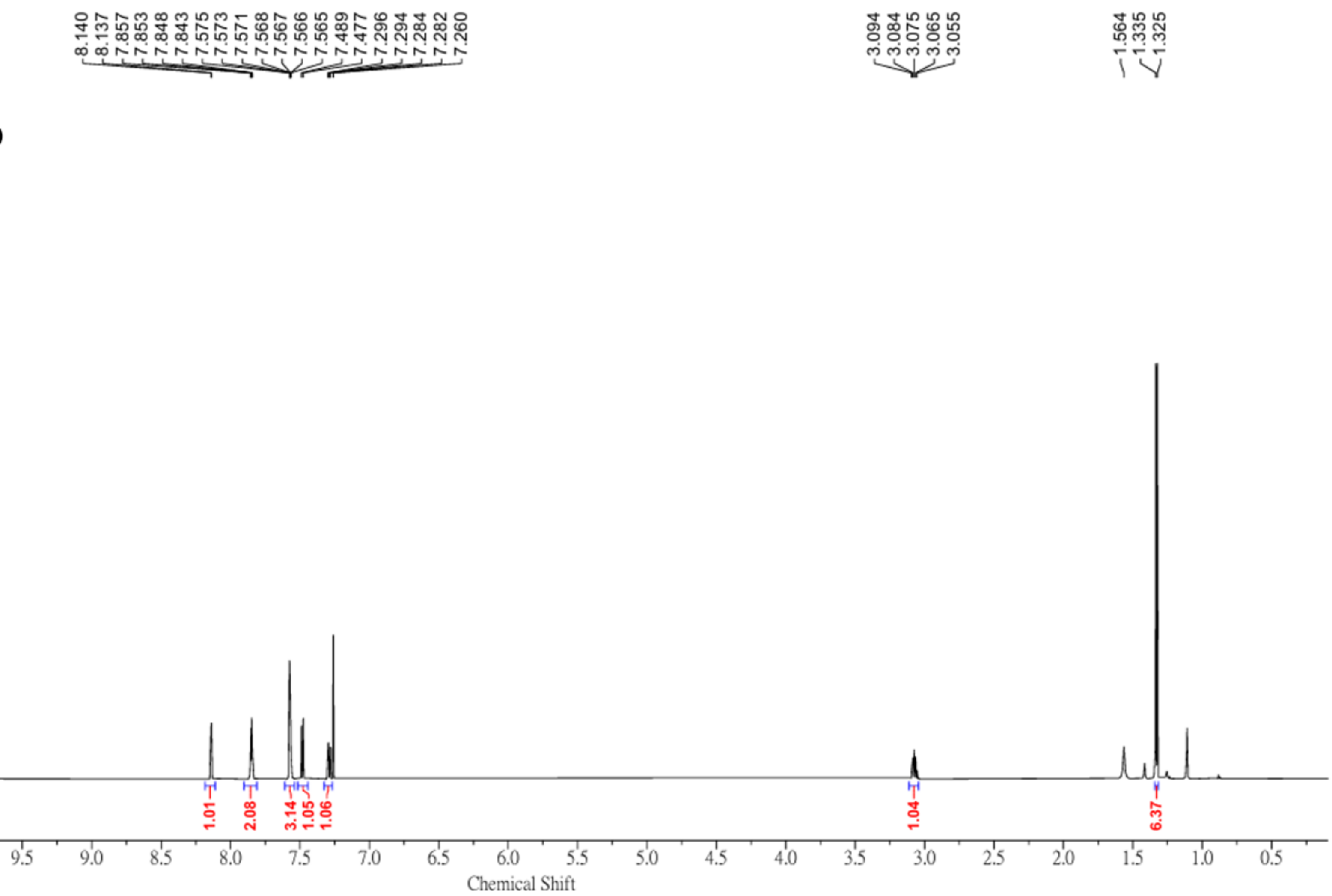

AK-03-181

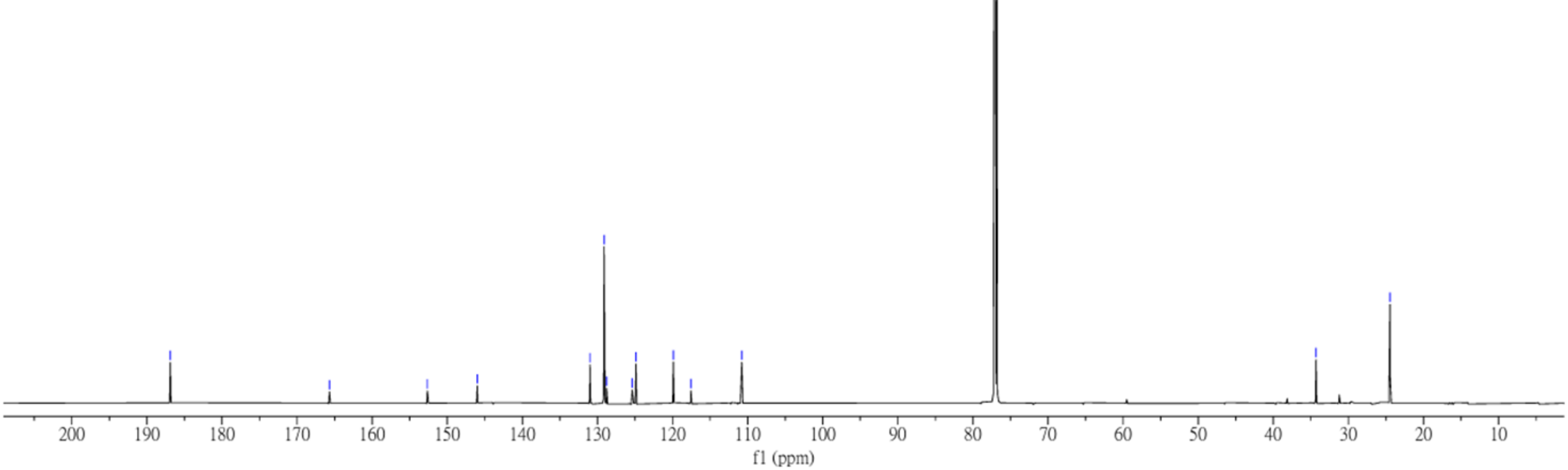

^{13}C NMR (175 MHz, $CDCl_3$)

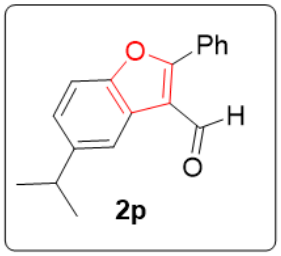

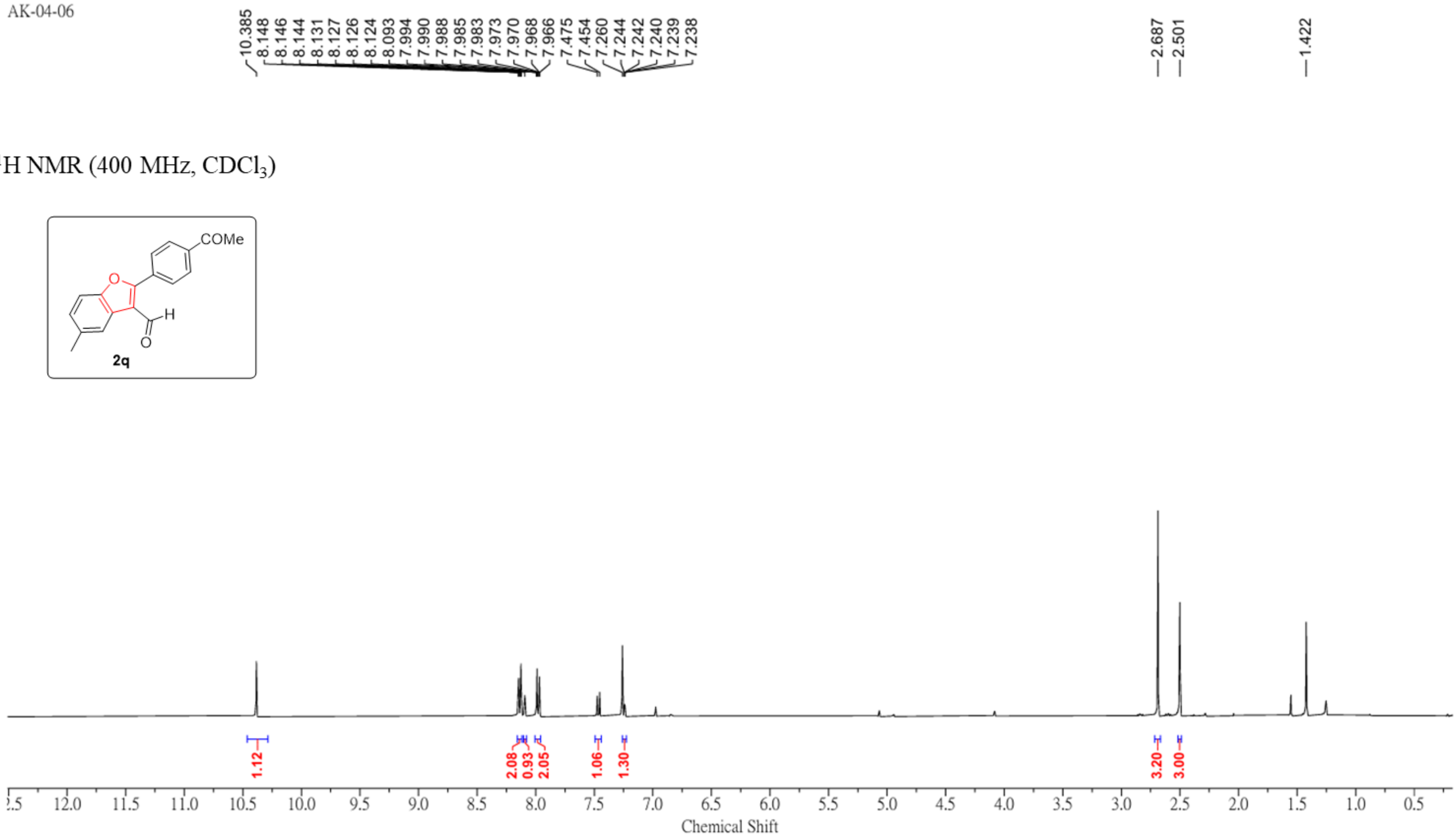
AK-04-06
10.385
8.148
8.146
8.144
8.131
8.127
8.126
8.124
8.093
7.994
7.990
7.988
7.985
7.983
7.973
7.970
7.968
7.966
7.475
7.454
7.260
7.244
7.242
7.240
7.239
7.238
2.687
2.501
1.422
^{1}H NMR (400 MHz, $CDCl_3$)
COMe
O
H
2q
1.12
2.08
0.93
2.05
1.06
1.30
3.20
3.00
12.0
11.5
11.0
10.5
10.0
9.5
9.0
8.5
8.0
7.5
7.0
6.5
6.0
5.5
5.0
4.5
4.0
3.5
3.0
2.5
2.0
1.5
1.0
0.5
Chemical Shift

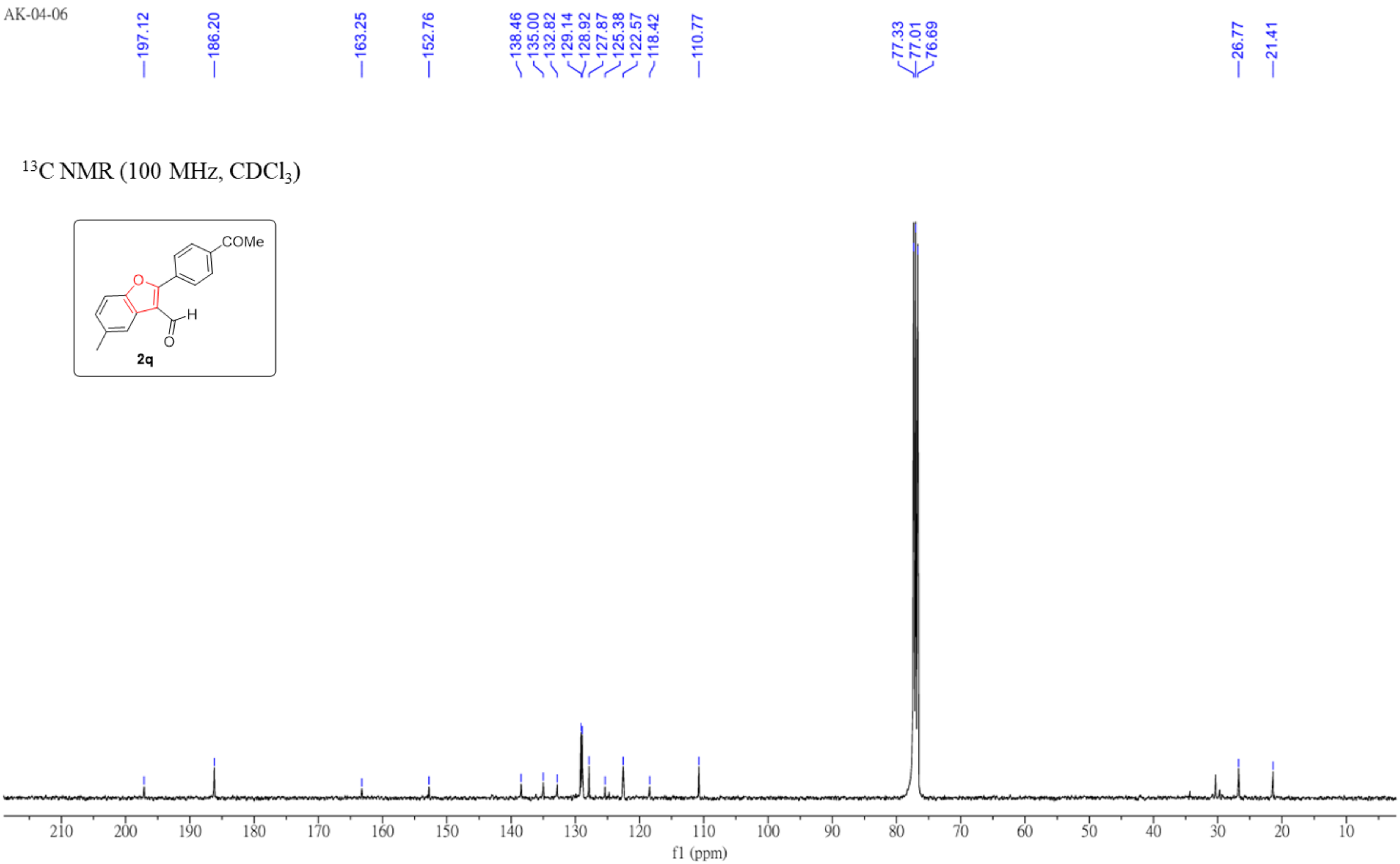
AK-04-06
197.12
186.20
163.25
152.76
138.46
135.00
132.82
129.14
128.92
127.87
125.38
122.57
118.42
110.77
77.33
77.01
76.69
26.77
21.41
^{13}C NMR (100 MHz, CDCl$_3$)
COMe
O
H
O
2q
210
200
190
180
170
160
150
140
130
120
110
100
90
80
70
60
50
40
30
20
10
f1 (ppm)

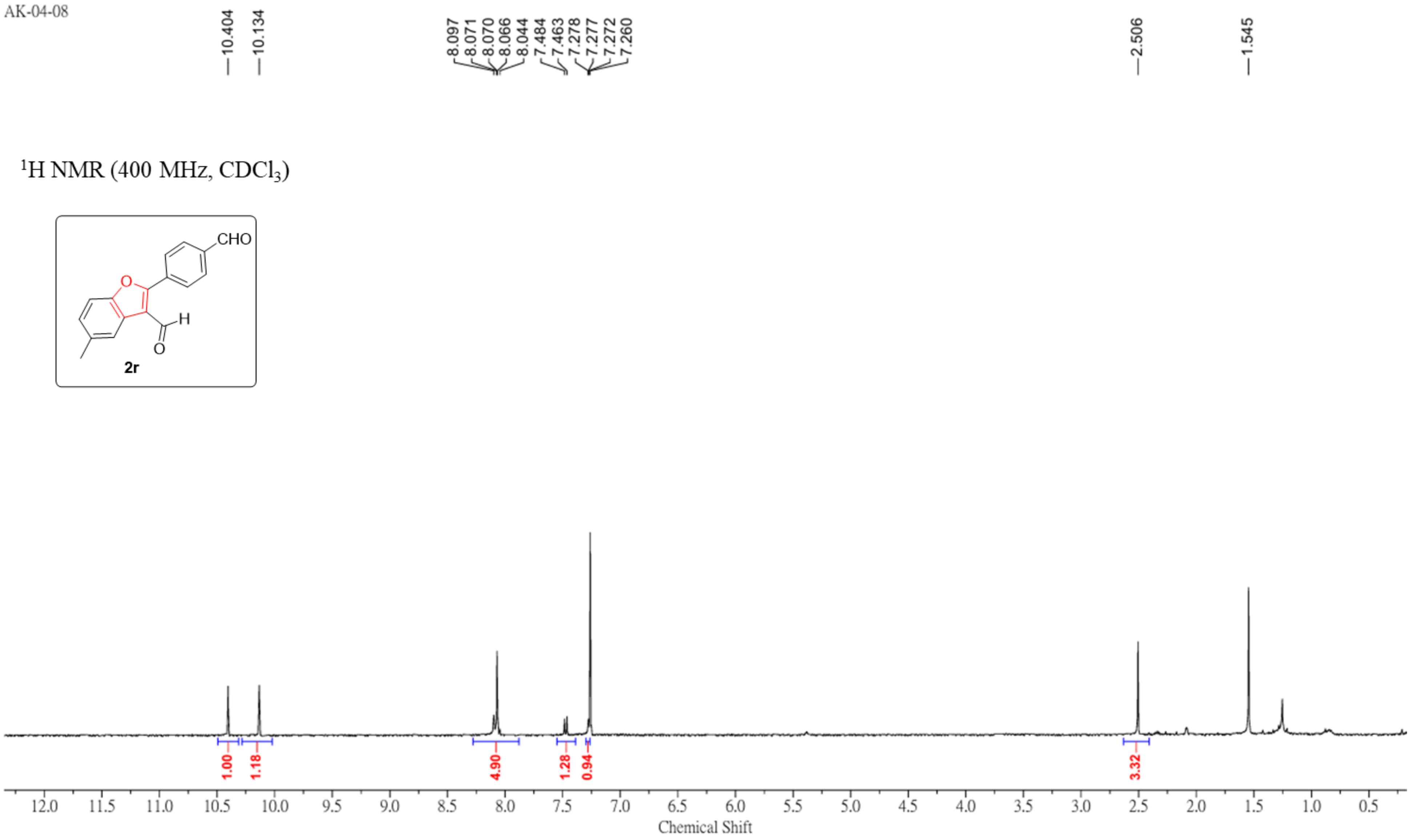

AK-04-08
10.404
10.134
8.097
8.071
8.070
8.066
8.044
7.484
7.463
7.278
7.277
7.272
7.260
2.506
1.545
^{1}H NMR (400 MHz, CDCl$_3$)
CHO
O
H
O
2r
1.00
1.18
4.90
1.28
0.94
3.32
12.0
11.5
11.0
10.5
10.0
9.5
9.0
8.5
8.0
7.5
7.0
6.5
6.0
5.5
5.0
4.5
4.0
3.5
3.0
2.5
2.0
1.5
1.0
0.5
Chemical Shift

AK-04-08

^{13}C NMR (175 MHz, $CDCl_3$)

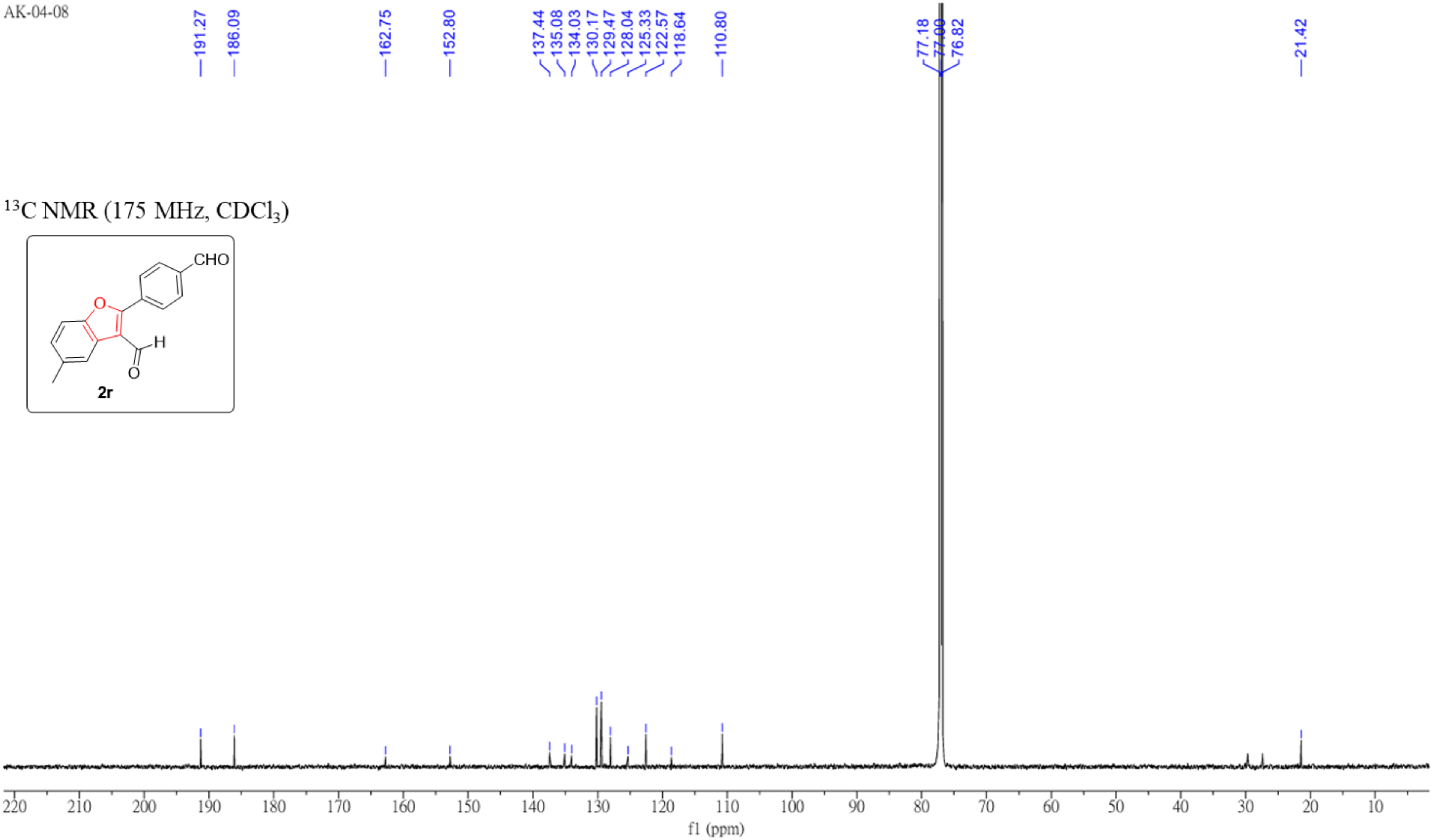

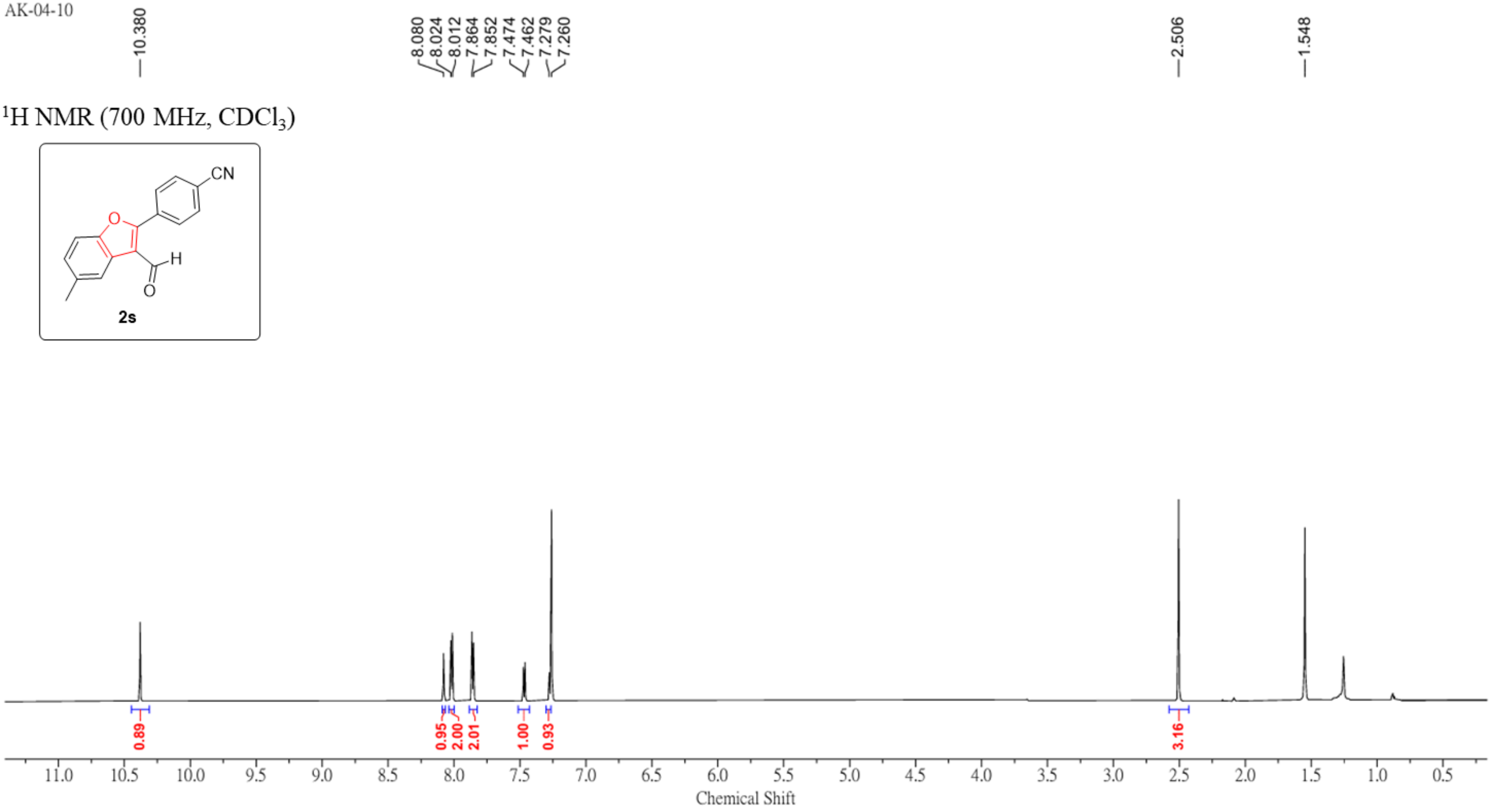
AK-04-10
^{1}H NMR (700 MHz, CDCl$_3$)
CN
O
H
2s
10.380
8.080
8.024
8.012
7.864
7.852
7.474
7.462
7.279
7.260
2.506
1.548
0.89
0.95
2.00
2.01
1.00
0.93
3.16
11.0 10.5 10.0 9.5 9.0 8.5 8.0 7.5 7.0 6.5 6.0 5.5 5.0 4.5 4.0 3.5 3.0 2.5 2.0 1.5 1.0 0.5
Chemical Shift

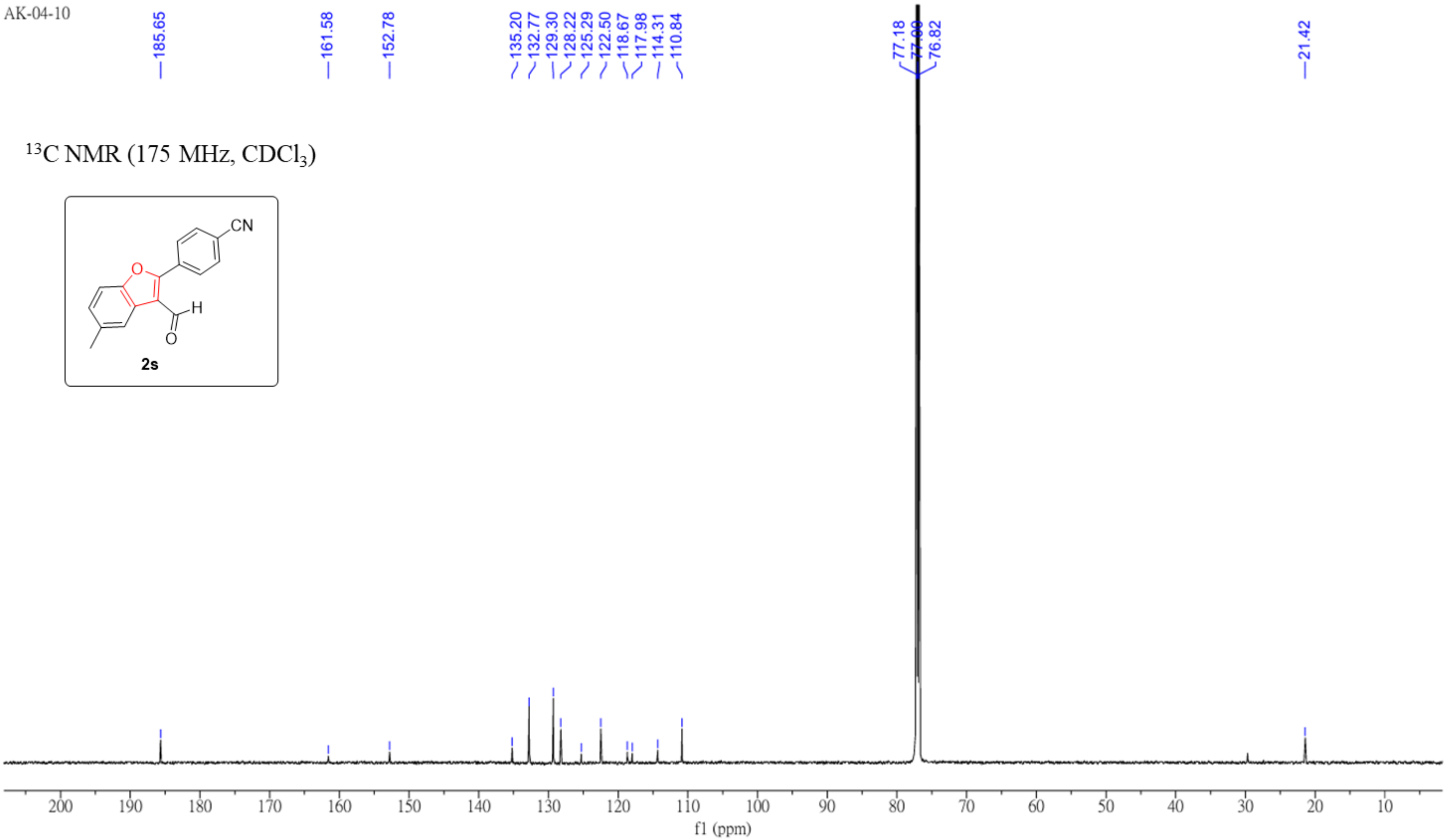
AK-04-10
185.65
161.58
152.78
135.20
132.77
129.30
128.22
125.29
122.50
118.67
117.98
114.31
110.84
77.18
76.82
21.42
13C NMR (175 MHz, CDCl3)
CN
O
H
O
2s
200
190
180
170
160
150
140
130
120
110
100
90
80
70
60
50
40
30
20
10
f1 (ppm)

AK-03-175A

^{1}H NMR (700 MHz, $CDCl_3$)

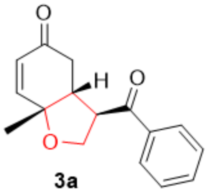

3a

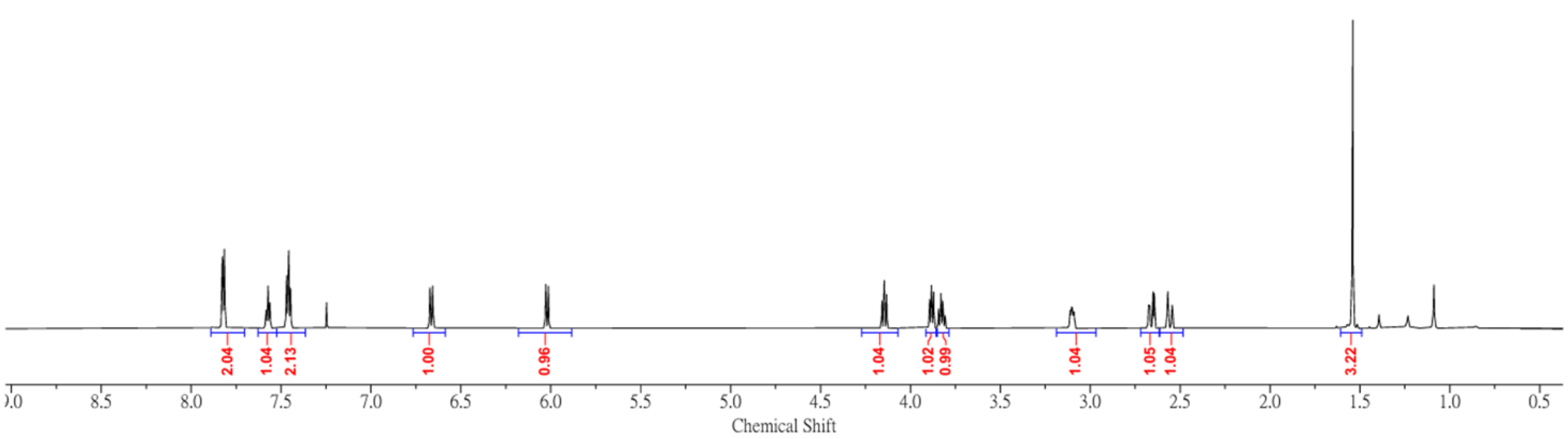

AK-03-175A

^{13}C NMR (175 MHz, $CDCl_3$)

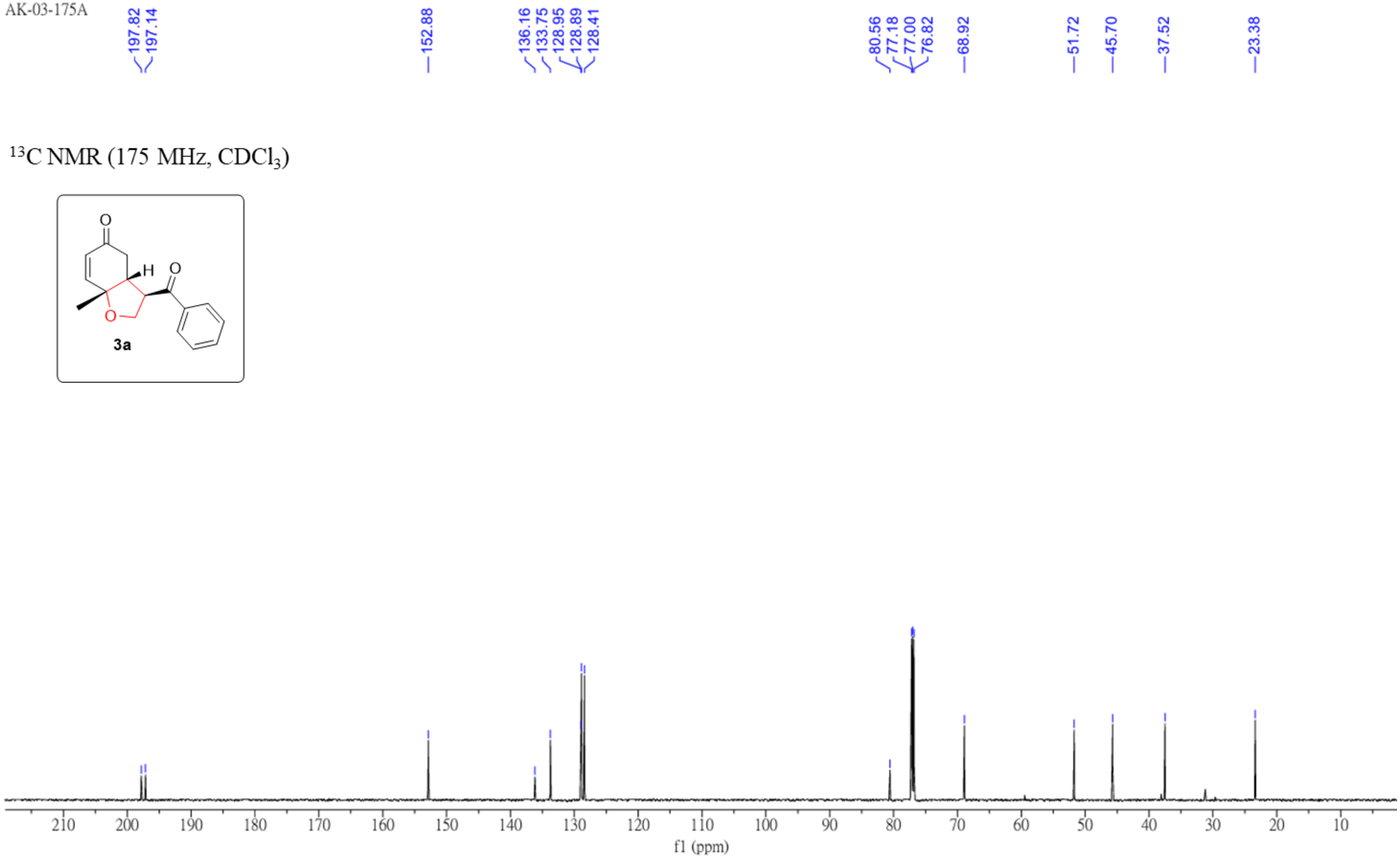

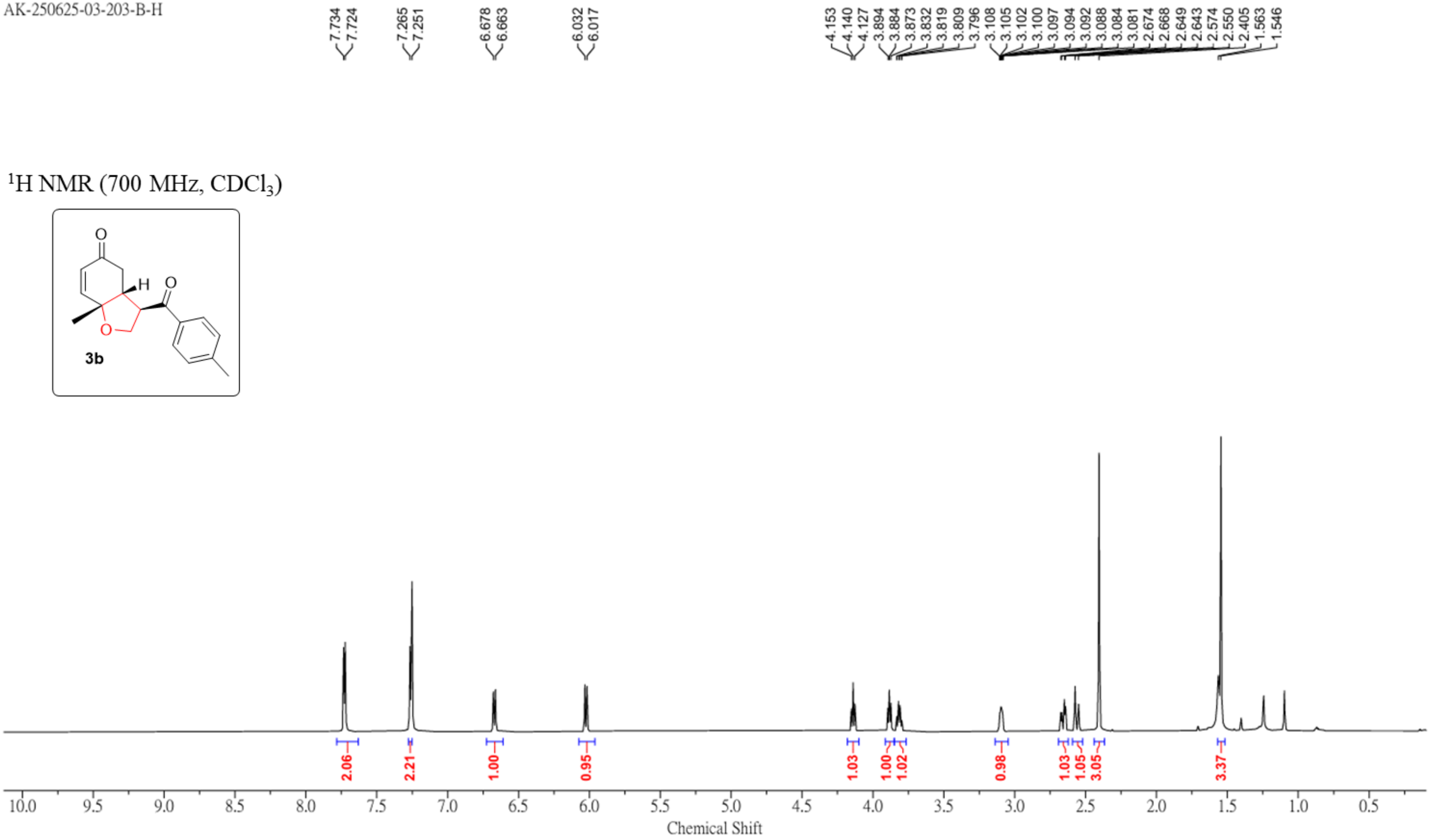
AK-250625-03-203-B-H
7.734
7.724
7.265
7.251
6.678
6.663
6.032
6.017
4.153
4.140
4.127
3.894
3.884
3.873
3.832
3.819
3.809
3.796
3.108
3.105
3.102
3.100
3.097
3.094
3.092
3.088
3.084
3.081
2.674
2.668
2.649
2.643
2.574
2.550
2.405
1.563
1.546
^{1}H NMR (700 MHz, $CDCl_3$)
O
H
O
O
3b
2.06
2.21
1.00
0.95
1.03
1.00
1.02
0.98
1.03
1.05
3.05
3.37
10.0
9.5
9.0
8.5
8.0
7.5
7.0
6.5
6.0
5.5
5.0
4.5
4.0
3.5
3.0
2.5
2.0
1.5
1.0
0.5
Chemical Shift

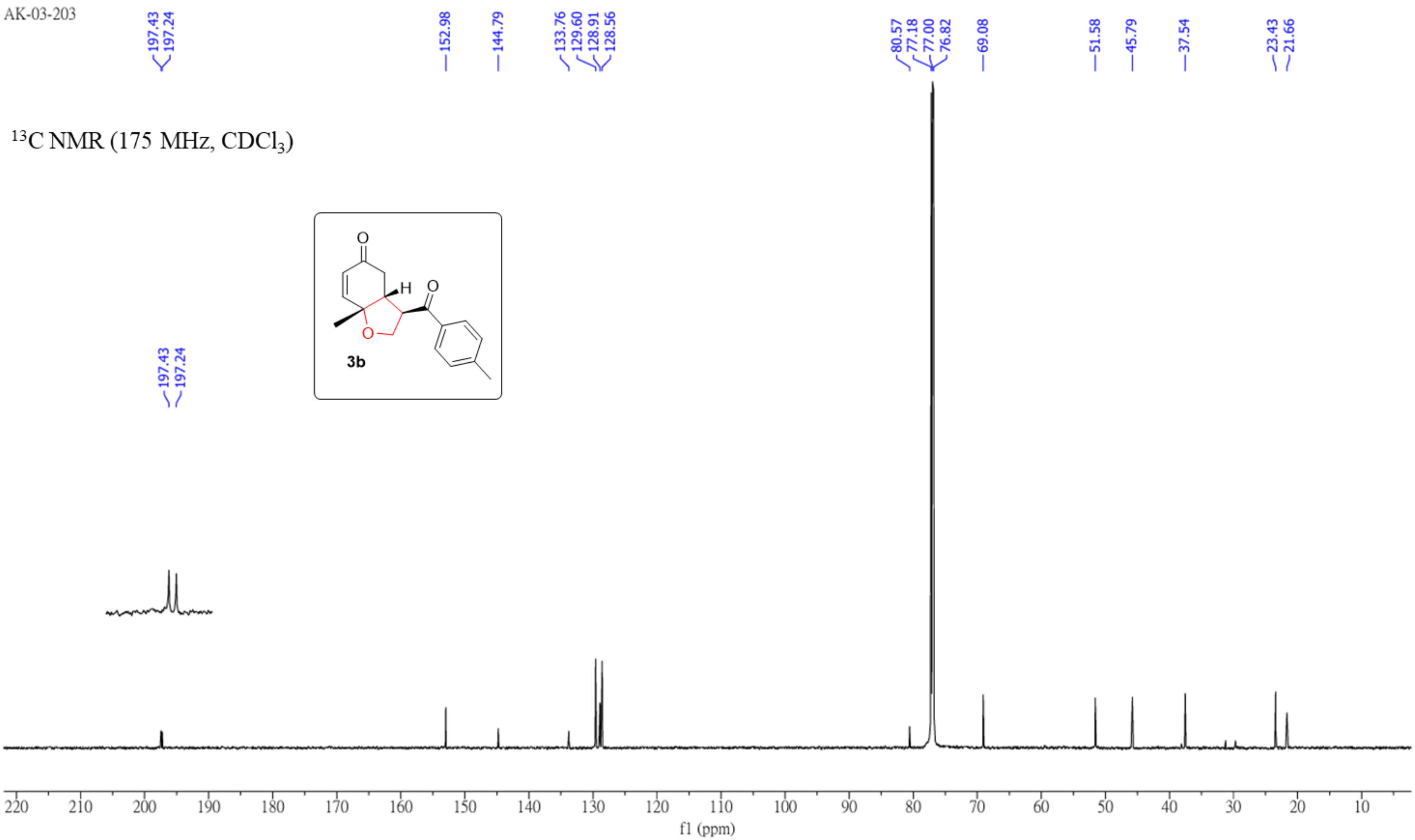
AK-03-203
197.43
197.24
152.98
144.79
133.76
129.60
128.91
128.56
80.57
77.18
77.00
76.82
69.08
51.58
45.79
37.54
23.43
21.66
13C NMR (175 MHz, CDCl3)
O
H
O
O
3b
197.43
197.24
220
210
200
190
180
170
160
150
140
130
120
110
100
90
80
70
60
50
40
30
20
10
f1 (ppm)

AK-03-205

^{1}H NMR (700 MHz, $CDCl_3$)

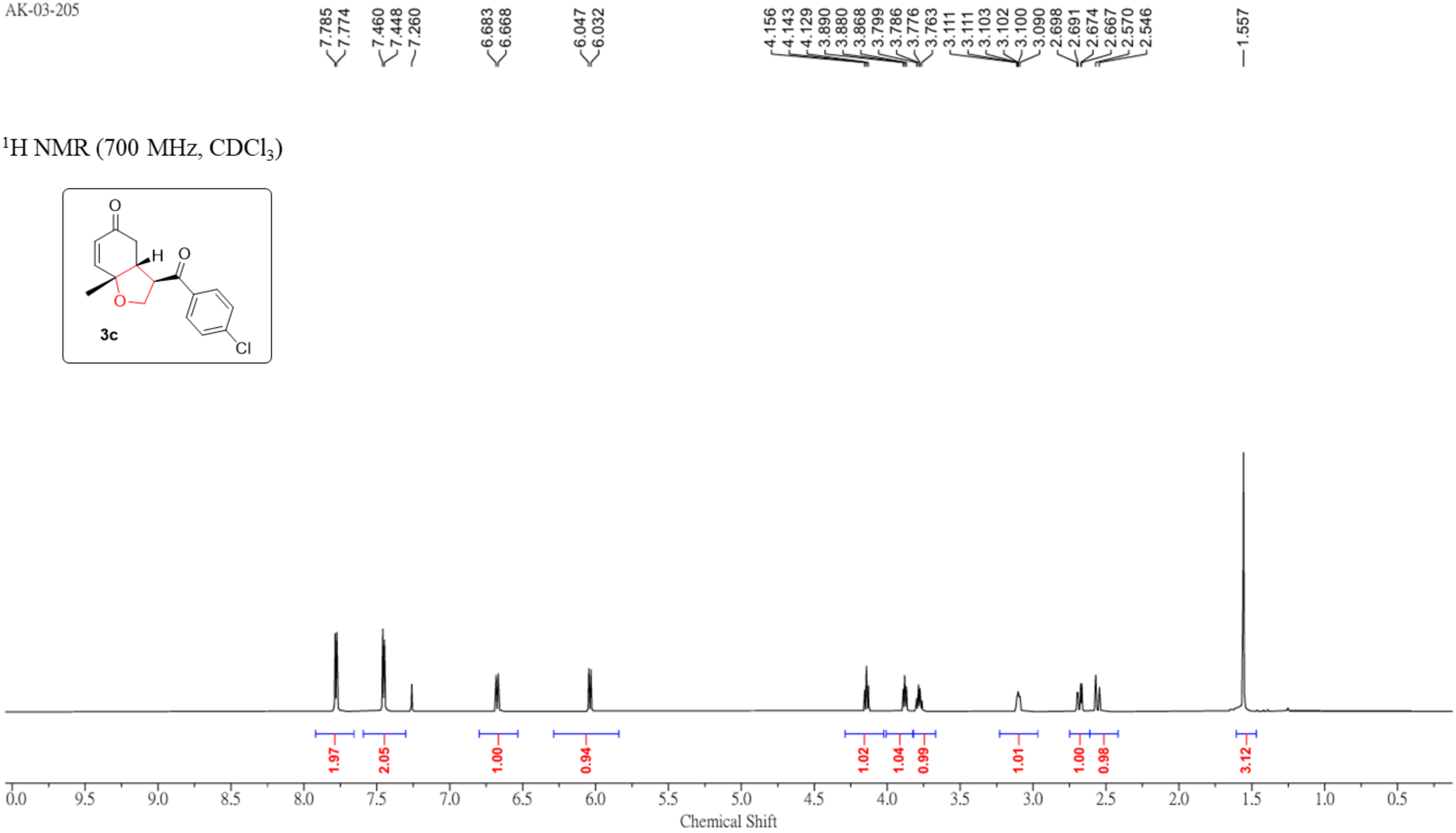

^{13}C NMR (175 MHz, $CDCl_3$)

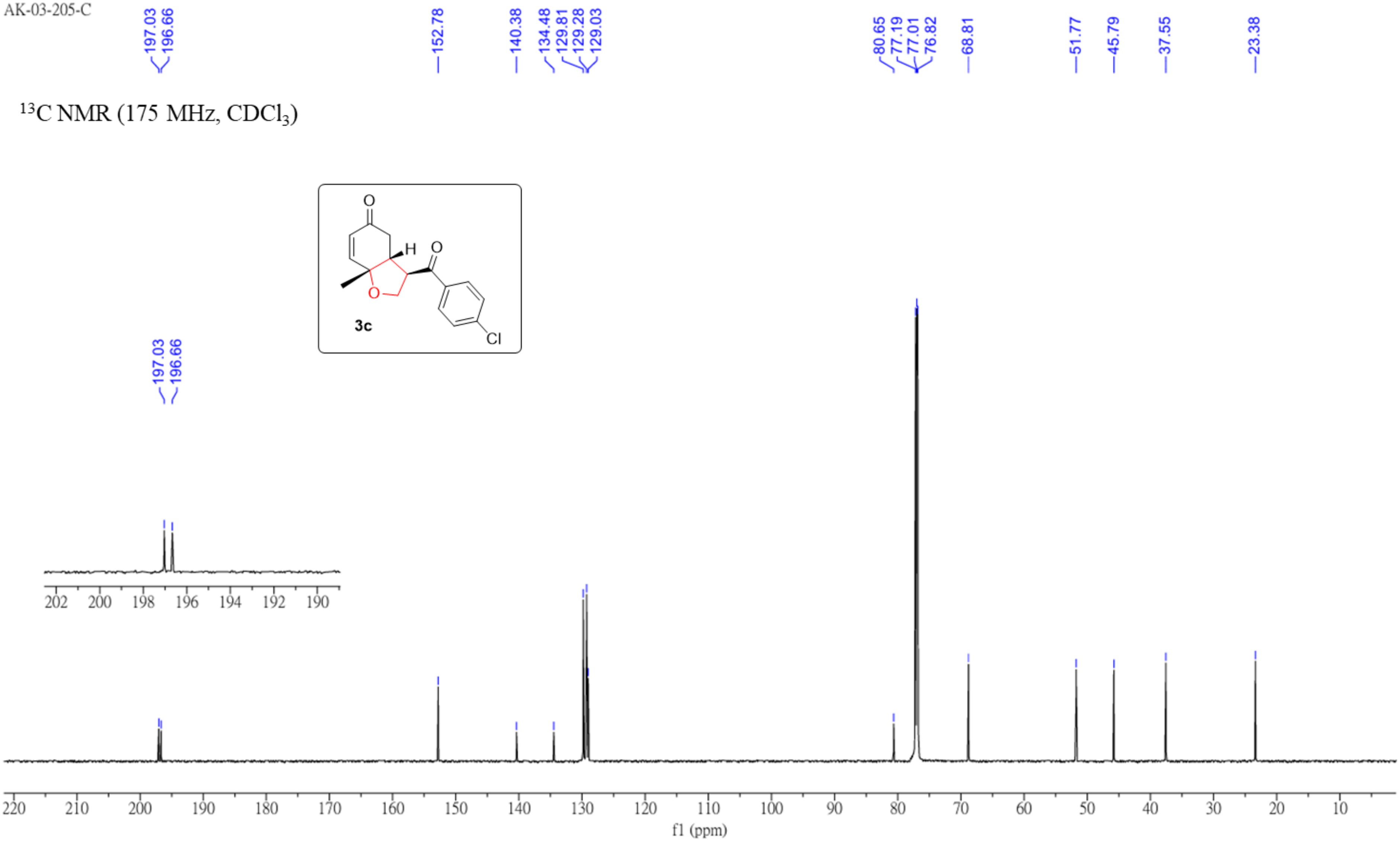

AK-03-206-H

^1H NMR (700 MHz, $CDCl_3$)

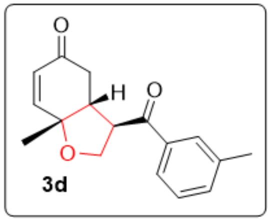

3d

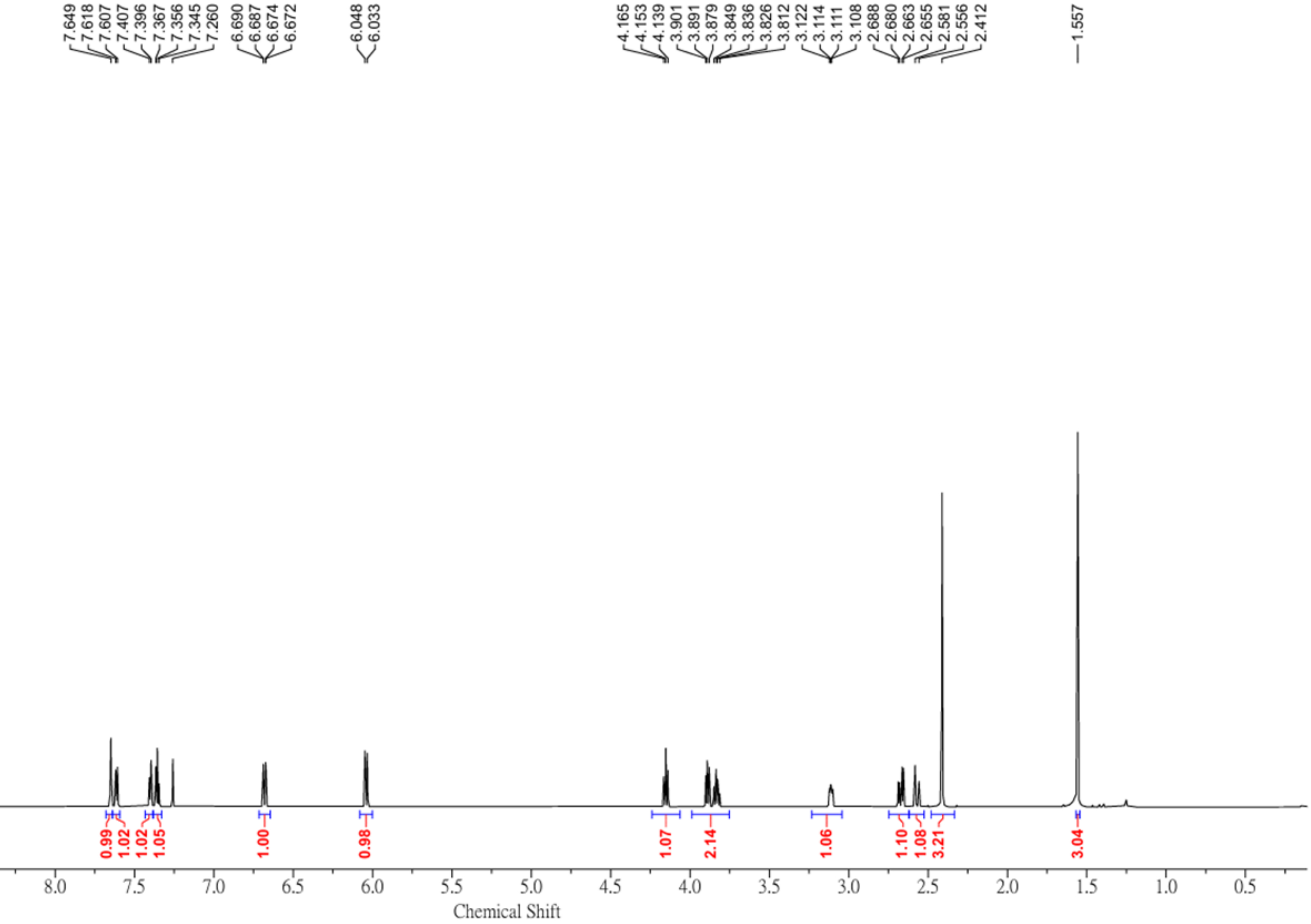

^{13}C NMR (175 MHz, $CDCl_3$)

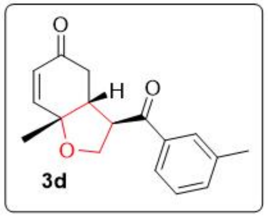

3d

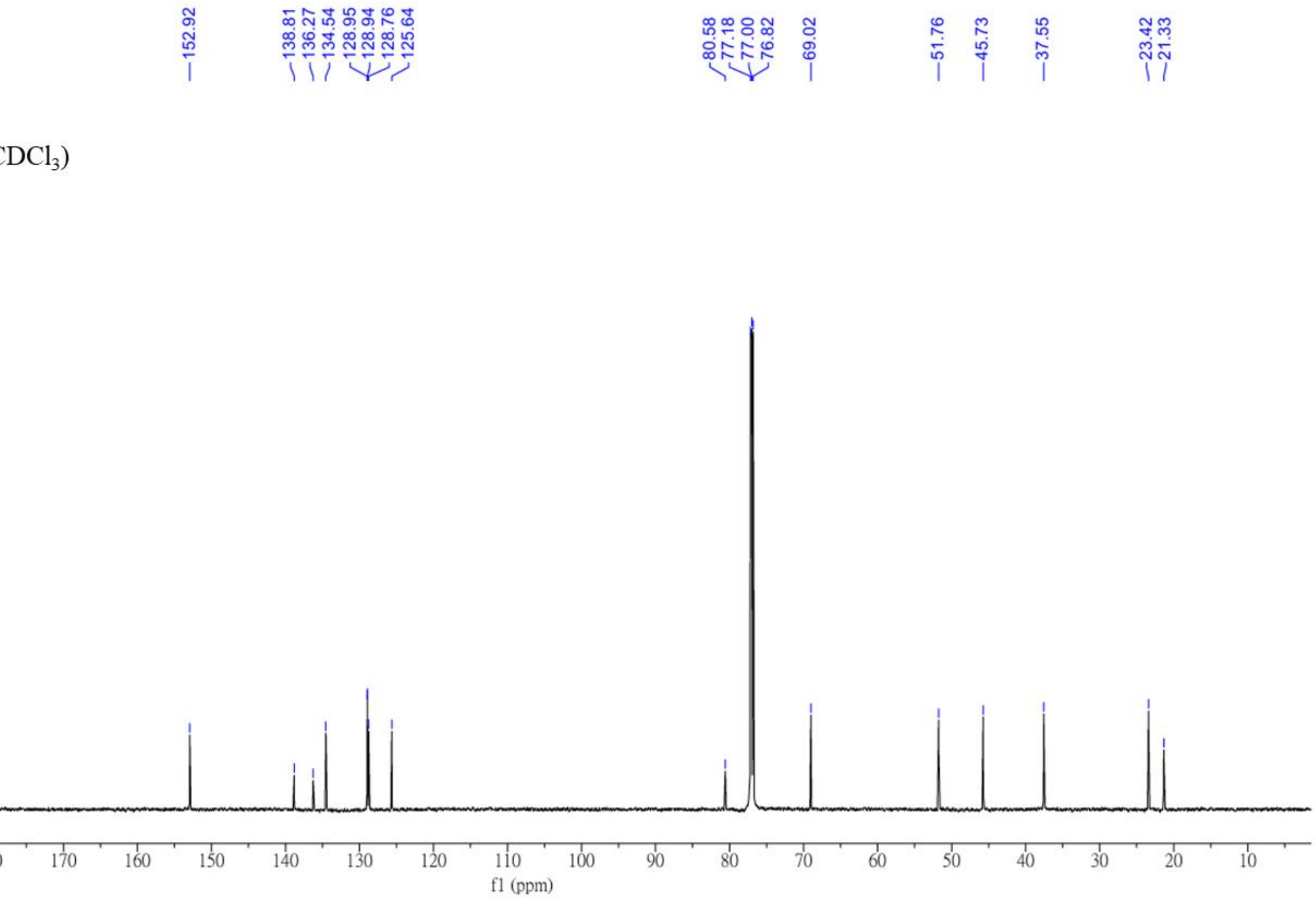

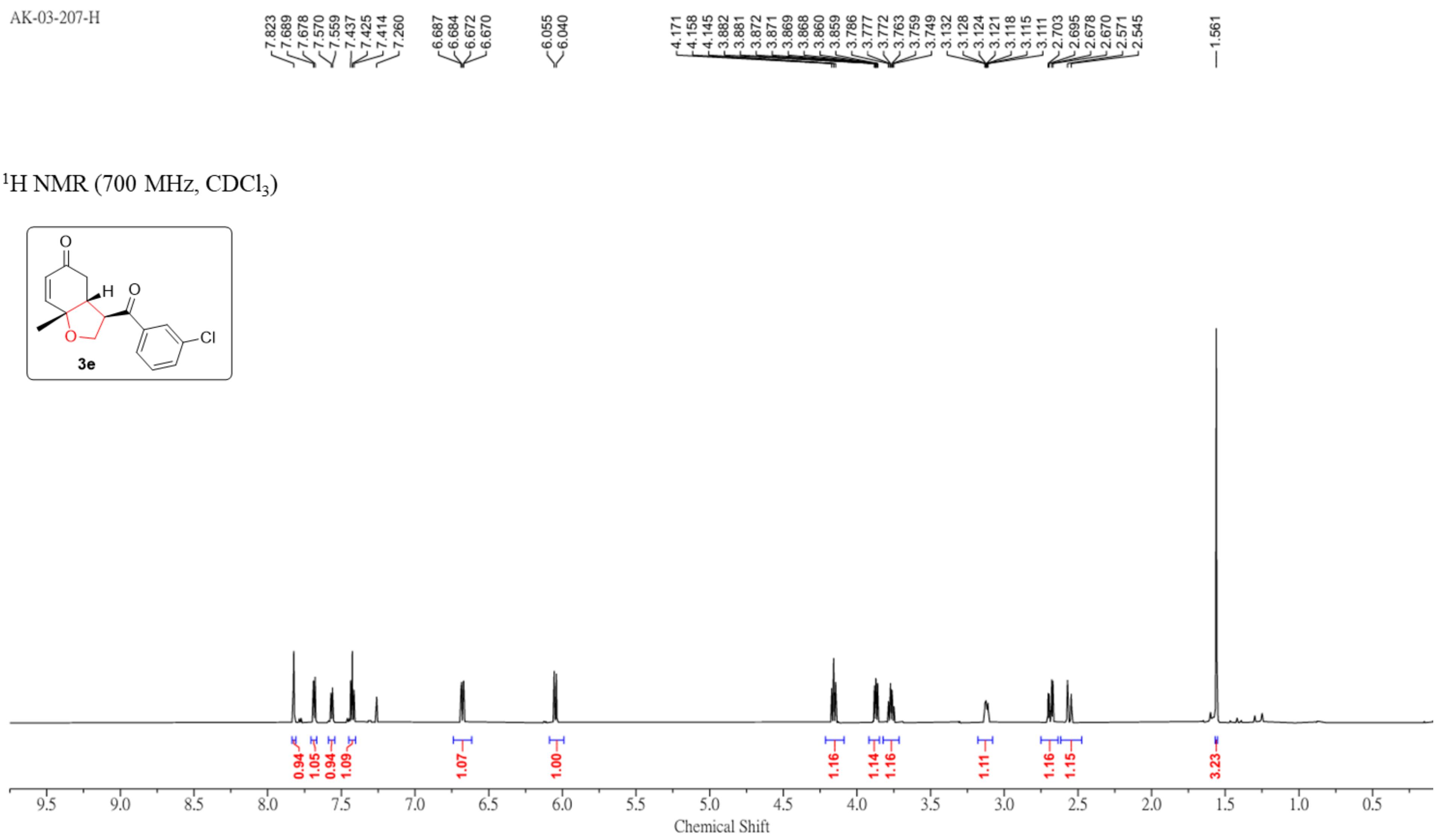

AK-03-207-H
7.823
7.689
7.678
7.570
7.559
7.437
7.425
7.414
7.260
6.687
6.684
6.672
6.670
6.055
6.040
4.171
4.158
4.145
3.882
3.881
3.872
3.871
3.869
3.868
3.860
3.859
3.786
3.777
3.772
3.763
3.759
3.749
3.132
3.128
3.124
3.121
3.118
3.115
3.111
2.703
2.695
2.678
2.670
2.571
2.545
1.561
¹H NMR (700 MHz, CDCl3)
O
H
O
O
Cl
3e
0.94
1.05
0.94
1.09
1.07
1.00
1.16
1.14
1.16
1.11
1.16
1.15
3.23
9.5
9.0
8.5
8.0
7.5
7.0
6.5
6.0
5.5
5.0
4.5
4.0
3.5
3.0
2.5
2.0
1.5
1.0
0.5
Chemical Shift

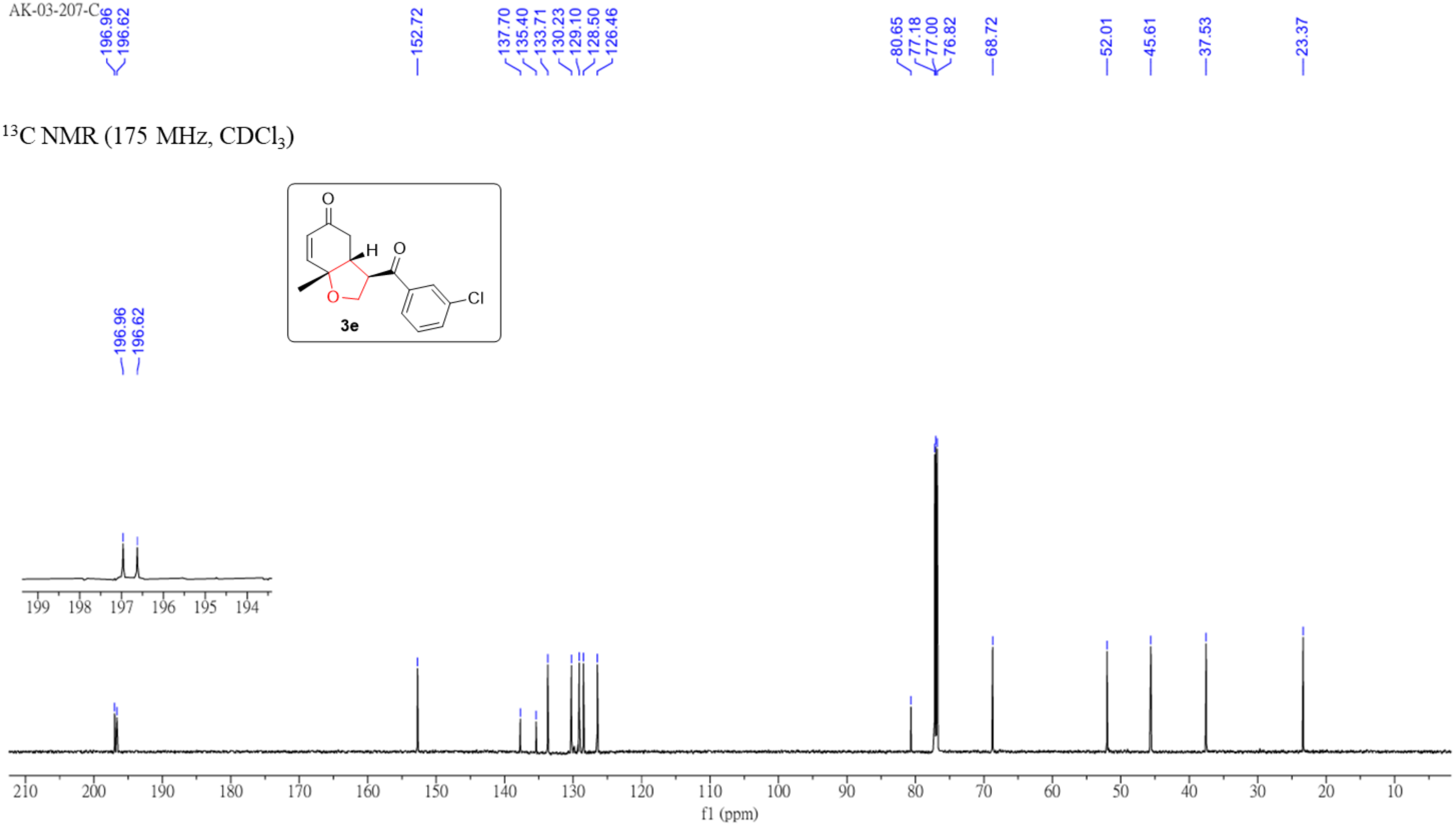
AK-03-207-C
196.96
196.62
152.72
137.70
135.40
133.71
130.23
129.10
128.50
126.46
80.65
77.18
77.00
76.82
68.72
52.01
45.61
37.53
23.37
13C NMR (175 MHz, CDCl3)
O
H
O
Cl
O
3e
196.96
196.62
199 198 197 196 195 194
210 200 190 180 170 160 150 140 130 120 110 100 90 80 70 60 50 40 30 20 10
f1 (ppm)

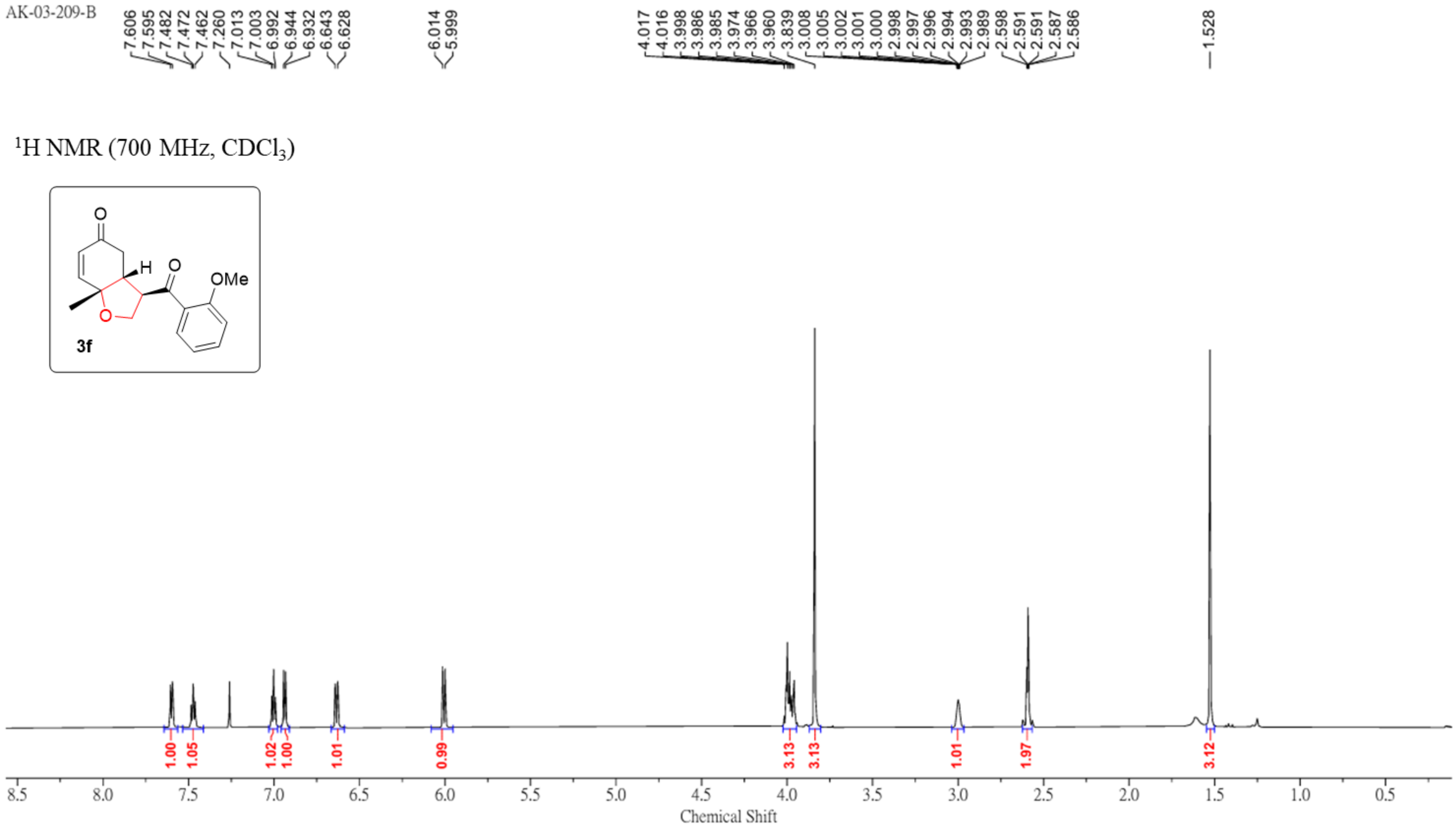
AK-03-209-B
7.606
7.595
7.482
7.472
7.462
7.260
7.013
7.003
6.992
6.944
6.932
6.643
6.628
6.014
5.999
4.017
4.016
3.998
3.986
3.985
3.974
3.966
3.960
3.839
3.008
3.005
3.002
3.001
3.000
2.998
2.997
2.996
2.994
2.993
2.989
2.598
2.591
2.591
2.587
2.586
1.528
¹H NMR (700 MHz, CDCl3)
OMe
3f
1.00
1.05
1.02
1.00
1.01
0.99
3.13
3.13
1.01
1.97
3.12
8.5
8.0
7.5
7.0
6.5
6.0
5.5
5.0
4.5
4.0
3.5
3.0
2.5
2.0
1.5
1.0
0.5
Chemical Shift

AK-250703-03-209-B-C

^{13}C NMR (175 MHz, $CDCl_3$)

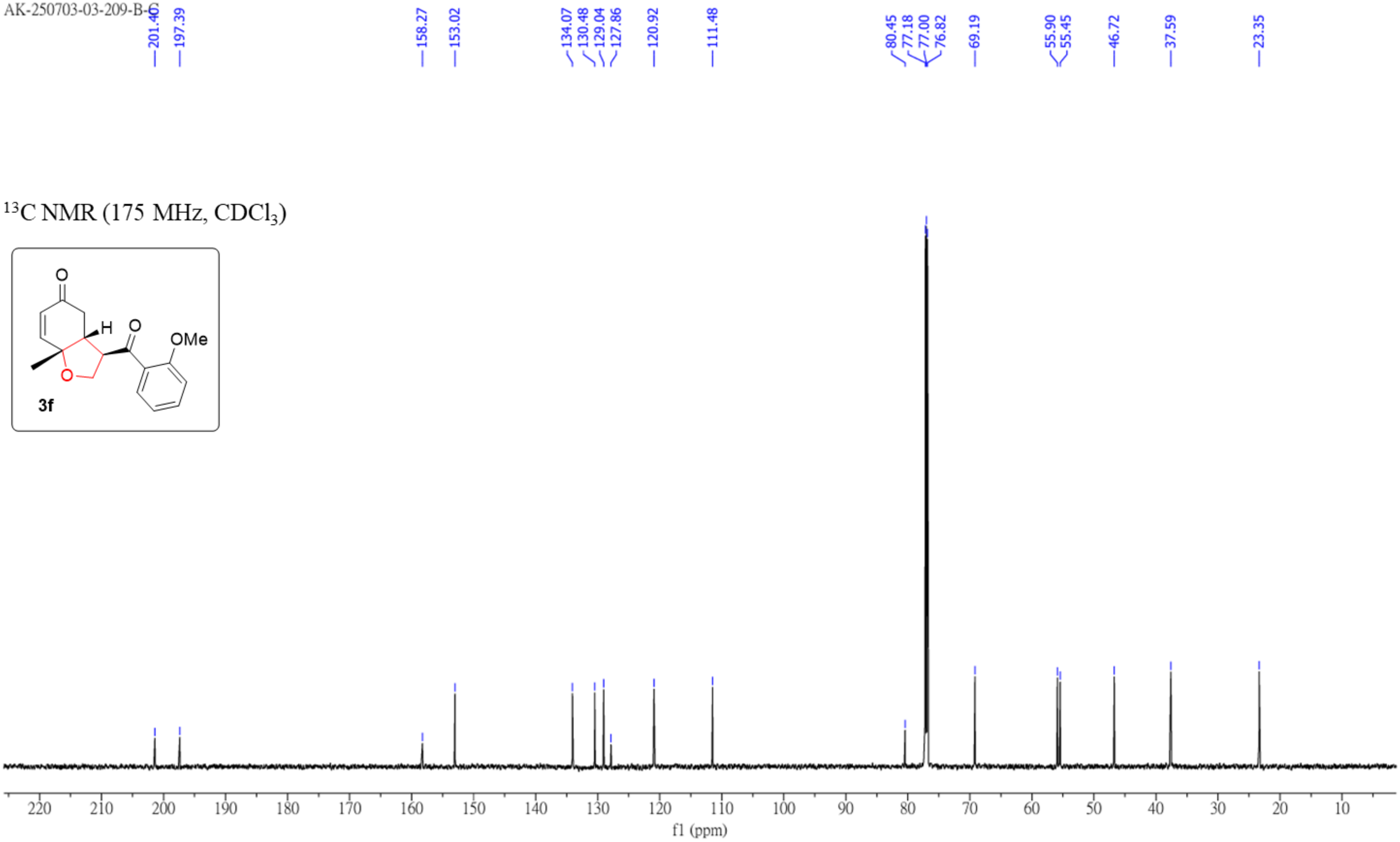

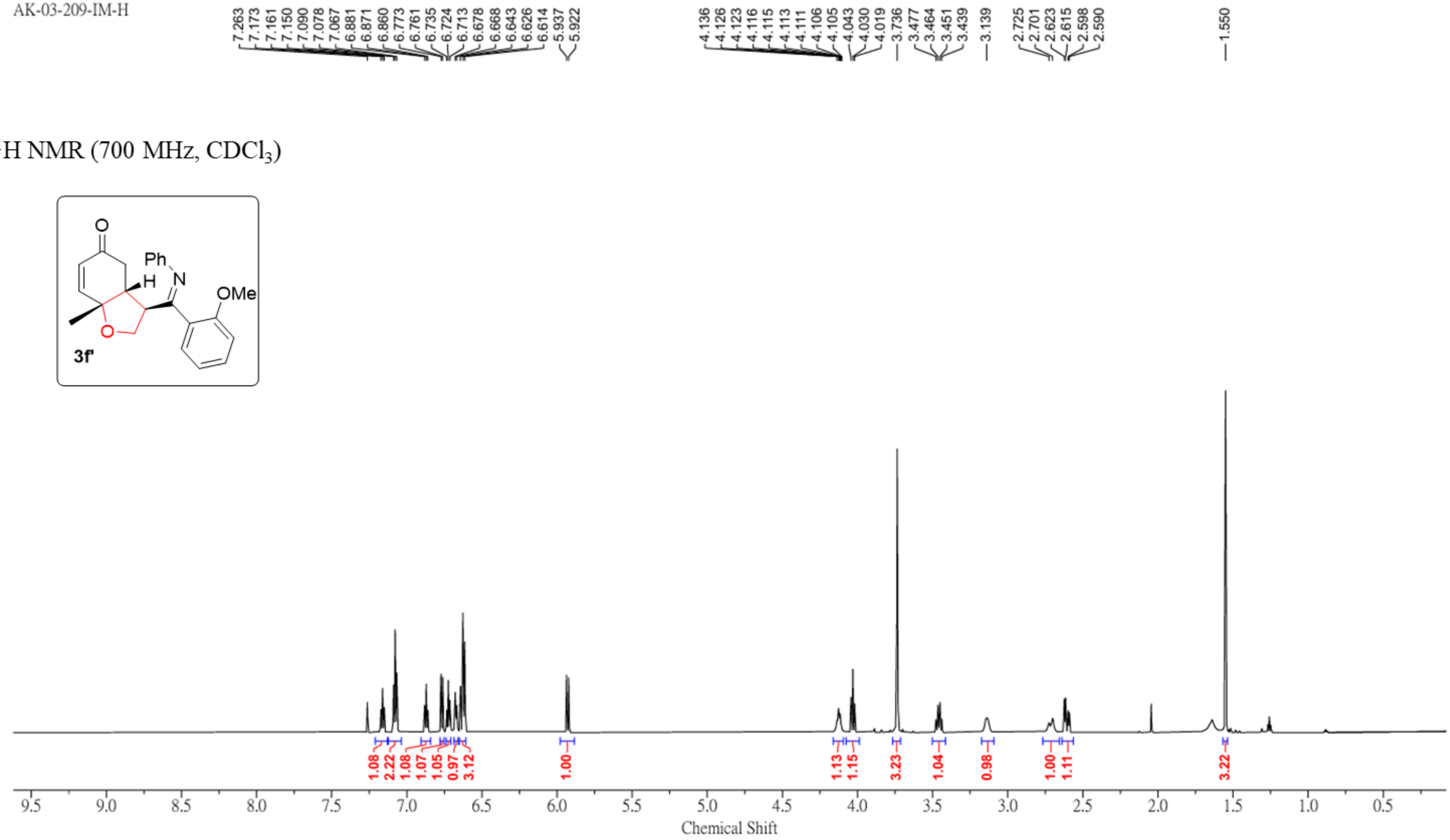

^{1}H NMR (700 MHz, $CDCl_3$)

AK-209-IMC

^{13}C NMR (175 MHz, $CDCl_3$)

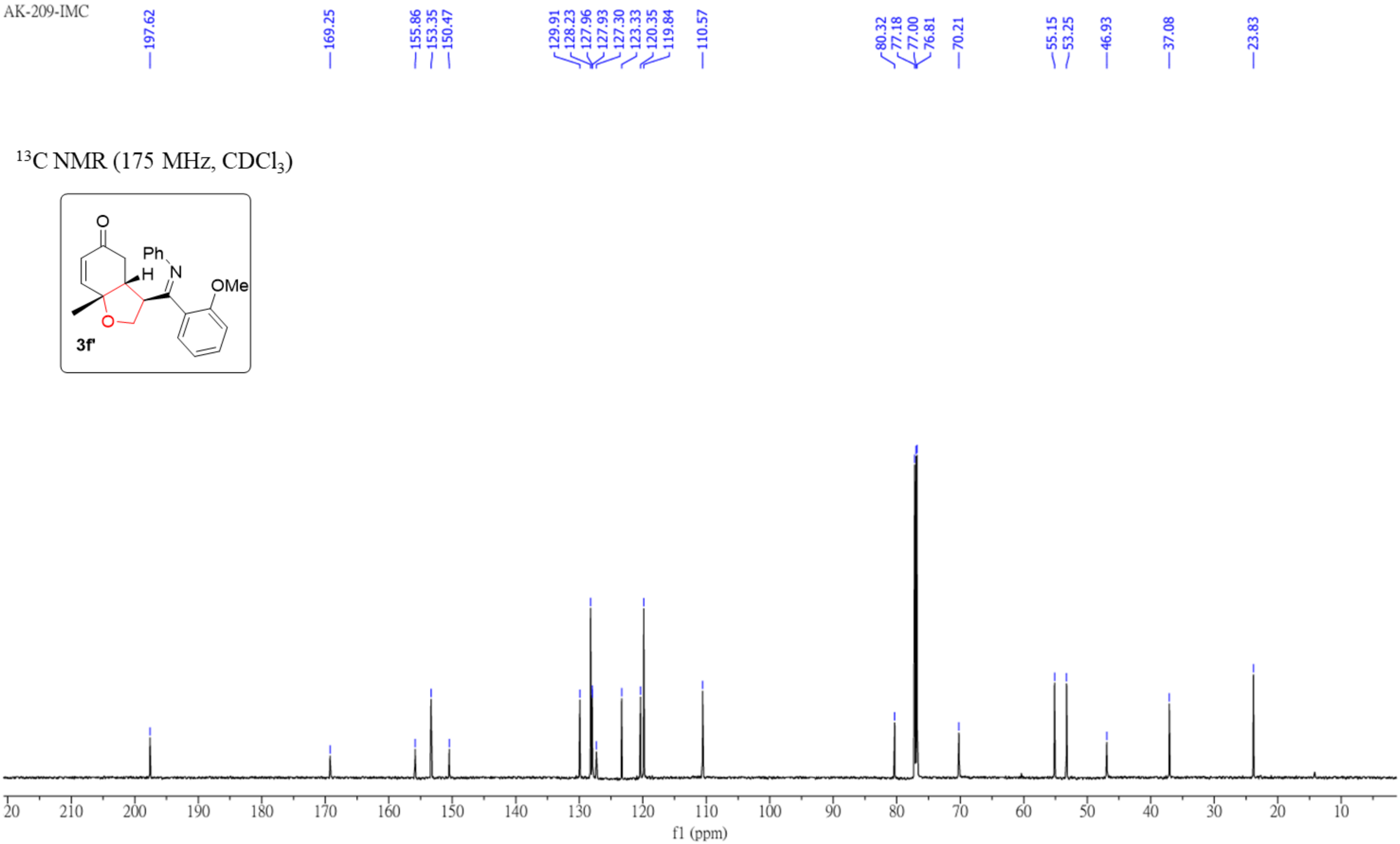

AK-03-212

^{1}H NMR (700 MHz, $CDCl_3$)

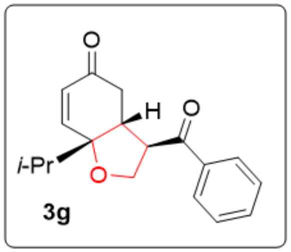

3g

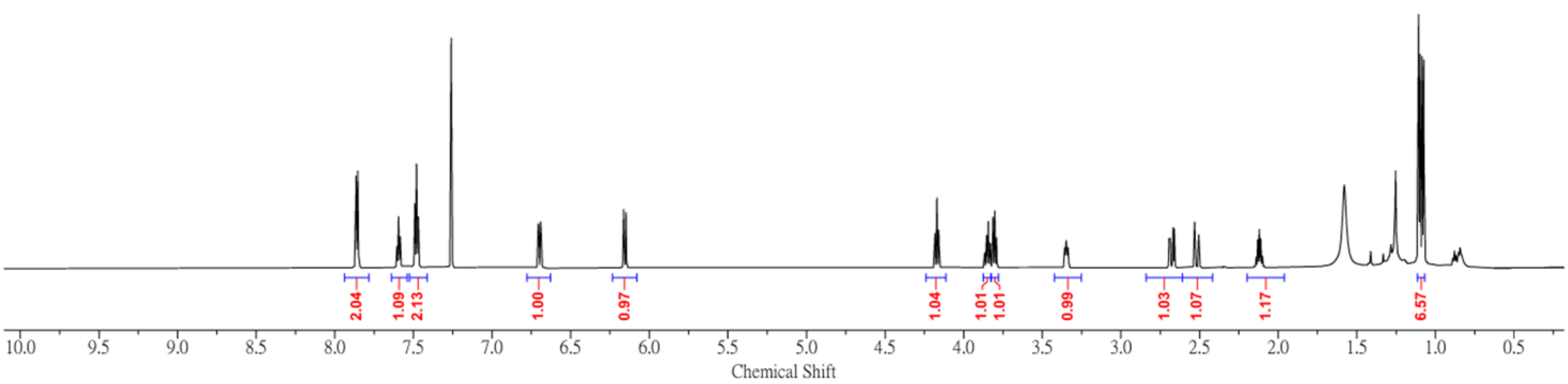

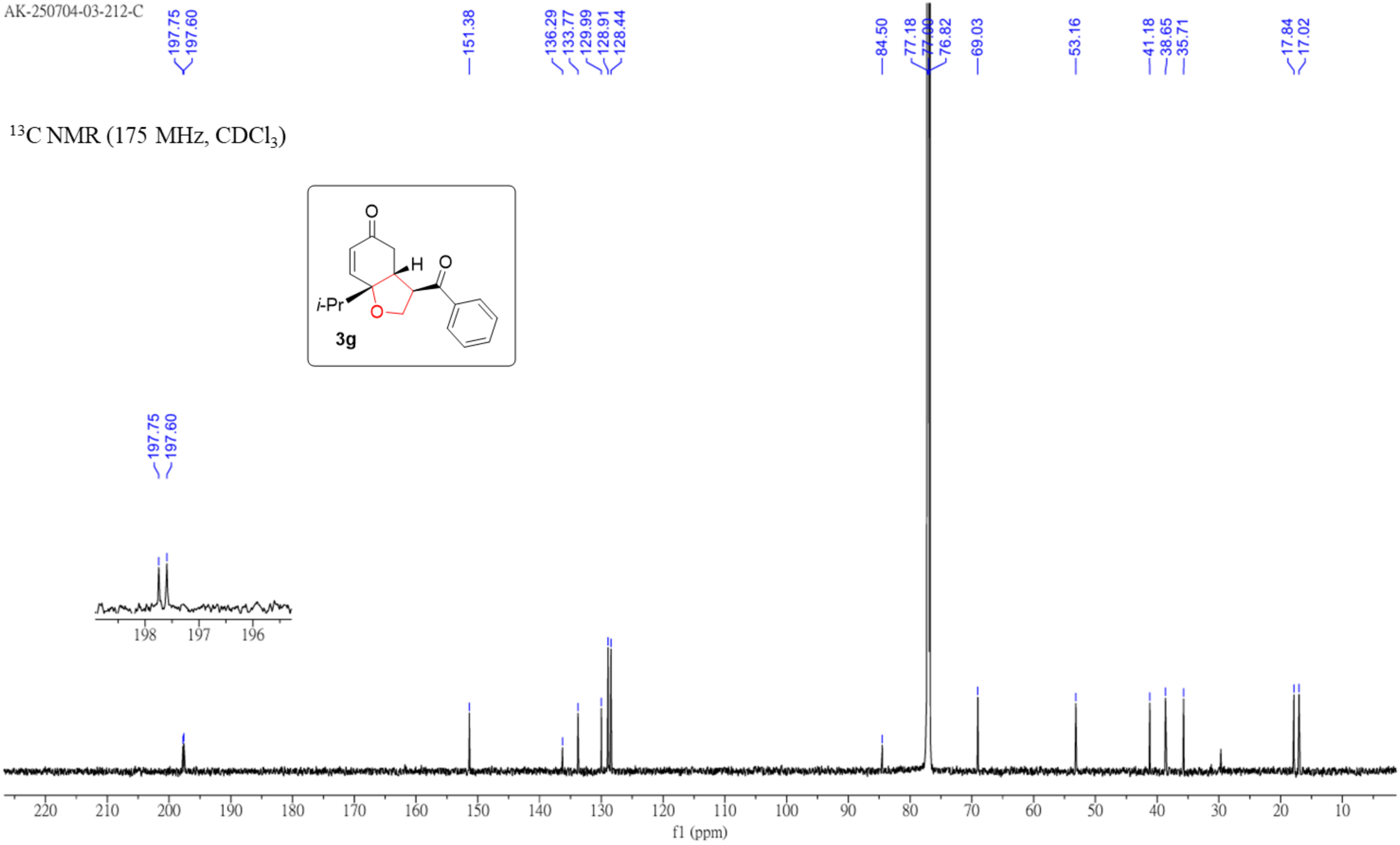
AK-250704-03-212-C
197.75
197.60
151.38
136.29
133.77
129.99
128.91
128.44
84.50
77.18
77.00
76.82
69.03
53.16
41.18
38.65
35.71
17.84
17.02
^{13}C NMR (175 MHz, $CDCl_3$)
O
H
O
i-Pr
O
3g
198
197
196
220 210 200 190 180 170 160 150 140 130 120 110 100 90 80 70 60 50 40 30 20 10
f1 (ppm)

^{1}H NMR (700 MHz, $CDCl_3$)

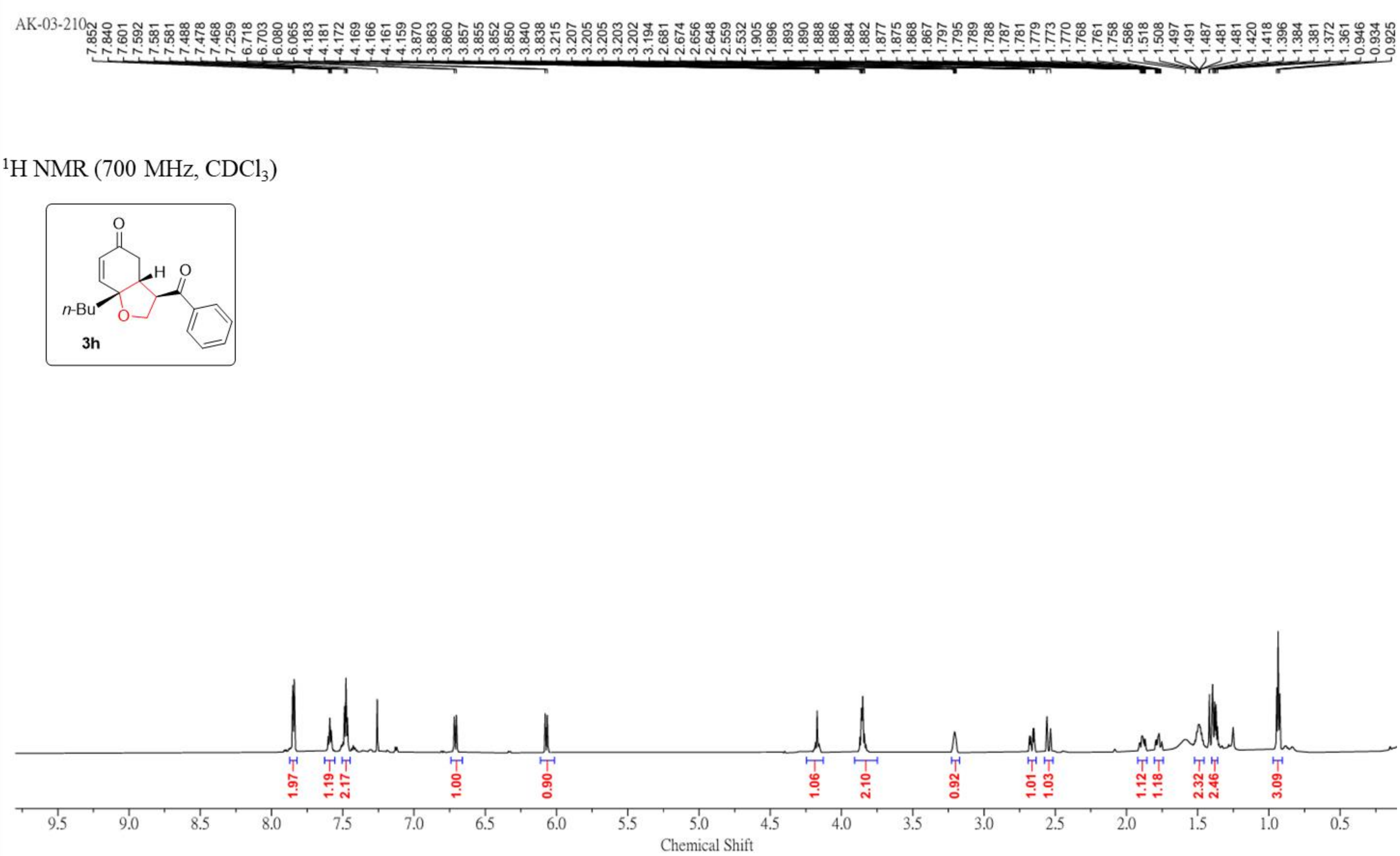

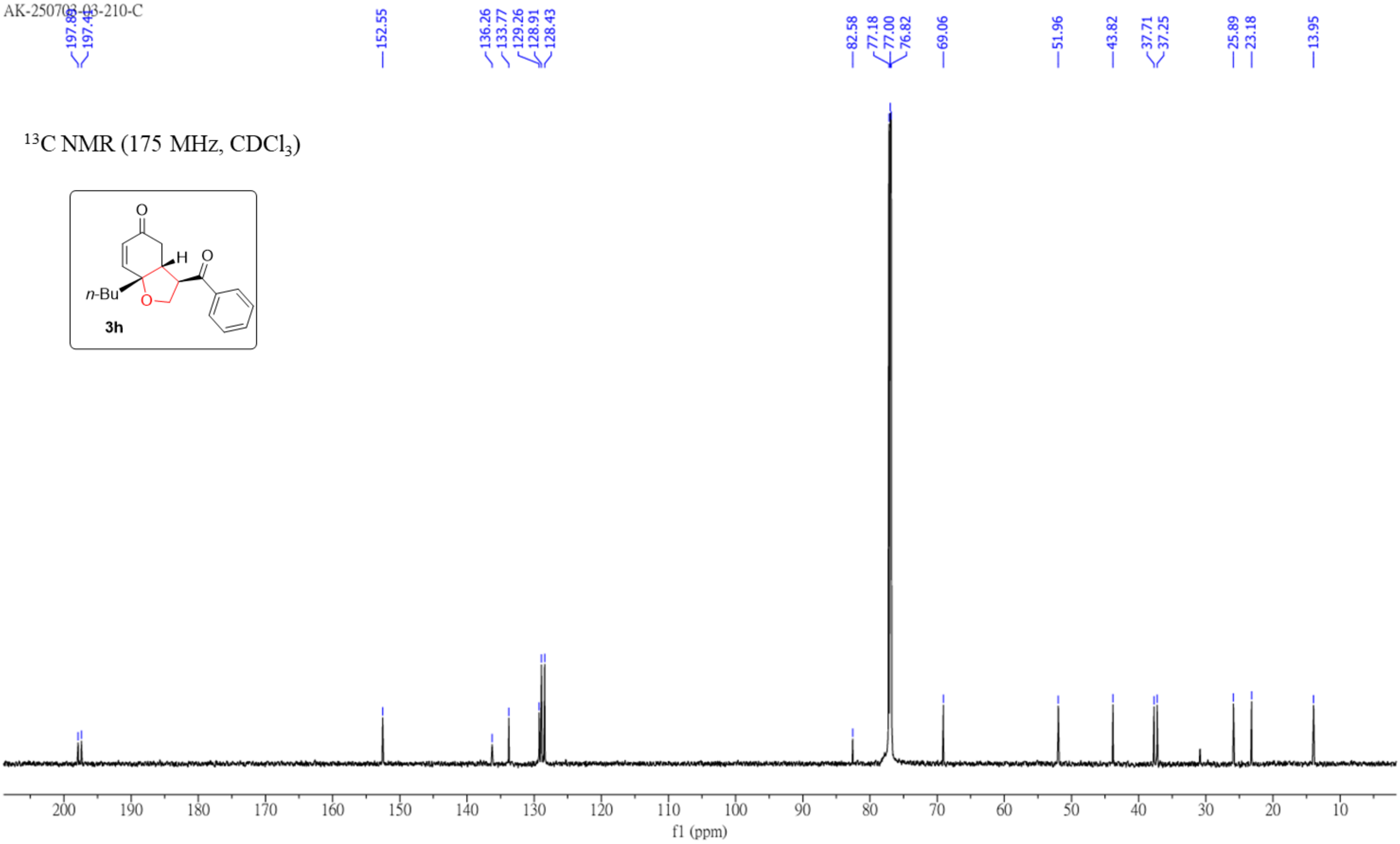
197.88
197.44
152.55
136.26
133.77
129.26
128.91
128.43
82.58
77.18
77.00
76.82
69.06
51.96
43.82
37.71
37.25
25.89
23.18
13.95
13C NMR (175 MHz, CDCl3)
O
H
O
n-Bu
O
3h
200 190 180 170 160 150 140 130 120 110 100 90 80 70 60 50 40 30 20 10
f1 (ppm)

AK-03-217

^1H NMR (700 MHz, $CDCl_3$)

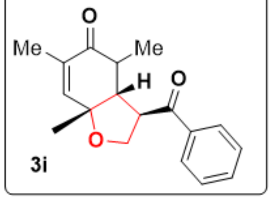

3i

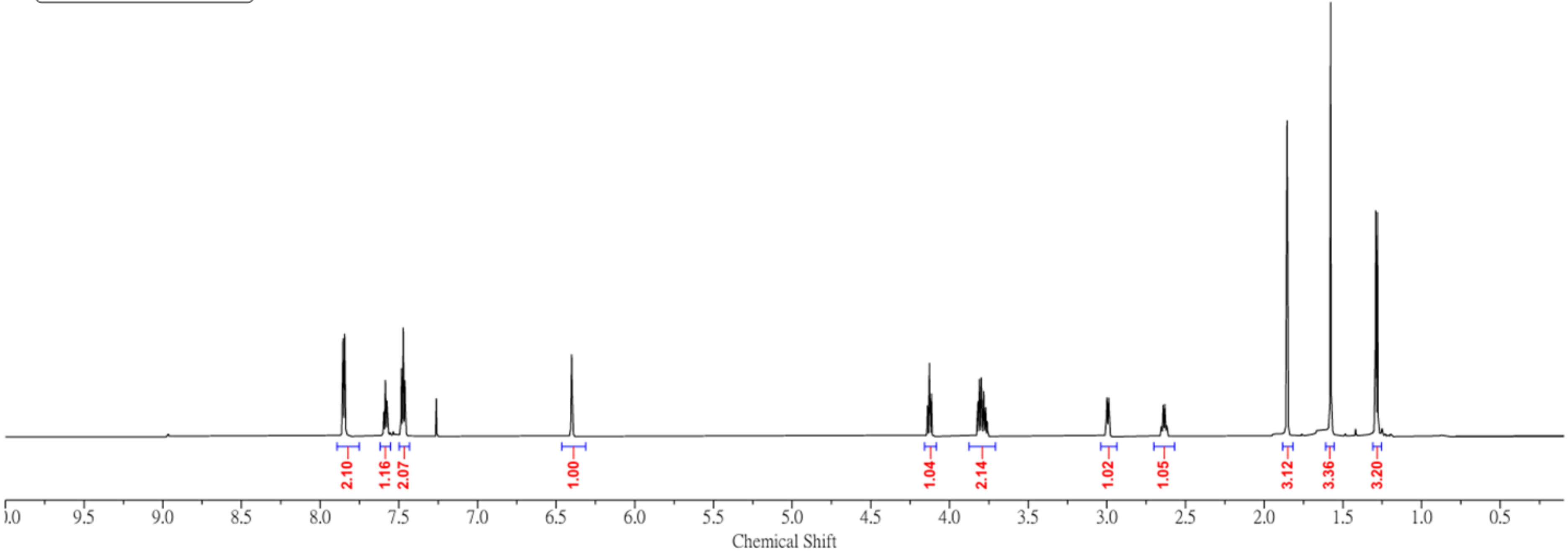

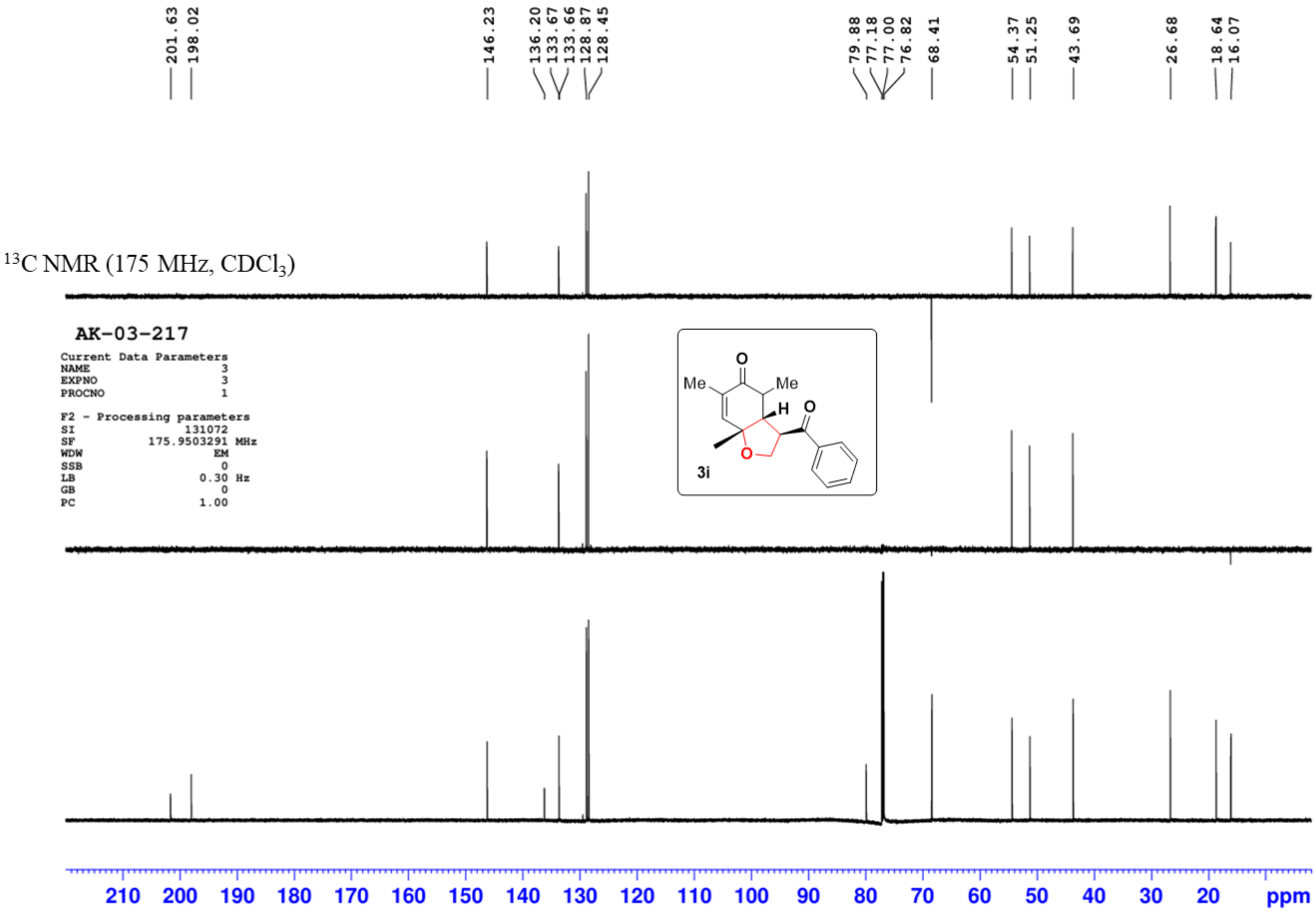
201.63
198.02
146.23
136.20
133.67
133.66
128.87
128.45
79.88
77.18
77.00
76.82
68.41
54.37
51.25
43.69
26.68
18.64
16.07
13C NMR (175 MHz, CDCl3)
AK-03-217
Current Data Parameters
NAME 3
EXPNO 3
PROCNO 1
F2 - Processing parameters
SI 131072
SF 175.9503291 MHz
WDW EM
SSB 0
LB 0.30 Hz
GB 0
PC 1.00
O
Me
Me
H
O
O
3i
210 200 190 180 170 160 150 140 130 120 110 100 90 80 70 60 50 40 30 20 ppm

AK-03-216

^{1}H NMR (700 MHz, $CDCl_3$)

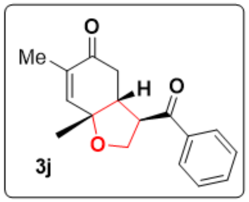

3j

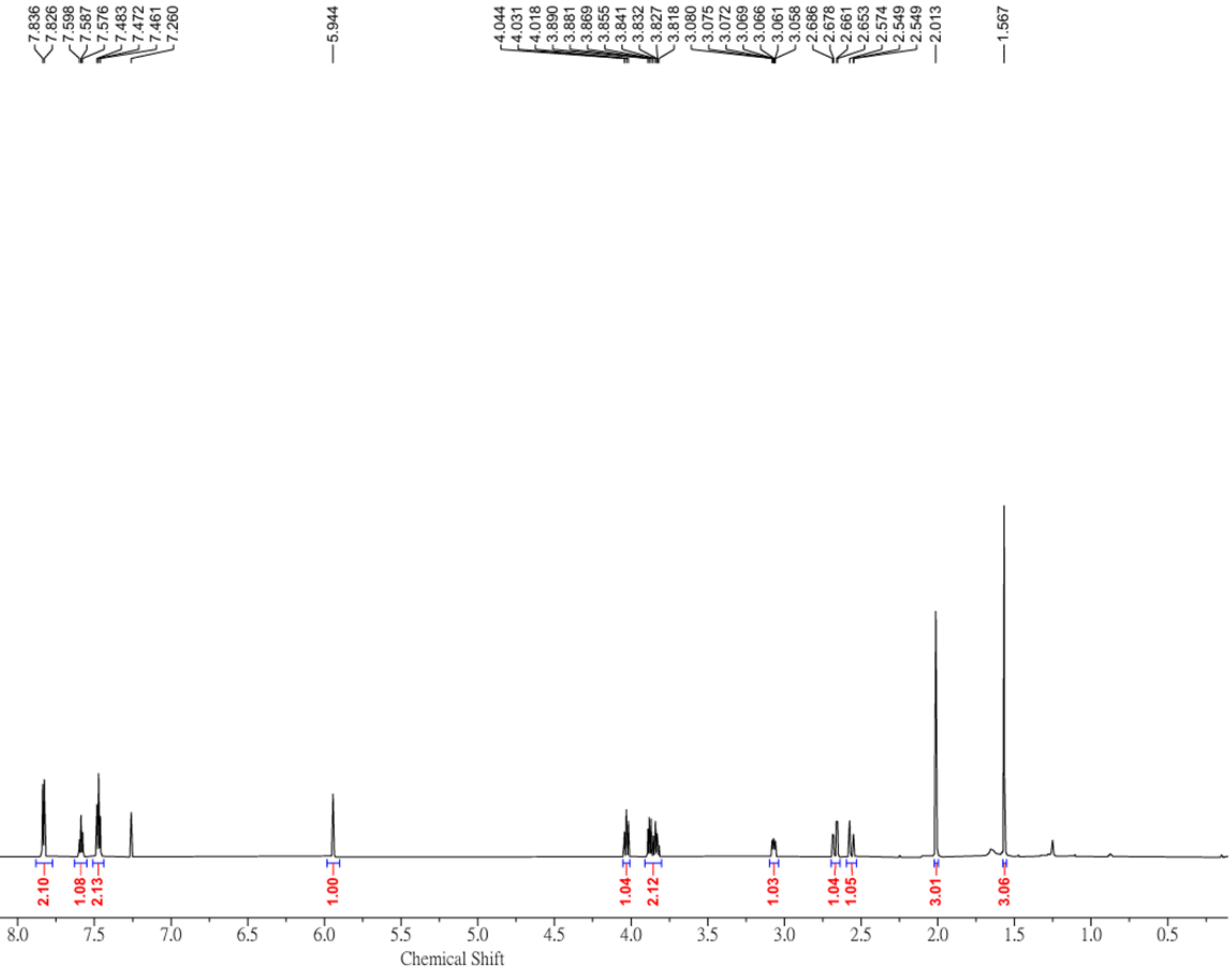

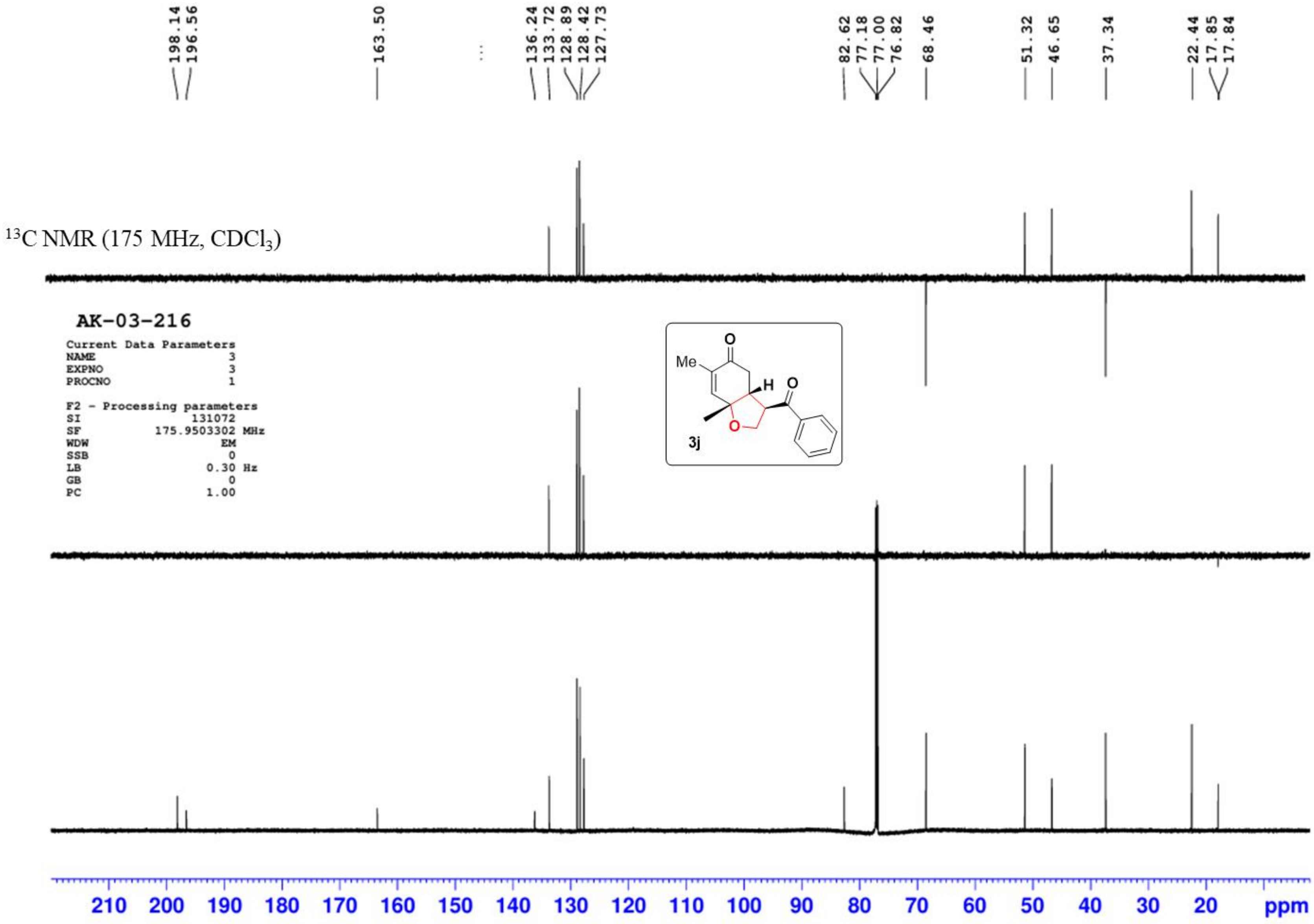
198.14
196.56
163.50
136.24
133.72
128.89
128.42
127.73
82.62
77.18
77.00
76.82
68.46
51.32
46.65
37.34
22.44
17.85
17.84
13C NMR (175 MHz, CDCl3)
AK-03-216
Current Data Parameters
NAME 3
EXPNO 3
PROCNO 1
F2 - Processing parameters
SI 131072
SF 175.9503302 MHz
WDW EM
SSB 0
LB 0.30 Hz
GB 0
PC 1.00
Me
O
H
3j
210 200 190 180 170 160 150 140 130 120 110 100 90 80 70 60 50 40 30 20 ppm

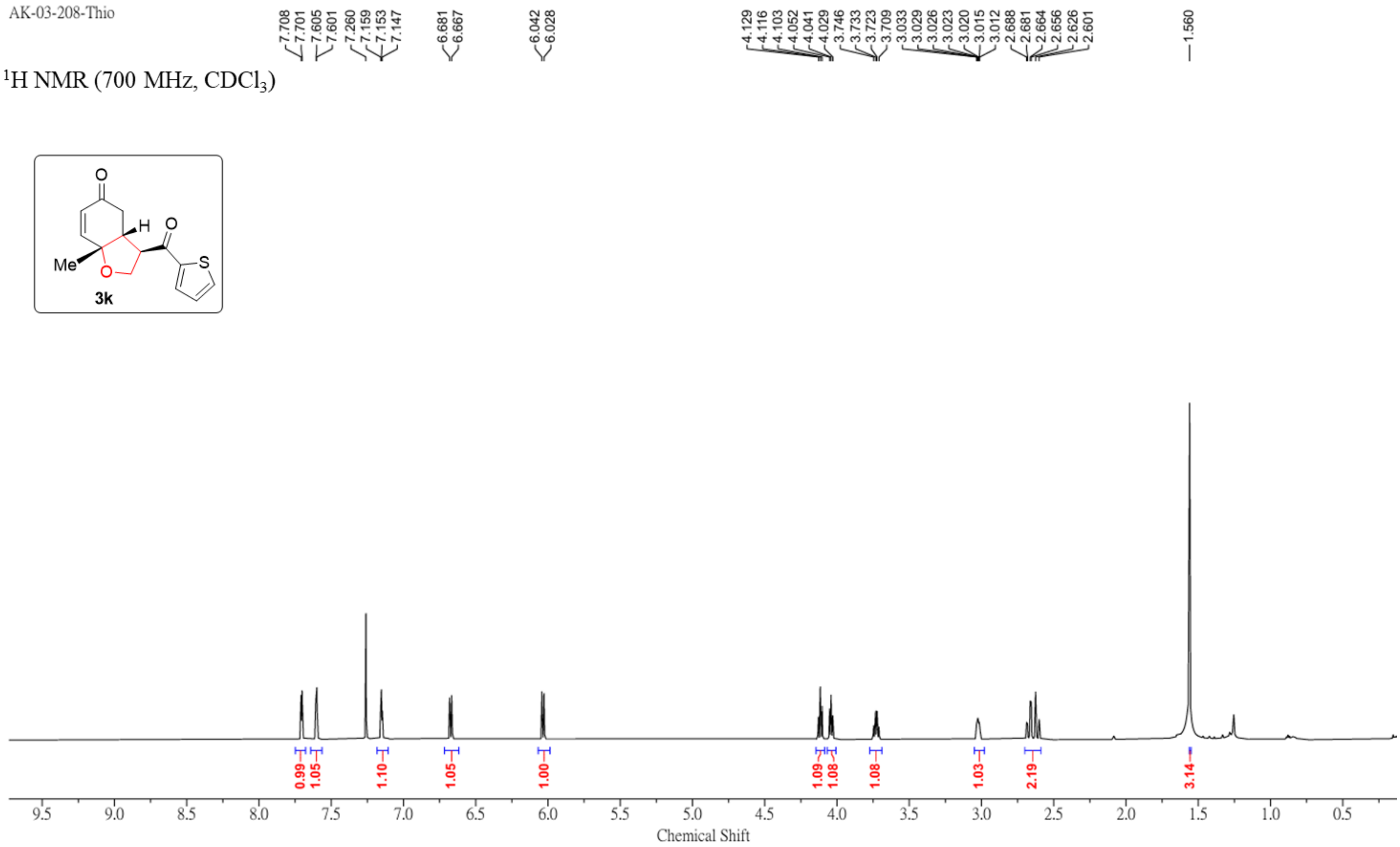
AK-03-208-Thio
^{1}H NMR (700 MHz, $CDCl_3$)
7.708
7.701
7.605
7.601
7.260
7.159
7.153
7.147
6.681
6.667
6.042
6.028
4.129
4.116
4.103
4.052
4.041
4.029
3.746
3.733
3.723
3.709
3.033
3.029
3.026
3.023
3.020
3.015
3.012
2.688
2.681
2.664
2.656
2.626
2.601
1.560
O
H
O
S
Me
O
3k
0.99
1.05
1.10
1.05
1.00
1.09
1.08
1.08
1.03
2.19
3.14
9.5
9.0
8.5
8.0
7.5
7.0
6.5
6.0
5.5
5.0
4.5
4.0
3.5
3.0
2.5
2.0
1.5
1.0
0.5
Chemical Shift

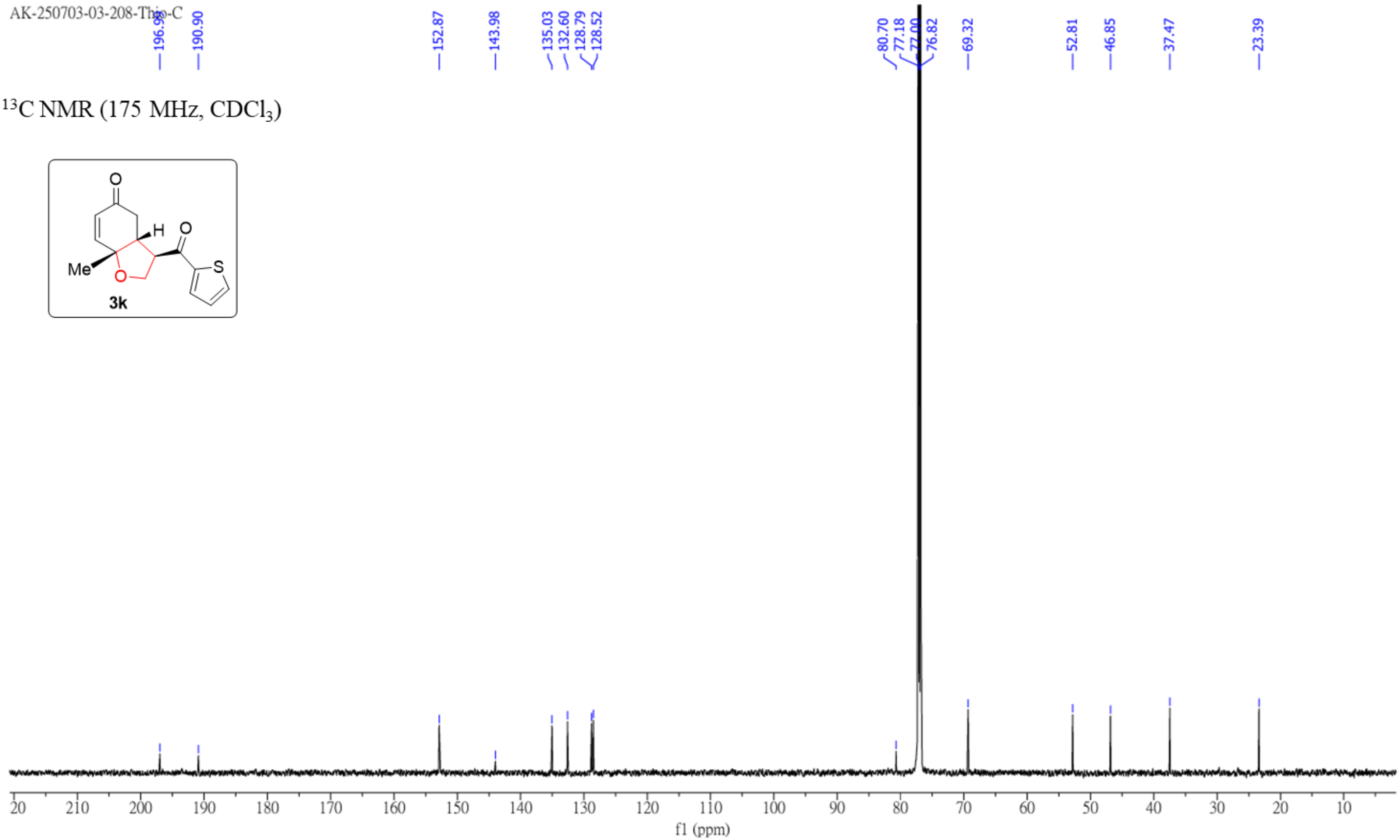
AK-250703-03-208-Thio-C
190.90
152.87
143.98
135.03
132.60
128.79
128.52
80.70
77.18
77.00
76.82
69.32
52.81
46.85
37.47
23.39
^{13}C NMR (175 MHz, $CDCl_3$)
O
H
O
S
Me
O
3k
f1 (ppm)

AK-03-211

^1H NMR (700 MHz, $CDCl_3$)

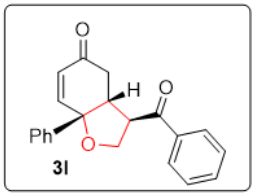

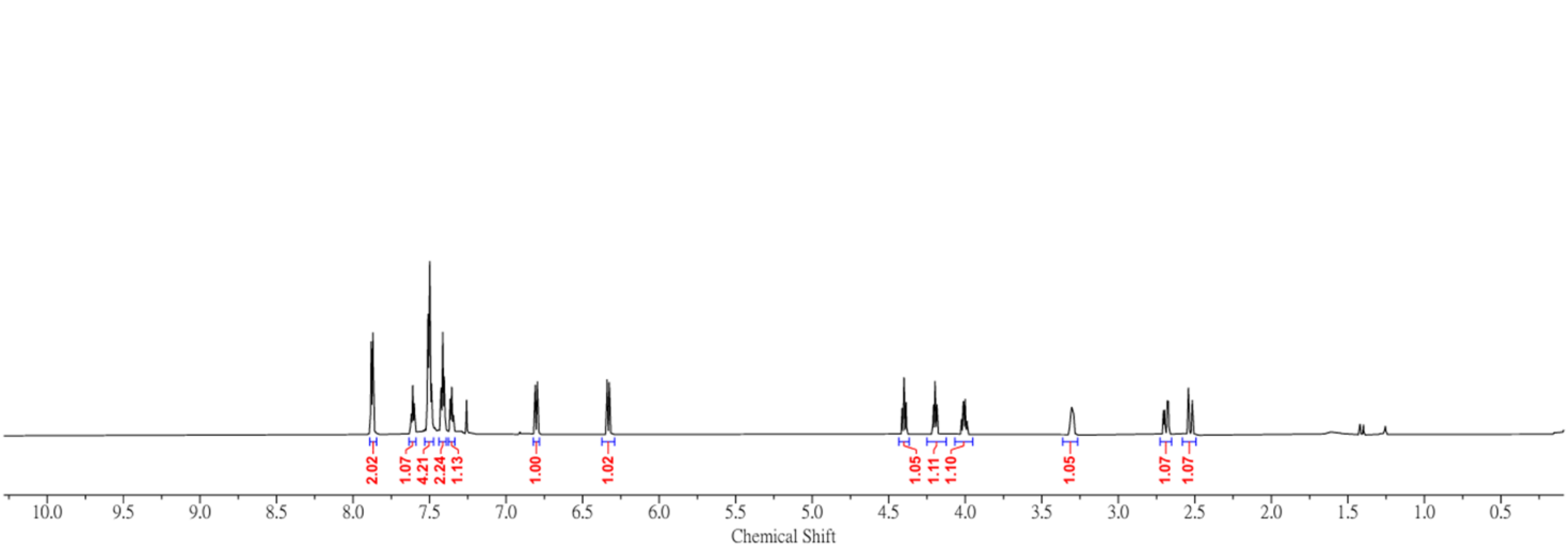

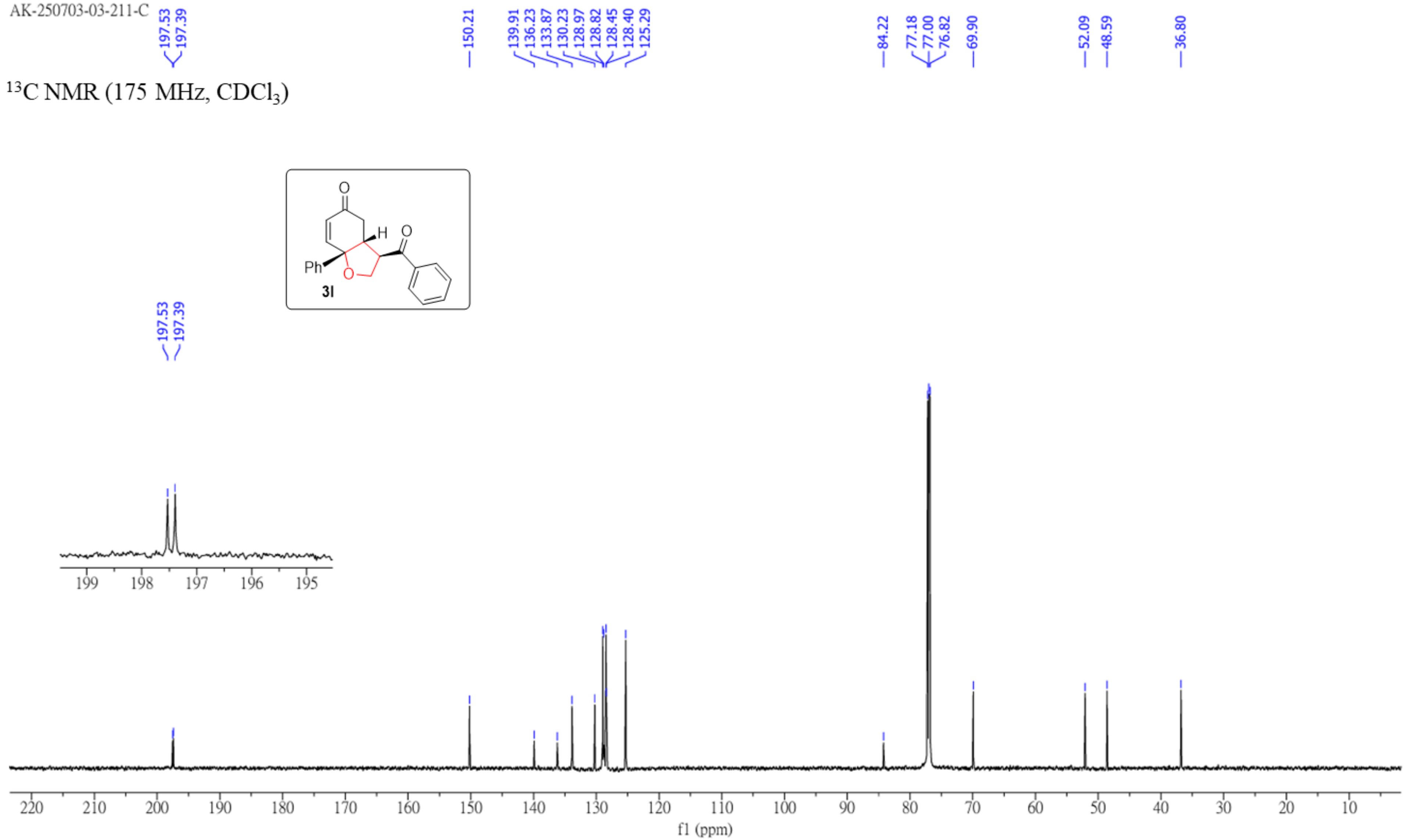
AK-250703-03-211-C
197.53
197.39
150.21
139.91
136.23
133.87
130.23
128.97
128.82
128.45
128.40
125.29
84.22
77.18
77.00
76.82
69.90
52.09
48.59
36.80
^{13}C NMR (175 MHz, $CDCl_3$)
O
H
O
Ph
O
3l
197.53
197.39
199
198
197
196
195
220
210
200
190
180
170
160
150
140
130
120
110
100
90
80
70
60
50
40
30
20
10
f1 (ppm)

AK-03-219-D

^{1}H NMR (700 MHz, DMSO)

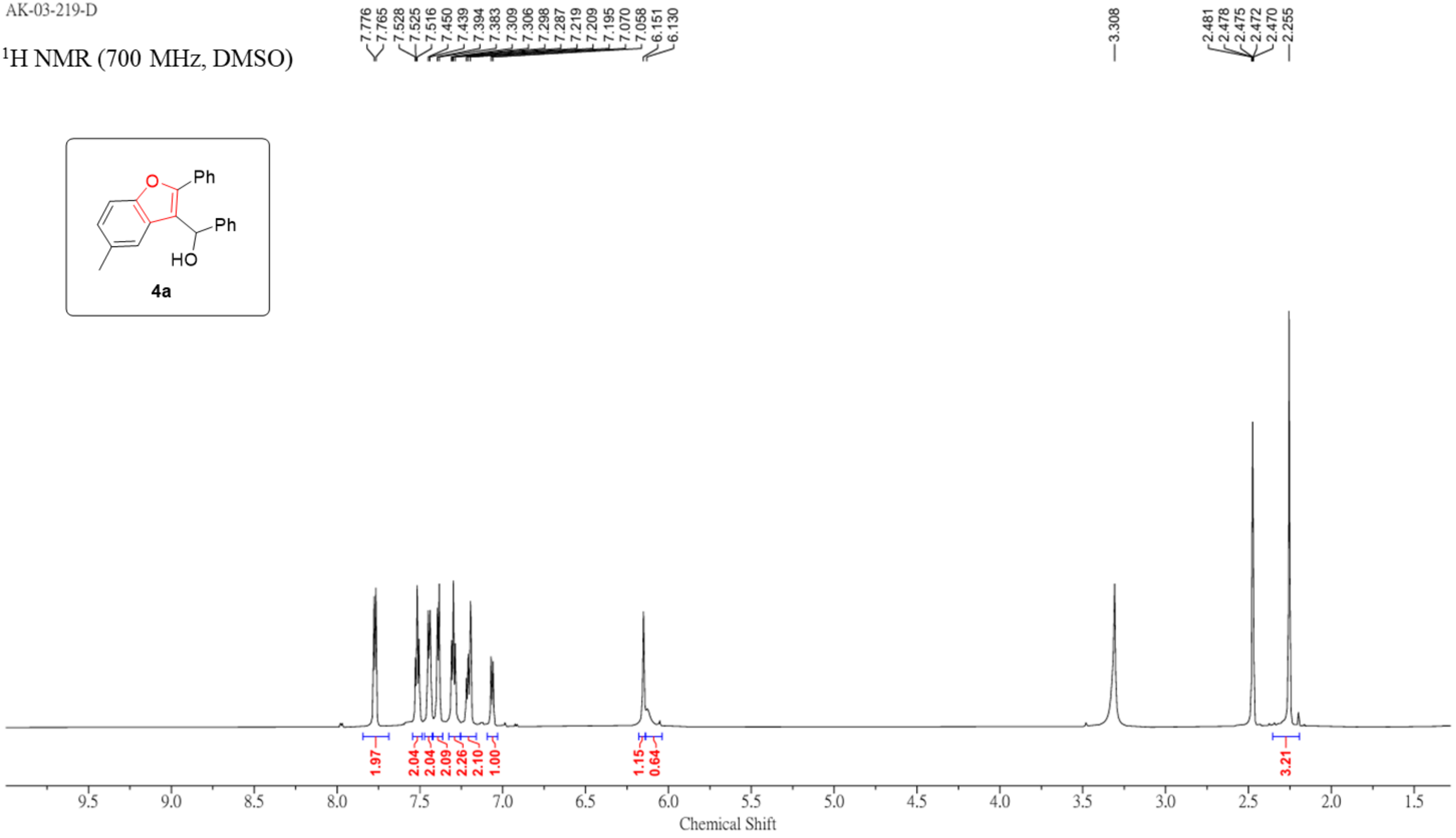

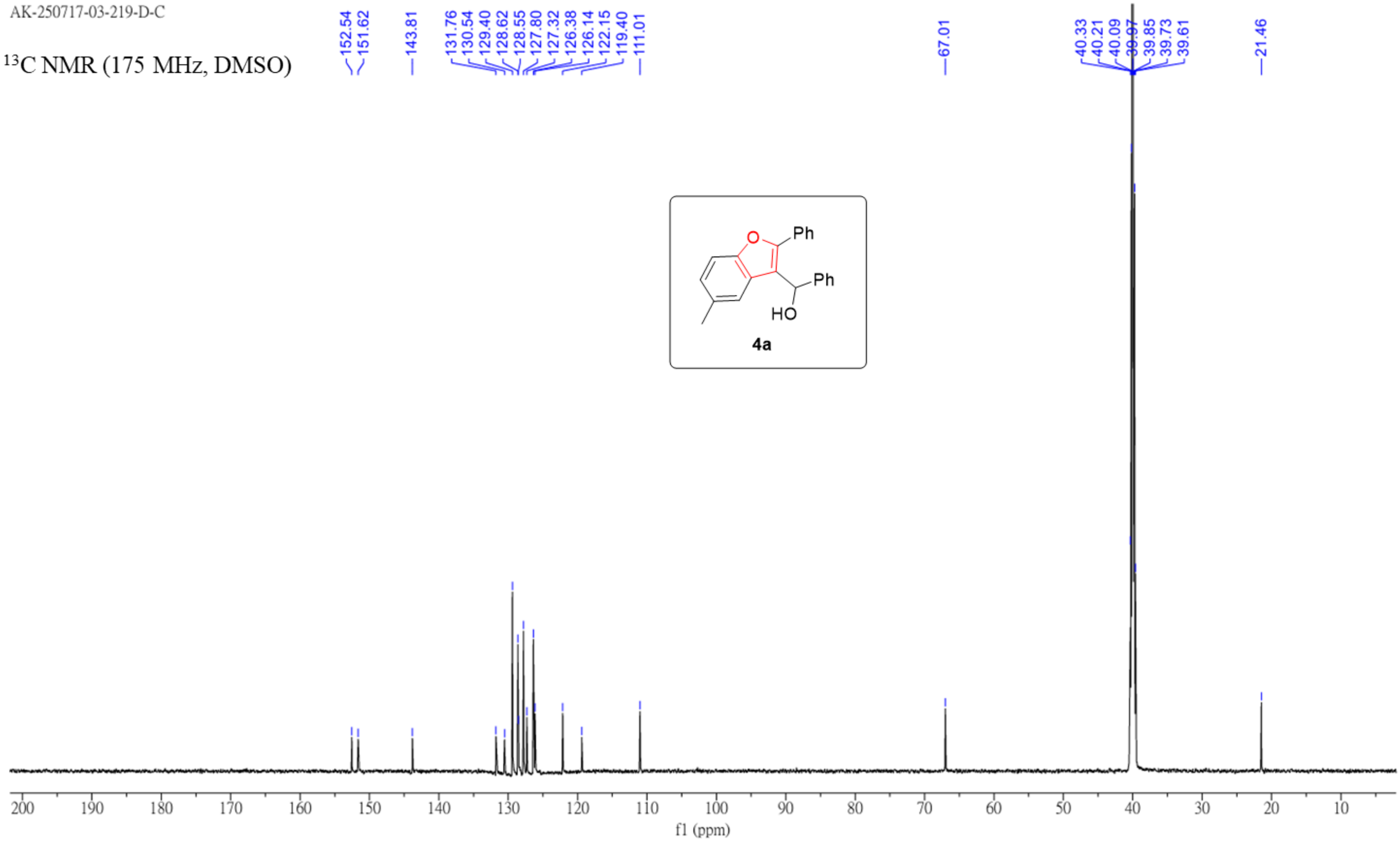

AK-250717-03-219-D-C
13C NMR (175 MHz, DMSO)
152.54
151.62
143.81
131.76
130.54
129.40
128.62
128.55
127.80
127.32
126.38
126.14
122.15
119.40
111.01
67.01
40.33
40.21
40.09
39.97
39.85
39.73
39.61
21.46
O
Ph
Ph
HO
4a
200
190
180
170
160
150
140
130
120
110
100
90
80
70
60
50
40
30
20
10
f1 (ppm)

AK-03-218-D

^{1}H NMR (700 MHz, DMSO)

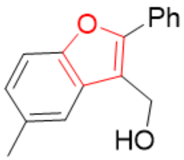

4b

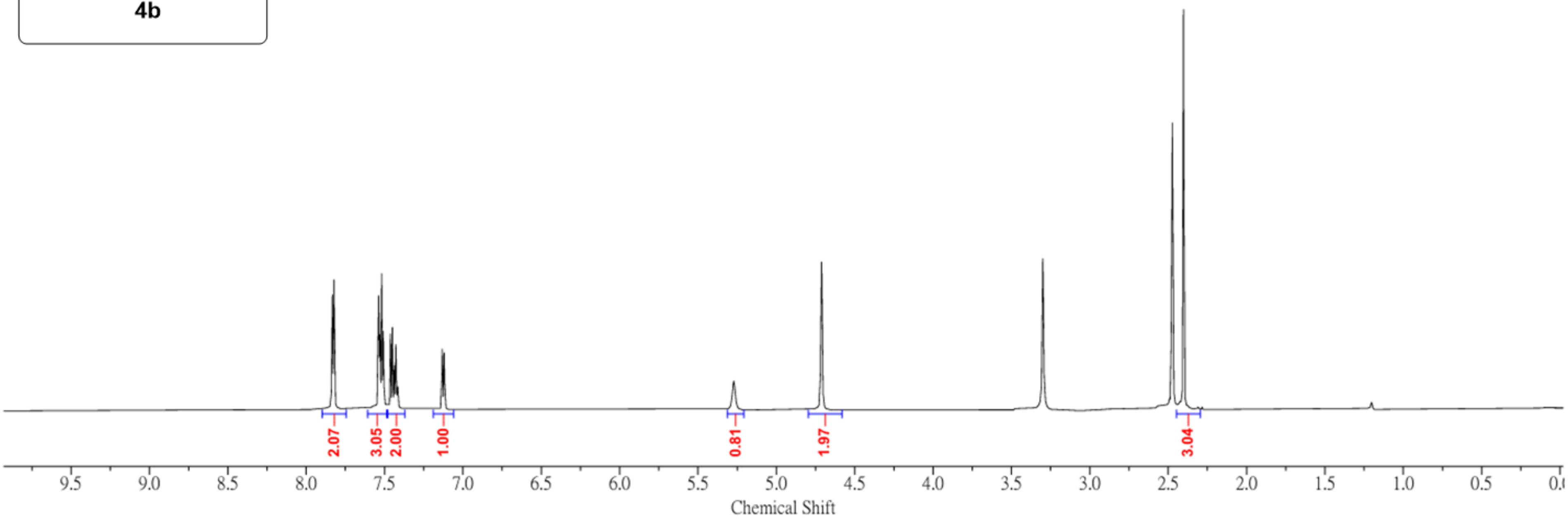

AK-250717-03-218-D-C

^{13}C NMR (175 MHz, DMSO)

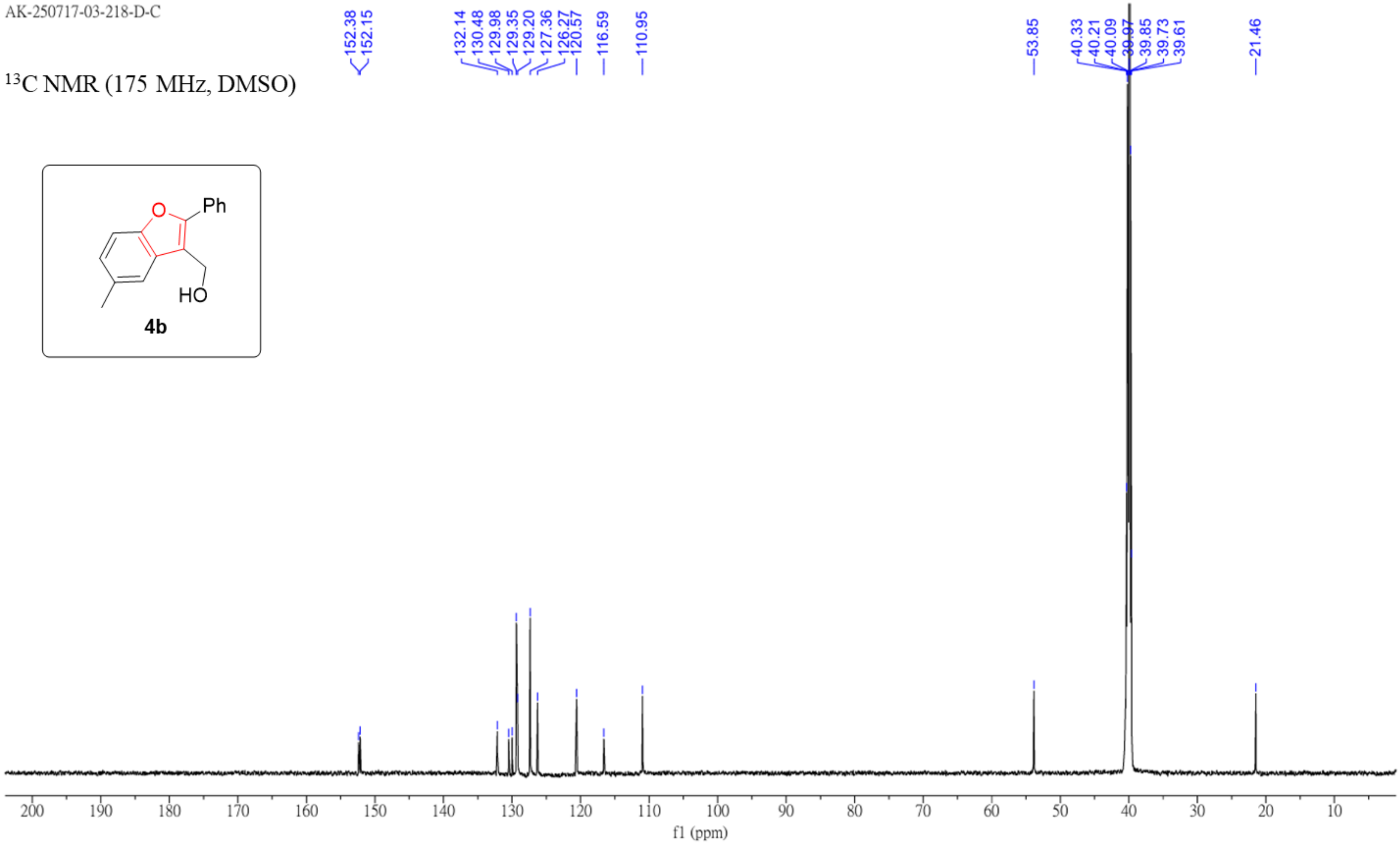

^{1}H NMR (400 MHz, $CDCl_3$)

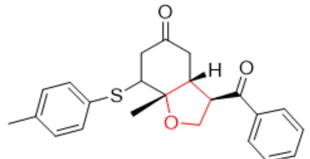

5a (dr=1.1:1)

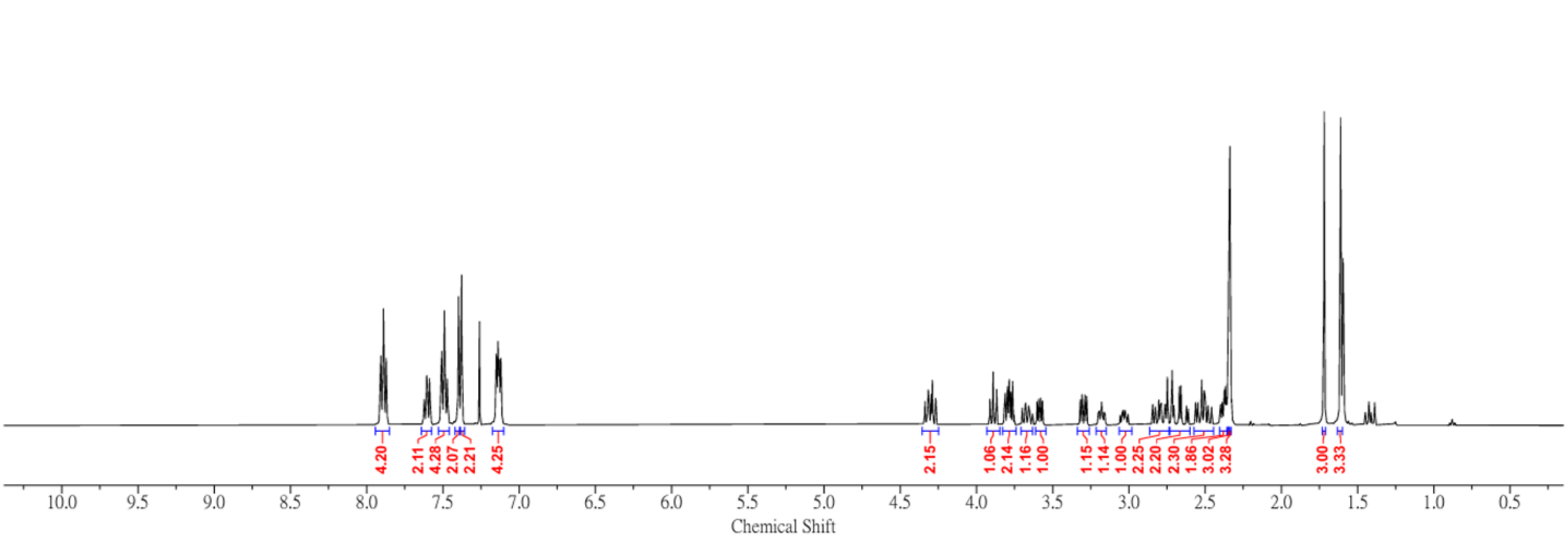

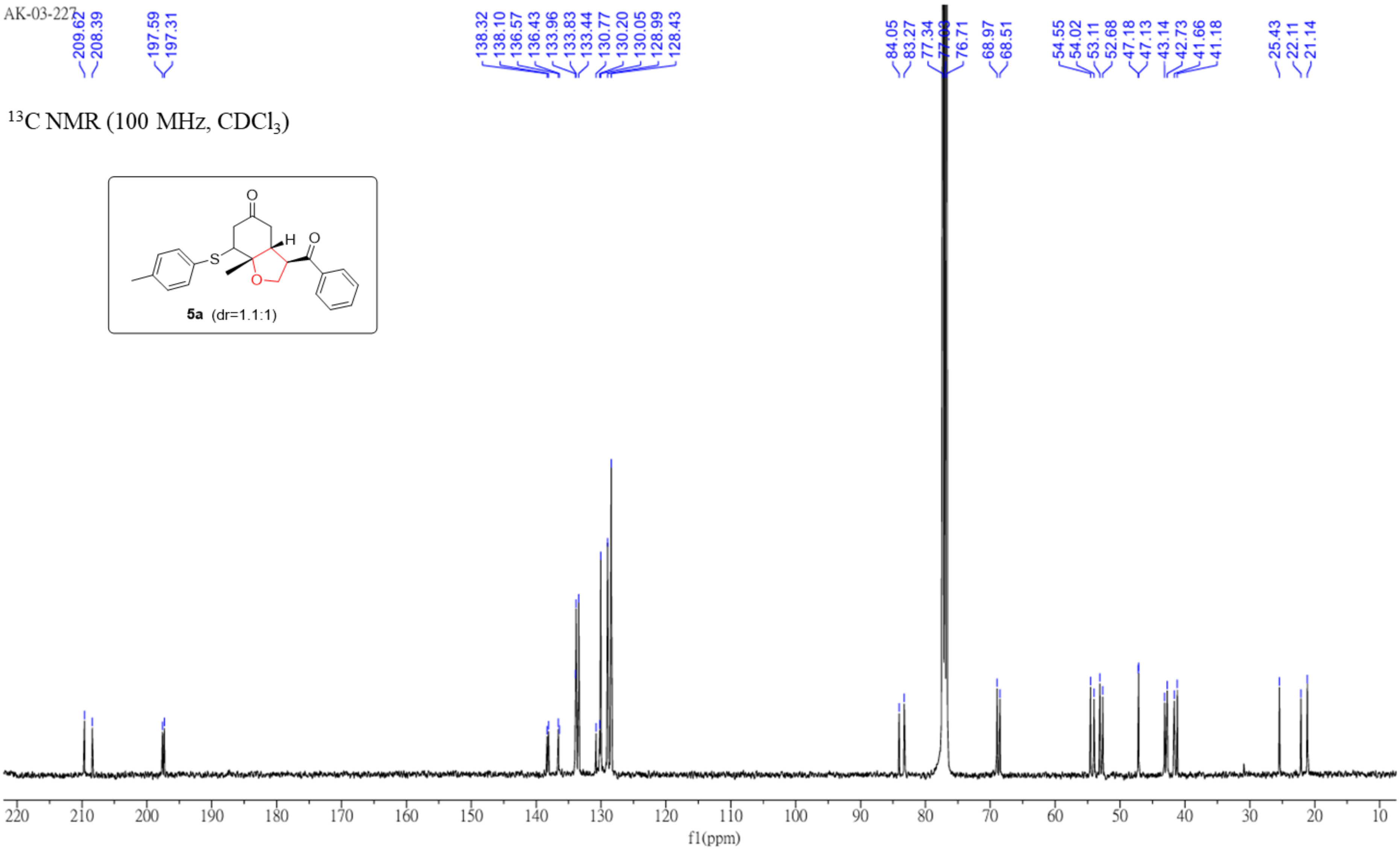
AK-03-227
209.62
208.39
197.59
197.31
138.32
138.10
136.57
136.43
133.96
133.83
133.44
130.77
130.20
130.05
128.99
128.43
84.05
83.27
77.34
76.71
68.97
68.51
54.55
54.02
53.11
52.68
47.18
47.13
43.14
42.73
41.66
41.18
25.43
22.11
21.14
^{13}C NMR (100 MHz, $CDCl_3$)
5a (dr=1.1:1)
f1(ppm)

^{1}H NMR (400 MHz, $CDCl_3$)

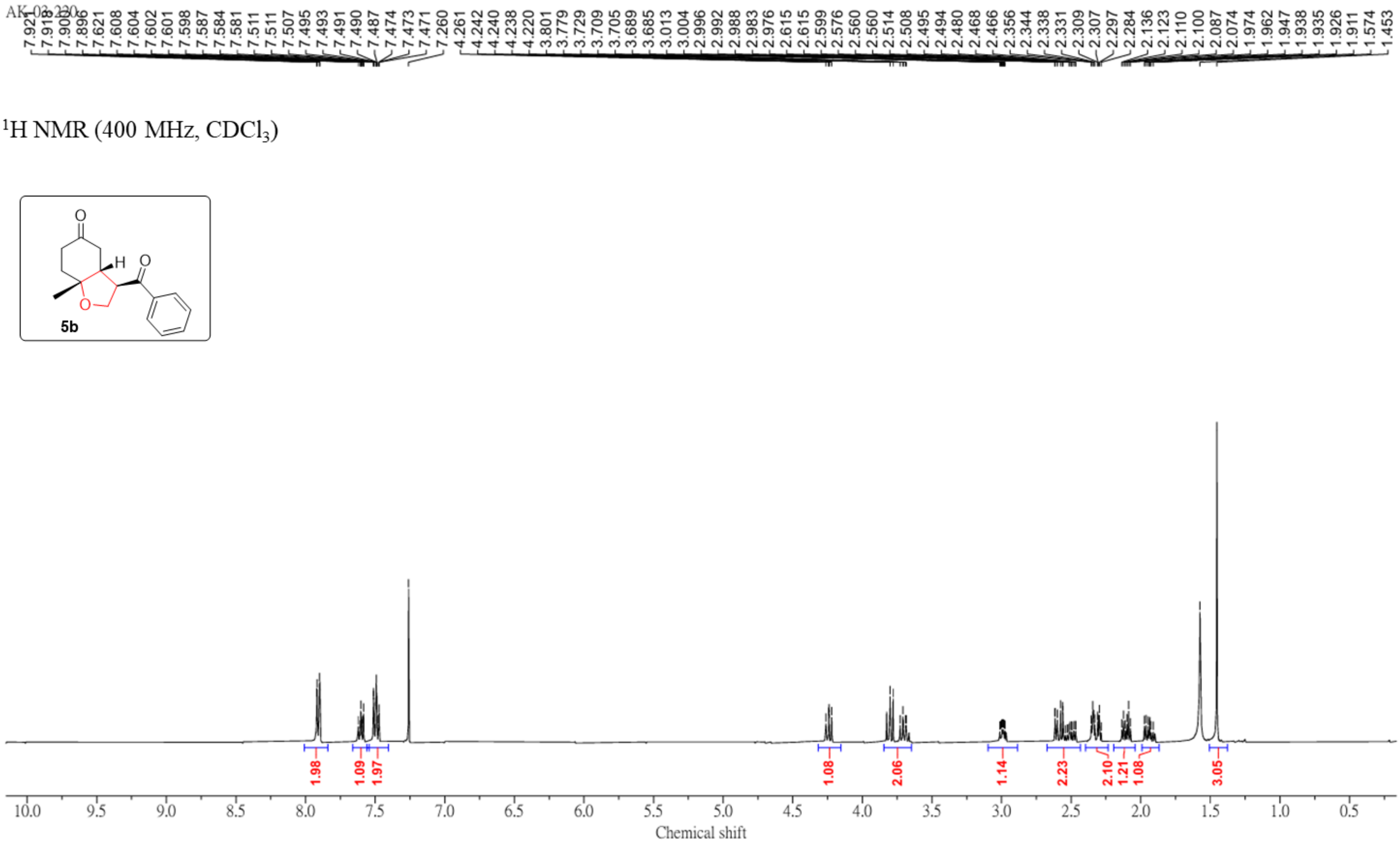

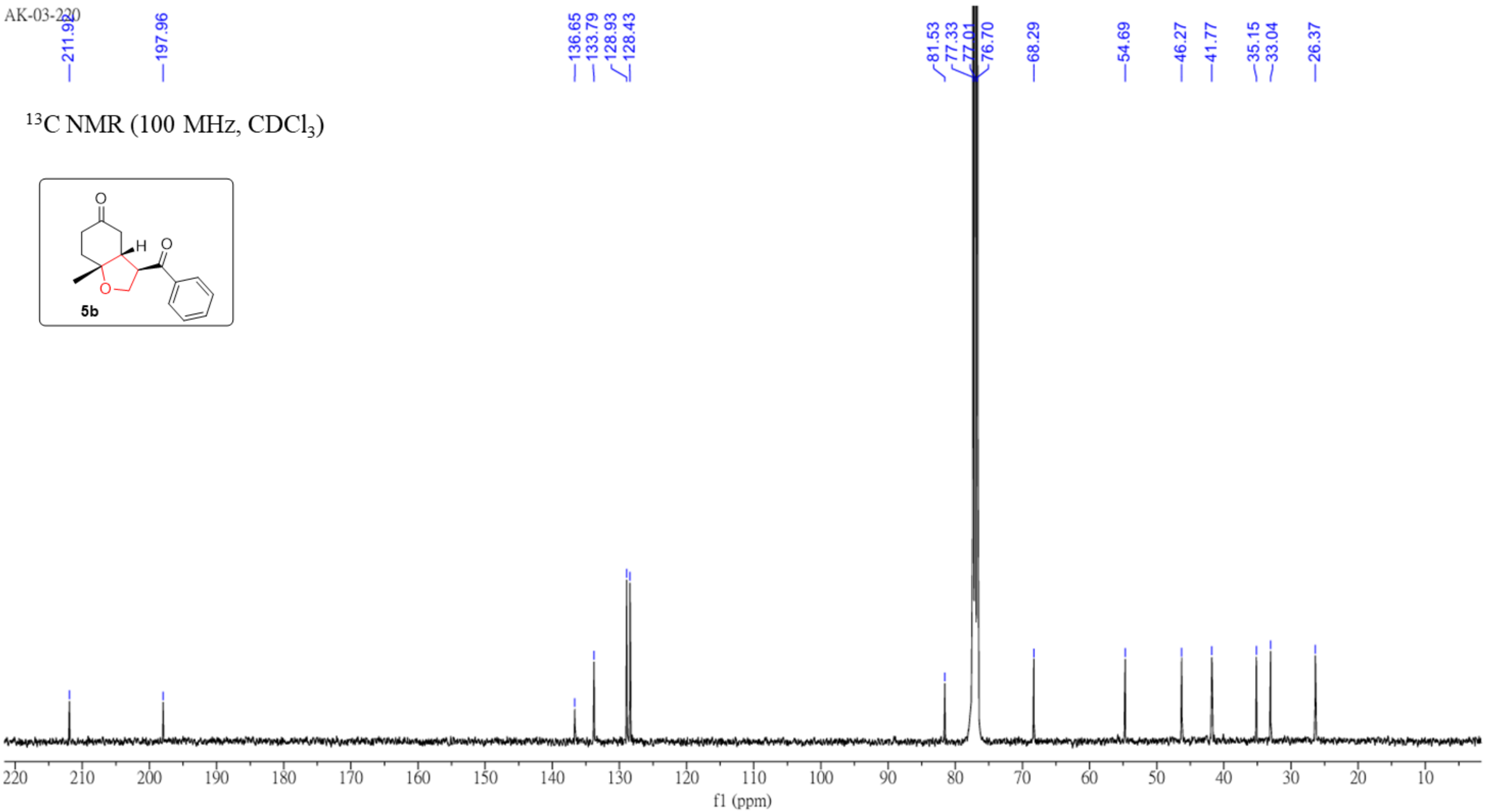
AK-03-220
211.92
197.96
136.65
133.79
128.93
128.43
81.53
77.33
77.01
76.70
68.29
54.69
46.27
41.77
35.15
33.04
26.37
^{13}C NMR (100 MHz, $CDCl_3$)
O
H
O
O
5b
f1 (ppm)

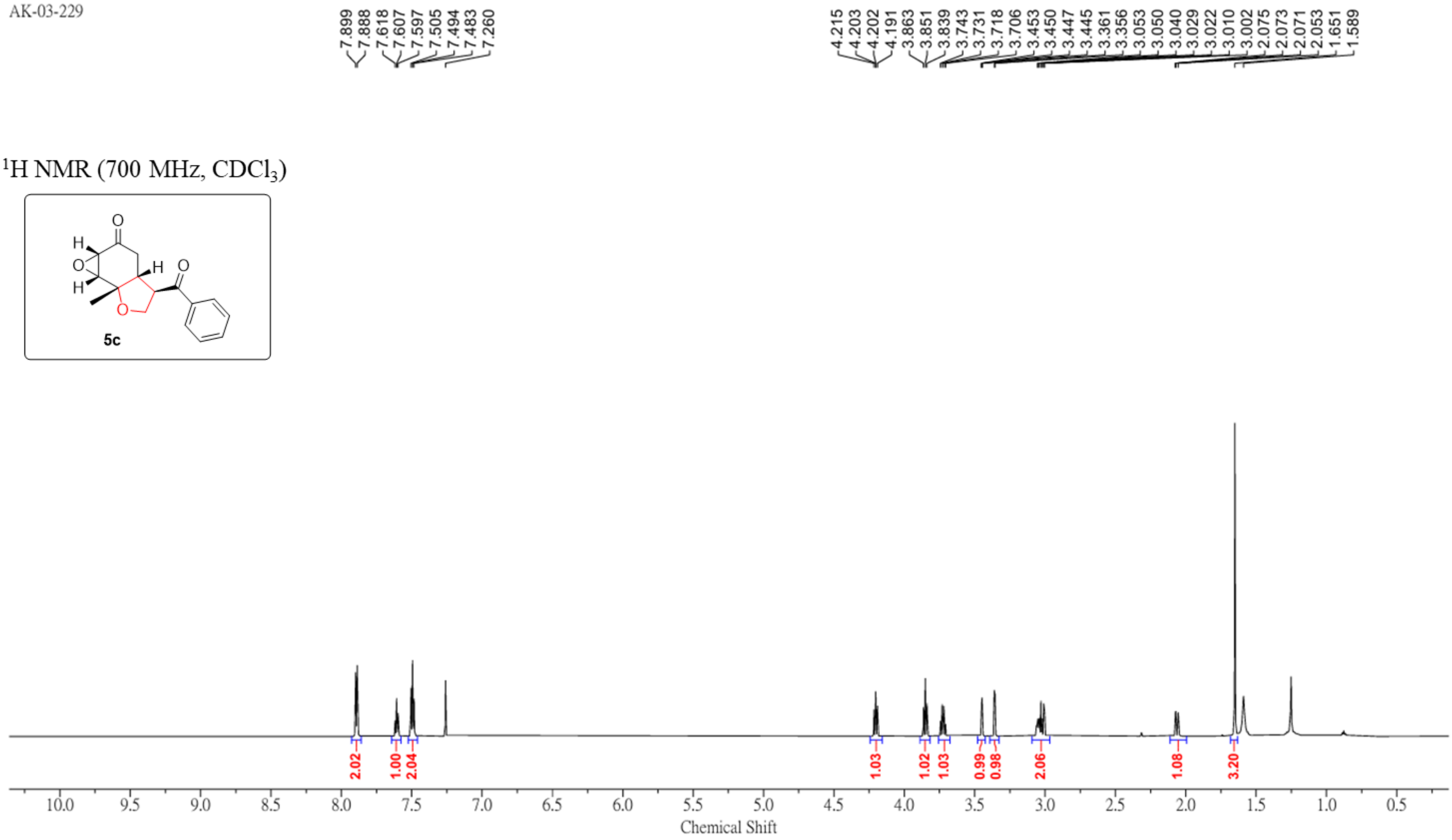
AK-03-229
7.899
7.888
7.618
7.607
7.597
7.505
7.494
7.483
7.260
4.215
4.203
4.202
4.191
3.863
3.851
3.839
3.743
3.731
3.718
3.706
3.453
3.450
3.447
3.445
3.361
3.356
3.053
3.050
3.040
3.029
3.022
3.010
3.002
2.075
2.073
2.071
2.053
1.651
1.589
¹H NMR (700 MHz, CDCl3)
O
H
O
H
H
O
O
5c
2.02
1.00
2.04
1.03
1.02
1.03
0.99
0.98
2.06
1.08
3.20
10.0
9.5
9.0
8.5
8.0
7.5
7.0
6.5
6.0
5.5
5.0
4.5
4.0
3.5
3.0
2.5
2.0
1.5
1.0
0.5
Chemical Shift

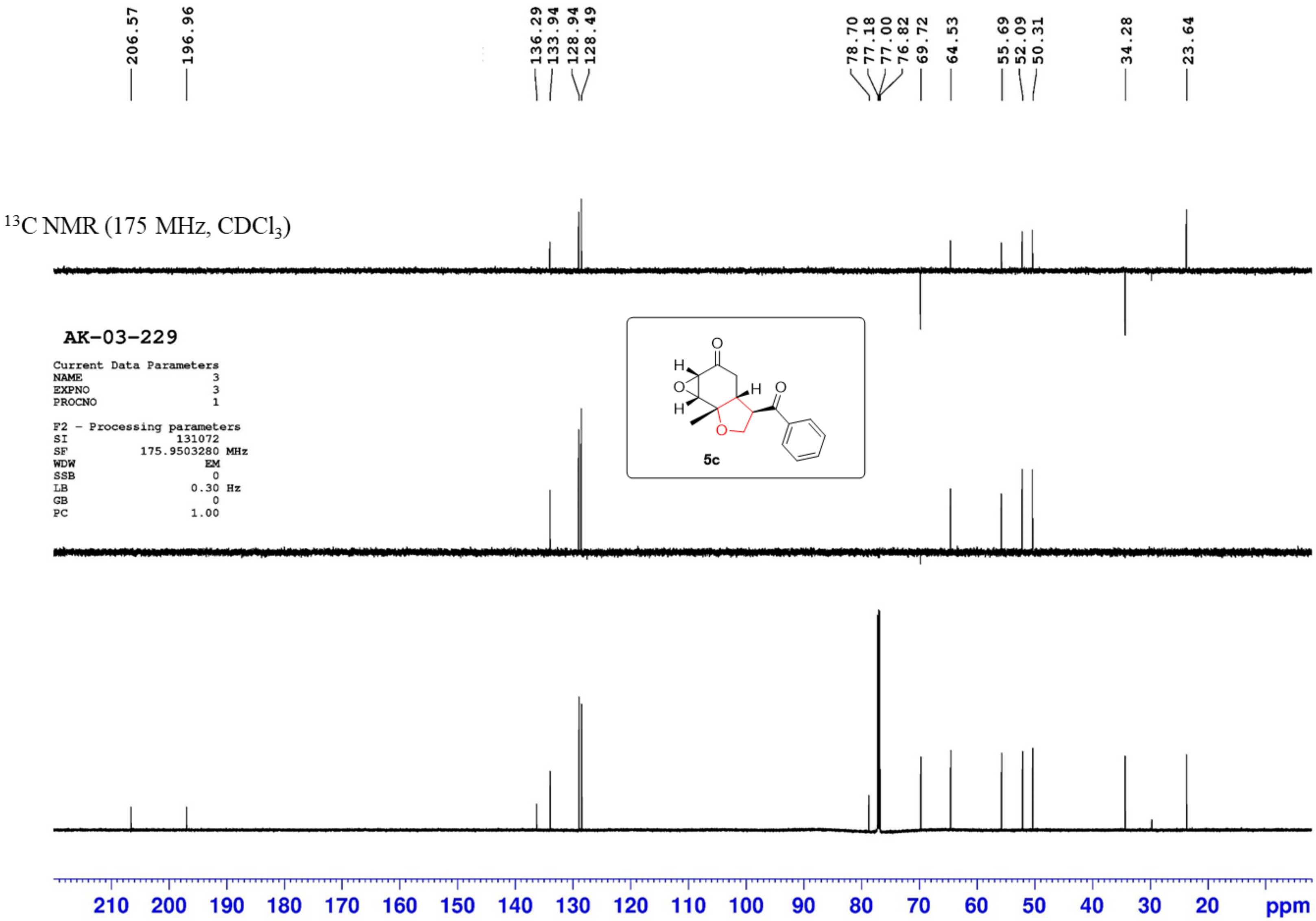
206.57
196.96
136.29
133.94
128.94
128.49
78.70
77.18
77.00
76.82
69.72
64.53
55.69
52.09
50.31
34.28
23.64
^{13}C NMR (175 MHz, CDCl$_3$)
AK-03-229
Current Data Parameters
NAME 3
EXPNO 3
PROCNO 1
F2 - Processing parameters
SI 131072
SF 175.9503280 MHz
WDW EM
SSB 0
LB 0.30 Hz
GB 0
PC 1.00
5c
210 200 190 180 170 160 150 140 130 120 110 100 90 80 70 60 50 40 30 20 ppm

AK-03-229

Sample Name:
AK-03-229
Data Collected on:
Varian-NMR-vnmrs700
Archive directory:

Sample directory:

FidFile: AK-250919-03-229-H

Pulse Sequence: PROTON (s2pul)
Solvent: cdcl3
Data collected on: Sep 19 2025

Temp. 25.0 C / 298.1 K
Operator: peng

Relax. delay 3.000 sec
Pulse 60.0 degrees
Acq. time 2.936 sec
Width 11160.7 Hz
16 repetitions
OBSERVE H1, 699.7422565 MHz
DATA PROCESSING
FT size 65536
Total time 1 min 35 sec

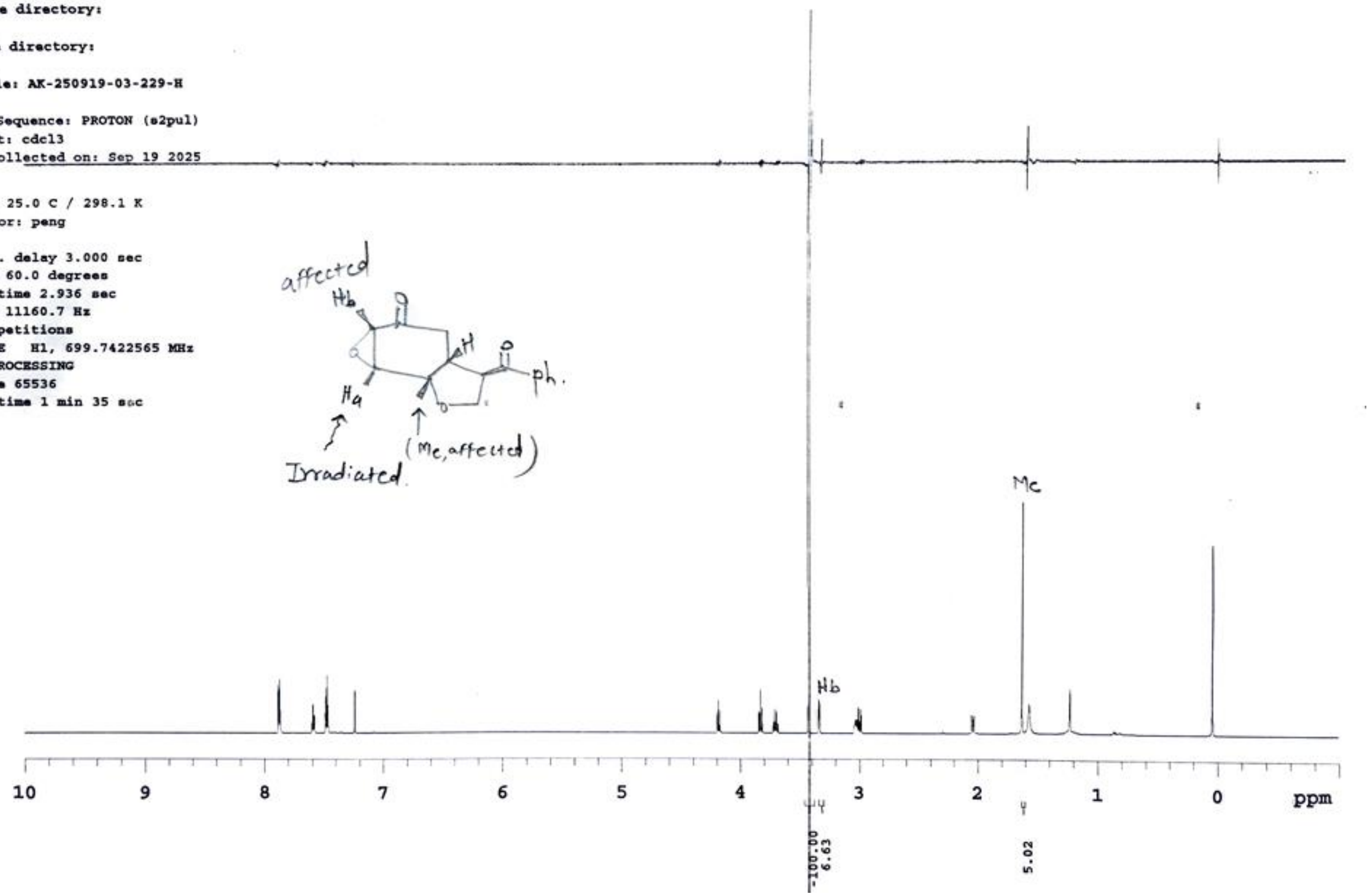

AK-03-229

Sample Name:
AK-03-229
Data Collected on:
Varian-NMR-vnmrs700
Archive directory:

Sample directory:

FidFile: AK-250919-03-229-H

Pulse Sequence: PROTON (s2pul)
Solvent: cdcl3
Data collected on: Sep 19 2025

Temp. 25.0 C / 298.1 K
Operator: peng

Relax. delay 3.000 sec
Pulse 60.0 degrees
Acq. time 2.936 sec
Width 11160.7 Hz
16 repetitions
OBSERVE H1, 699.7422565 MHz
DATA PROCESSING
FT size 65536
Total time 1 min 35 sec

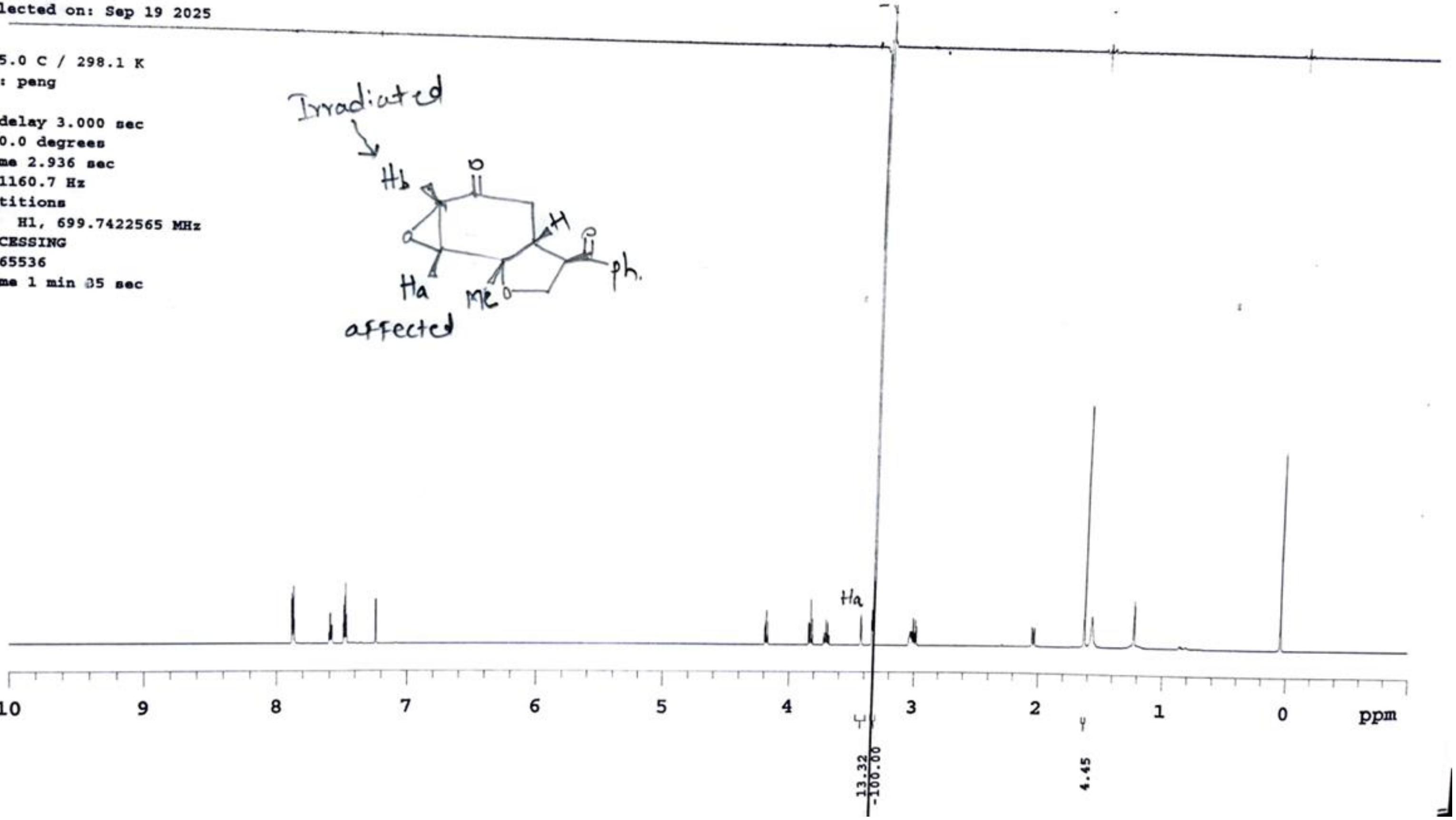

AK-03-229

Sample Name:
AK-03-229
Data Collected on:
Varian-NMR-vnmrs700
Archive directory:

Sample directory:

FidFile: gHSQC

Pulse Sequence: gHSQC
Solvent: cdcl3
Data collected on: Oct 1 2025

Temp. 25.0 C / 298.1 K
Operator: peng

Relax. delay 1.000 sec
Acq. time 0.286 sec
Width 6983.2 Hz
2D Width 38722.2 Hz
8 repetitions
2 x 400 increments
OBSERVE H1, 699.7422552 MHz
DECOUPLE C13, 175.9626450 MHz
Power 40 dB
on during acquisition
off during delay
W40_Cold modulated
DATA PROCESSING
Gauss apodization 0.069 sec
F1 DATA PROCESSING
Gauss apodization 0.002 sec
FT size 4096 x 4096
Total time 2 hr, 21 min

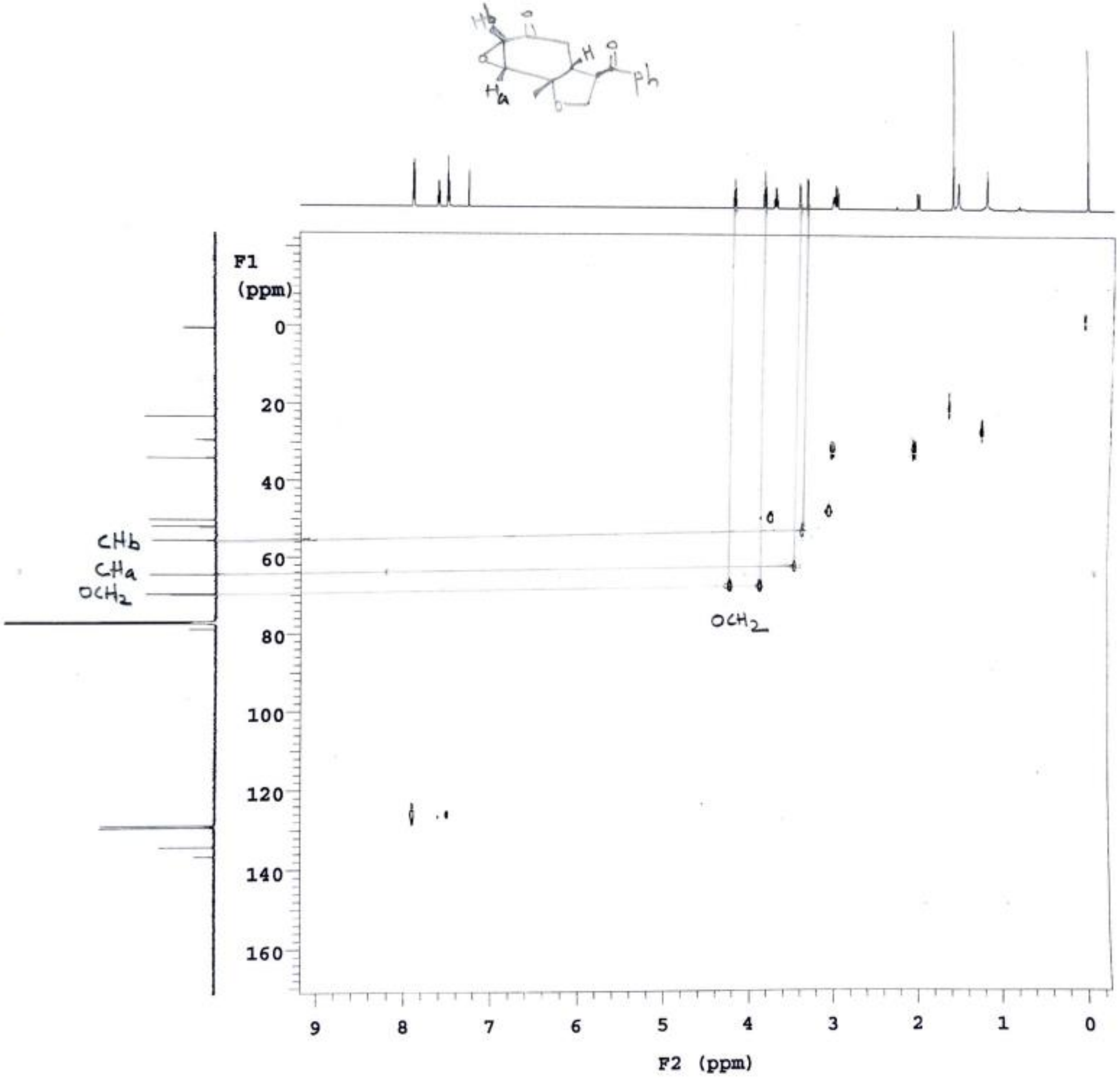

AK-04-19-Ester Substrate

^{1}H NMR (400 MHz, $CDCl_3$)

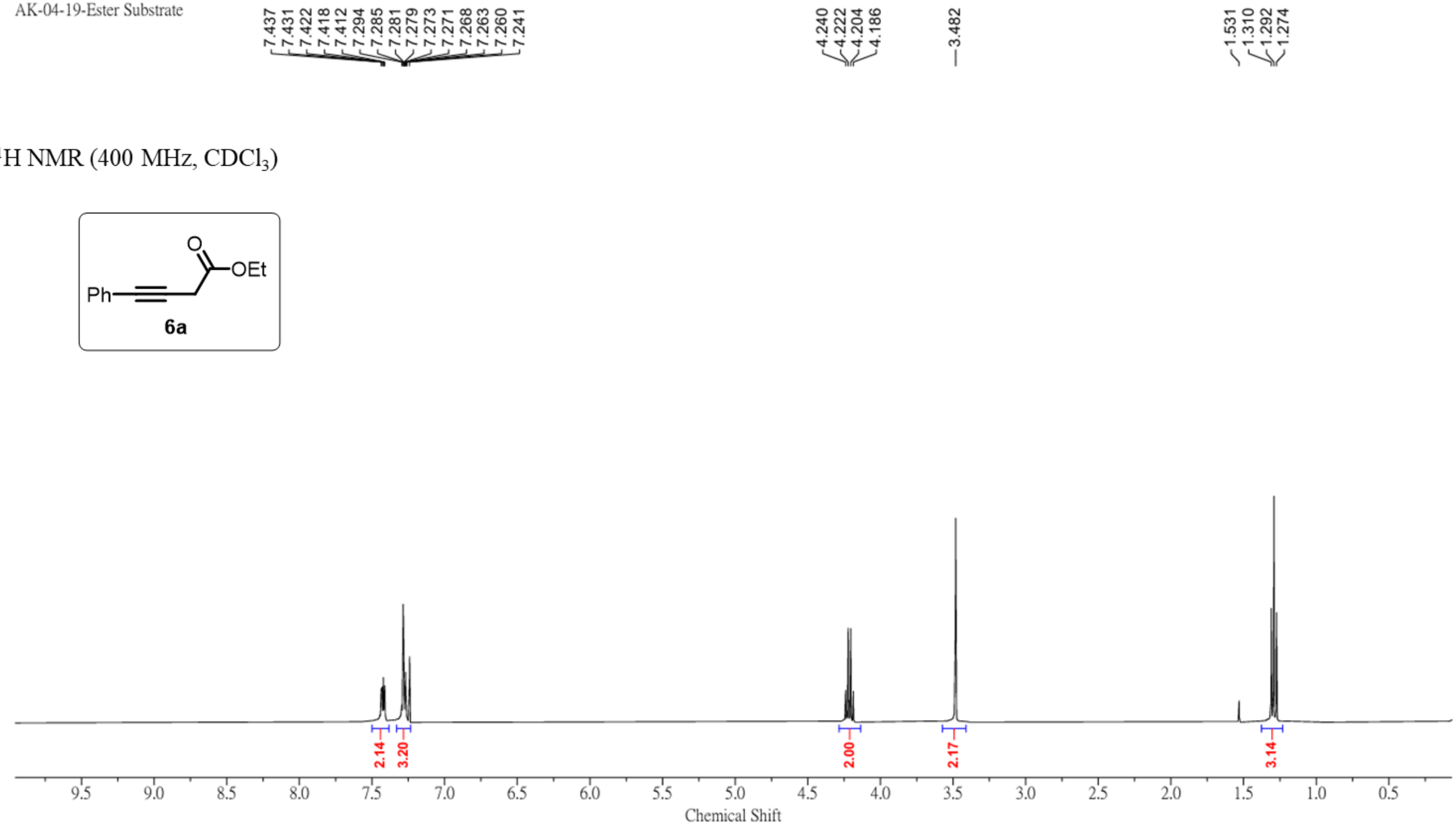

AK-04-19-Ester -C

^{13}C NMR (100 MHz, $CDCl_3$)

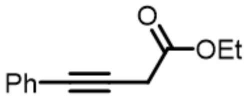

6a

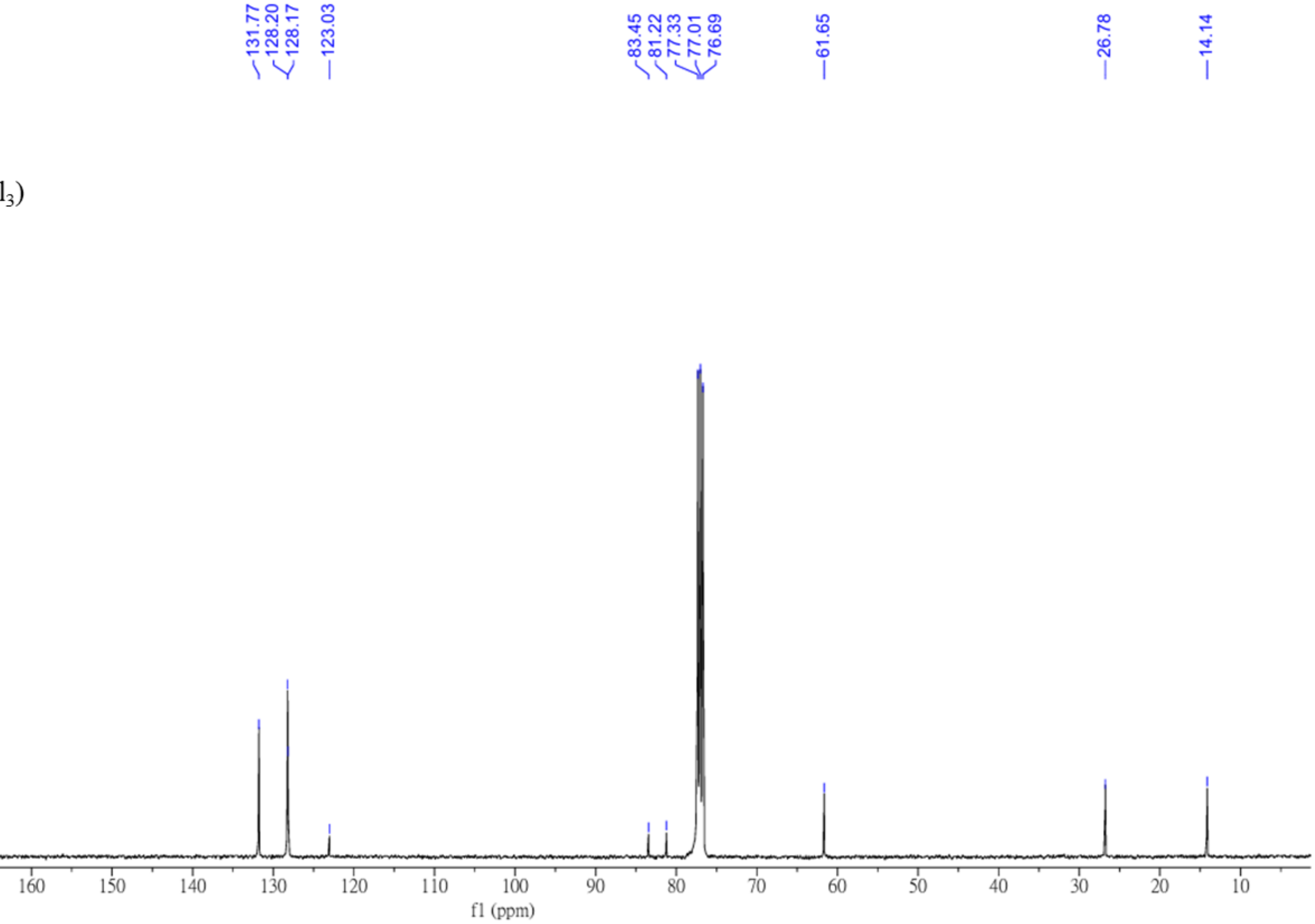

AK-04-19-allene-H

^{1}H NMR (400 MHz, $CDCl_3$)

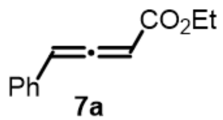

7a

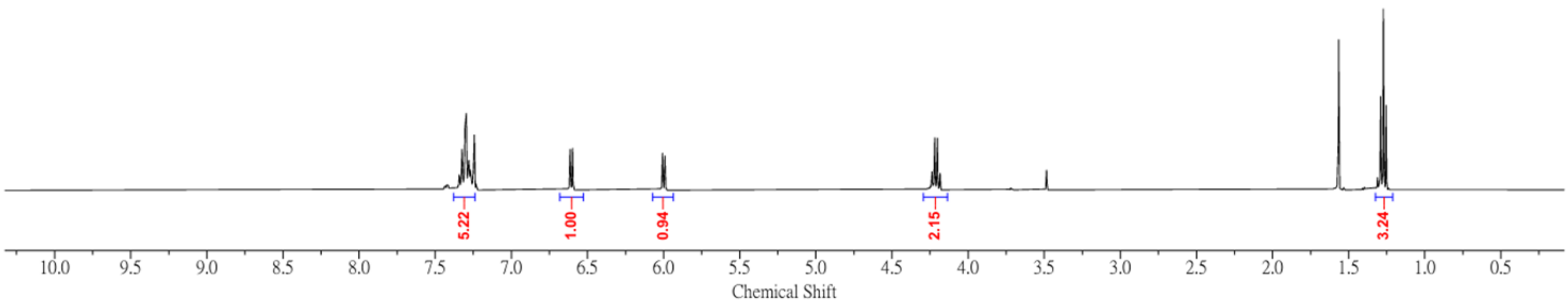

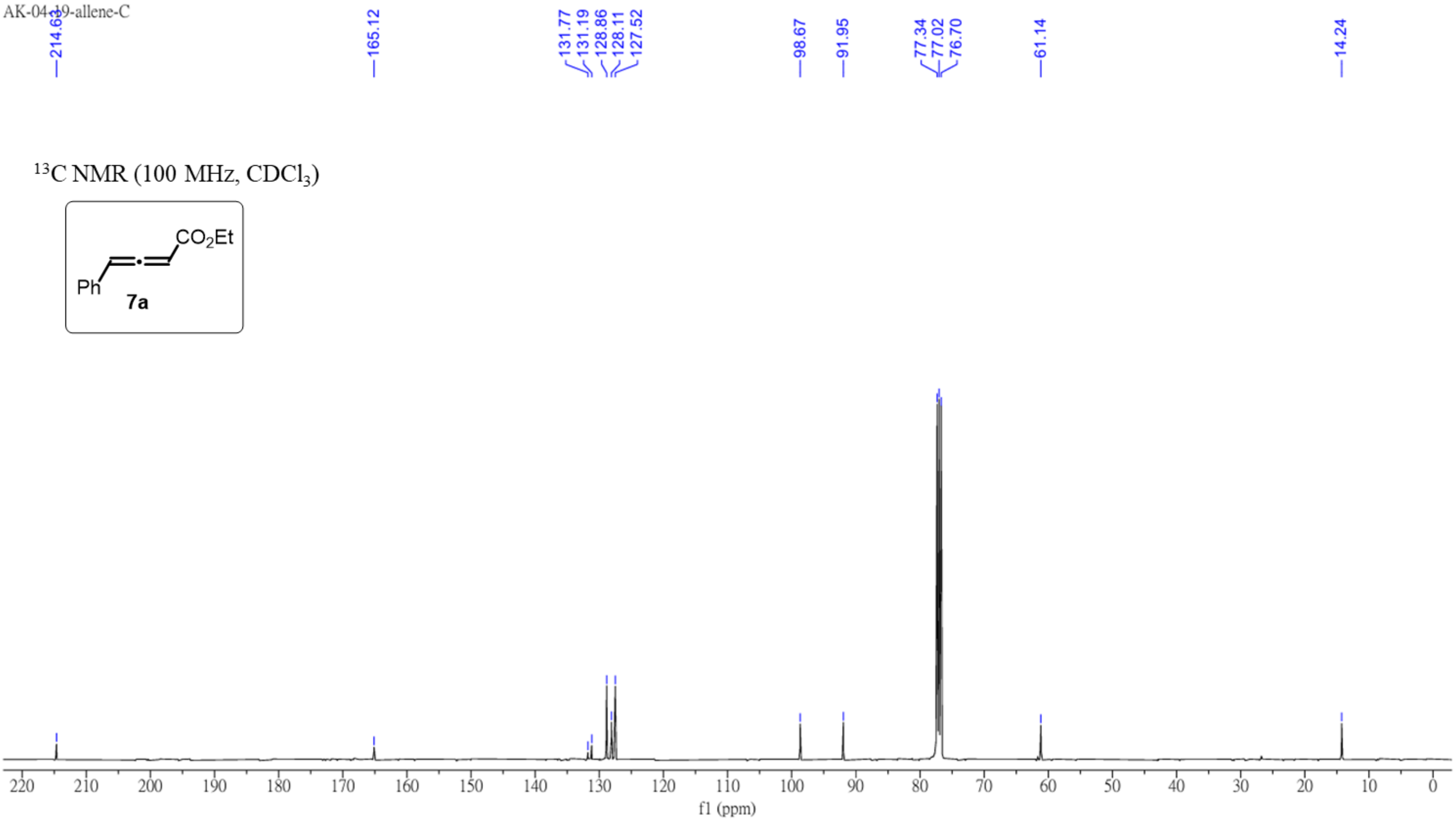

^{13}C NMR (100 MHz, $CDCl_3$)

7a

AK-04-20

^{1}H NMR (400 MHz, $CDCl_3$)

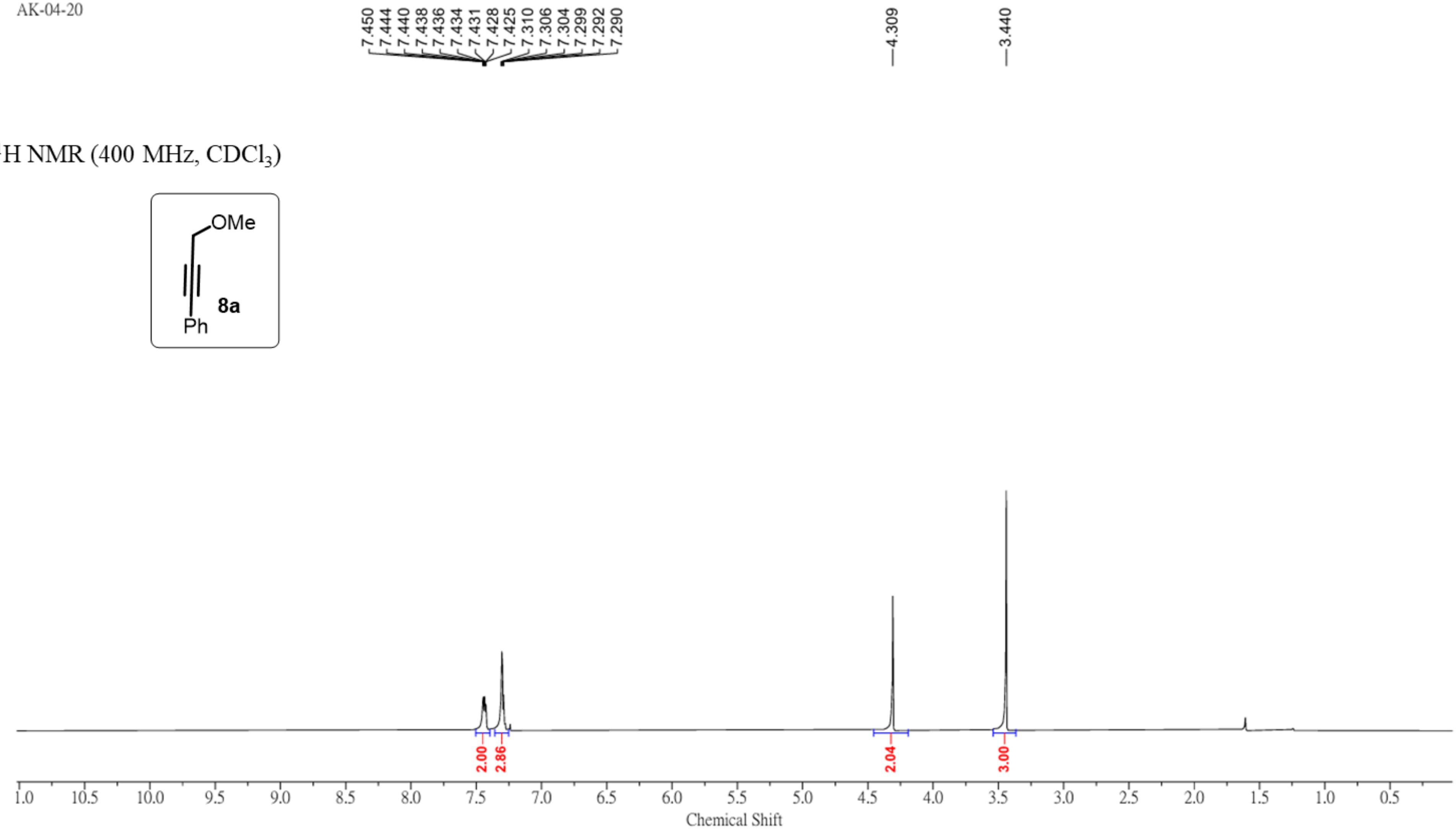

AK-04-20

^{13}C NMR (100 MHz, $CDCl_3$)

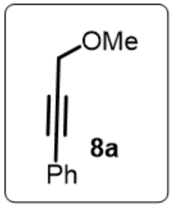

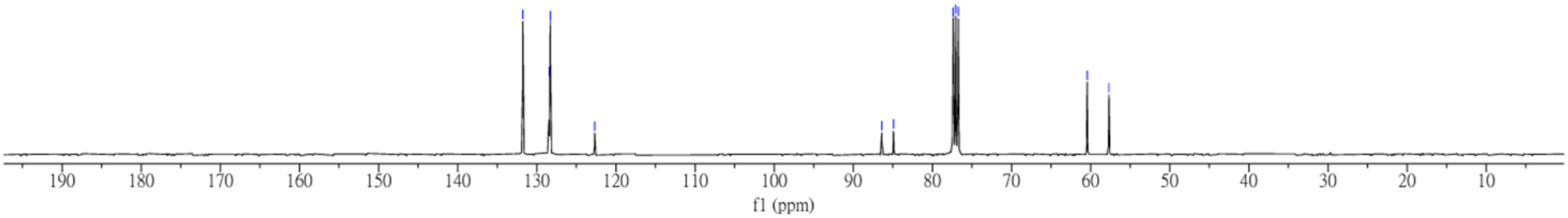

^{1}H NMR (400 MHz, $CDCl_3$)

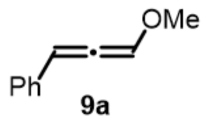

9a

after workup, ^{1}H and ^{13}C instantly recorded

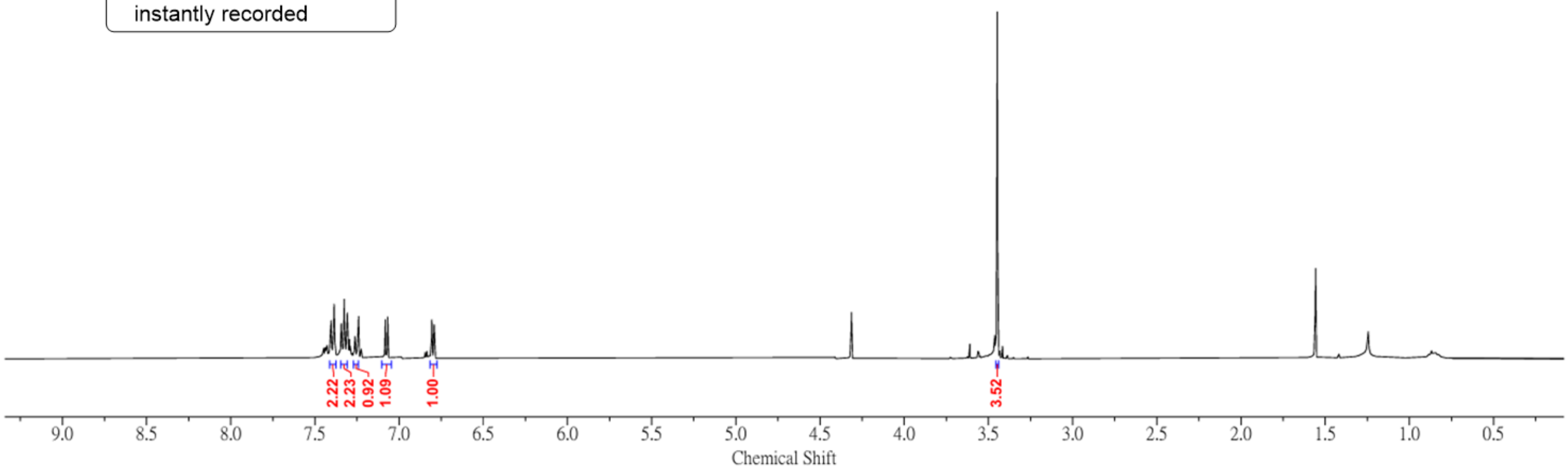

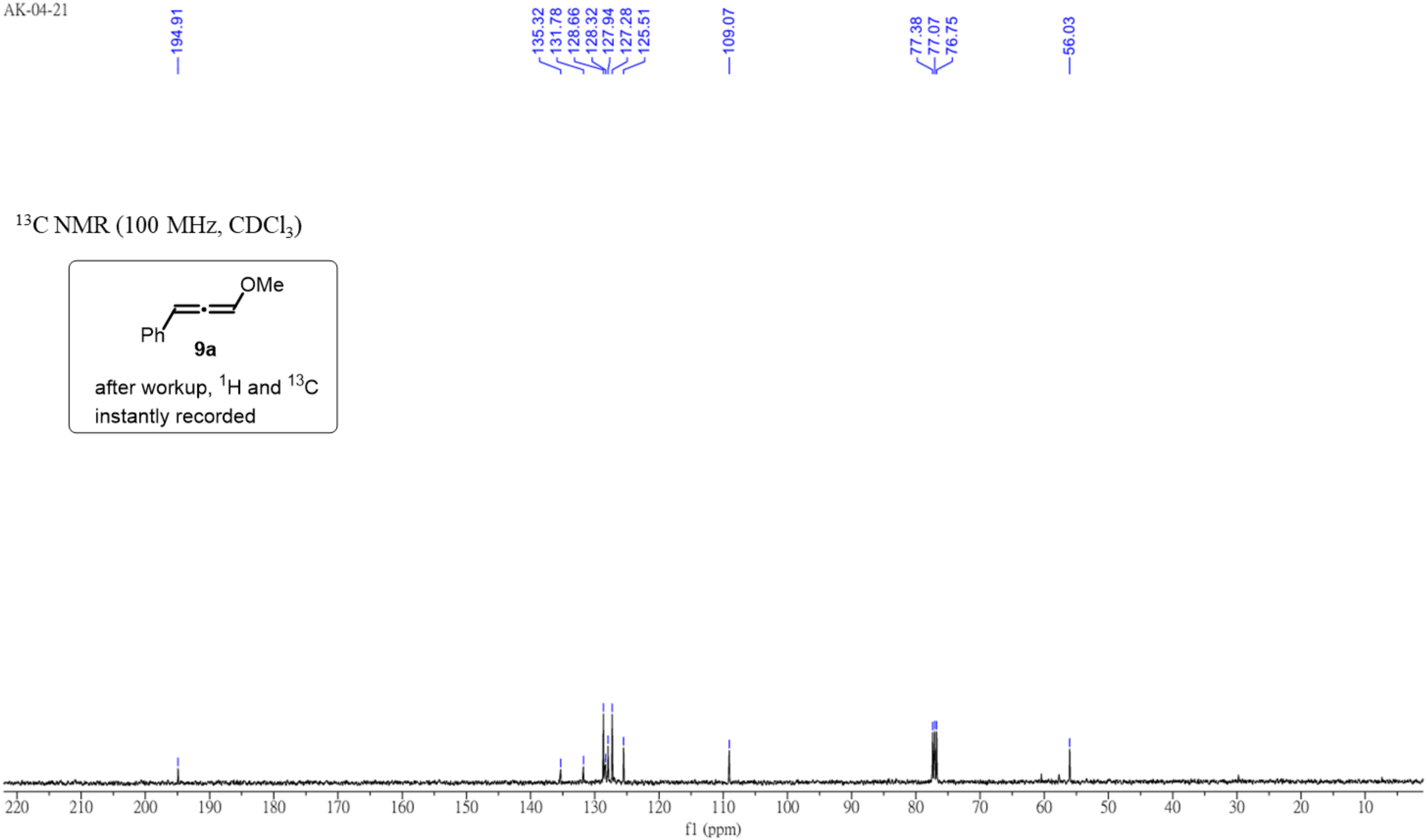

^{13}C NMR (100 MHz, $CDCl_3$)

9a

after workup, ^{1}H and ^{13}C instantly recorded

^1H NMR (400 MHz, $CDCl_3$)

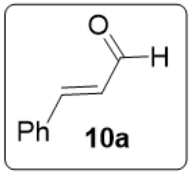

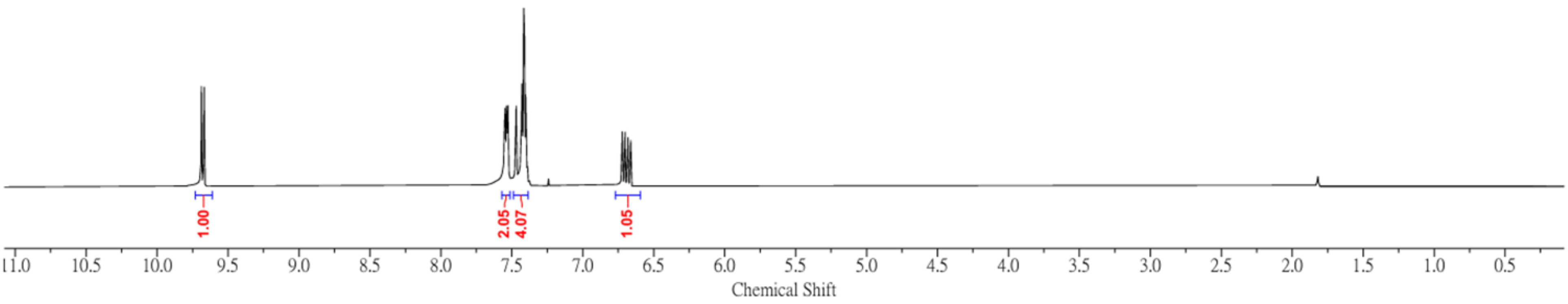

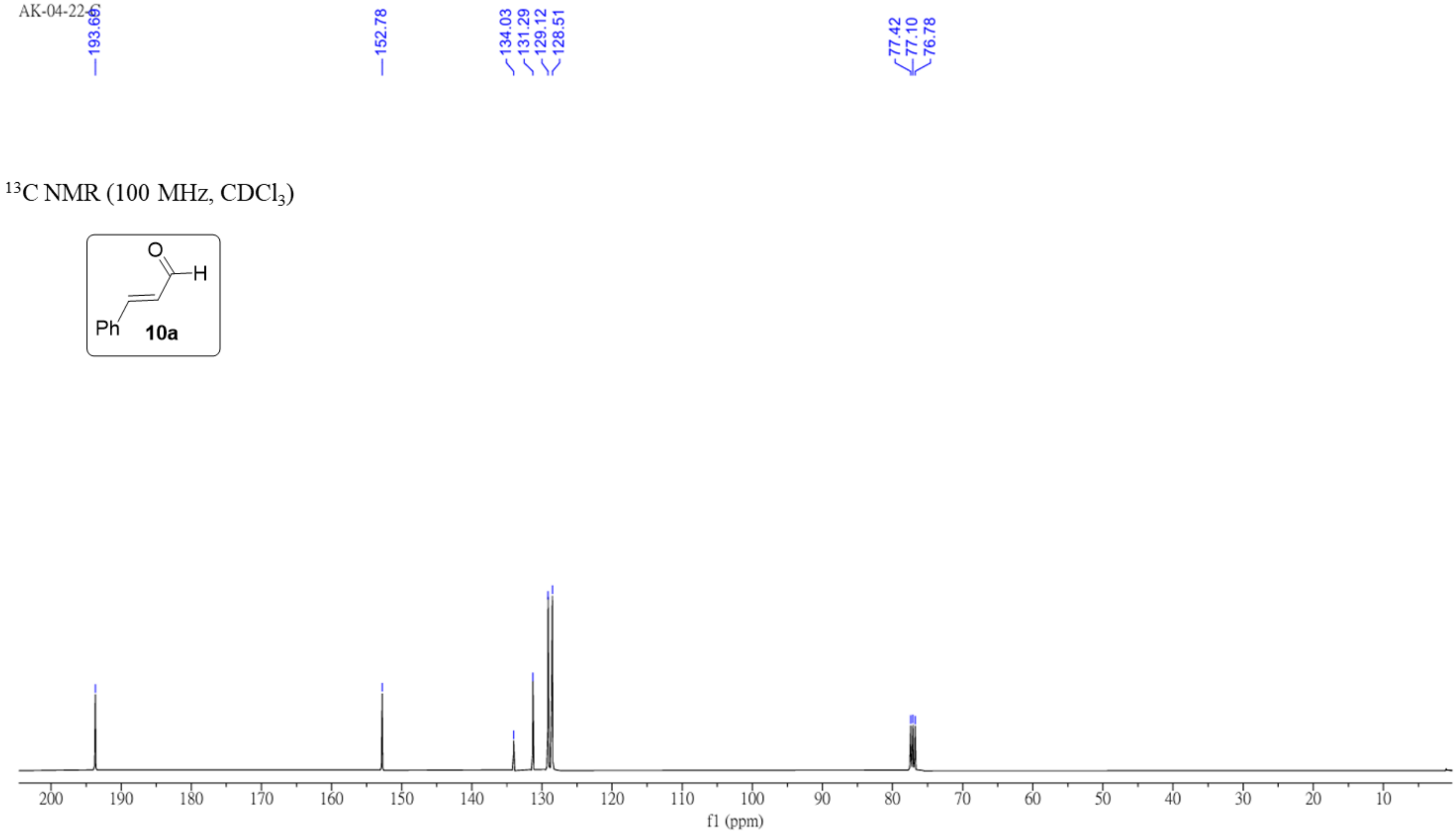
AK-04-22-C
193.69
152.78
134.03
131.29
129.12
128.51
77.42
77.10
76.78
^{13}C NMR (100 MHz, $CDCl_3$)
O
H
Ph
10a
200 190 180 170 160 150 140 130 120 110 100 90 80 70 60 50 40 30 20 10
f1 (ppm)

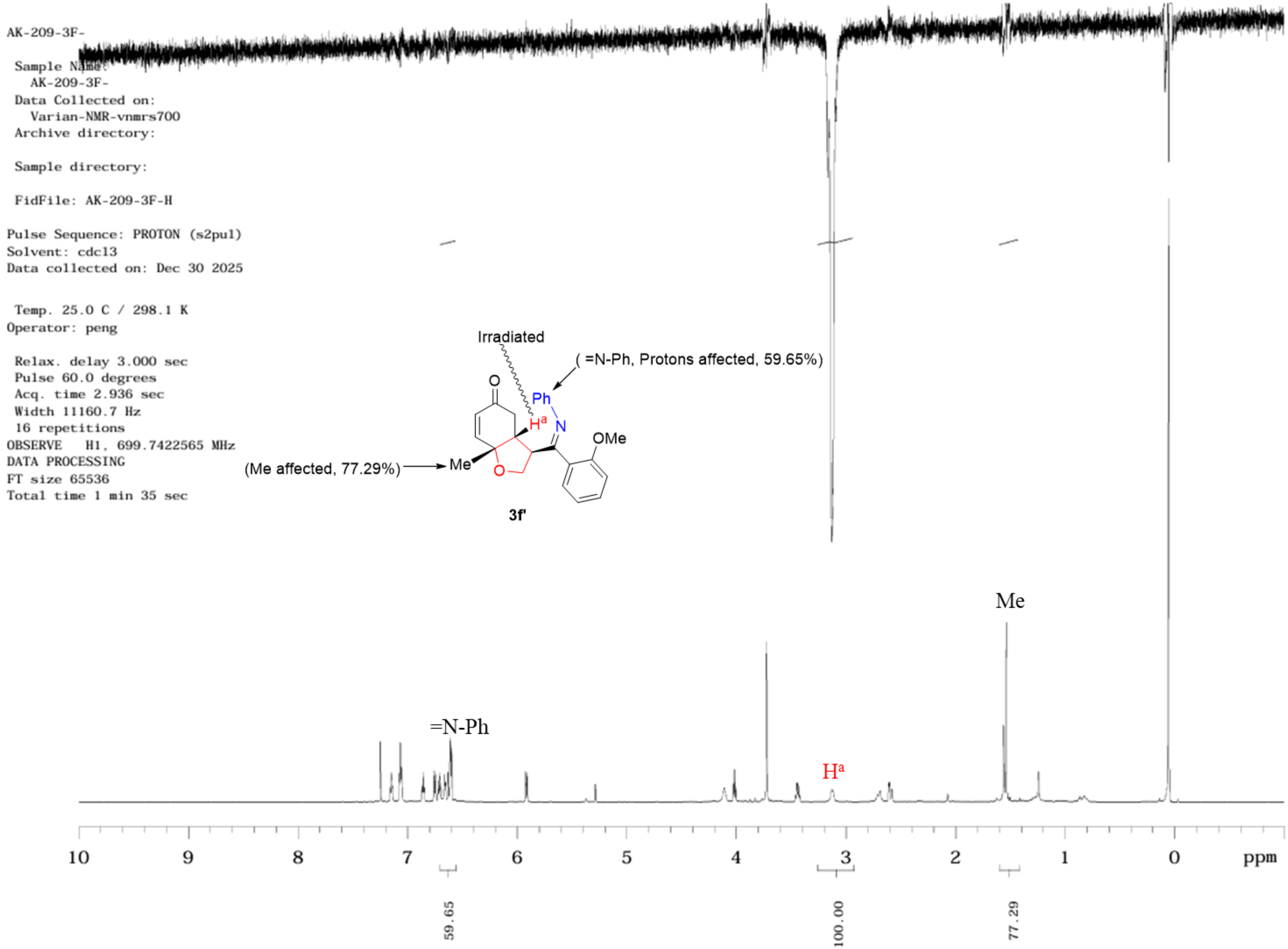
AK-209-3F-
Sample Name:
AK-209-3F-
Data Collected on:
Varian-NMR-vnmrs700
Archive directory:
Sample directory:
FidFile: AK-209-3F-H
Pulse Sequence: PROTON (s2pul)
Solvent: cdcl3
Data collected on: Dec 30 2025
Temp. 25.0 C / 298.1 K
Operator: peng
Relax. delay 3.000 sec
Pulse 60.0 degrees
Acq. time 2.936 sec
Width 11160.7 Hz
16 repetitions
OBSERVE H1, 699.7422565 MHz
DATA PROCESSING
FT size 65536
Total time 1 min 35 sec
Irradiated
(=N-Ph, Protons affected, 59.65%)
(Me affected, 77.29%)
3f'
Me
=N-Ph
Ha
10
9
8
7
6
5
4
3
2
1
0
ppm
59.65
100.00
77.29

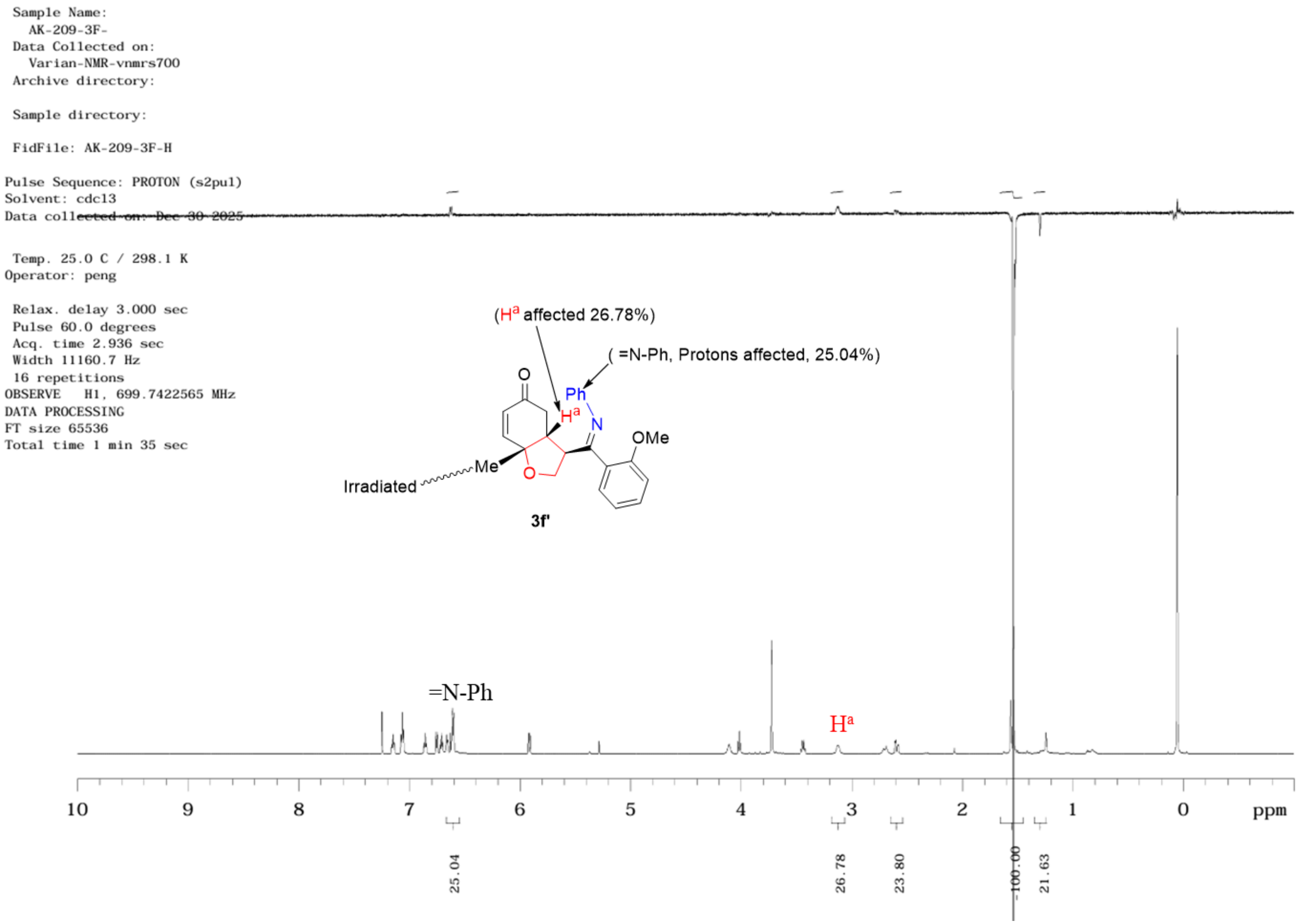
Sample Name:
AK-209-3F-
Data Collected on:
Varian-NMR-vnmrs700
Archive directory:
Sample directory:
FidFile: AK-209-3F-H
Pulse Sequence: PROTON (s2pul)
Solvent: cdcl3
Data collected on: Dec 30 2025
Temp. 25.0 C / 298.1 K
Operator: peng
Relax. delay 3.000 sec
Pulse 60.0 degrees
Acq. time 2.936 sec
Width 11160.7 Hz
16 repetitions
OBSERVE H1, 699.7422565 MHz
DATA PROCESSING
FT size 65536
Total time 1 min 35 sec
(Ha affected 26.78%)
(=N-Ph, Protons affected, 25.04%)
Irradiated
Me
O
Ph
N
Ha
OMe
3f'
=N-Ph
Ha
10
9
8
7
6
5
4
3
2
1
0
ppm
25.04
26.78
23.80
-100.00
21.63
